# Origin of Single-Molecule Reaction Chirality

**DOI:** 10.34133/research.1150

**Published:** 2026-02-24

**Authors:** Chen Yang, Shuyao Zhou, Yilin Guo, Xinmiao Xie, Ju Wang, Yanwei Li, Jingyuan Hu, Linghai Xie, Zhirong Liu, Guangwu Li, Xuefeng Guo

**Affiliations:** ^1^ Beijing National Laboratory for Molecular Sciences, National Biomedical Imaging Center, College of Chemistry and Molecular Engineering, Peking University, Beijing 100871, P. R. China.; ^2^ Department of Chemistry and Biochemistry, University of California, Los Angeles, Los Angeles, CA 90095-1569, USA.; ^3^ Center of Single-Molecule Sciences, Institute of Modern Optics, Frontiers Science Center for New Organic Matter, College of Electronic Information and Optical Engineering, Nankai University, Tianjin 300350, P. R. China.; ^4^ Environment Research Institute, Shandong University, Qingdao, P. R. China.; ^5^ Institute of Chemistry, Henan Academy of Sciences, Zhengzhou 450046, P. R. China.; ^6^ Center for Molecular Systems and Organic Devices, Key Laboratory for Organic Electronics & Information Displays and Institute of Advanced Materials, Nanjing University of Posts & Telecommunications, Nanjing 210023, P. R. China.

## Abstract

The origin of molecular chirality remains an enigma in chemistry, particularly regarding how single-molecule events overcome intrinsic stochasticity to establish population-level chirality. Here, we present a viable strategy for real-time, from-the-beginning single-molecule trajectory monitoring of asymmetric evolution from a single initial molecule with single-event resolution, allowing direct observation of spontaneous mirror symmetry breaking in a single-molecule Diels–Alder reaction system. We monitor the asymmetric evolution in real time using the chirality-induced spin selectivity effect. This approach enables the capture of initial symmetry breaking at the single-molecule level and the identification of the excess-compensation mechanism. In addition, the introduction of an external electric field to the symmetry-breaking species enables universal asymmetric synthesis without the need for a catalyst. The increase in the number of molecules leads to symmetry breaking, which is also contingent on the coupling with the external environment. This work deepens our understanding of the molecular principles underlying the origin of life and has many implications for precise chiral synthesis and drug design.

## Introduction

The homochiral architecture of life—from helical DNA to l-amino acids and d-carbohydrates—represents a universal signature demanding fundamental physical explanation. Decoding the emergence from single-molecule chirality to macroscopics holds transformative potentials across domains: illuminating specific recognition in biological processes [1,2], enabling unique performances in material applications [3,4], and advancing catalyst-free chirality amplification [5]. Current hypotheses include polarized light irradiation [6], spontaneous mirror symmetry breaking (SMSB) [7,8], extraterrestrial delivery [9], and magnetochiral effects [10]. Crucially, all paradigms must resolve the single-molecule uncertainty: how achiral precursors overcome quantum fluctuations to establish directional chirality amplification. For example, the SMSB, a concept supported by notable phenomena such as the Soai reaction [11,12] and Viedma deracemization [13,14], involve multiple autocatalytic steps from the initial symmetric breaking. The balance between the entropy of chemical reactions and environmental entropy flow leads to a non-equilibrium steady state [8]. Unveiling the full amplification mechanism of the initial symmetry breaking necessitates real-time monitoring of the symmetry evolution at single-molecule resolution, which will clarify how ensemble chirality accumulates from single-molecule chirality.

Single-molecule detection is a rapidly developing concept that consists of a variety of techniques, including super-resolution imaging [15,16], scanning probe microscopy [17–19], nanocavities or nanopores [20,21], single-molecule junctions [22,23], and so on [24]. These techniques characterize the weak optical, mechanical, and electrical signals of individual molecules. A range of intrinsic properties that are often obscured in ensemble measurements, such as molecular recognition [25], configuration changes [26,27], quantum effects [28,29], and reaction mechanisms [30,31], have been studied through single-molecule detection. Therefore, by tracing individual molecules and demonstrating the emergent complexity that arises as the scale increases from single molecule to ensemble, it should be possible to monitor the evolution of symmetry breaking.

The dynamics of molecules during chemical reactions can be visualized by anchoring a single molecule between nanogap electrodes and recording the current signal [32]. The interaction between the anchored molecule and surrounding free molecules allows emergent properties, such as enantiomeric excess (ee), to be characterized [33]. Among various molecular junction techniques, graphene–molecule–graphene single-molecule junctions (GMG-SMJs) stand out for their in situ monitoring capability [23]. GMG-SMJs provide stable electrodes and well-defined molecule–electrode coupling, enabling them to withstand complex chemical reaction conditions. The fixed electrodes and molecules eliminate the need for repeated junction formation, facilitate in situ monitoring, and meet the requirements for low-temperature measurements, which can minimize the effects of reaction noises during single-molecule asymmetric evolution.

**Fig. 1.**
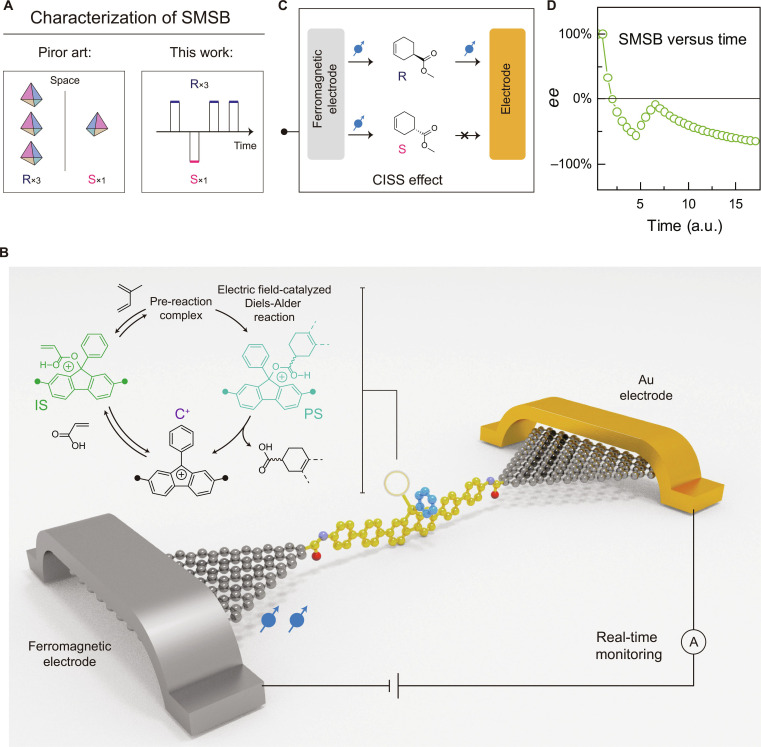
Study of emergent chirality at single-molecule scale. (A) The traditional strategy of studying SMSB is to detect the excess of chiral enantiomers in the macroscopic system at the spatial scale. The strategy in ​​this work is to monitor the evolution of reaction symmetry in real time with single-event resolution. (B) Schematic diagram of real-time monitoring of SMSB via single-molecule junctions. The molecular bridge provides the substrate binding site for monitoring the Diels–Alder reaction, accompanied by forming a chiral carbon. (C) Schematic diagram of using the CISS effect to identify chirality. Enantiomers have different filtering effects on spin-polarized electrons. (D) The ee values versus time, indicating SMSB at the single-molecule level.

In this work, we shift the research paradigm from traditional macroscopic characterization of ee to real-time observation that focuses on individual molecules (Fig. 1A), using GMG-SMJs. In detail, we designed a cascade composed of 2 parts: irreversible Diels–Alder cycloaddition and the corresponding species that can associate/dissociate with the 9-phenyl-9-fluorenyl cation (C^+^) center on the SMJ (Fig. 1B). Association/dissociation of the C^+^ center and carboxyl moiety, including the acrylic acid intermediate state (IS), pre-reaction charge transfer complexes (CTs), and corresponding product states (PSs), is shown in Fig. 1B. This approach allows us to track the evolution process and obtain a series of key insights, including potential chiral intermediates, the reaction trajectories of symmetry breaking, stereoselective interactions between molecules, the transfer and amplification mechanisms of chirality, and the evolution of asymmetry at the molecular scale.

## Results and Discussion

### Device fabrication and characterization

To fabricate GMG-SMJs, a piece of graphene on a chip was etched with a dashed-line pattern using oxygen plasma. The resulting nanogap, featuring carboxyl terminals, facilitates the covalent bonding of a molecular bridge with amino terminals [34]. Details of the device preparation procedure are provided in Fig. S1. Incorporation of the molecular bridge was confirmed by comparison of current–voltage (*I*–*V*) scans before (no response) and after (response to some extent) molecular connection (Figs. S2 and S3). The only-one-molecule connection was also supported by the super-resolution imaging and single-molecule localization (Fig. S4). Due to the formation of a C^+^ from the central 9-phenyl-9-fluorenol on the molecular bridge under acidic conditions [35], the reversible transition between C^+^ and its corresponding acrylate enables the characterization of reactions involving acrylic acid, such as the Diels–Alder cycloaddition (vide infra). Here, the Diels–Alder cycloaddition between acrylic acid and isoprene was studied, which is accompanied by the formation of a chiral carbon (inset in Fig. 1B and C). To assess molecular chirality, ferromagnetic metal leads were introduced for spin injection. Real-time current changes in chirality were monitored via the chirality-induced spin selectivity (CISS) effect [36] (Fig. 1C), which means that the chiral structure can impose a filtering effect on spin-polarized electron transport and thus can be identified. By recording the chiral products over time, we quantified the accumulated number of enantiomers, allowing the corresponding ee value to be calculated (Fig. 1D). Therein, ee = (*N*_R_−*N*_S_)/(*N*_R_+*N*_S_), where *N*_R_ ​and *N*_S_ ​are accumulated counts of *R* and *S* enantiomers extracted from event-resolved trajectories.

### Visualization of the reaction evolution trajectories

**Fig. 2.**
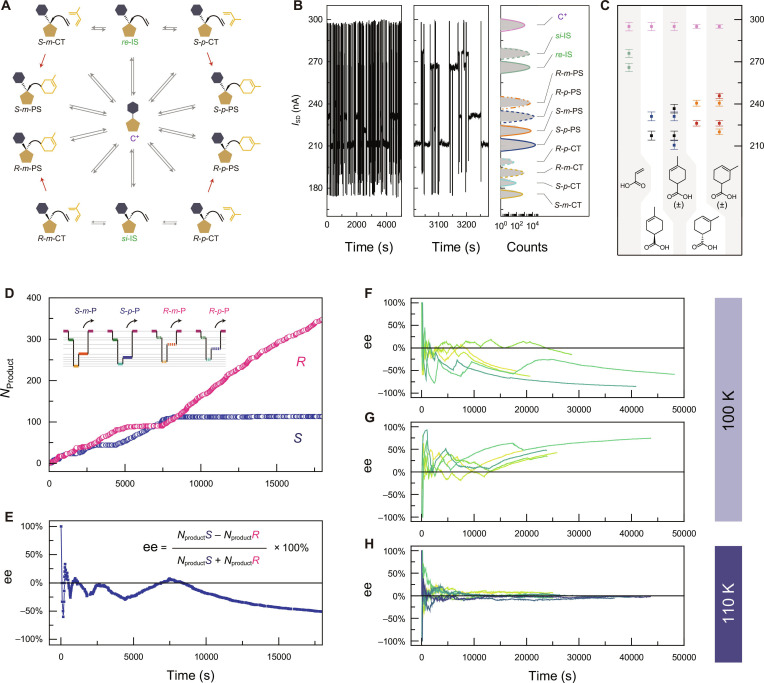
Monitoring of the Diels–Alder reaction at single-molecule resolution. (A) Detected species and corresponding conversion relationship in the whole reaction scenario. (B) *I*−*t* curves of the Diels–Alder reaction at 100 K and 1 V, enlarged image, statistical results, and assignments of the states. The histogram illustrates the thermodynamic properties at equilibrium. Notably, there is no obvious chiral preference, as both *R*-PS and *S*-PS exhibit nearly equivalent occupancy. In addition, the preference of products with *para* configuration (*p*-PS) was observed, which is consistent with the results obtained from computational simulations (Fig. S5). (C) Assignment of conductance states by ex situ synthesis with added standard samples. (D) Plot of cumulative chiral product number versus time. Insets show typical current curves for different chiral products generated. (E) Plot of the corresponding ee value versus time. (F and G) Multiple reaction evolution trajectories indicate a break in the symmetry of the reaction, resulting in *S*- (F) or *R*- (G) dominated products. (H) The evolution trajectories of several reactions indicate that the reactions tend to racemize at 110 K. *m*: *meta*-configuration. *p*:*para*-configuration. CT: pre-reaction charge transfer complex salt state. P: product. PS: product state. *re* and *si* represent the *re* and *si* face of the C^+^, respectively. Evolution trajectories derived from 5 independent single-molecule devices.

The Diels–Alder cycloaddition between acrylic acid and isoprene was monitored through the reversible association–dissociation of carboxylic acid with a carbocation molecular bridge center in a trifluoroacetic acid solvent (Fig. 2A and Fig. S5). To further minimize the noise in both the reaction system and electric circuit, we conducted the reaction at a low temperature of 100 K. We also considered the local electrical heating of the molecular junction during measurements, which may serve as an energy source for the dissipation system and enable continuous synthesis at molecular junction sites at low temperatures [37,38]. Under a constant bias voltage of 1 V, we observed a current signal exhibiting multiple state switching (Fig. 2B). Including the uncomplexed C^+^ state of the molecular bridge, a total of 11 conductance states were identified based on the statistical histogram (Fig. 2B, right panel). These conductance states originated from adding different controlled species, including carboxylic acid, chiral products with varying regioselectivity, and racemic products (Fig. 2C). By comparing the absolute current amplitudes, we assigned 4 PSs with distinct chirality and regioselectivity. The detailed assignments for all species involved in the regioselective and chiral pathways are provided in the Supplementary Materials (Figs. S6 to S34). Two association states between acrylic acid and the molecular bridge were observed, corresponding to a pair of mirror-image configurations (*re*-IS and *si*-IS). Detailed theoretical simulation is provided in the Supplementary Materials. The remaining 4 states were assigned to the CTs, identified as an inevitable intermediate, supported by the time sequence analysis (Figs. S14 and S22 to S24). The assignments were also supported by concentration-dependent measurements (Figs. S7 to S9), inelastic electron tunneling spectra (Figs. S10 to S13), a theoretical study of the transmission spectra and calculated *I*−*V* curves (Figs. S15 and S16), the tracking with macroscopic gas chromatography–mass spectrometry (GC-MS) (Figs. S17 to S20), and optical characterizations of the single-molecule products (Figs. S31 to S34). The 4 typical reaction cycles were provided in the inset of Fig. 2D; these cycles generated 4 stereochemical products.

The calculated energy profile further supports the smooth progression of this reaction under an electric field (Figs. S5A and S35 to S50). Specifically, as the electric field strength increases—regardless of direction—the energy barrier for the cycloaddition reaction decreases, facilitating chemical reactions at low temperatures. In comparison with the switching among CT states at 0.1 V, the cycloadditions were initiated with the application of a 1.0-V bias. The resulting chiral products were quantified according to a specific cycle of electric signals (Fig. 2D). The 2 configurations alternated in dominance, ultimately leading to the *R* configuration becoming predominant, indicating symmetry breaking. The corresponding ee values were obtained, showing initial +100%, followed by oscillation around 0%, and finally a continued growth, exceeding −50% (Fig. 2E). Additional evolution trajectories at 100 K are presented in Fig. 2F and G, illustrating the SMSB, ultimately dominated by either *R* or *S* configurations. Therefore, complete racemization, particularly at the single-molecule scale, is unattainable and the direction of asymmetric evolution remains random. This SMSB phenomenon is sensitive to the external environment. When the temperature was raised to 110 K, we observed a gradual disappearance of symmetry breaking in the reaction, indicating that the energy difference of the symmetry breaking is masked by the noise within the reaction system (Fig. 2H and Fig. S51). Another example is the vanished stereoselectivity by the mechanistic crossover of the Diels–Alder reaction at high temperature [39]. The *cis*-cycloaddition rule was broken by the stepwise mechanism (Figs. S52 to S56).

### Mechanism of SMSB

**Fig. 3.**
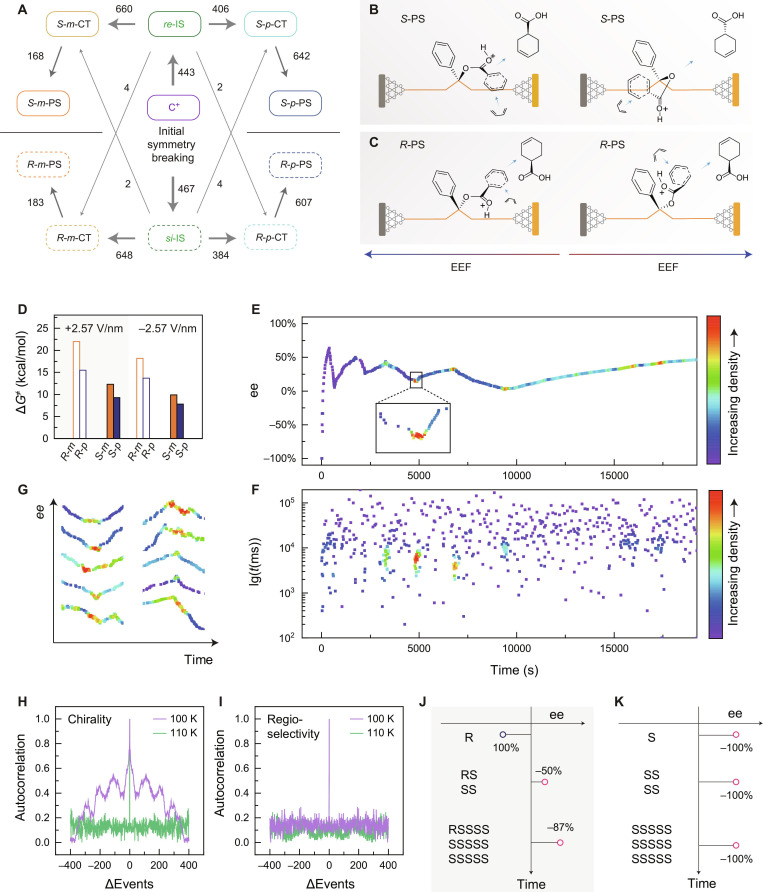
Mechanism of SMSB of the Diels–Alder reaction. (A) Statistical conversion relationship mapping among C^+^, IS, CTs, and PSs with 1-V bias and 100-K and +2-T magnetization. (B) Reaction scenarios at the *re* face and at different directions of the EEF. (C) Reaction scenarios at the *si* face and at different directions of the EEF. (D) For a given IS configuration, the energy barriers of various reaction pathways under different electric fields. (E) Plot of the ee value versus time. The density on time scale is represented by color. Inset: Enlarged image of the ee transition point. (F) Plot of the corresponding dwell time versus time. The density on time scale is represented by color. (G) Density map of transition points of multiple reaction evolution trajectories. (H) Autocorrelation function of the dwell time of chiral products at different temperatures. (I) Autocorrelation function of the dwell time of products involving regio-selectivity at different temperatures. (J) Proposed excess-compensation mechanism of the chiral amplification. (K) Conventional concept of the chiral amplification.

The reaction pathways were extracted, and the statistical results are provided in Fig. 3A. A strong correlation is found between the configuration of IS and the chirality of the CT states. Regardless of the regio-selectivity or the direction of the bias voltage, the *R*-CT states only originated from the IS of ~276 nA (defined as the *si*-IS), whereas the *S*-CT was generated from the IS of ~265 nA (defined as the *re*-IS). The dipole of the reaction moiety in relation to the external electric field (EEF) was studied theoretically, with the dominant conformations illustrated in Fig. 3B and C. Due to the alignment of the unfixed carboxylic dipole on the *si*-IS along the EEF, the dienophile exhibited a specific preference for the addition direction of isoprene. More importantly, the subsequent cycloaddition transition states (TSs) in this pathway were found to be more stable (Fig. 3D and Figs. S57 and S58), resulting in a single-chirality *R*-PS. The mirrored *re*-IS would induce a flipping of chirality. Subsequently, symmetry breaking can be achieved through several oscillations at the single-molecule level. The chirality originating from the IS configuration allows the continuous chiral amplification at low temperatures. At room temperature, the energy fluctuations are too large to realize symmetry breaking. Conversely, the continuous generation of chiral products is difficult at low temperatures. In this work, we found that symmetry breaking does not occur during the cycloaddition; rather, it is determined much earlier, even before the formation of the pre-reaction complex. As such, minimal energy is needed to sustain symmetry breaking at low temperatures. This finding is consistent with the chiral origins observed in interstellar chemistry [40].

### Mechanism of chirality amplification

We also observed that the initial chirality was opposite to that being ultimately stabilized among the various evolutionary trajectories (Fig. 2F and G), prompting consideration of the correlation of reaction events. A typical evolutionary trajectory of the ee value is illustrated in Fig. 3E, with the density of reaction events over time indicated by a color bar. In the initial oscillations, transitions of the ee value were accompanied by an increase in the frequency of reaction events, a trend supported by the corresponding dwell time statistics (Fig. 3F). Additional trajectories presented in Fig. 3G further corroborate this observation. Considering the exothermic nature of the cycloaddition reaction, persistent symmetry breaking may be disrupted by the thermal noise generated during the reaction, causing the system to spontaneously transfer to a racemic state of lower energy until the excess of the other enantiomer is reached. Notably, the system becomes stable after several oscillation cycles and sufficient to counteract the thermal noise, resulting in persistent symmetry breaking (Figs. 2F and G and 3E). Furthermore, we quantified the autocorrelation of the dwell time (Fig. 3H), revealing a pronounced chiral memory effect at 100 K, which is indicative of an autocatalytic process. However, this autocorrelation rapidly disappeared as the temperature increased to 110 K, transforming into a completely random process (Fig. 3H). We also characterized the autocorrelation of regioselectivity and found that there was no obvious memory effect at different temperatures (Fig. 3I), underscoring the uniqueness of chirality as a stereochemical property. Consequently, we propose an excess-compensation mechanism to explain the observed symmetry breaking. Specifically, once a small (but statistically significant) excess of enantiomers (i.e., the initial few molecules) is produced in the system, an enantiomer compensation mechanism is triggered, reducing the overall energy and initiating the racemization. The subsequent production of the opposite enantiomer diminishes the significance of the initial ee value of 100%, thereby stabilizing the system [8]. The generation of multiple opposite enantiomers at this stage begins to expand the enantiomeric distribution, leading to symmetry breaking within the system (Fig. 3J). In comparison with the direct amplification of the initial symmetry breaking (Fig. 3K), symmetry breaking with oscillations of ee values appears to be energetically more favorable.

### On-line asymmetric synthesis

Investigating the single-molecule SMSB can guide us toward achieving universal asymmetric synthesis. Essentially, if we could observe the reaction trajectories, including the multiple pathways and symmetry breaking occurring during the reaction, we could selectively screen for the desired reaction pathway by artificially choosing the corresponding key intermediates or TSs (Figs. S59 to S61). Note that it is not the electric field that favors the formation of one enantiomer, which necessitates the rigid constraining of the dienophile. Instead, it is the selective opening of the desired reaction pathway at low temperatures based on the existing pre-reaction complex. Enrichment of the pre-reaction complex cannot affect the final selectivity of the following asymmetric reaction paths with high-energy TSs according to the Curtin–Hammett principle at the macroscopic scale. However, these TSs showed here an obvious negative correlation with the increased electric field (Fig. S5), which allows for selection of the desired product through capturing the required pre-reaction complexes in the junction. This approach could be termed as “on-line asymmetric synthesis”. However, considerable challenges remain in macroscopic synthesis, particularly regarding time and spatial resolution, as well as the techniques required to control the reaction. Given the sensitivity of asymmetric systems to external energy, careful design of the time interval, amplitude, and width of external input pulses is essential.

**Fig. 4.**
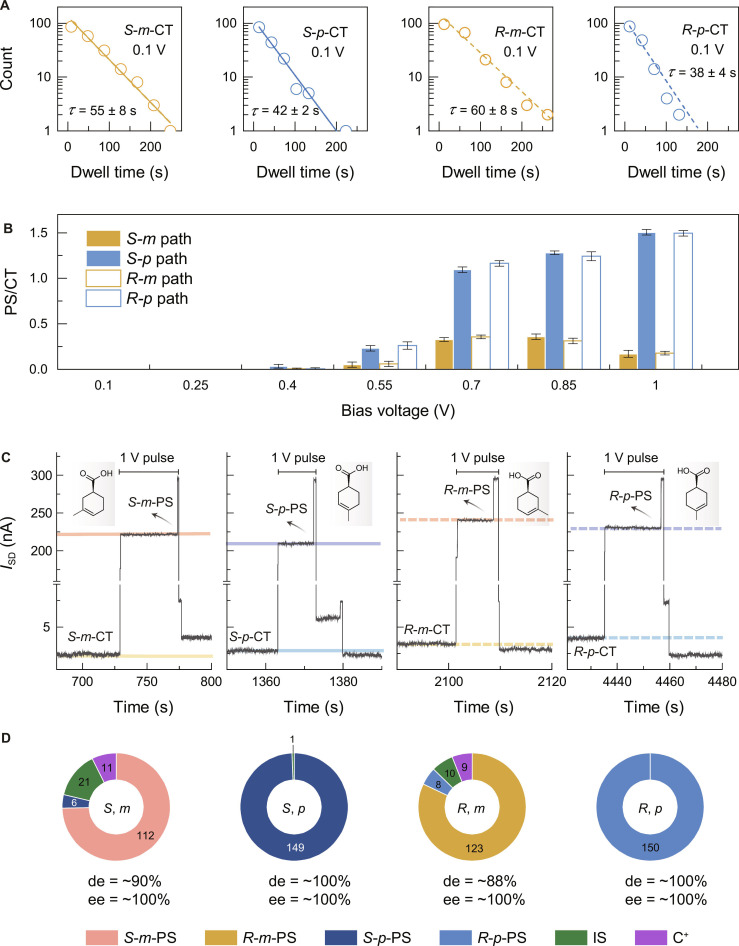
On-line asymmetric synthesis. (A) Statistical results of the dwell times of CTs. The lifetimes were obtained by linear fitting in semilogarithmic coordinates. (B) Equilibrium between CT and PS at different bias voltages. (C) *I*−*t* curves of the reaction, where a 1-V pulse was applied at a required CT state and removed at the C^+^ state to prepare the corresponding PSs. (D) Statistics of the application of 150 electrical pulses to *S*-*m*-CT, *R*-*m*-CT, *S*-*p*-CT, and *R*-*p*-CT to synthesize the targets *S*-*m*-PS, *R*-*m*-PS, *S*-*p*-PS, and *R*-*p*-PS, respectively. The statistics were derived from 20 separate devices, each operated approximately 30 times.

The dwell times of the 4 pre-reaction complexes were extracted, and the statistical results were linearly fitted in semilogarithmic coordinates (Fig. 4A). The obtained lifetimes on the second scale provide sufficient time for online regulation. In addition, appropriate separation of external energy input can guide the system to evolve in the desired direction while minimizing the disturbance from the thermal noise. Furthermore, bias voltage-dependent measurements demonstrate the effective triggering of the conversion from CTs to PSs at 1.0 V (Fig. 4B and Figs. S62 to S95). Consequently, asymmetric pathways can be selectively activated based on the detection of measurable initial symmetry breaking, including noncovalent complexes or intermediates. In terms of the energetic landscape, online regulation enables the single-molecule reaction to operate far from its inherent equilibrium through external energy (electrical pulse) input, ultimately facilitating the enrichment of the pure isomer (Fig. S60).

In detail, based on the detectable CTs with regioselectivity, the product with desired configuration was prepared by the application of 1 V at the corresponding CT state and removal at the C^+^ state (Fig. S61). To regulate the chirality of the products, although the absolute configuration (*re* or *si*) of the IS was not clear, their distinction and clarification of their conversion to the target chirality met our requirement for on-line control (Fig. 4C). Specifically, a previous *si*- (*re*-) IS allowed us to prepare an *R-* (*S-*) PS, whereas the current *p*- (*m*-) CT determined the regio-selectivity. Thus, the desired PS with stereo- and regio-selectivity [ee near 100% and diastereomeric excess (de) > 88%] (Fig. 4D) can be precisely prepared. More details about the operations to form desired species and the corresponding statistics are shown in the Supplementary Materials. Note that the conventional synthesis of chiral *m*-PS requires the hydrolysis of the corresponding chiral ester precursor (please see the Supplementary Materials). This on-line asymmetric synthesis also guides us to realize the precise regulation to the *endo* and *exo* selectivity of Diels–Alder reaction (Figs. S96 to S122) and regio-selective Suzuki–Miyaura cross-coupling with single-molecule resolution (details are provided in the Supplementary Materials and Figs. S123 to S129). The precise control of asymmetry and direct synthesis will provide a reliable access to complex molecules such as natural products.

## Conclusion

We directly observed SMSB in the Diels–Alder reaction and characterized the dynamics of chirality evolution at the single-molecule level. This enables a molecular-level view of how chirality evolves and propagates from the first few symmetry-breaking molecules to an increasing molecular population, complementing established ensemble paradigms such as Soai reaction and Viedma deracemization. We determine that the SMSB stems from the initial configuration of the acrylic acid substrates, rather than from the assumed pre-reaction complexes. The subsequent chiral amplification observed is particularly noteworthy. Through a combination of temperature-dependent measurements, autocorrelation analysis, and theoretical simulations, an excess-compensation mechanism was proposed. Symmetry breaking arises from the excessive compensation for an initial single-molecule chirality, driving the entire system away from equilibrium and toward an enantiomerically dominated state after several oscillation periods. Our elucidation of the mechanism behind spontaneous asymmetry provides important insights into the emergence of chirality in living systems and contributes to a molecular understanding of the biological evolution.

The sensitivity of the reaction system to the external environmental energy enabled us to regulate chirality. The full asymmetric reaction paths involving the *endo* and *exo*, *para* and *meta*, *R* and *S*, and *Z* and *E* configurations can be selectively turned on by electrical control. On-line manipulation according to the monitored reaction trajectories provides the ability to control the direction of the reaction, which meets society’s need for atomic economy and green chemistry. In combination with artificial intelligence, this proof-of-concept strategy will prompt the development of promising circuits or technologies with broad applications in precise chemical synthesis.

## Materials and Methods

### Device fabrication and molecular connection

High-quality single-layer graphene as the electrode was grown on a 25-μm-thick copper sheet by high-temperature chemical vapor deposition. With the support by spin-coated PMMA 950 and etching of the copper by the FeCl_3_ solution, graphene was then transferred to a 1.5 × 1.5 cm silicon wafer with a 300-nm SiO_2_ layer. After removing PMMA by acetone, graphene was spin-coated by photoresist to prepare a 40-μm-wide ribbon template by ultraviolet lithography. With the etching by oxygen plasma and removing the photoresist by acetone, a corresponding 40-μm-wide graphene sheet on a chip was obtained. The source and drain electrode array with 8-nm Cr, 60-nm Au, and 40-nm SiO_2_ was evaporated successively using the same template method. To inject/detect the spin carriers, the array of drain electrodes [0.6-nm Al (post-oxidation to Al_2_O_3_), 80-nm Ni, and 40-nm SiO_2_] and source electrodes (8-nm Cr, 60-nm Au, and 40-nm SiO_2_) were evaporated successively using the template method.

To open the windows on graphene and integrate the molecular bridges, a 150-nm-length and 5-nm-width dash line on the pre-spin-coated PMMA film was prepared by electron beam lithography. With the gradual etching by oxygen plasma and isotropous broadening of PMMA (graphene), a series of narrow gaps (1 to 10 nm) with carboxyl terminals between graphene point contacts were obtained between metal electrodes. The incomplete cutting of graphene can be characterized by *I*−*V* scans and further electrically burned (from 0 to 10 V) to ensure the detection of subsequent molecular connection.

To fix one molecule into graphene electrode pairs, freshly prepared point electrode arrays were added to a pyridine solution containing 0.1 mM of the molecular bridge (deprotection of Boc by CF_3_COOH) and 1 mM 1-(3-dimethylaminopropyl)-3-ethylcarbodiimide hydrochloride. After 48 h, the devices were removed from the solution and rinsed with deionized water and acetone, followed by drying with flowing N_2_. Finally, the recovery of the *I*−*V* response shows that the molecular bridge was integrated into graphene electrodes successfully by amide bonds.

### Electrical characterization

The single-molecule device was placed in a vacuum cryogenic probe station (Lake Shore TTPX for the on-line asymmetric synthesis). Especially for the chiral regulation, the device needs to be placed in physical property measurement system (PPMS) in advance to magnetize the Ni electrode by a direction- and strength-tunable magnetic field. The *I–V* curves were measured by an Agilent 4155C semiconductor parameter system. The output terminal of the UHFLI lock-in amplifier provided a constant bias or tunable pulse for the *I–t* measurement. The current signal of the molecular loop was amplified by a DHPCA amplifier and then recorded by a high-speed acquisition card (NIDAQ).

### Theoretical calculations

The geometries and energies of the intermediates and transition structures were optimized at the M06-2X/6-311+g(d,p) level, and empirical corrections for dispersion were included at the GD3 level. Trifluoroacetic acid solvation environment was considered by employing the SMD implicit solvation model with the following solvent descriptors referred from the CRC handbook: dielectric constant (ε) = 8.42; square of refraction ($n^{2}$) = 1.65; the fraction of nonhydrogen atoms that are electronegative halogen atoms (ψ) = 0.43; the other solvent descriptors were defined the same as these of acetic acid. Frequency calculations were performed to verify that the intermediates have no imaginary frequency, while the transition structures have only one imaginary frequency. The orientation of EEFs was set along C−C bond linking the 2 benzene rings of the fluorene group. The influence of the EEFs on the Gibbs free energy barriers was explored by gradually increasing the EEF strength from −2.57 V/nm to 2.57 V/nm [0.001 atomic unit (a.u.) corresponds to 514 V/m]. The reported Gibbs free energies were calculated at 298 K and 1 M. All calculations were performed with the Gaussian 16 software [41].

To analyze the transport properties of molecular junctions, we relaxed the structures of the simplified 2-probe molecular junctions of the substrate and the ortho-product at the B3LYP/6-31g(d) level, used the SMD implicit solvation model in the structural relaxation, and then carried out density functional theory (DFT) calculation within the nonequilibrium Green’s function (NEGF) formalism [42], as implemented in the Atomistix toolkit (ATK) package [43]. The 2 semi-infinite graphene electrodes were set as p-doped (0.003 holes per carbon), and the width of vacuum space (along *y* axis) was set as 20 Å before calculation. We adopted general gradient approximation (GGA), and we chose Fritz–Haber Institute (FHI) pseudopotential and double zeta polarized basis set. The cut-off energy for the real space grid was set at 100 Hartree. The NEGF-DFT self-consistent calculations were deemed converged when every element of the Hamiltonian matrix and the density matrix were converged to less than 10^−5^ a.u. Afterward, the transmission spectra were calculated, and the k-point meshes were set as 24 ×1.

## Data Availability

The data that support the findings of this study are available from the corresponding author upon request.

## References

[1] Liu YR, Wu ZL, Armstrong DW, Wolosker H, Zheng YB. Detection and analysis of chiral molecules as disease biomarkers. Nat Rev Chem. 2023;7(5):355–373. 37117811 10.1038/s41570-023-00476-z PMC10175202

[2] Han B, He X-H, Liu Y-Q, He G, Peng C, Li J-L. Asymmetric organocatalysis: An enabling technology for medicinal chemistry. Chem Soc Rev. 2021;50:1522–1586. 33496291 10.1039/d0cs00196a

[3] Yang SH, Naaman R, Paltiel Y, Parkin SSP. Chiral spintronics. Nat Rev Phys. 2021;3(5):328–343.

[4] Kim YH, Zhai Y, Lu H, Pan X, Xiao C, Gaulding EA, Harvey SP, Berry JJ, Vardeny ZV, Luther JM, et al. Chiral-induced spin selectivity enables a room-temperature spin light-emitting diode. Science. 2021;371(6534):1129–1133. 33707260 10.1126/science.abf5291

[5] Deng M, Yu JH, Blackmond DG. Symmetry breaking and chiral amplification in prebiotic ligation reactions. Nature. 2024;626:1019–1024. 38418914 10.1038/s41586-024-07059-y

[6] Bailey J, Chrysostomou A, Hough JH, Gledhill TM, McCall A, Clark S, Menard F, Tamura M. Circular polarization in star-formation regions: Implications for biomolecular homochirality. Science. 1998;281(5377):672–674. 9685254

[7] Piñeros WD, Tlusty T. Spontaneous chiral symmetry breaking in a random driven chemical system. Nat Commun. 2022;13:2244. 35474070 10.1038/s41467-022-29952-8 PMC9042824

[8] Buhse T, Cruz T-M, Noble-Teran ME, Hochberg D, Ribo JM, Crusats MJ-C. Spontaneous deracemizations. Chem Rev. 2021;121(4):2147–2229. 33464058 10.1021/acs.chemrev.0c00819

[9] Bocková J, Jones NC, Hoffmann SV, Meinert C. The astrochemical evolutionary traits of phospholipid membrane homochirality. Nat Rev Chem. 2024;8:652–664. 39025922 10.1038/s41570-024-00627-w

[10] Ozturk SF, Liu ZW, Sutherland JD, Sasselov DD. Origin of biological homochirality by crystallization of an RNA precursor on a magnetic surface. Sci Adv. 2023;9(23):eadg8274. 37285423 10.1126/sciadv.adg8274 PMC10246896

[11] Athavale SV, Simon A, Houk KN, Denmark SE. Demystifying the asymmetry-amplifying, autocatalytic behaviour of the Soai reaction through structural, mechanistic and computational studies. Nat Chem. 2020;12(4):412–423. 32203445 10.1038/s41557-020-0421-8 PMC7117993

[12] Soai K, Shibata T, Morioka H, Choji K. Asymmetric autocatalysis and amplification of enantiomeric excess of a chiral molecule. Nature. 1995;378:767–768.

[13] Viedma C, Ortiz JE, de Torres T, Izumi T, Blackmond DG. Evolution of solid phase homochirality for a proteinogenic amino acid. J Am Chem Soc. 2008;130:15274–15275. 18954052 10.1021/ja8074506

[14] Viedma C. Chiral symmetry breaking during crystallization: Complete chiral purity induced by nonlinear autocatalysis and recycling. Phys Rev Lett. 2005;94: Article 065504. 15783745 10.1103/PhysRevLett.94.065504

[15] Huang B, Bates M, Zhuang XW. Super-resolution fluorescence microscopy. Annu Rev Biochem. 2009;78:993–1016. 19489737 10.1146/annurev.biochem.77.061906.092014 PMC2835776

[16] Dong JR, Lu Y, Xu Y, Chen F, Yang J, Chen Y, Feng J. Direct imaging of single-molecule electrochemical reactions in solution. Nature. 2021;596:244–249. 34381236 10.1038/s41586-021-03715-9

[17] Xu BQ, Tao NJJ. Measurement of single-molecule resistance by repeated formation of molecular junctions. Science. 2003;301(5637):1221–1223. 12947193 10.1126/science.1087481

[18] Florin EL, Moy VT, Gaub HE. Adhesion forces between individual ligand-receptor pairs. Science. 1994;264(5157):415–417. 8153628 10.1126/science.8153628

[19] Liu CM, Kubo K, Wang E, Han KS, Yang F, Chen G, Escobedo FA, Coates GW, Chen P. Single polymer growth dynamics. Science. 2017;358(6361):352–355. 29051377 10.1126/science.aan6837

[20] Armani AM, Kulkarni RP, Fraser SE, Flagan RC, Vahala KJ. Label-free, single-molecule detection with optical microcavities. Science. 2007;317(5839):783–787. 17615303 10.1126/science.1145002

[21] Deamer D, Akeson M, Branton D. Three decades of nanopore sequencing. Nat Biotechnol. 2016;34(5):518–524. 27153285 10.1038/nbt.3423 PMC6733523

[22] Li Y, Yang C, Guo XF. Single-molecule electrical detection: A promising route toward the fundamental limits of chemistry and life science. Acc Chem Res. 2020;53(1):159–169. 31545589 10.1021/acs.accounts.9b00347

[23] Dief EM, Low PJ, Díez-Pérez I, Darwish N. Advances in single-molecule junctions as tools for chemical and biochemical analysis. Nat Chem. 2023;15(5):600–614. 37106094 10.1038/s41557-023-01178-1

[24] Gao CY, Gao Q, Zhao C, Huo Y, Zhang Z, Yang J, Jia C, Guo X. Technologies for investigating single-molecule chemical reactions. Natl Sci Rev. 2024;11(8): Article nwae236. 39224448 10.1093/nsr/nwae236 PMC11367963

[25] Bi XY, Czajkowsky DM, Shao ZF, Ye J. Digital colloid-enhanced Raman spectroscopy by single-molecule counting. Nature. 2024;628(8009):771–775. 38632399 10.1038/s41586-024-07218-1

[26] Jia CC, Migliore A, Xin N, Huang S, Wang J, Yang Q, Wang S, Chen H, Wang D, Feng B, et al. Covalently bonded single-molecule junctions with stable and reversible photoswitched conductivity. Science. 2016;352(6292):1443–1445. 27313042 10.1126/science.aaf6298

[27] Tan M, Sun F, Zhao X, Zhao Z, Zhang S, Xu X, Adijiang A, Zhang W, Wang H, Wang C, et al. Evolution of photoisomeric single-molecule junctions under ultraviolet irradiation and mechanical stretching. J Am Chem Soc. 2024;146(10):6856–6865. 38413090 10.1021/jacs.3c13752

[28] Liang WJ, Shores MP, Bockrath M, Long JR, Park H. Kondo resonance in a single-molecule transistor. Nature. 2002;417:725–729. 12066180 10.1038/nature00790

[29] Liu JY, Huang XY, Wang F, Hong WJ. Quantum interference effects in charge transport through single-molecule junctions: Detection, manipulation, and application. Acc Chem Res. 2019;52(1):151–160. 30500161 10.1021/acs.accounts.8b00429

[30] Yang C, Zhang L, Lu C, Zhou S, Li X, Li Y, Yang Y, Li Y, Liu Z, Yang J, et al. Unveiling the full reaction path of the Suzuki–Miyaura cross-coupling in a single-molecule junction. Nat Nanotechnol. 2021;16:1214–1223. 34475558 10.1038/s41565-021-00959-4

[31] Aragonès AC, Haworth NL, Darwish N, Ciampi S, Mannix EJ, Wallace GG, Diez-Perez I, Coote ML. Electrostatic catalysis of a Diels-Alder reaction. Nature. 2016;531(7592):88–91. 26935697 10.1038/nature16989

[32] Chen HL, Jia C, Zhu X, Yang C, Guo X, Stoddart JF. Reactions in single-molecule junctions. Nat Rev Mater. 2023;8:165–185.

[33] Hu WL, Li M, Xiong W, Zhou S, Zou Q, Lü JT, Tian H, Guo X. Real-time direct monitoring of chirality fixation and recognition at the single-molecule level. J Am Chem Soc. 2024;146(26):17765–17772. 38902874 10.1021/jacs.4c03071

[34] Yang C, Yang CY, Guo YL, Feng JF, Guo XF. Graphene–molecule–graphene single-molecule junctions to detect electronic reactions at the molecular scale. Nat Protoc. 2023;18:1958–1978. 37045993 10.1038/s41596-023-00822-x

[35] Gu CH, Hu C, Wei Y, Lin D, Jia C, Li M, Su D, Guan J, Xia A, Xie L, et al. Label-free dynamic detection of single-molecule nucleophilic-substitution reactions. Nano Lett. 2018;18(7):4156–4162. 29874453 10.1021/acs.nanolett.8b00949

[36] Bloom BP, Paltiel Y, Naaman R, Waldeck DH. Chiral induced spin selectivity. Chem Rev. 2024;124(4):1950–1991. 38364021 10.1021/acs.chemrev.3c00661 PMC10906005

[37] Li HPB, Tebikachew BE, Wiberg C, Moth-Poulsen K, Hihath J. A memristive element based on an electrically controlled single-molecule reaction. Angew Chem Int Ed Engl. 2020;59(28):11641–11646. 32222017 10.1002/anie.202002300

[38] Huang ZF, Chen F, D’agosta R, Bennett PA, di Ventra M, Tao N. Local ionic and electron heating in single-molecule junctions. Nat Nanotechnol. 2007;2:698–703. 18654408 10.1038/nnano.2007.345

[39] Yang C, Liu Z, Zhou S, Lu C, Guo Y, Ramirez M, Zhang Q, Li Y, Liu Z, Houk KN, et al. Electric field–catalyzed single-molecule Diels-Alder reaction dynamics. Sci Adv. 2021;7(4):eabf0689. 33523936 10.1126/sciadv.abf0689 PMC7817103

[40] McGuire BA, Carroll PB, Loomis RA, Finneran IA, Jewell PR, Remijan AJ, Blake GA. Discovery of the interstellar chiral molecule propylene oxide (CH_3_CHCH_2_O). Science. 2016;352(6292):1449–1452. 27303055 10.1126/science.aae0328

[41] Frisch MJ, Trucks GW, Schlegel HB, Scuseria GE, Robb MA, Cheeseman JR, Scalmani G, Barone V, Petersson GA, Nakatsuji H, et al. *Gaussian 16 Rev. C.01.*

[42] Brandbyge M, Mozos JL, Ordejón P, Taylor J, Stokbro K. Density-functional method for nonequilibrium electron transport. Phys Rev B. 2002;65(16):165401.

[43] Smidstrup S, Markussen T, Vancraeyveld P, Wellendorff J, Schneider J, Gunst T, Verstichel B, Stradi D, Khomyakov PA, Vej-Hansen UG, et al. QuantumATK: An integrated platform of electronic and atomic-scale modelling tools. J Phys Condens Mat. 2020;32(1): Article 015901. 10.1088/1361-648X/ab4007 31470430

