# Supplementary material for: Origin of Single-Molecule Reaction Chirality
Source: Research (Wash D C). 2026 Feb 24;9:1150. doi: 10.34133/research.1150 (PMC12929813; doi:10.34133/research.1150)
Supplement: Supplementary 1 — Supplementary Text Figs. S1 to S129 [file research.1150.f1.pdf]

Supplementary Materials for

Origin of single-molecule reaction chirality

This PDF file includes:

Supplementary Text
Figs. S1 to S129
References

1. **Schematic of the fabrication procedure of a single-molecule device.**

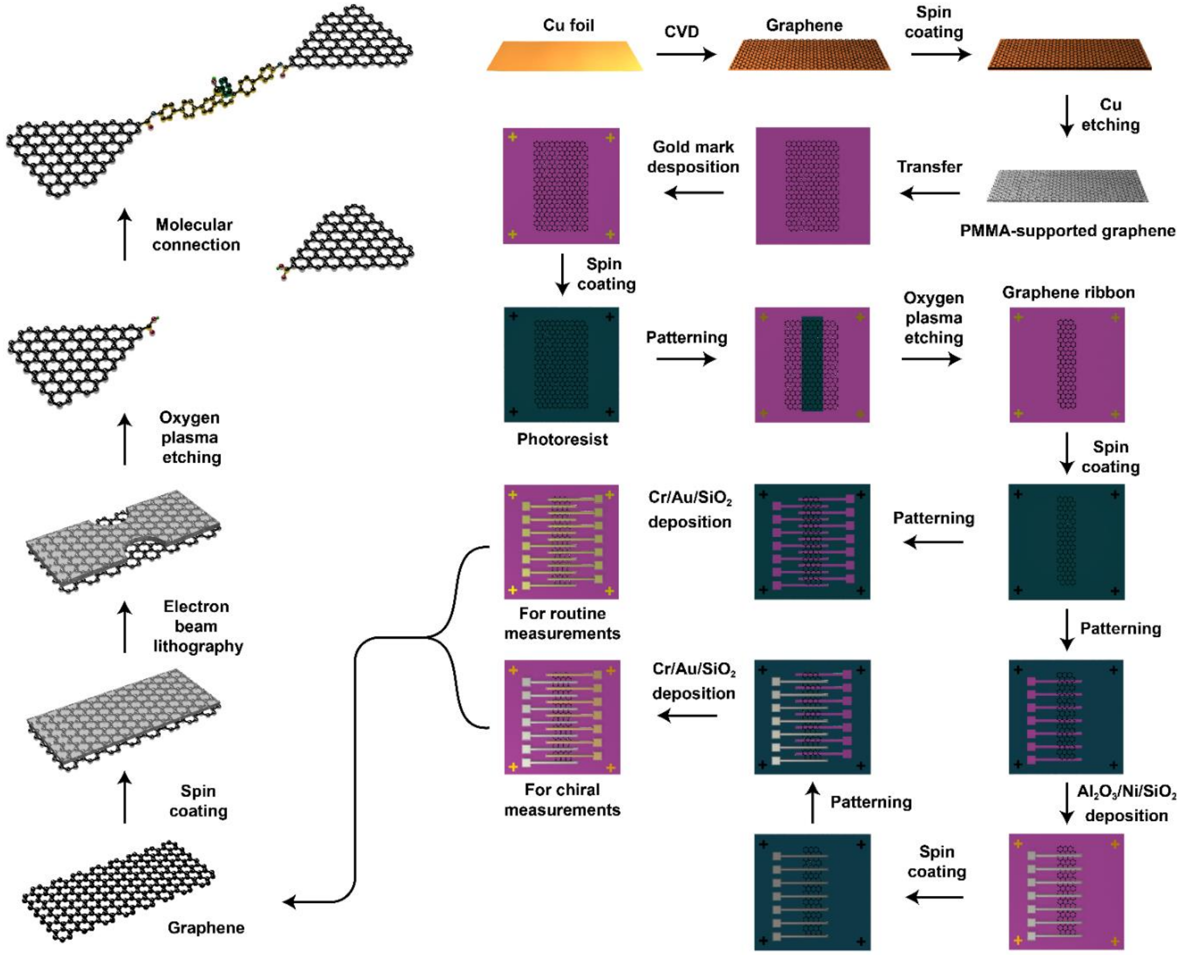

Fig. S1. The fabrication procedure of graphene field-effect transistors and the preparation of single-molecule devices.

2. Electrical characterization of the single-molecule device

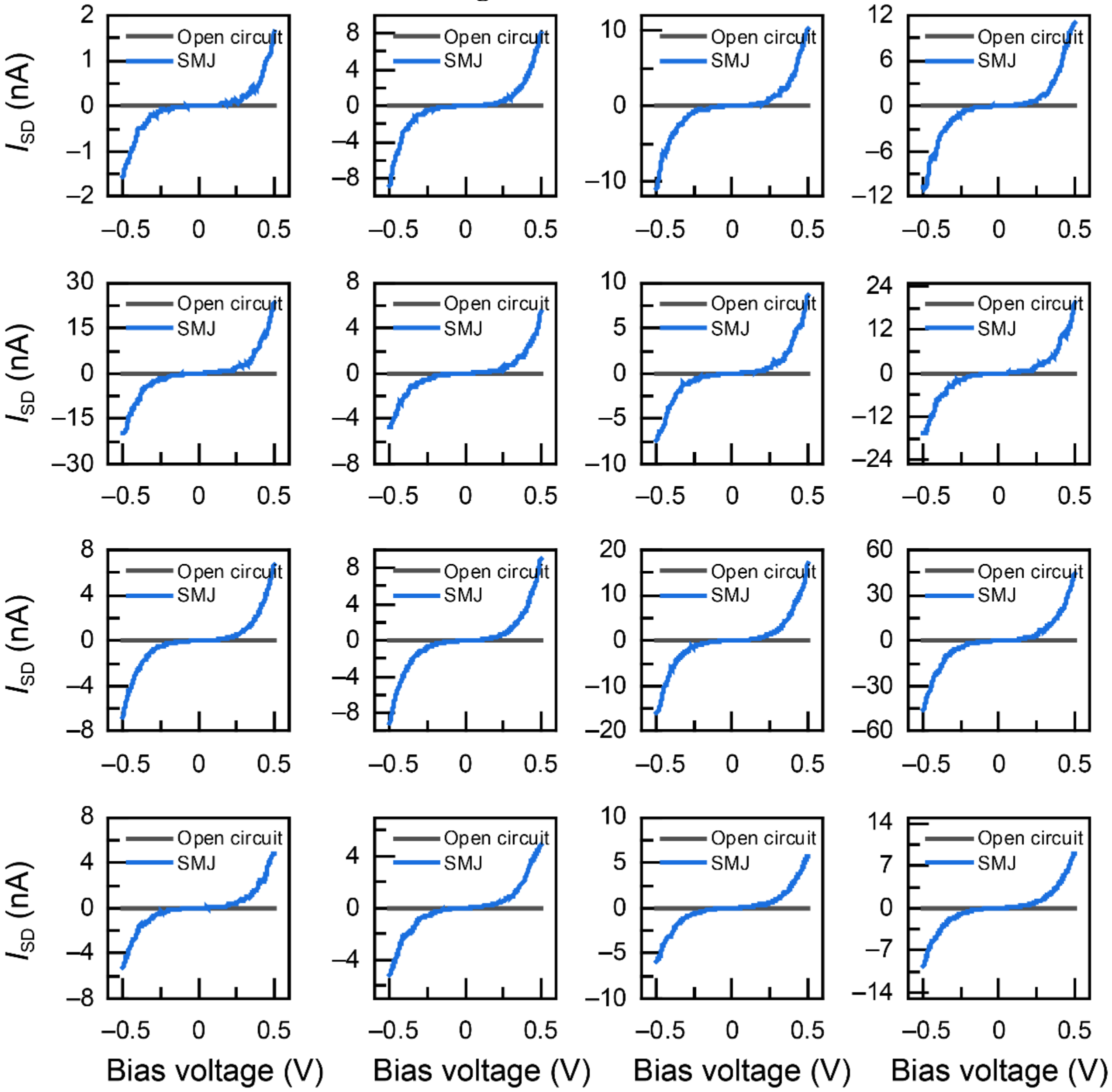

Fig. S2. 16 *I*−*V* curves of 9-phenyl-9-fluorenol single-molecule junctions. The successful preparation of single-molecule junctions was determined by comparing the current-voltage (*I*−*V*) curve after oxygen plasma etching (open circuit) with the curve after connecting the molecules between metal electrodes (recover to some extent).

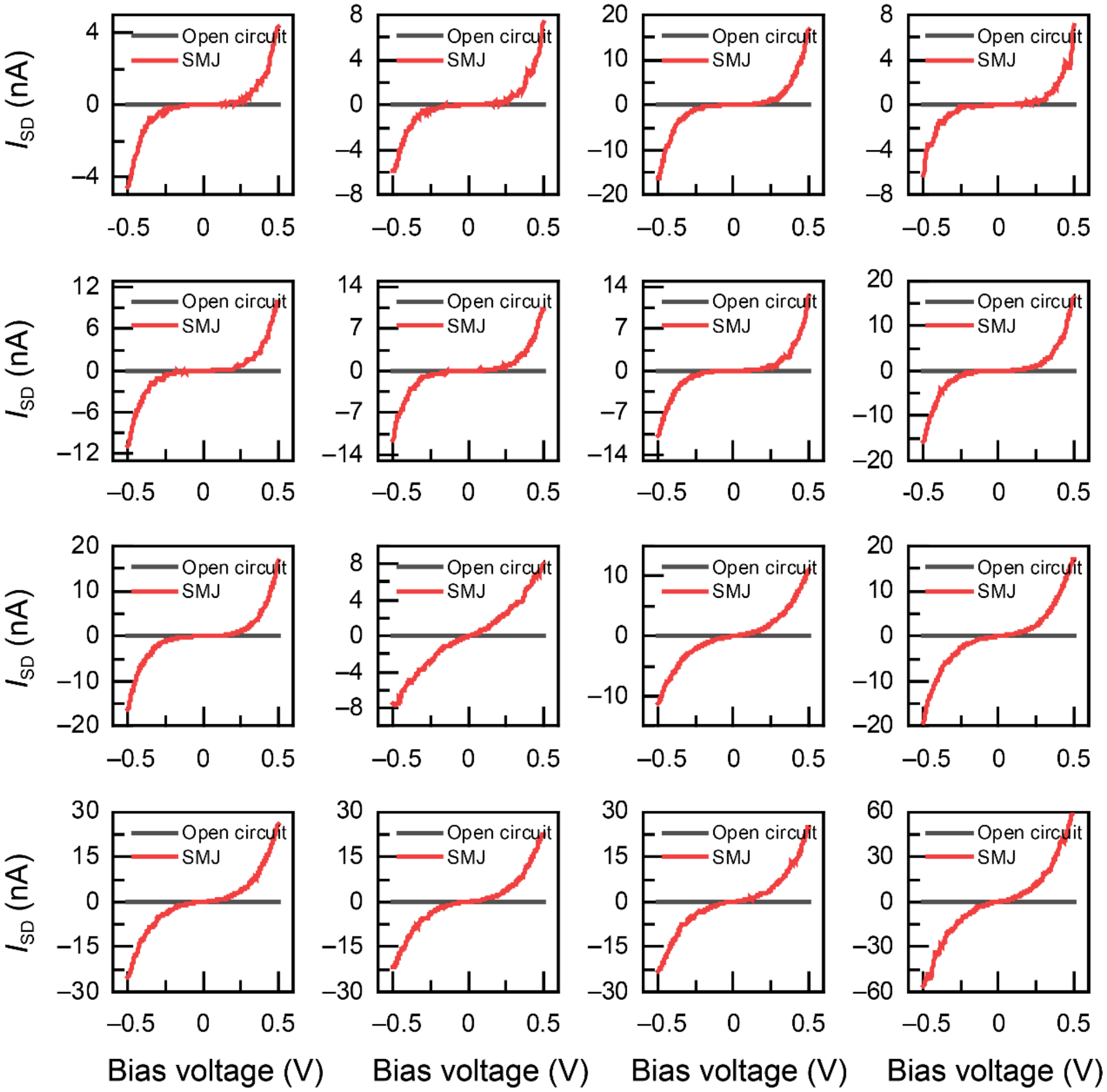

Fig. S3. 16 *I*−*V* curves of maleimide single-molecule junctions. The successful preparation of single-molecule junctions was determined by comparing the current−voltage (*I*−*V*) curve after oxygen plasma etching (open circuit) with the curve after connecting the molecules between metal electrodes (recover to some extent).

3. Single-molecule connection analysis

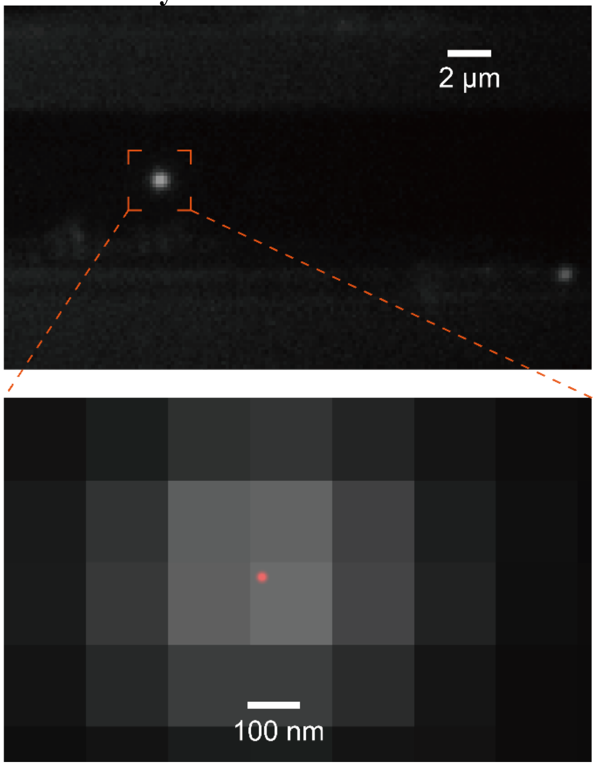

Fig. S4. Superhigh resolution imaging and single-molecule localization. The interaction between Eosin Y and 9-phenyl-9-fluorenyl cation was recorded by using a ×100 oil lens for an exposure time of 50 ms (EM gain = 500), resulting in the acquisition of 5,000 images. The localization of a single molecule was achieved by stochastic optical reconstruction (bottom panel). Only one 9-phenyl-9-fluorenyl cation was detected and marked (as red spot) between metal electrodes.

4. The assignments of the conductance states in *para* & *meta* paths

The monitoring of the species during the reaction was based on the association-dissociation of the C^+ molecular bridge. Therefore, the assignments of the conductance states can be realized by addition of the control standard compounds (Fig. S6) and the corresponding concentration-dependent measurements. The addition of acrylic acid and the concentration-dependent measurements illustrate the assignments of the C^+ and IS state (Fig. S7, more IS states at higher concentrations). The chemical bonds could also be characterized by inelastic electron tunneling spectra (IETS, Figs. S10 and 11, similar to infrared and Raman spectra), which further support the above assignments.

The addition of both isoprene and acrylic acid at low temperatures provided the assignments of *m*-CT and *p*-CT (newly appeared), while the further concentration-dependent (Figs. S7 and 8) and bias voltage-dependent measurements (Section 9) provided the assignments of *m*-PS and *p*-PS. The IETS (Figs. S12 and 13) and the addition of the corresponding products (Fig. S6) further support the assignments of *m*-PS and *p*-PS. The statistical conversion time sequence supports the monitored meta and para pathways (Fig. S14, strong correlation between corresponding CT and PS), and the statistics of the occupancy of the above six species are also in line with the theoretical thermodynamic preference. In addition, the assignments also agree with the simulation of the electron transmission spectra and calculated $I-V$ curves (Figs. S15 and 16).

We have also characterized the reaction at the routine condition (343 K) and tracked it with macroscopic GC-MS (Section 5). Similar trends of the yield over time indicate the reliability of single-molecule monitoring.

We also characterized the products (cycloaddition between propionic acid and isoprene) with single-molecule resolved fluorescent spectra to show the smooth proceeding of single-molecule catalysis (Section 7).

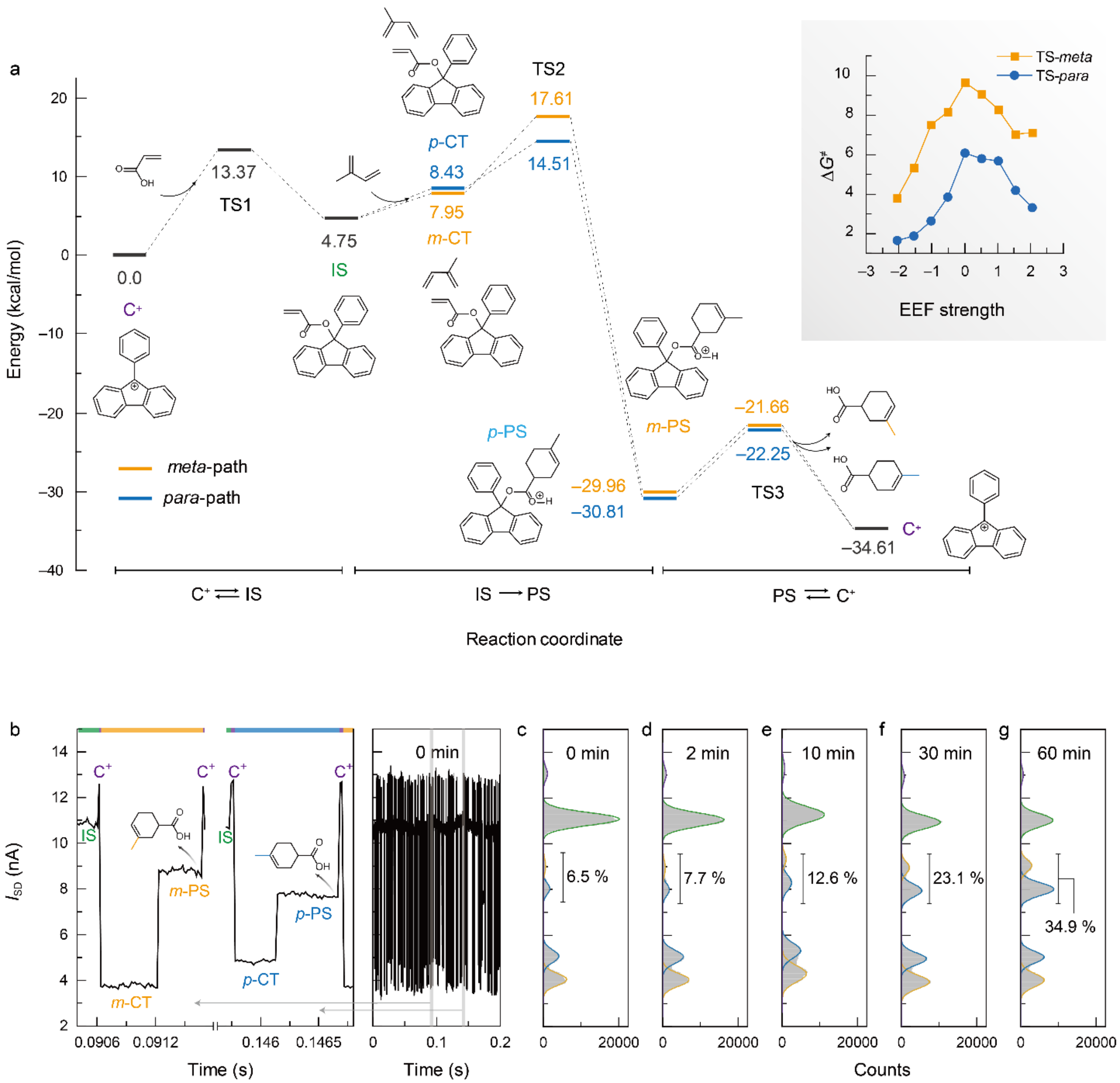

Fig. S5. Theoretical and experimental studies of the cascade reaction. A. Energy profile of the cascade reaction at 0 V/nm. The insert shows the energy barrier of cycloaddition versus the EEF strength. **B.** Routine monitoring of the cascade reaction at 343 K and 0.1 V. The left-hand panel shows the enlarged *I*−*t* curves and the assignments of the current levels. **C−G.** Corresponding frequency distributions of the *I*−*t* curves at 0, 2, 10, 30, and 60 min, respectively. *m*: *meta*-configuration. *p*: *para*-configuration. IS: associated intermediate state. CT: pre-reaction charge transfer complex salt state. PS: product state. TS: transition state.

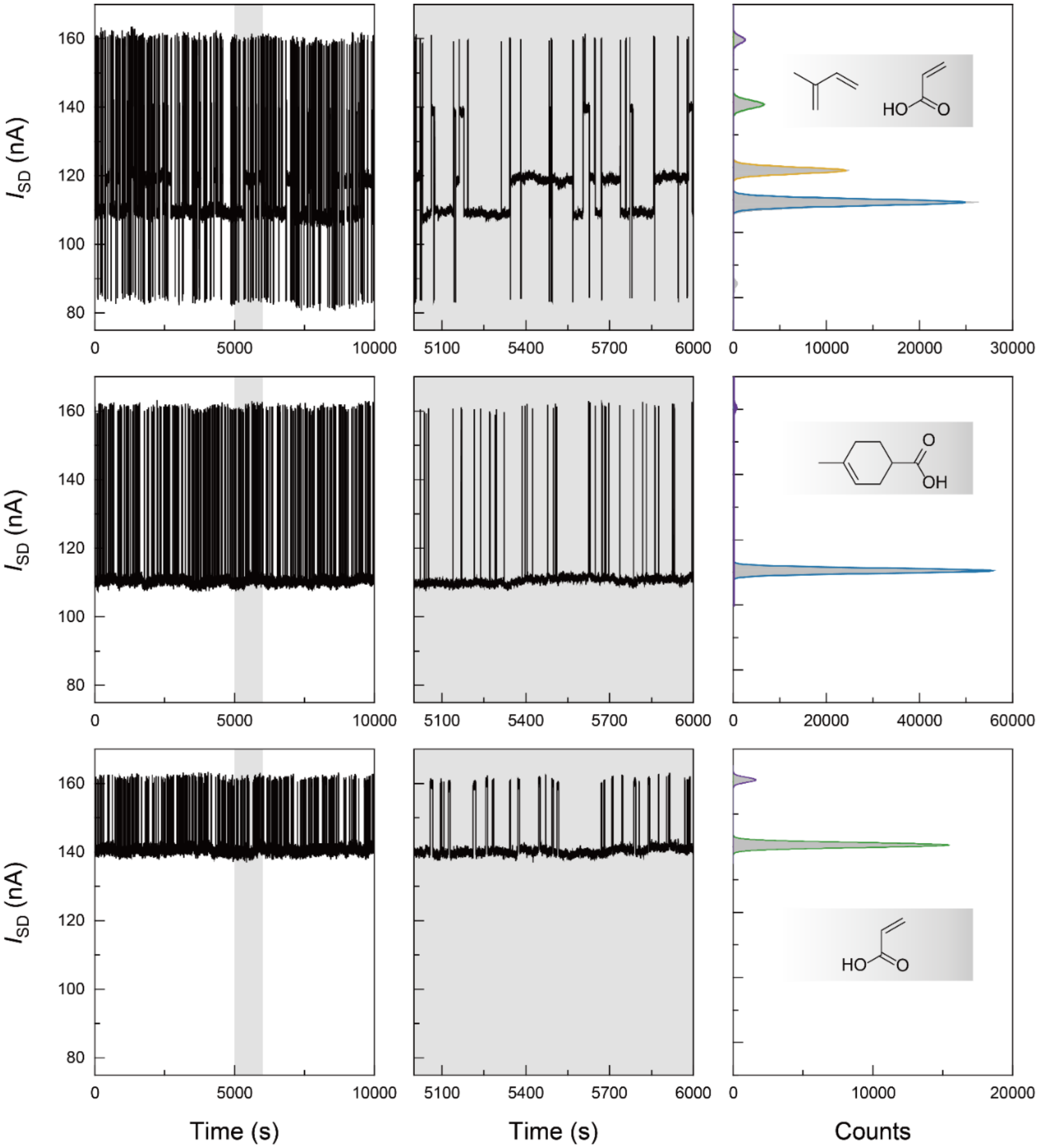

Fig. S6. Control experiments of the reaction between acrylic acid and isoprene. By comparing the *I*–*t* curves at 1 V and 100 K with the addition of only racemic 4-methyl-3-cyclohexene-1-carboxylic acid (1 mM, middle panel) or acrylic acid (1 mM, bottom panel), the *p*-PS and IS can be assigned in the reaction signals (top panel).

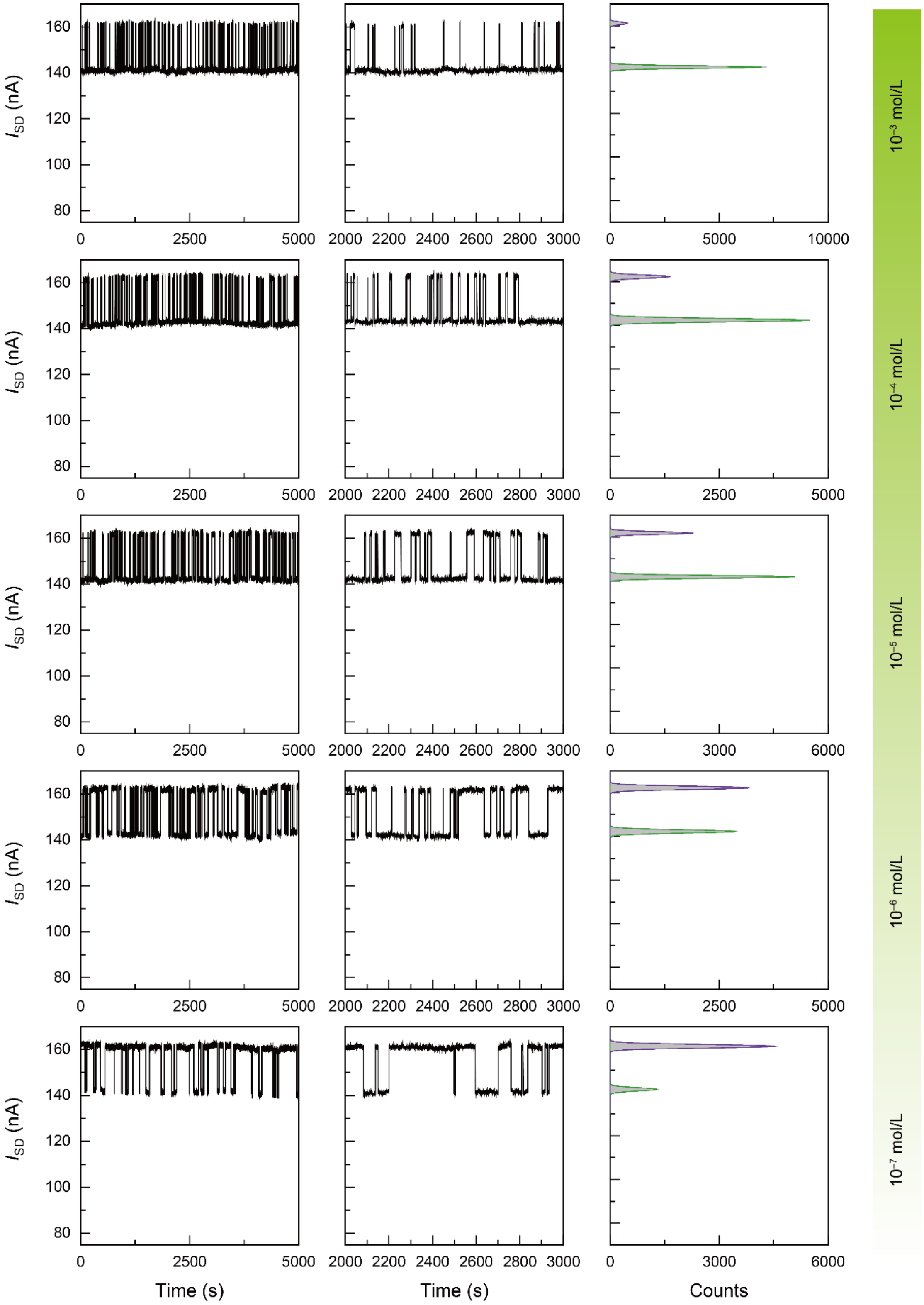

Fig. S7. Acrylic acid concentration-dependent measurements without addition of isoprene. *I*–*t* curves, corresponding enlarged images and statistical histograms measured in TFA solutions with 10^{-7} mol/L, 10^{-6} mol/L, 10^{-5} mol/L, 10^{-4} mol/L and 10^{-3} mol/L acrylic acid at 1 V bias.

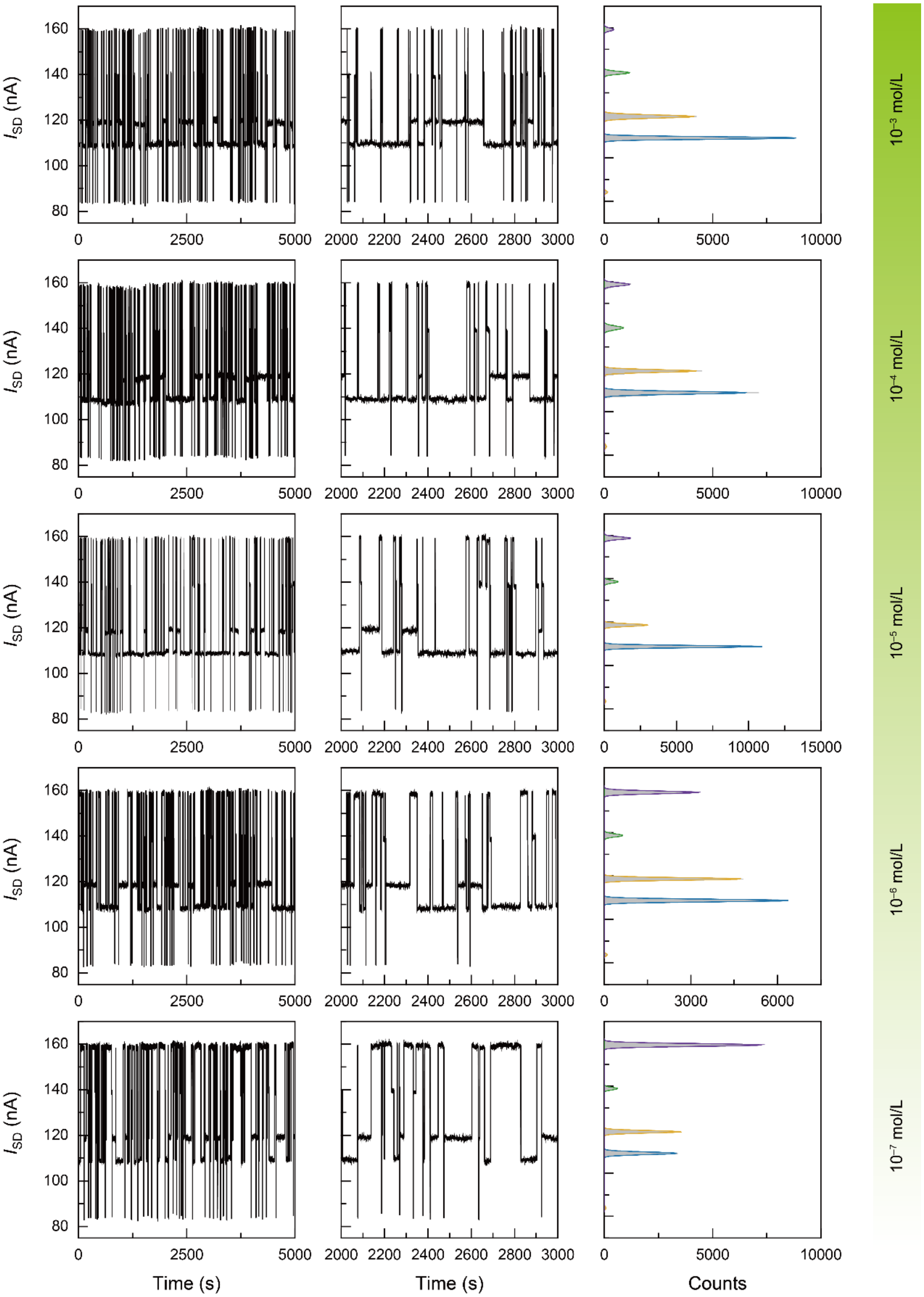

Fig. S8. Acrylic acid concentration-dependent measurements with addition of isoprene (10^{-3} mol/L). *I*–*t* curves, corresponding enlarged images and statistical histograms measured in TFA solutions with 10^{-7} mol/L, 10^{-6} mol/L, 10^{-5} mol/L, 10^{-4} mol/L and 10^{-3} mol/L acrylic acid at 1 V bias.

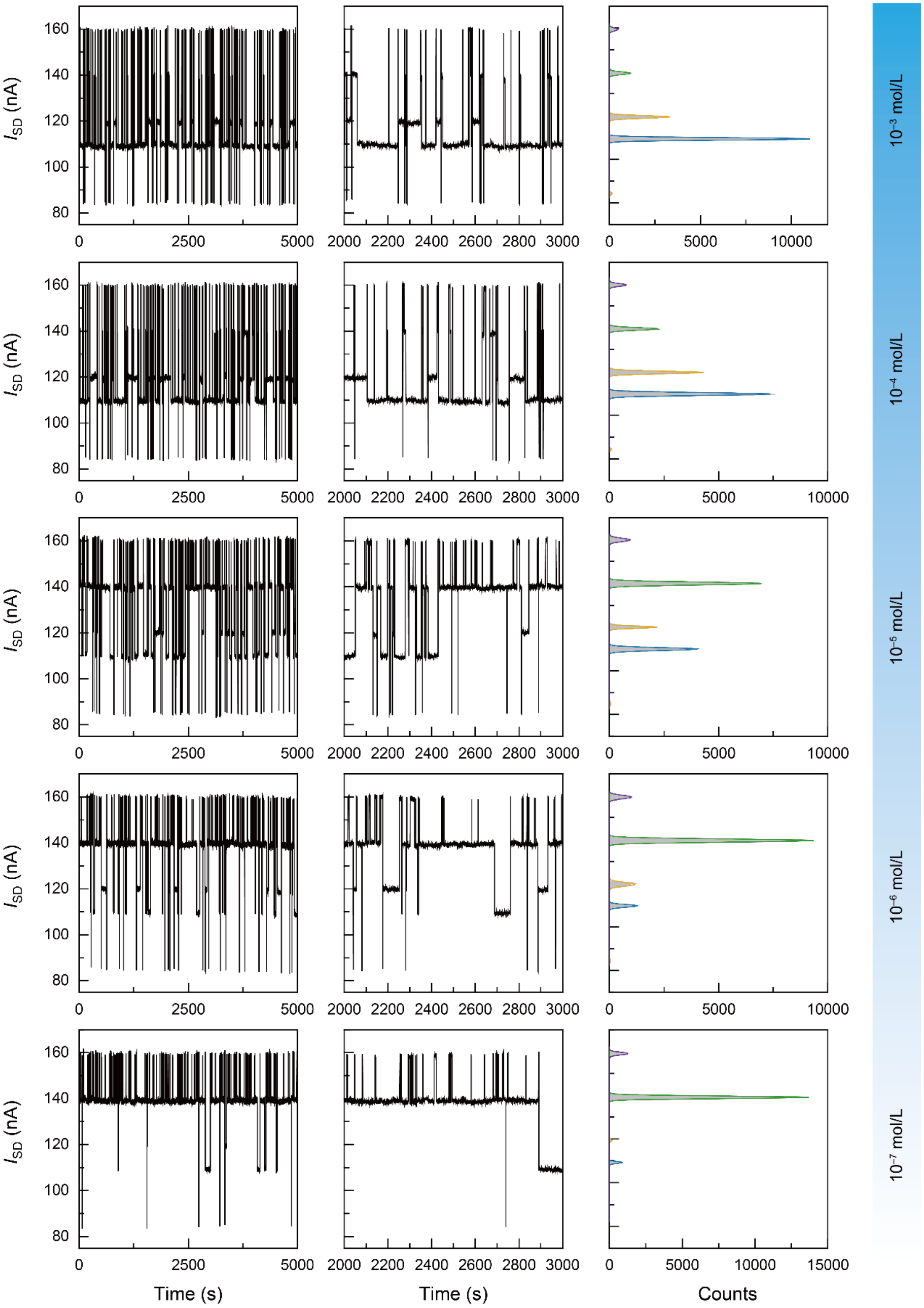

Fig. S9. Isoprene concentration-dependent measurements with addition of acrylic acid (10^{-3} mol/L). I–t curves, corresponding enlarged images and statistical histograms measured in TFA solutions with 10^{-7} mol/L, 10^{-6} mol/L, 10^{-5} mol/L, 10^{-4} mol/L and 10^{-3} mol/L isoprene at 1 V bias.

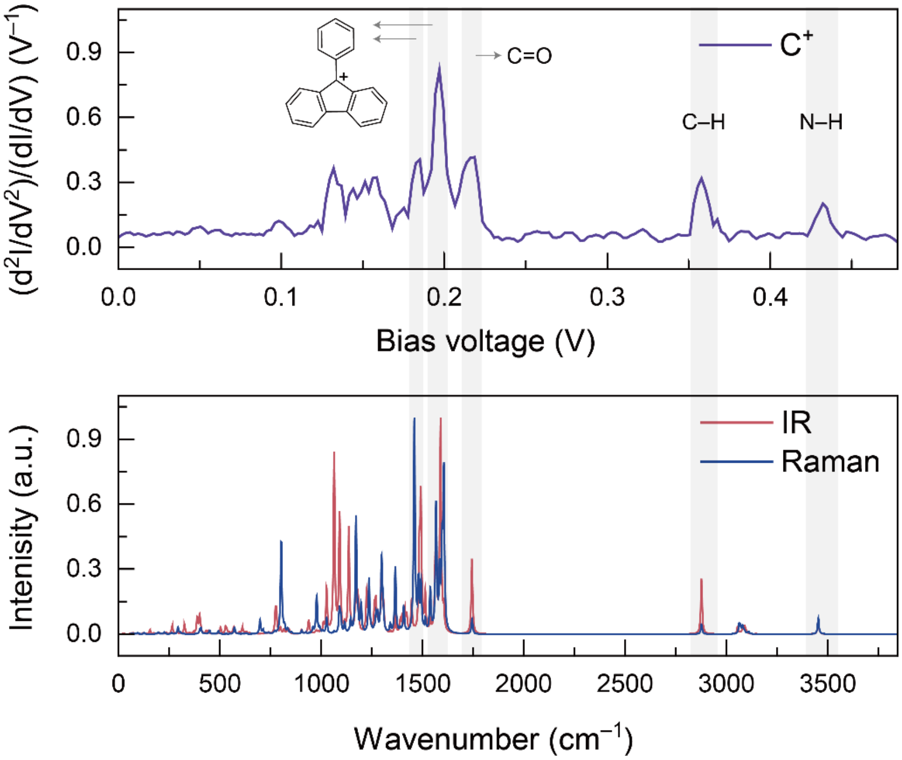

Fig. S10. IETS of the C^+ intermediate. Top: The C^+ intermediate was captured and stabilized at 2 K. The IETS was measured by a lock-in second-harmonic technique with an AC modulation of 7 mV at a frequency of 661 Hz. Bottom: calculated infrared and Raman spectra of the molecular bridge at the C^+ state. The peaks assigned to specific vibrational modes are marked out in the IETS ($\nu = h\omega/e$). We observed the peaks of δ (9-phenyl-9-fluorenyl cation) (~183 mV and ~197 mV) and ν(C−H) (~358 mV). In addition, the specific peaks of the amide bond anchor can be detected as ν(C=O) (~215 mV) and ν(N−H) (~432 mV). All these peaks in the IETS indicate intrinsic vibrations of the C^+ intermediate.

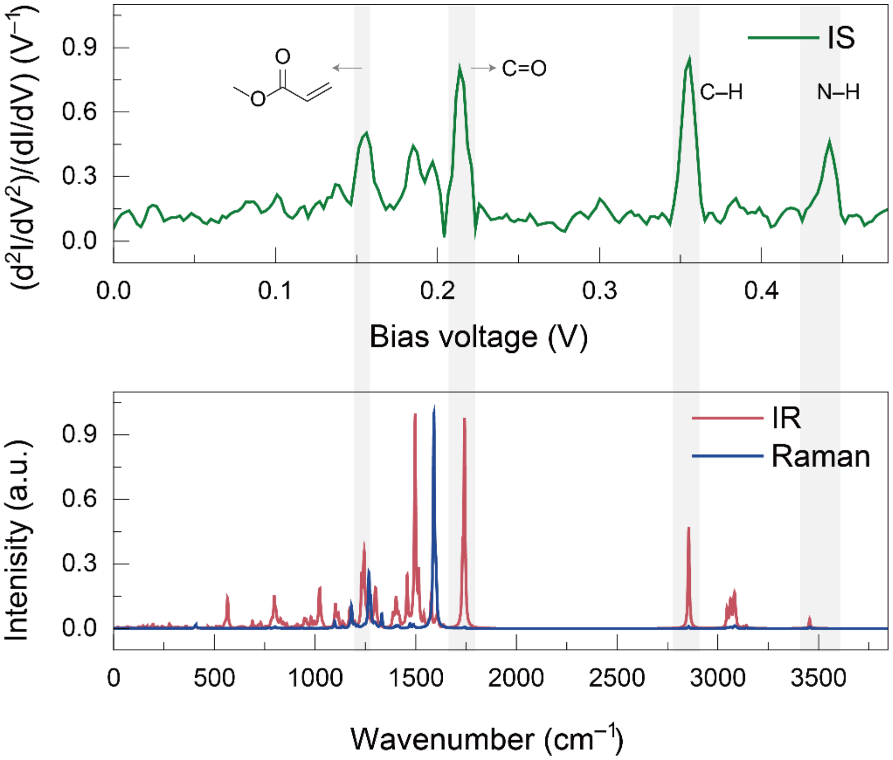

Fig. S11. IETS of the IS intermediate. Top: The IS intermediate was captured and stabilized at 2 K. The IETS was measured by a lock-in second-harmonic technique with an AC modulation of 7 mV at a frequency of 661 Hz. Bottom: calculated infrared and Raman spectra of the molecular bridge at IS state. The peaks assigned to specific vibrational modes are marked out in the IETS ($v = h\omega/e$). We observed the peaks of δ (acrylic acid moiety) (~154 mV) and ν(C−H) (~355 mV). In addition, the specific peaks of the amide bond anchor can be detected as ν(C=O) (~215 mV) and ν(N−H) (~440 mV). All these peaks in the IETS indicate intrinsic vibrations of the IS intermediate.

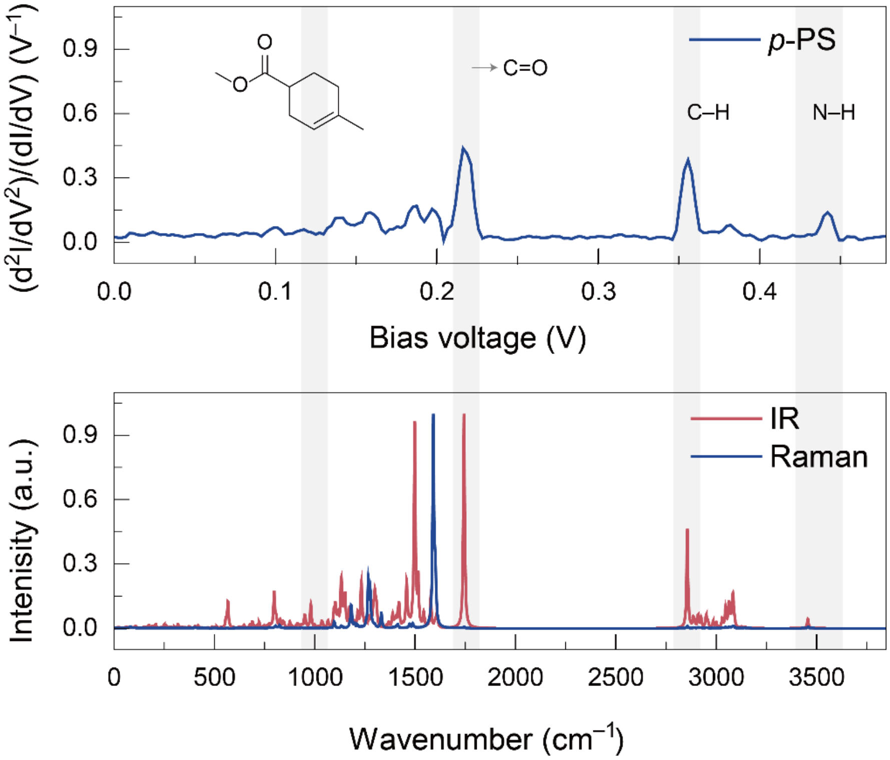

Fig. S12. IETS of the *p*-PS intermediate. Top: The *p*-PS intermediate was captured and stabilized at 2 K. The IETS was measured by a lock-in second-harmonic technique with an AC modulation of 7 mV at a frequency of 661 Hz. Bottom: calculated infrared and Raman spectra of the molecular bridge at IS state. The peaks assigned to specific vibrational modes are marked out in the IETS ($v = h\omega/e$). We observed the specific peaks of the amide bond anchor as ν(C=O) (~215 mV) and ν(N−H) (~442 mV), and ν(C−H) (~354 mV).

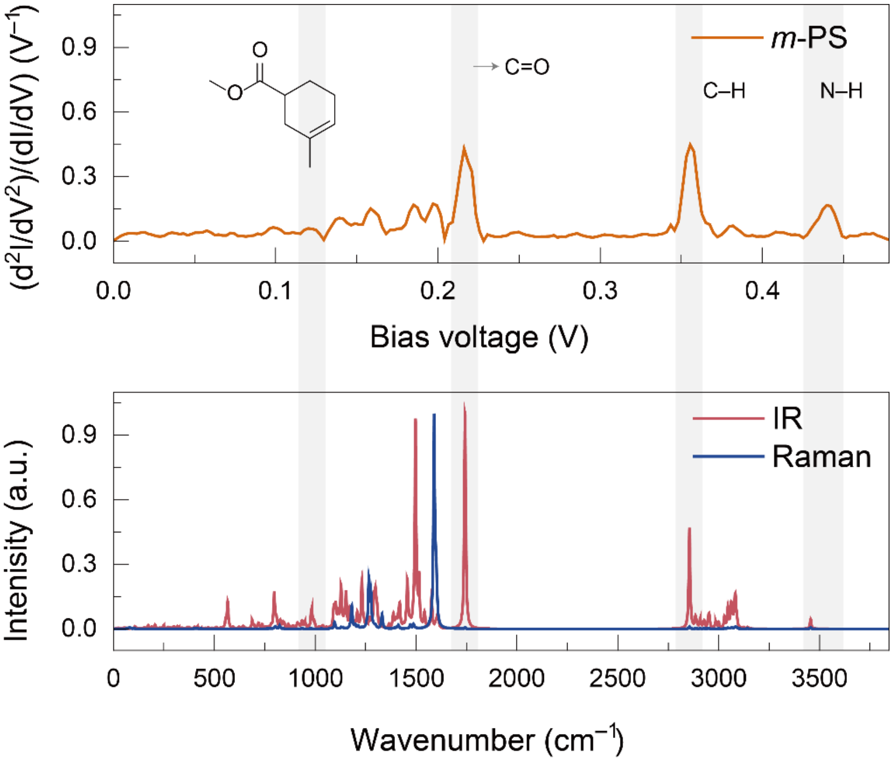

Fig. S13. IETS of the *m*-PS intermediate. Top: The *m*-PS intermediate was captured and stabilized at 2 K. The IETS was measured by a lock-in second-harmonic technique with an AC modulation of 7 mV at a frequency of 661 Hz. Bottom: calculated infrared and Raman spectra of the molecular bridge at IS state. The peaks assigned to specific vibrational modes are marked out in the IETS ($v = h\omega/e$). We observed the specific peaks of the amide bond anchor as ν(C=O) (~215 mV) and ν(N−H) (~442 mV), and ν(C−H) (~354 mV).

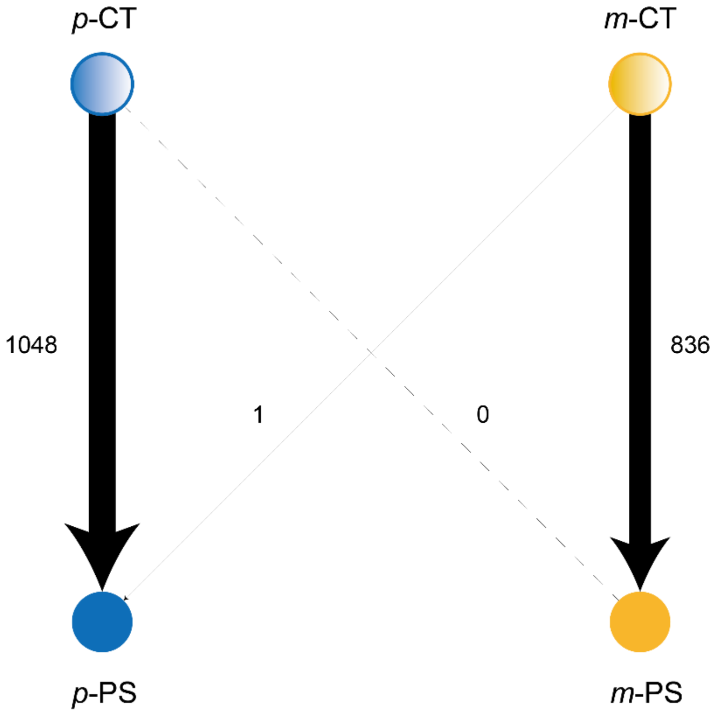

Fig. S14. The statistical conversion relationship between CTs and PSs. The thickness of the arrow intuitively indicates the statistics of the conversion numbers, which showing the existence of two paths and support to the corresponding assignments.

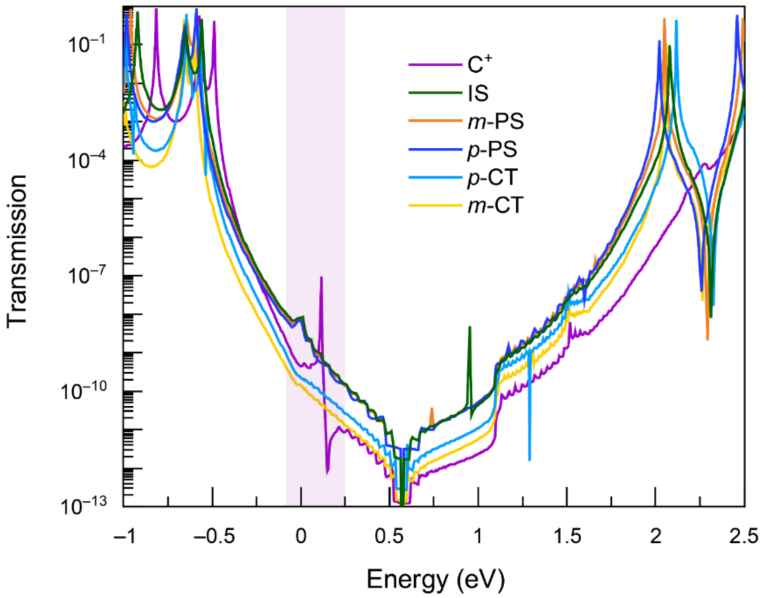

Fig. S15. Transmission spectra of the six species during the reaction between acrylic acid and isoprene. Because the LUMO (electron transmission channel) is closest to the electrode Fermi level (0 eV), the 9-phenyl-9-fluorenyl cation (C^+, violet line) has a highest conductance state. The other species were HOMO-dominated electron transmission and further comparisons are provided below.

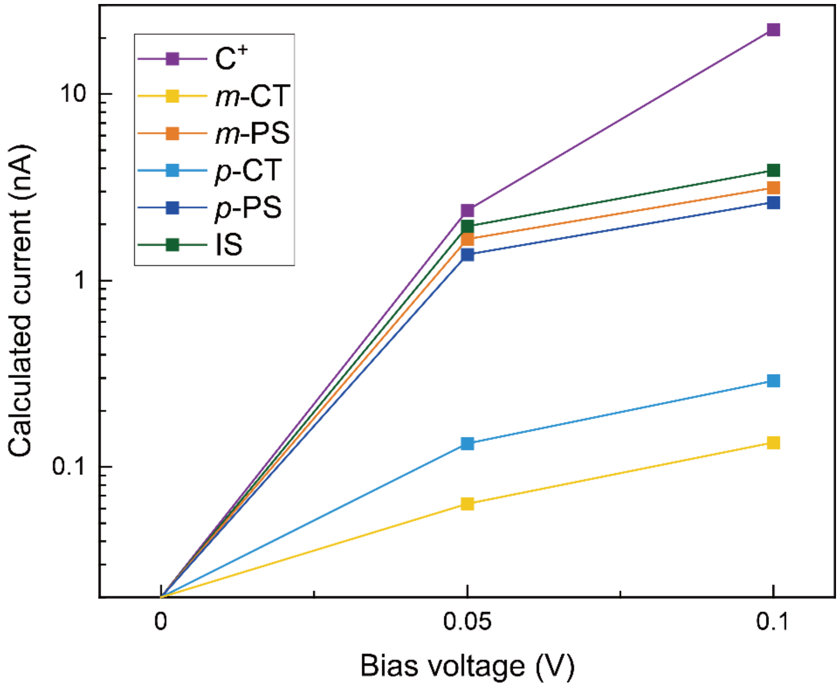

Fig. S16. Calculated *I*−*V* curves of the six intermediates. The conductance from high to low is: C^+, IS, *m*-PS, *p*-PS, *p*-CT, and *m*-CT, respectively. It should mention that to correct the systematic error of the computational simulation, we consider only the IS current (with addition of excess acrylic acid) and extract a factor which matches the value of the calculated current with that of the measured current. We then apply the same factor to the calculated *I*−*V* curves of the other species.

5. The measurements of the reaction between acrylic acid and isoprene over time

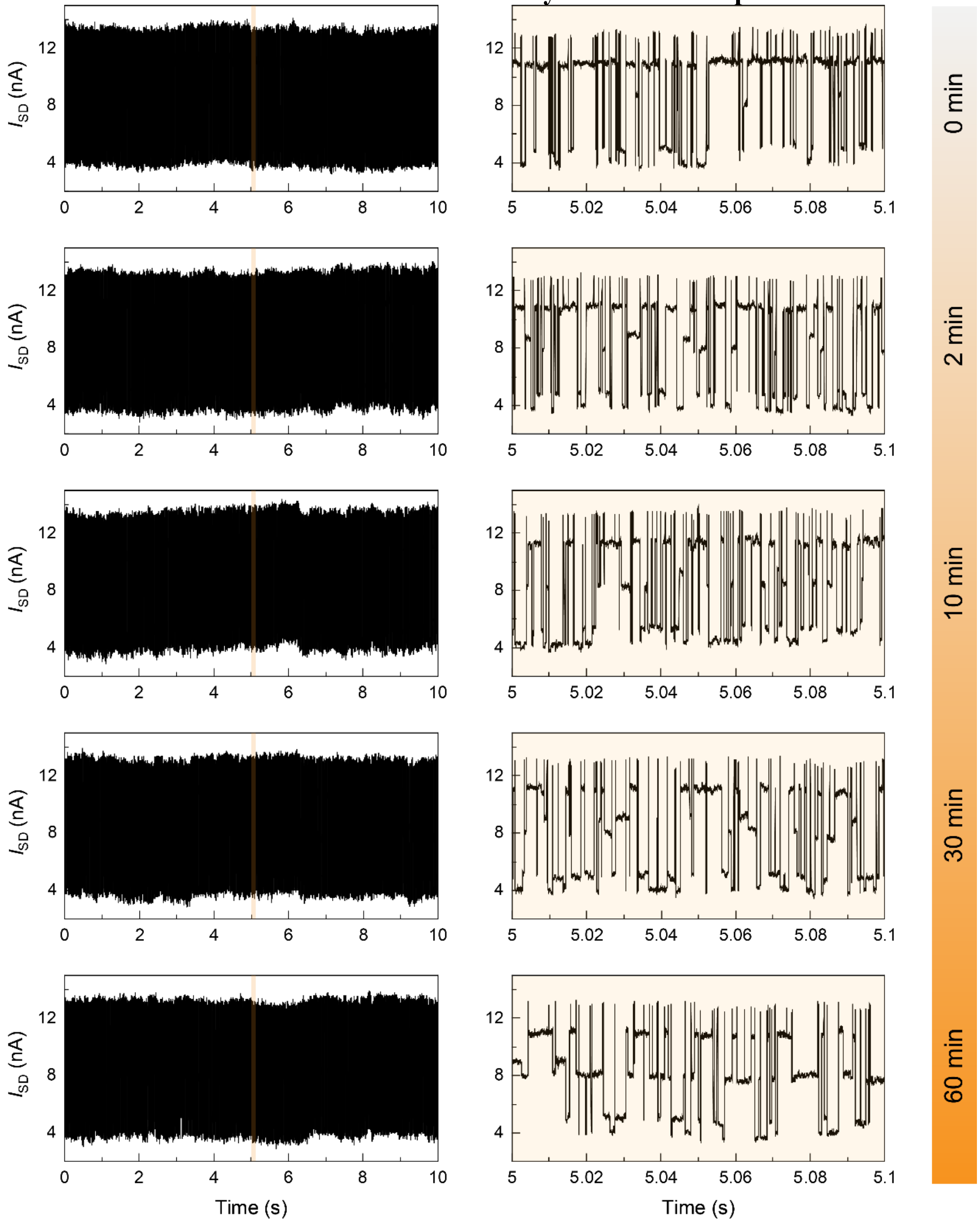

Fig. S17. The reaction signal monitored by the C⁺ molecular bridge over time. The Diels-Alder cycloaddition between acrylic acid (1 mM) and isoprene (1 mM) in trifluoroacetate was monitored at 343 K and 0.1 V bias voltage. After the addition of the substrate, the reaction signal was monitored and recorded at 0 min, 2 min, 10 min, 30 min, and 60 min, respectively. The corresponding enlarged images were provided in the right panel. The statistical results were provided in Fig. S5C−G.

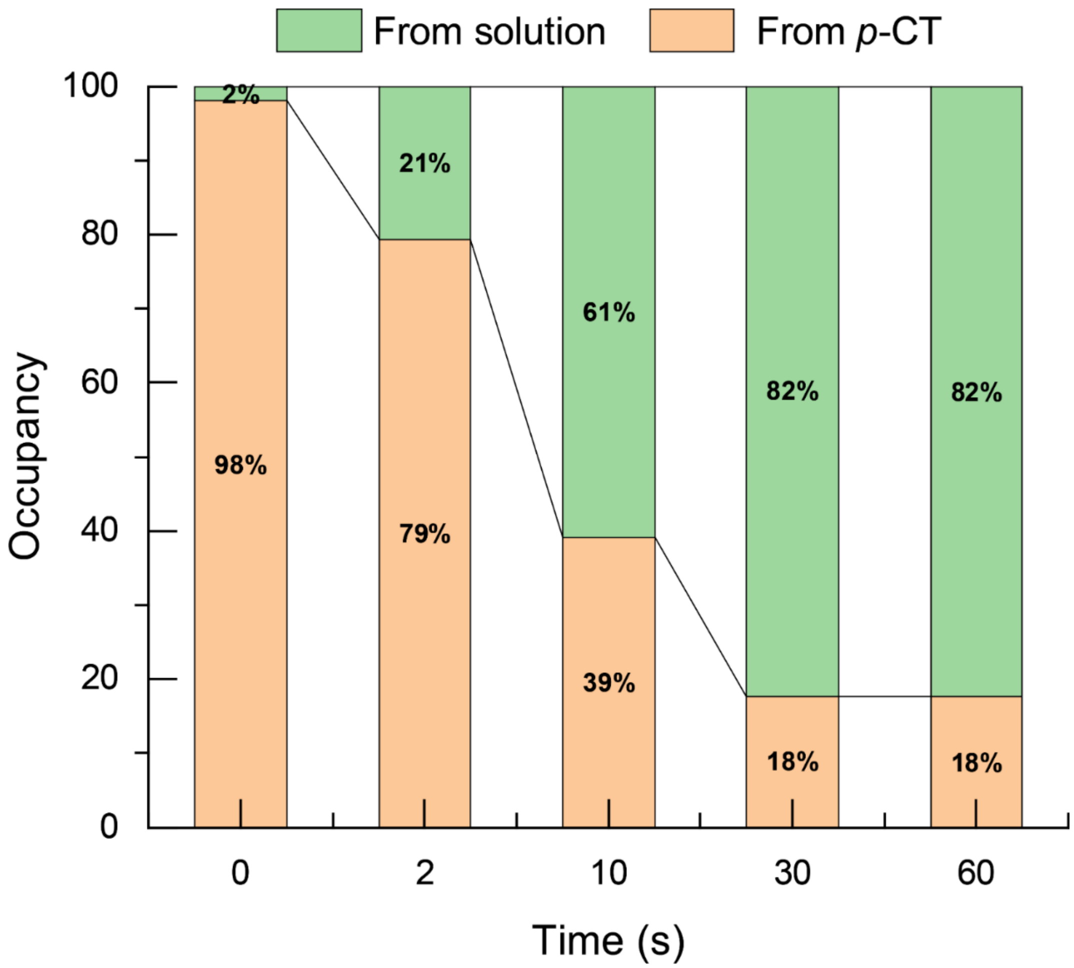

Fig. S18. The statistics on the trajectories of *p*-PS over time. According to the time sequence relationship, the numbers of the conversion from C^+ and *p*-CT to *p*-PS were recorded, which represent the reaction trajectories in solution and SMJs, respectively. With increased product molecules from the solution, the occupancy of *p*-PS detected by SMJs increased (Fig. S5C−G), which were mainly contributed by the trajectories originating from the C^+ state directly.

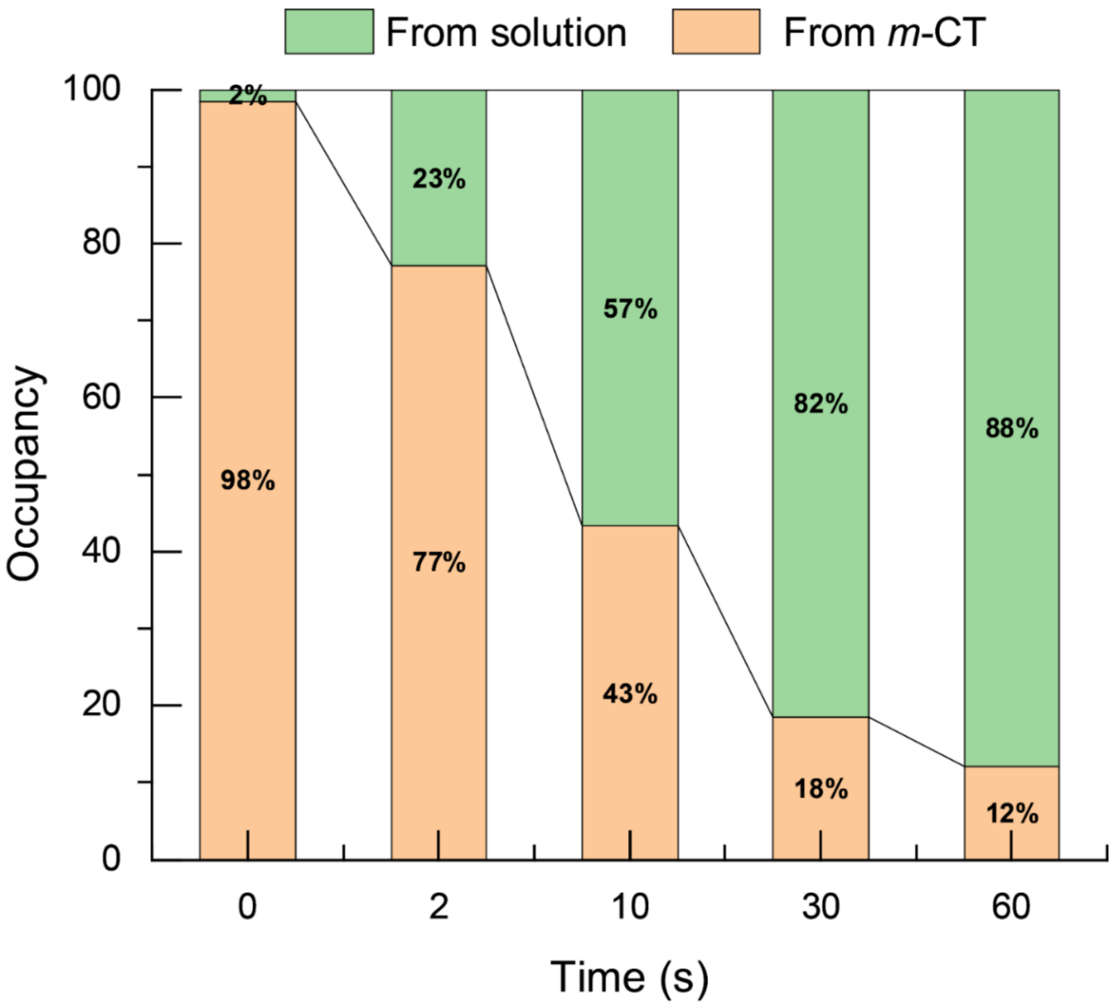

Fig. S19. The statistics on the trajectories of *m*-PS over time. According to the time sequence relationship, the conversion numbers from C^+ and *m*-CT to *m*-PS were recorded, which represent the reaction trajectories in solution and SMJs, respectively. With increased product molecules from the solution, the occupancy of *m*-PS detected by SMJs increased (Fig. S5C−G), which were mainly contributed by the trajectories originating from the C^+ state directly.

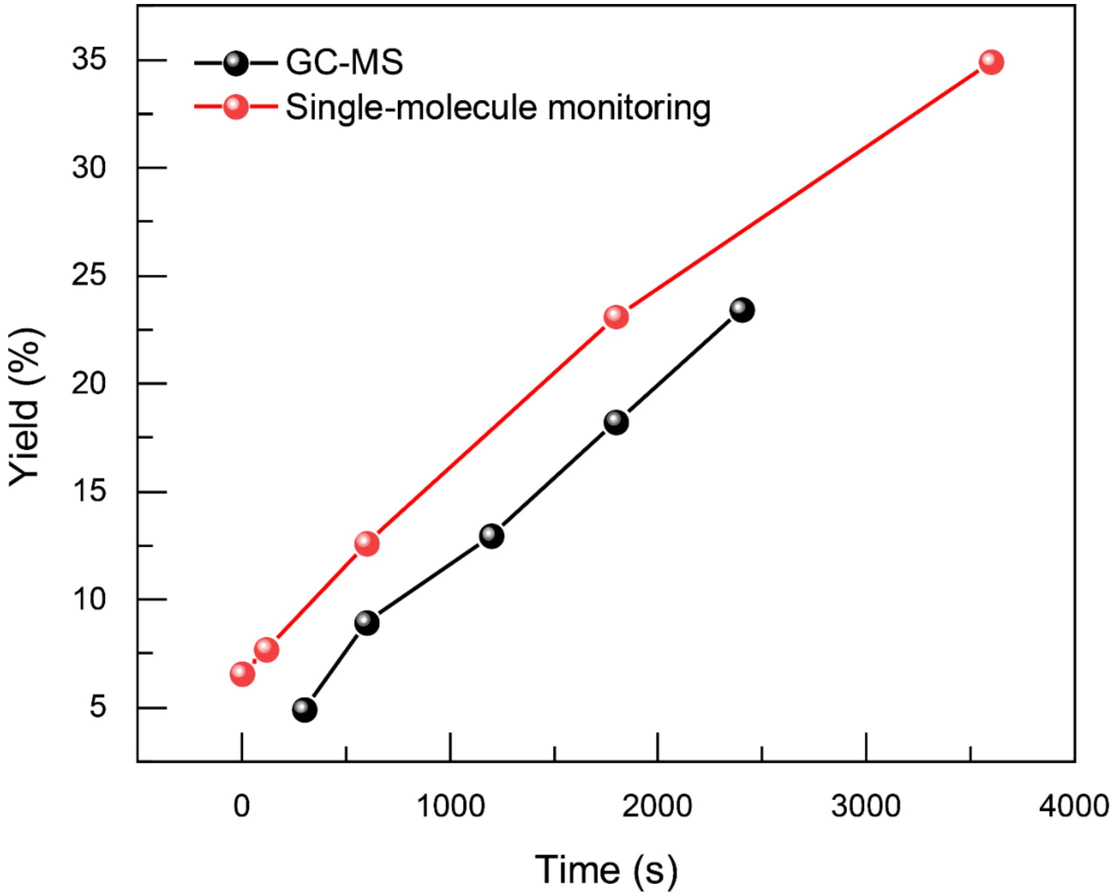

Fig. S20. The yield of the Diels-Alder reaction over time detected by GC-MS and SMJs. The yield in SMJs was calculated by the ratio of the statistical gaussian peak area (Fig. S5C−G), while the yield in macroscopic experiment was obtained according to the *n*-decane internal standard in GC. Similar trends indicate the reliability of single-molecule monitoring. The higher reaction rate detected by the SMJ originates from the catalysis in the junction by the EEF.

6. The assignments of the conductance states in *para* & *meta* and *R* & *S* paths

The monitoring of the species during the reaction was based on the association-dissociation of the C^+ molecular bridge. Therefore, the assignments of the conductance states with spin injection can also be realized by adding the control standard chiral compounds (Fig. S21). First of all, with the +2 T magnetization of the Ni electrode, the routine reaction shows 11 current states (Fig. S21, the first row), implying the formation of IS, *m*-PS, *p*-PS, *m*-CT and *p*-CT states. Then, the only addition of acrylic acid shows the formation of the IS state. As we discussed in the main text, although the absolute configuration (*re* or *si*) of the IS was not clear, the distinction of their conversion trajectories to the target chirality met our requirement for on-line control. Furthermore, the addition of the (1*R*)-4-methyl-3-cyclohexene-1-carboxylic acid supports the assignment to the *R*-*p*-PS state (The peak fitted by a blue dash line). The other peak fitted with a black dash line was the association of (1*R*)-4-methyl-3-cyclohexene-1-carboxylic acid with C^+ at the other face, which was not observed in the routine reaction (Fig. S21, the first row) due to the close relationship between the configuration of IS and subsequent formed CT and PS (discussed in the main text). The addition of racemic 4-methyl-3-cyclohexene-1-carboxylic acid further provides the assignment of the *S*-*p*-PS state and also supports the above assignment of the *R*-*p*-PS state. Similarly, we also synthesized the standard (1*R*)-3-methylcyclohex-3-ene-1-carboxylic acid, which supports the assignment to the *R*-*m*-PS state (The peak fitted by an orange dash line). The addition of racemic 3-methylcyclohex-3-ene-1-carboxylic acid further provides the assignment of the *S*-*m*-PS state. The corresponding CT states can be assigned by the statistical conversion time sequence (Figs. S22−24). In addition, the flipping of the conductance of the enantiomers at an opposite magnetic field could also be supported by the addition of the standard chiral samples (Figs. S25−28), showing the reliability of monitoring chirality changes by the CISS effect.

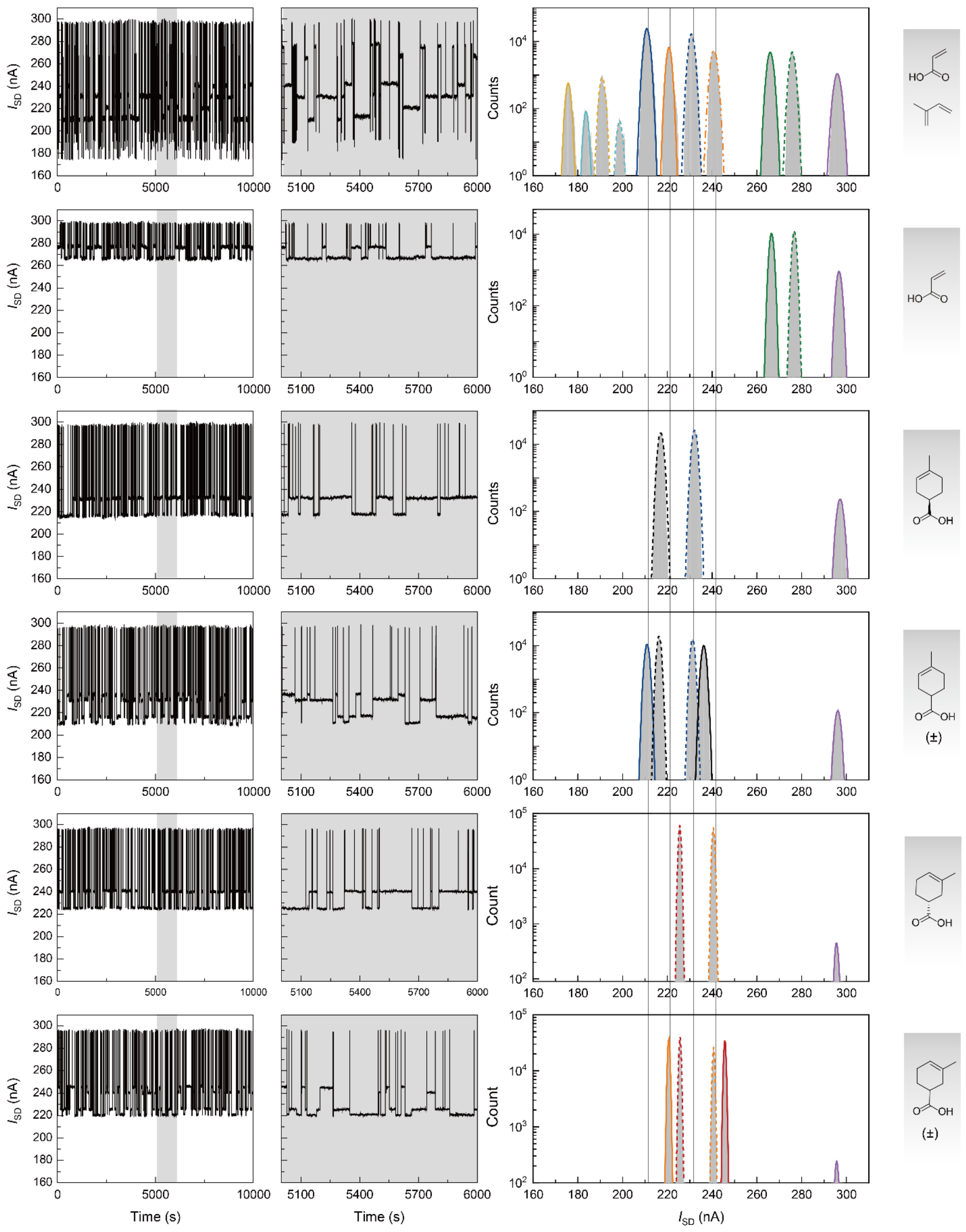

Fig. S21. Control experiments of the reaction between acrylic acid and isoprene with spin injection. The Ni electrode was magnetized with a +2 T field to produce/detect spin-polarized

currents. By comparing *I*−*t* curves at 1 V and 100 K with the addition of only acrylic acid (1 mM, the second row), (1*R*)-4-methyl-3-cyclohexene-1-carboxylic acid (1 mM, the third row), racemic 4-methyl-3-cyclohexene-1-carboxylic acid (1 mM, the 4th row), (1*R*)-3-methylcyclohex-3-ene-1-carboxylic acid (1 mM, the 5th row) or racemic 3-methylcyclohex-3-ene-1-carboxylic acid (1 mM, the 6th row), the (*re*- and *si*-) IS, *S*-*p*-PS, *R*-*p*-PS, *S*-*m*-PS and *R*-*m*-PS can be assigned in reaction signals (top panel). The other states can be assigned by the statistics of the conversion relationship below.

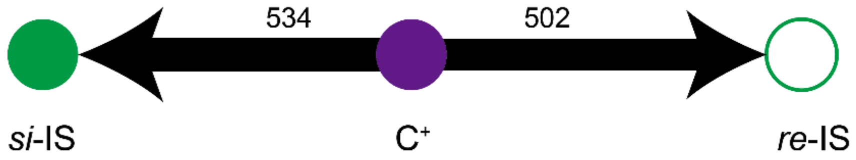

Fig. S22. The statistical conversion relationship mapping between C^+ and ISs with 1 V bias, 100 K and +2 T magnetization. The thickness of the arrow intuitively indicates the statistics of the conversion numbers, which shows an equal probability for acrylic acid to associate with C^+ at the *re*- and *si*- faces.

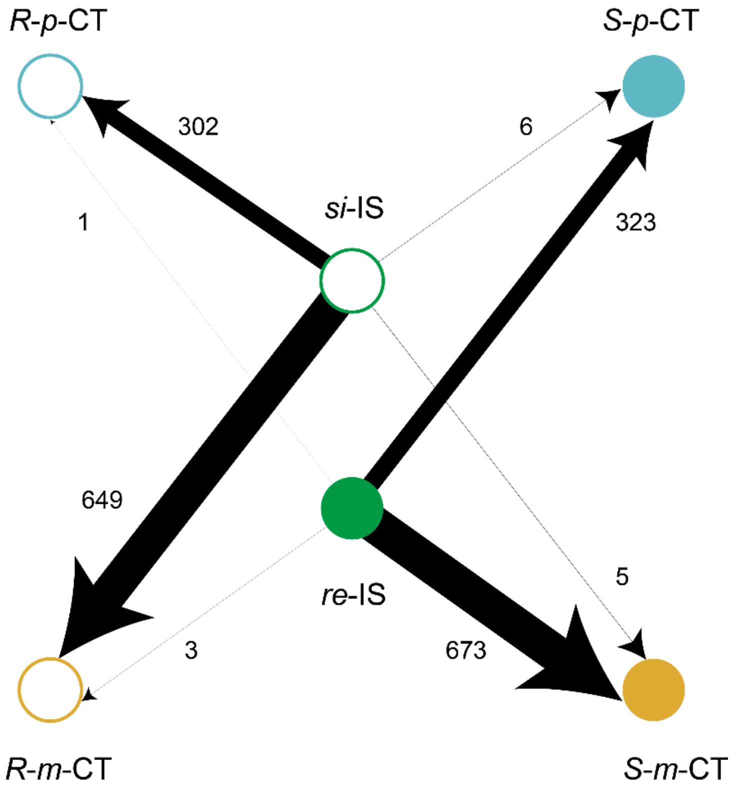

Fig. S23. The statistical conversion relationship mapping between ISs and CTs with 1 V bias, 100 K and +2 T magnetization. The thickness of the arrow intuitively indicates the statistics of the conversion numbers, which shows mutually independent transformation from prochiral ISs to chiral CTs. In addition, the higher probability was found to form the corresponding *m*-CT, which is in lines with the lower energy of *m*-CT in the energy profile.

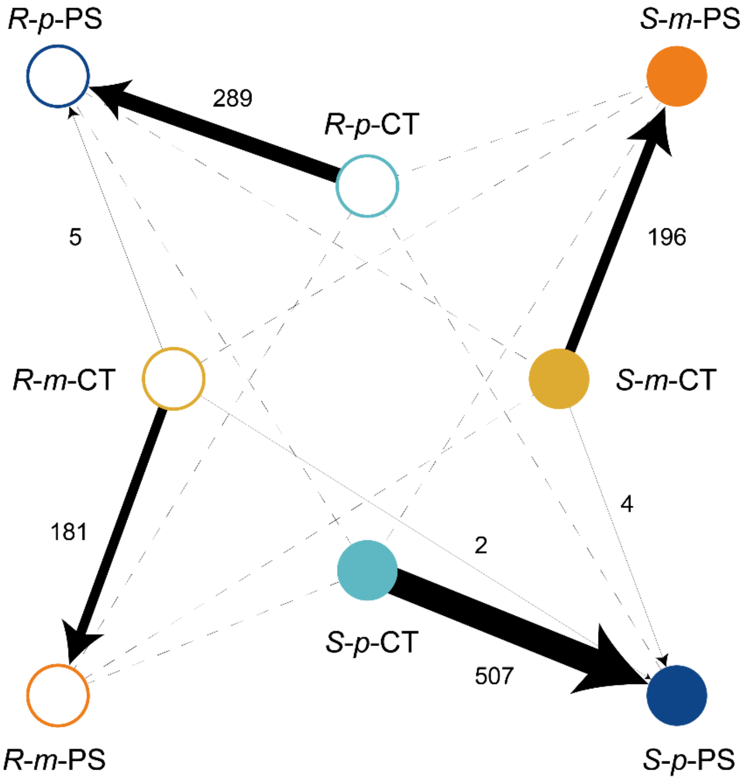

Fig. S24. The statistical conversion relationship mapping between CTs and PSs with 1 V bias, 100 K and +2 T magnetization. The thickness of the arrow intuitively indicates the statistics of the conversion numbers, which shows mutually independent transformation from chiral CTs to chiral PSs and supports the assignments of the conductance states. In addition, the higher probability was found to form the corresponding *p*-PS, which is in lines with the lower TSs in the energy profile. Dash line: no conversion between the two species.

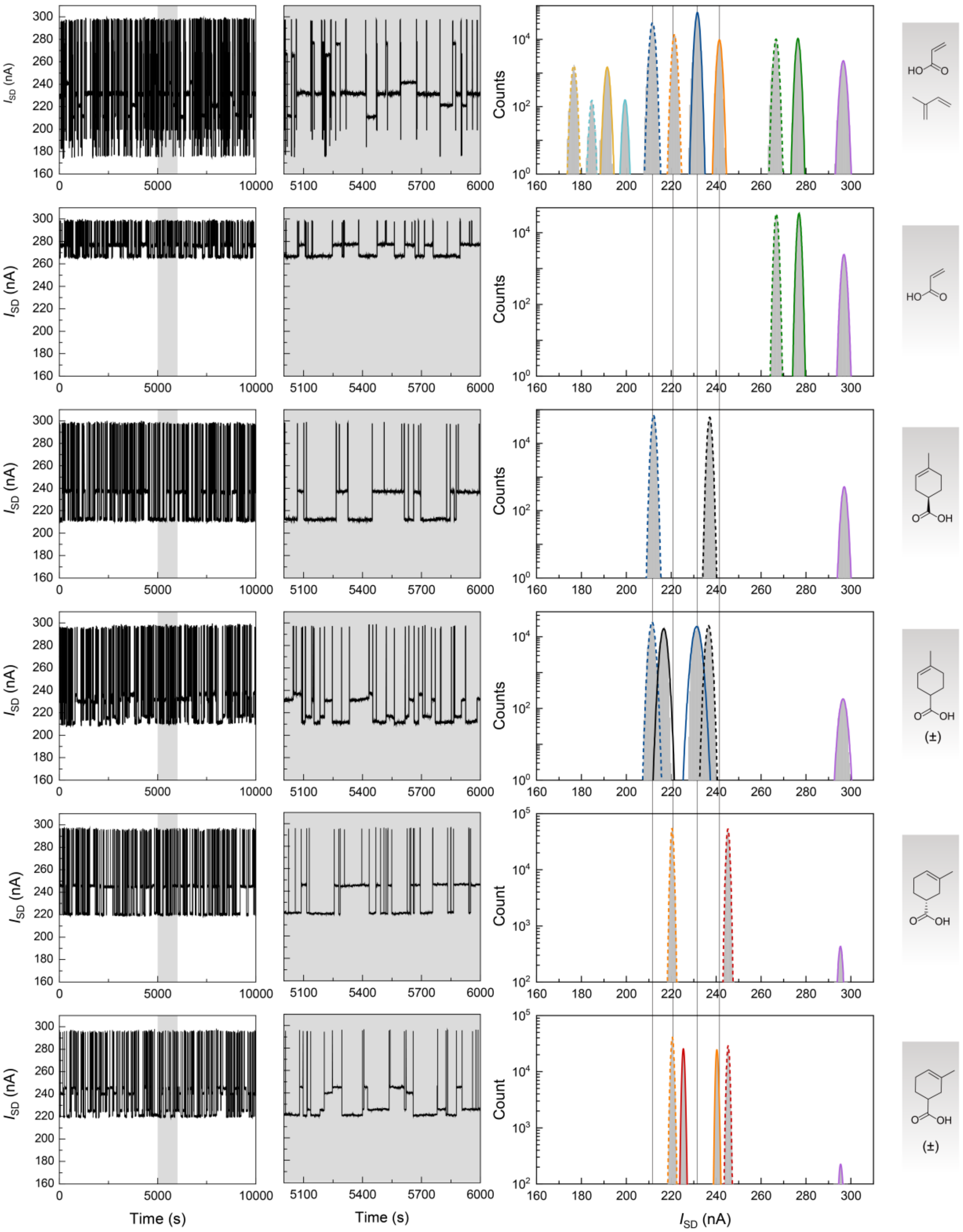

Fig. S25. Control experiments of the reaction between acrylic acid and isoprene with the spin injection through a −2T magnetized Ni electrode. By comparing I−t curves at 1 V and 100 K with the addition of only acrylic acid (1 mM, the second row), (1R)-4-methyl-3-cyclohexene-1-carboxylic acid (1 mM, the third row), racemic 4-methyl-3-cyclohexene-1-carboxylic acid (1 mM,

the 4th row), (1*R*)-3-methylcyclohex-3-ene-1-carboxylic acid (1 mM, the 5th row) or racemic 3-methylcyclohex-3-ene-1-carboxylic acid (1 mM, the 6th row), the (*re*- and *si*-) IS, *S*-*p*-PS, *R*-*p*-PS, *S*-*m*-PS and *R*-*m*-PS can be assigned in reaction signals (top panel). The other states can be assigned by the statistics of the conversion relationship below.

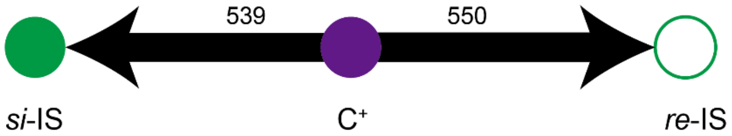

Fig. S26. The statistical conversion relationship mapping between C⁺ and ISs at 1 V bias, 100 K and −2 T magnetization. The thickness of the arrow intuitively indicates the statistics of the conversion numbers, which shows an equal probability for acrylic acid to associate with C^+ at the *re-* and *si-* faces.

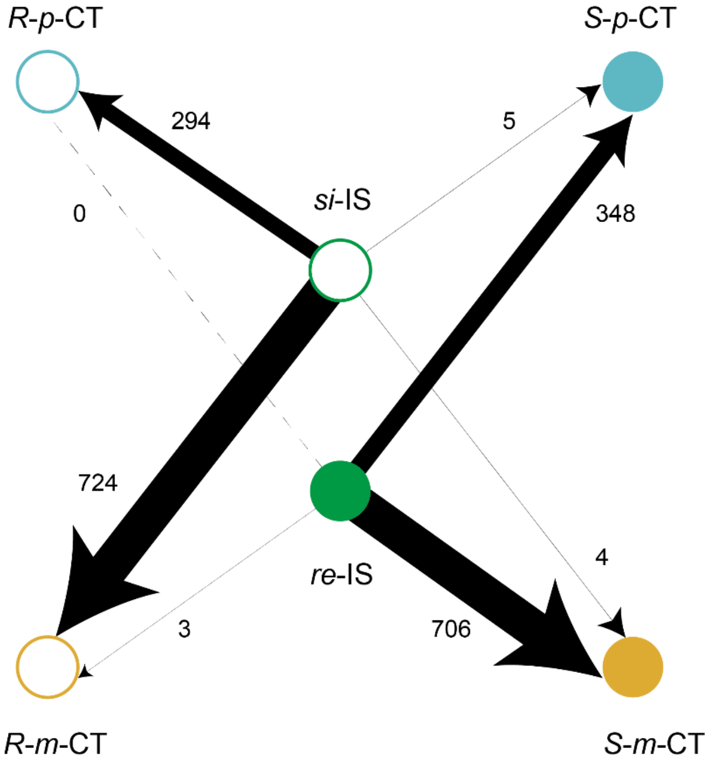

Fig. S27. The statistical conversion relationship mapping between ISs and CTs at 1 V bias, 100 K and −2 T magnetization. The thickness of the arrow intuitively indicates the statistics of the conversion numbers, which shows mutually independent transformation from prochiral ISs to chiral CTs. In addition, the higher probability was found to form the corresponding *m*-CT, which is in lines with the lower energy of *m*-CT in the energy profile.

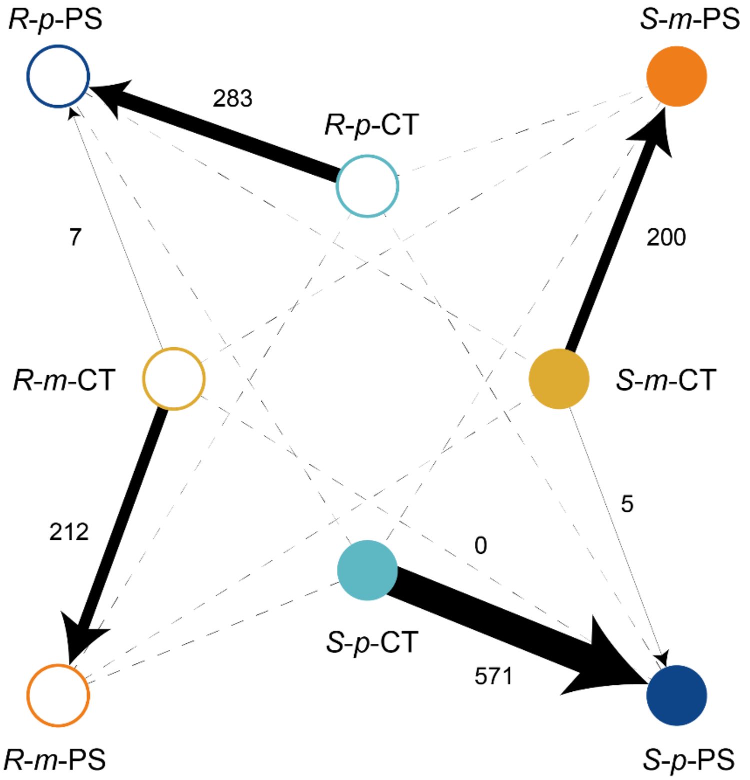

Fig. S28. The statistical conversion relationship mapping between CTs and PSs at 1 V bias, 100 K and −2 T magnetization. The thickness of the arrow intuitively indicates the statistics of the conversion numbers, which shows mutually independent transformation from chiral CTs to chiral PSs. In addition, the higher probability was found to form the corresponding *p*-PS, which is in lines with the lower TSs in the energy profile. Dash line: no conversion between the two species.

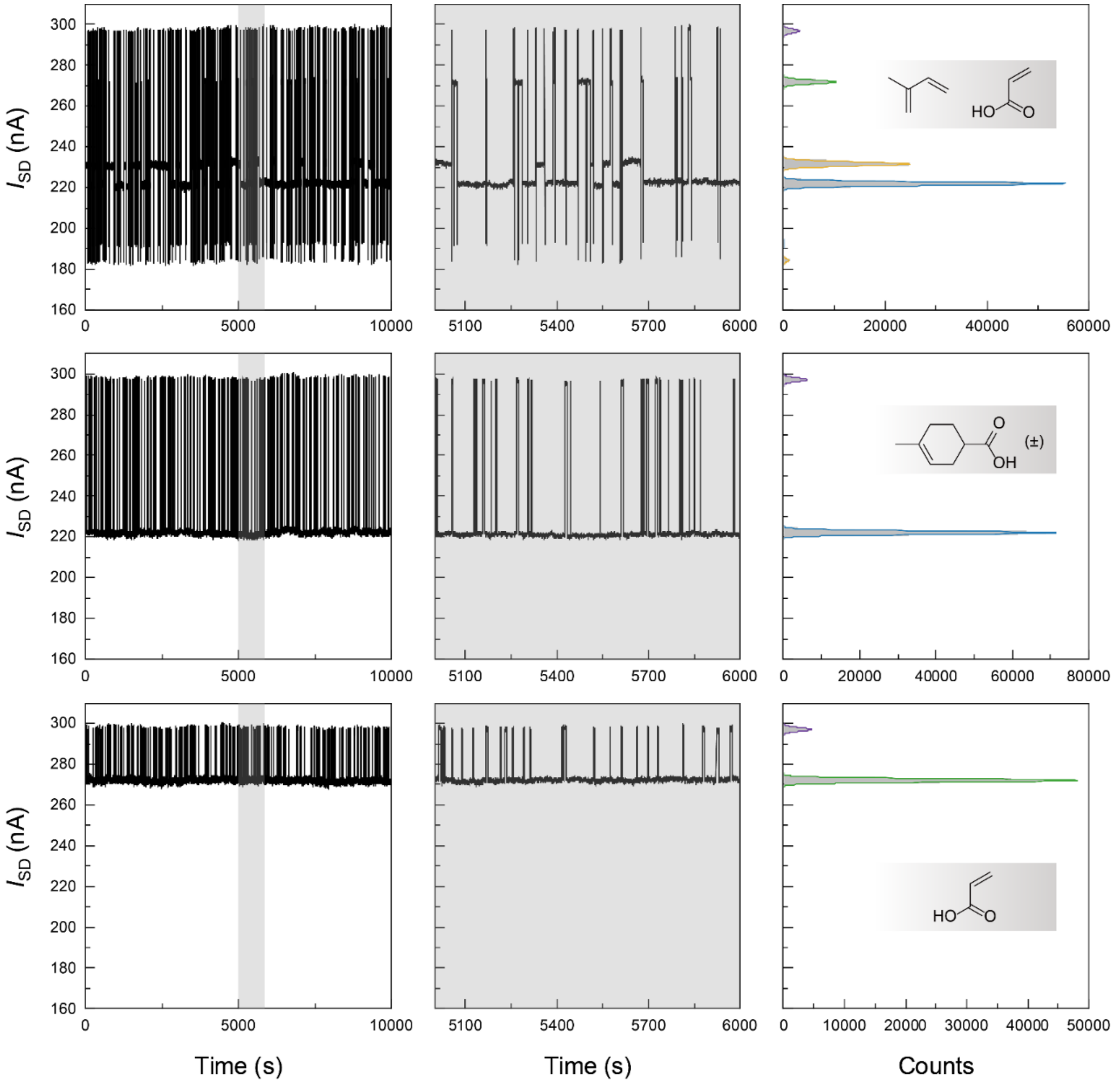

Fig. S29. Control experiments of the reaction between acrylic acid and isoprene with an unmagnetized Ni electrode. By comparing I–t curves at 1 V and 100 K with addition of only racemic 4-methyl-3-cyclohexene-1-carboxylic acid (1 mM, middle panel) and acrylic acid (1 mM, bottom panel), the IS and *p*-PS can be assigned in reaction signals (top panel). The chirality cannot be effectively distinguished.

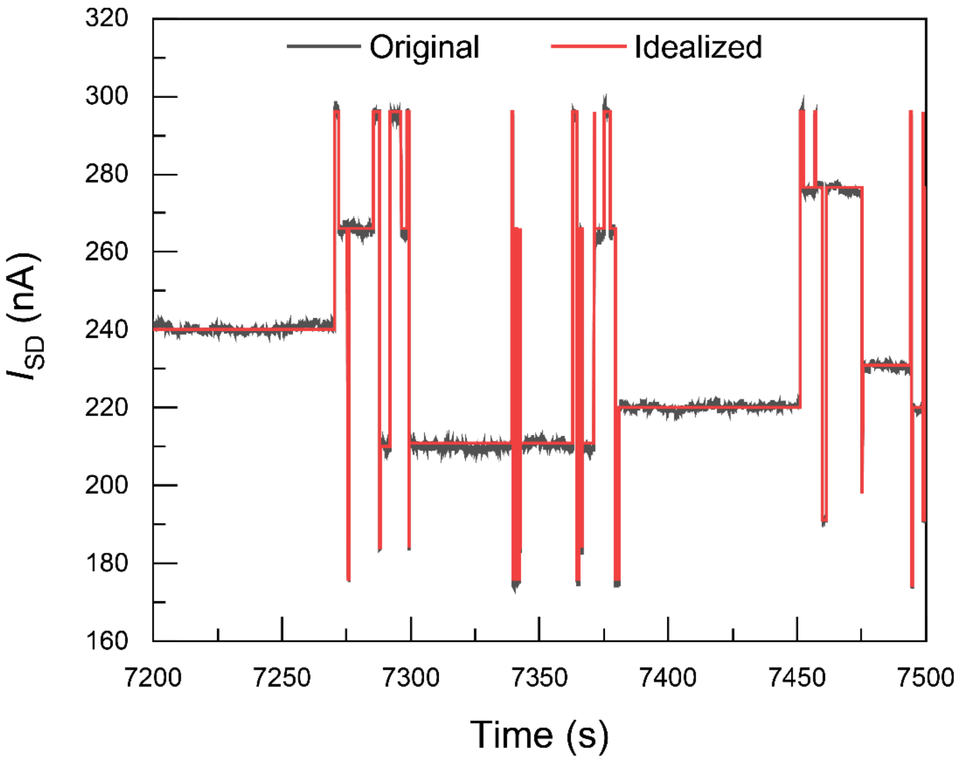

Fig. S30. The idealized fitting of the *I*–*t* curve. The idealization of the *I*–*t* curve can be used to extract the time sequence relationship and time intervals of current levels.

7. Optical characterization of the products from the Diels-Alder reaction between propiolic acid and isoprene

To characterize the product of single-molecule catalysis with an optical method, the reaction that generates fluorescent products should be chosen. Here, the reaction between propiolic acid and isoprene was studied, where trace amounts of benzoic acid (measurable fluorescence) were formed by oxygen catalysis[1]. By long-term (one week) multiple operations under O_2, the benzoic acid, as well as the corresponding *meta* and *para* configurations could be detected by the single-molecule fluorescent spectra. In comparison with the macroscopic fluorescent spectra of the standard samples (*m*-toluic acid and *p*-toluic acid), single-molecule spectra demonstrate the successful proceeding of single-molecule catalysis.

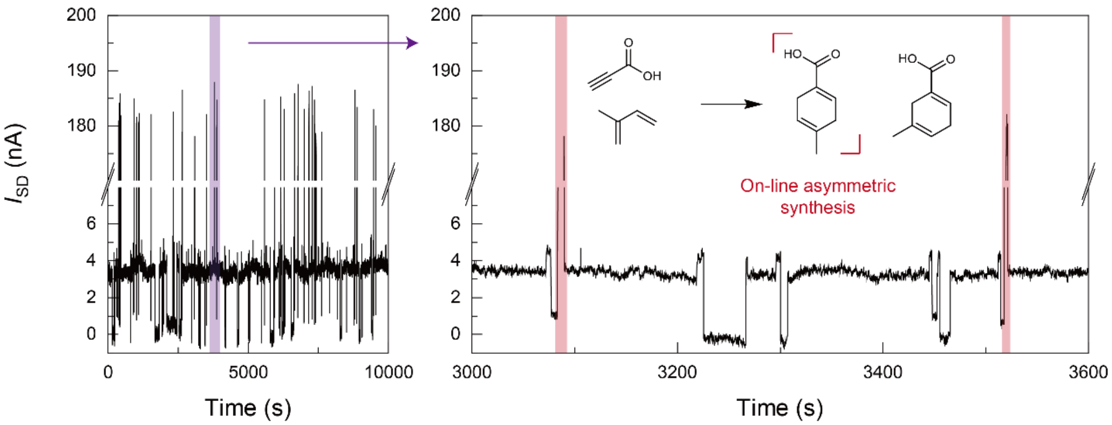

Fig. S31. Typical *I*–*t* curves of the direct synthesis of *p*-PS. The Diels-Alder cycloaddition between propiolic acid (1 mM) and isoprene (1 mM) in trifluoroacetate was monitored at 100 K and 0.1 V bias voltage by the C^+ molecular bridge. A 1 V bias voltage was applied at the *p*-CT and removed at the C^+ state to prepare a *p*-PS. The corresponding enlarged image was provided in the right panel. The red regions of the *I*–*t* curve in the right panel show the application of 1 V bias.

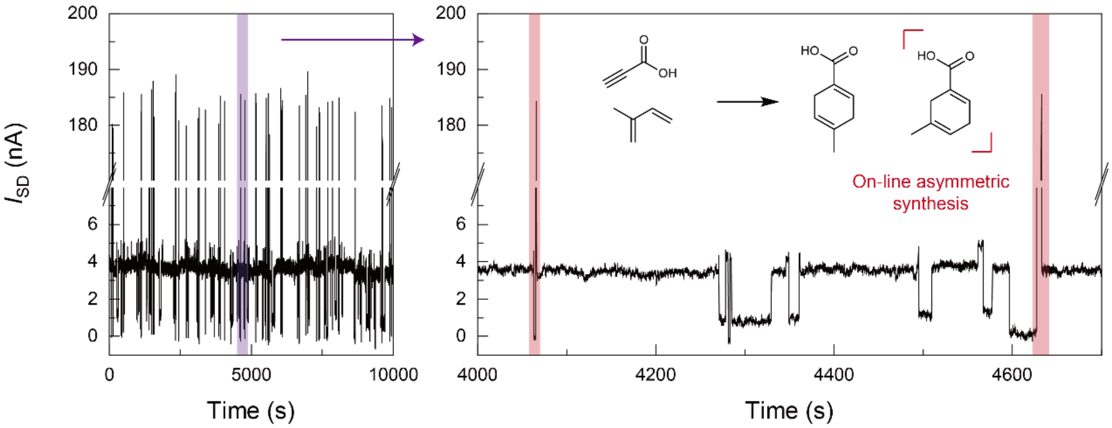

Fig. S32. Typical *I*–*t* curves of the direct synthesis of *m*-PS. The Diels-Alder cycloaddition between propiolic acid (1 mM) and isoprene (1 mM) in trifluoroacetate was monitored at 100 K and 0.1 V bias voltage by the C^+ molecular bridge. A 1 V bias voltage was applied at the *m*-CT and removed at the C^+ state to prepare a *m*-PS. The corresponding enlarged image was provided in the right panel. The red regions of the *I*–*t* curve in the right panel show the application of 1 V bias.

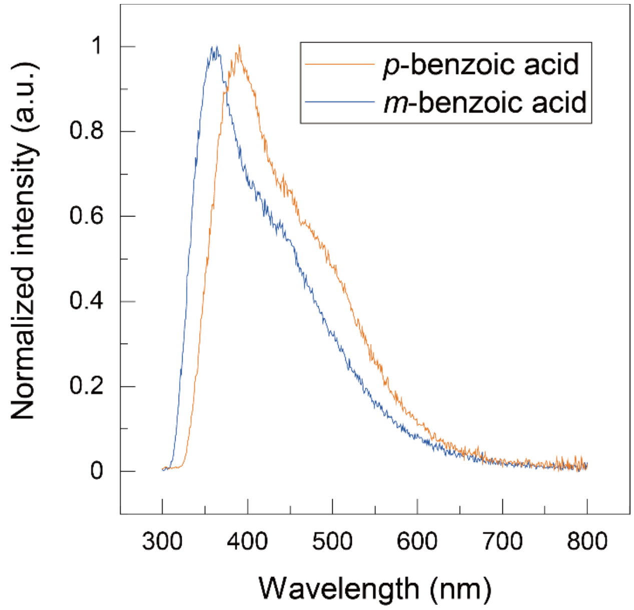

Fig. S33. Normalized fluorescent emission spectra of the standard *meta*- (i.e., *m*-toluic acid) and *para*- (i.e., *p*-toluic acid) benzoic acids. The specific peaks of *m*-toluic acid and *p*-toluic acid were found at ~360 and ~388 nm, respectively.

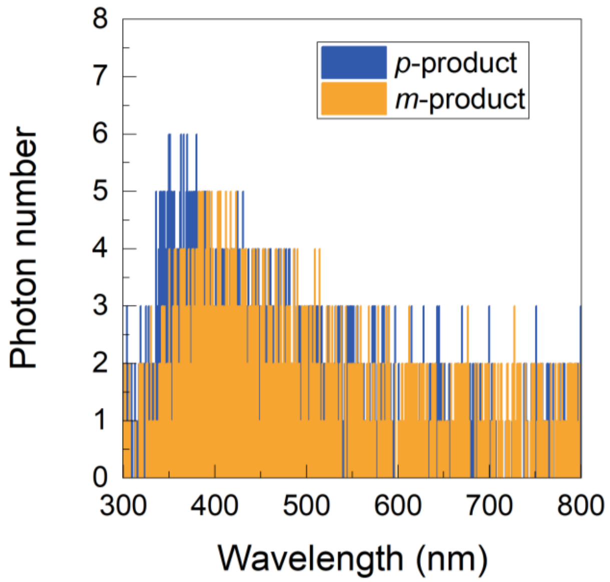

Fig. S34. Energy-resolved single-photon counting at the single-catalyst reaction site. With a 60-s exposure time, the fluorescent spectra of the synthesized products with *meta* and *para* configurations were collected, respectively. In comparison with the macroscopic fluorescent spectra of the standard samples (*m*-toluic acid and *p*-toluic acid), the statistics of single-molecule emission demonstrate the successful proceeding of single-molecule catalysis.

8. The theoretical energy potential surfaces with different EEFs

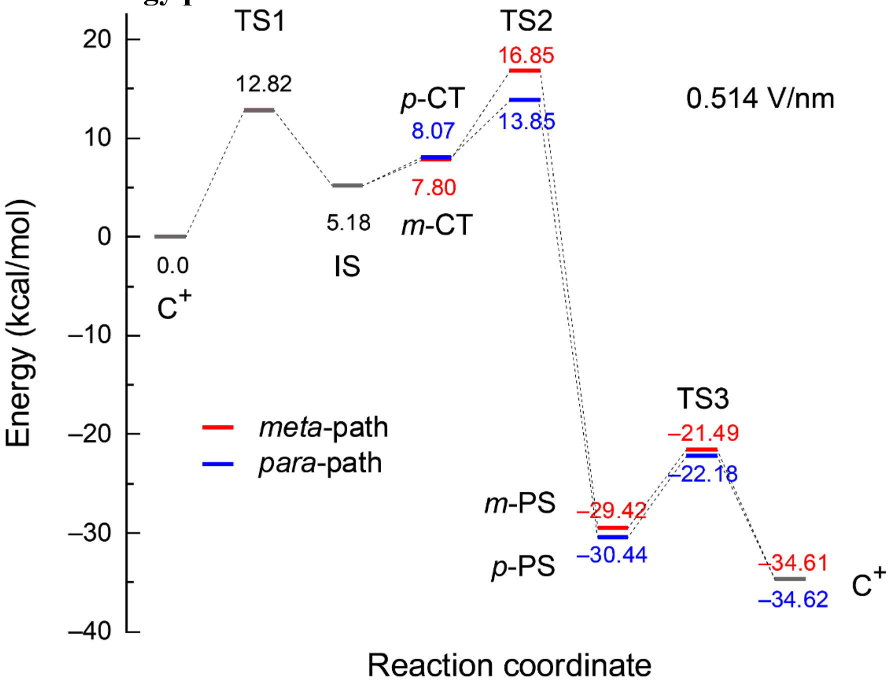

Fig. S35. The theoretical cascade reaction energy potential surface with the EEF of 0.514 V/nm.

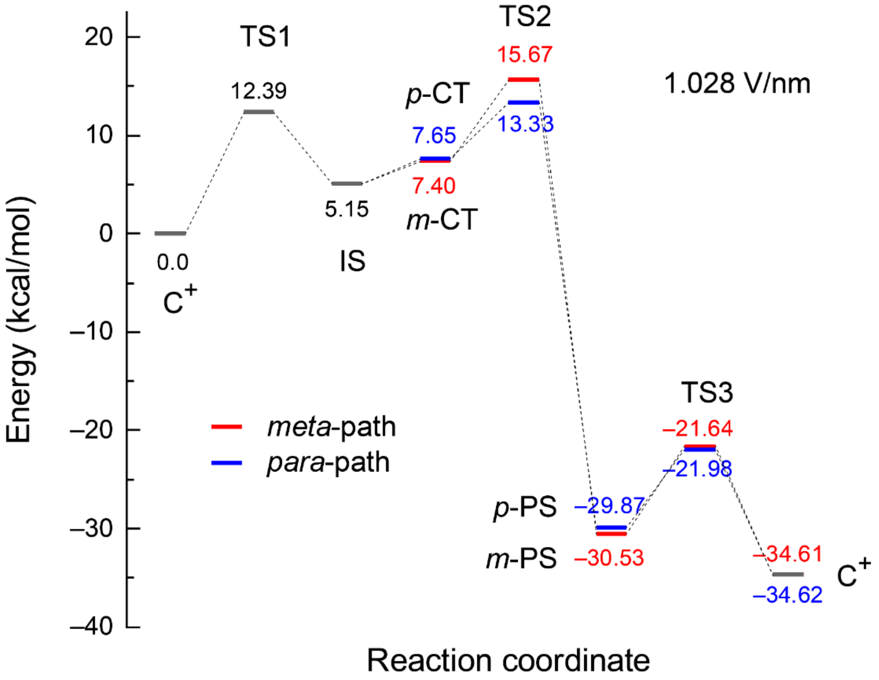

Fig. S36. The theoretical cascade reaction energy potential surface with the EEF of 1.028 V/nm.

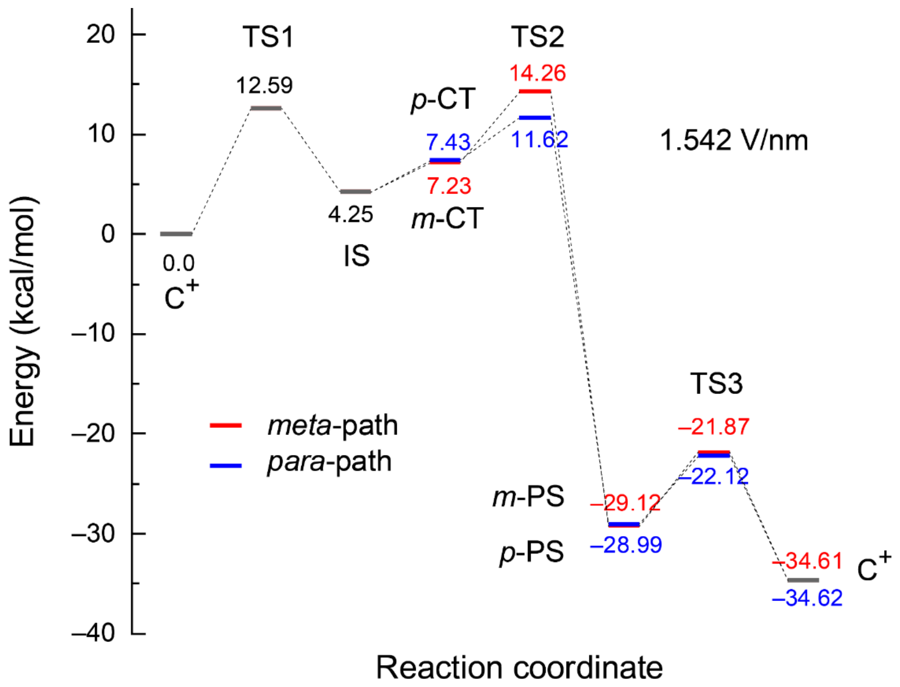

Fig. S37. The theoretical cascade reaction energy potential surface with the EEF of 1.542 V/nm.

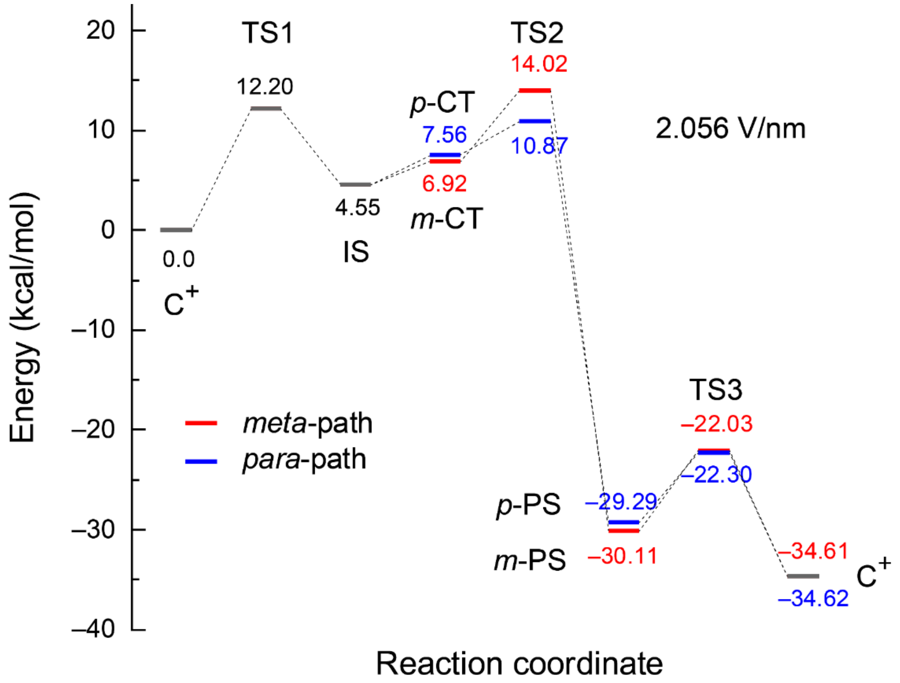

Fig. S38. The theoretical cascade reaction energy potential surface with the EEF of 2.056 V/nm.

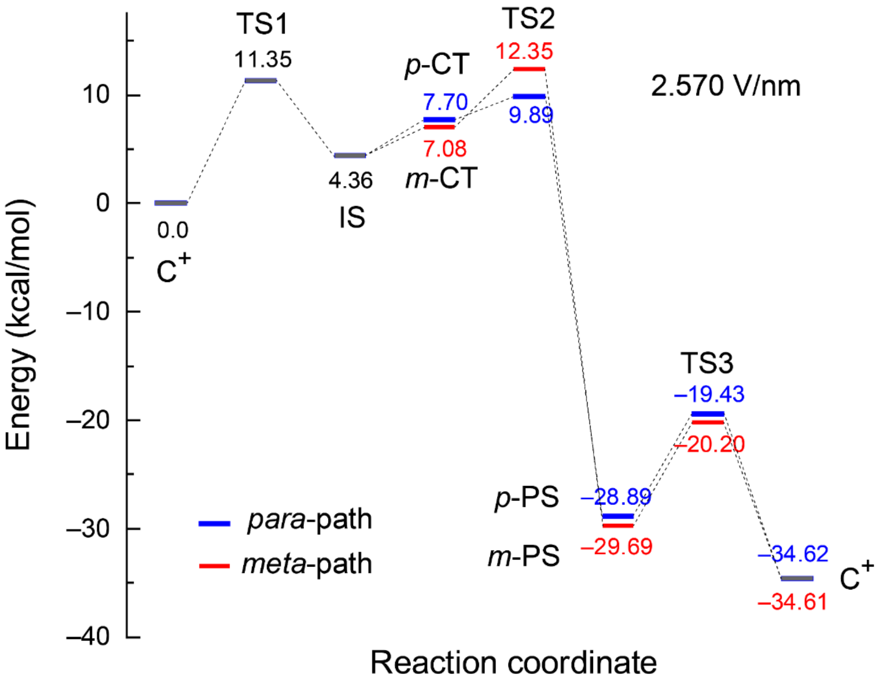

Fig. S39. The theoretical cascade reaction energy potential surface with the EEF of 2.570 V/nm.

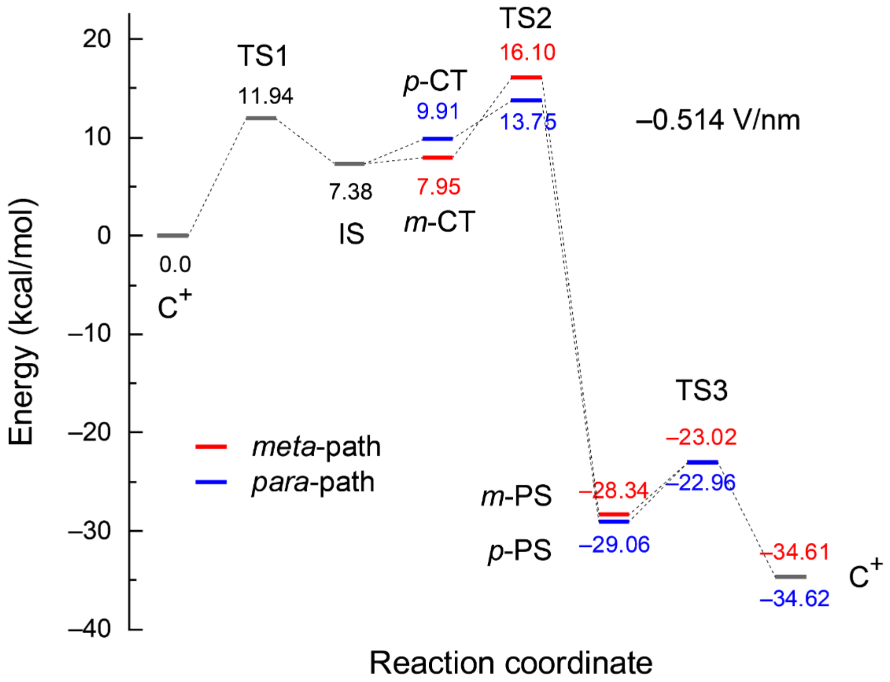

Fig. S40. The theoretical cascade reaction energy potential surface with the EEF of −0.514 V/nm.

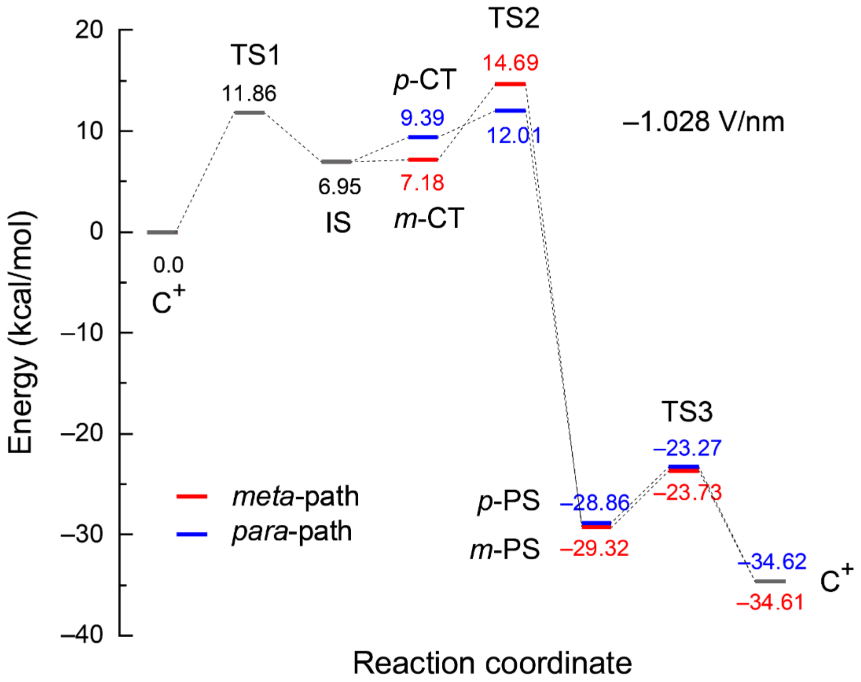

Fig. S41. The theoretical cascade reaction energy potential surface with the EEF of −1.028 V/nm.

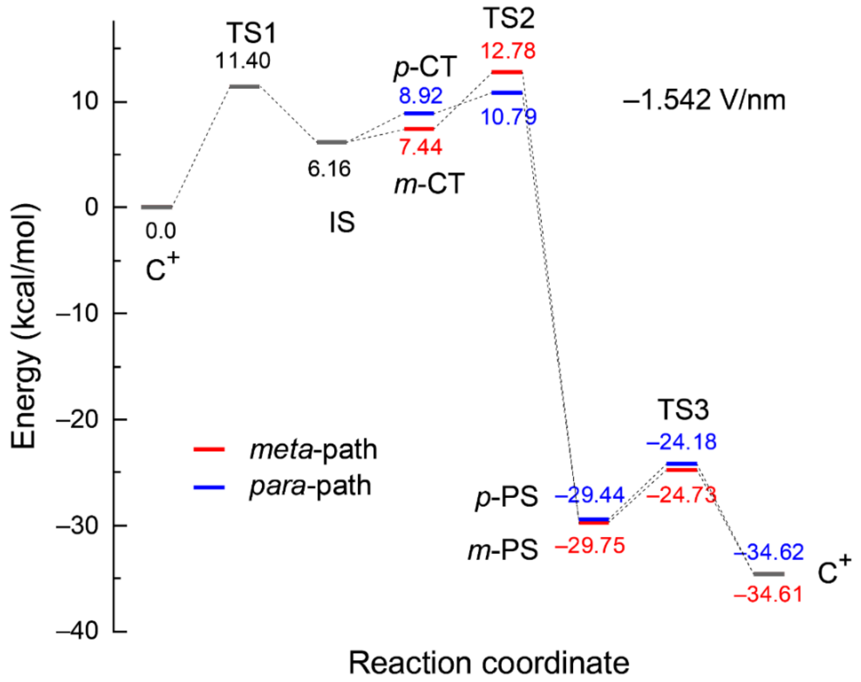

Fig. S42. The theoretical cascade reaction energy potential surface with the EEF of −1.542 V/nm.

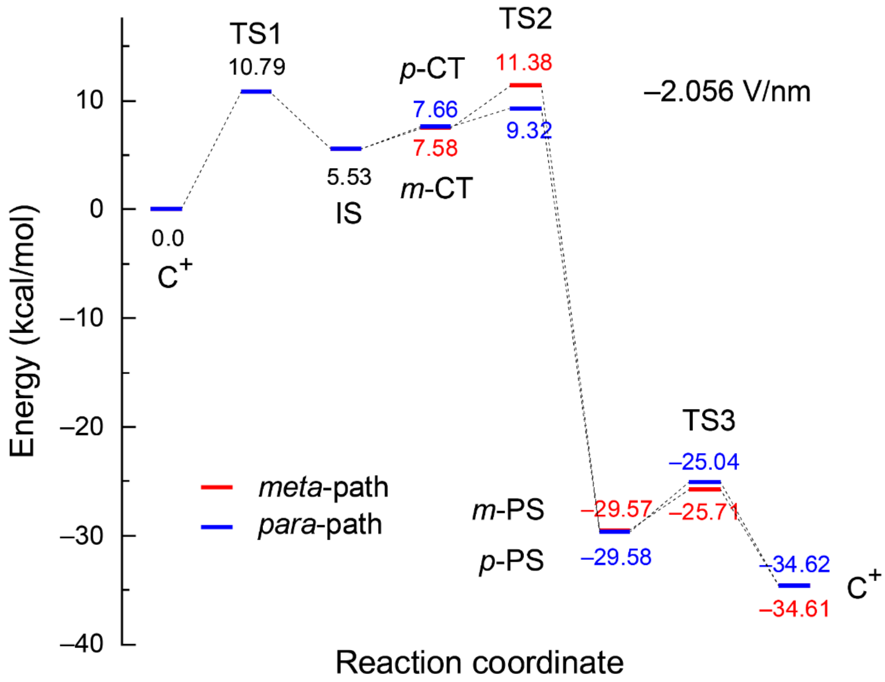

Fig. S43. The theoretical cascade reaction energy potential surface with the EEF of −2.056 V/nm.

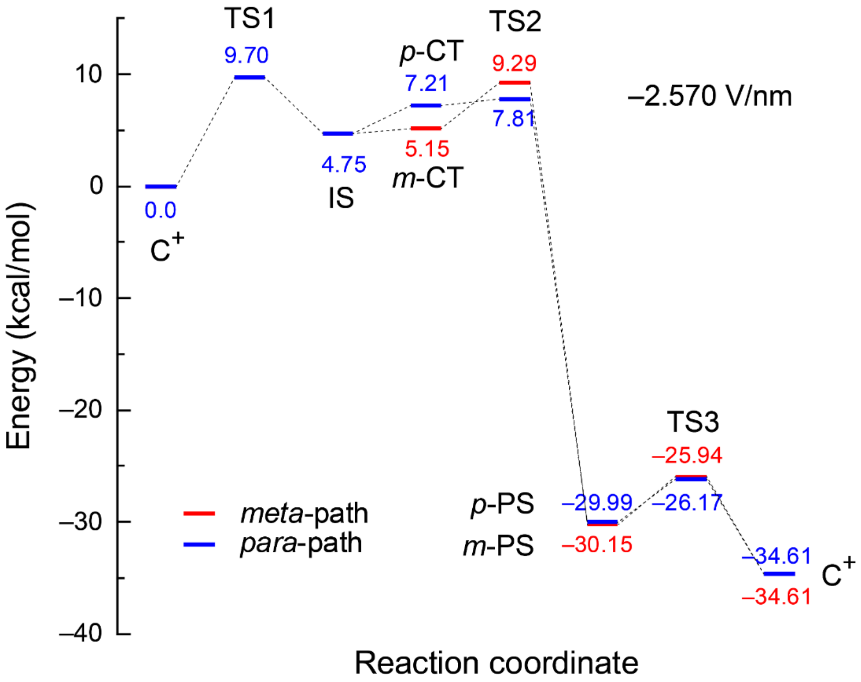

Fig. S44. The theoretical cascade reaction energy potential surface with the EEF of −2.570 V/nm.

9. Bias voltage-dependent measurements of the reaction including *para* & *meta* paths

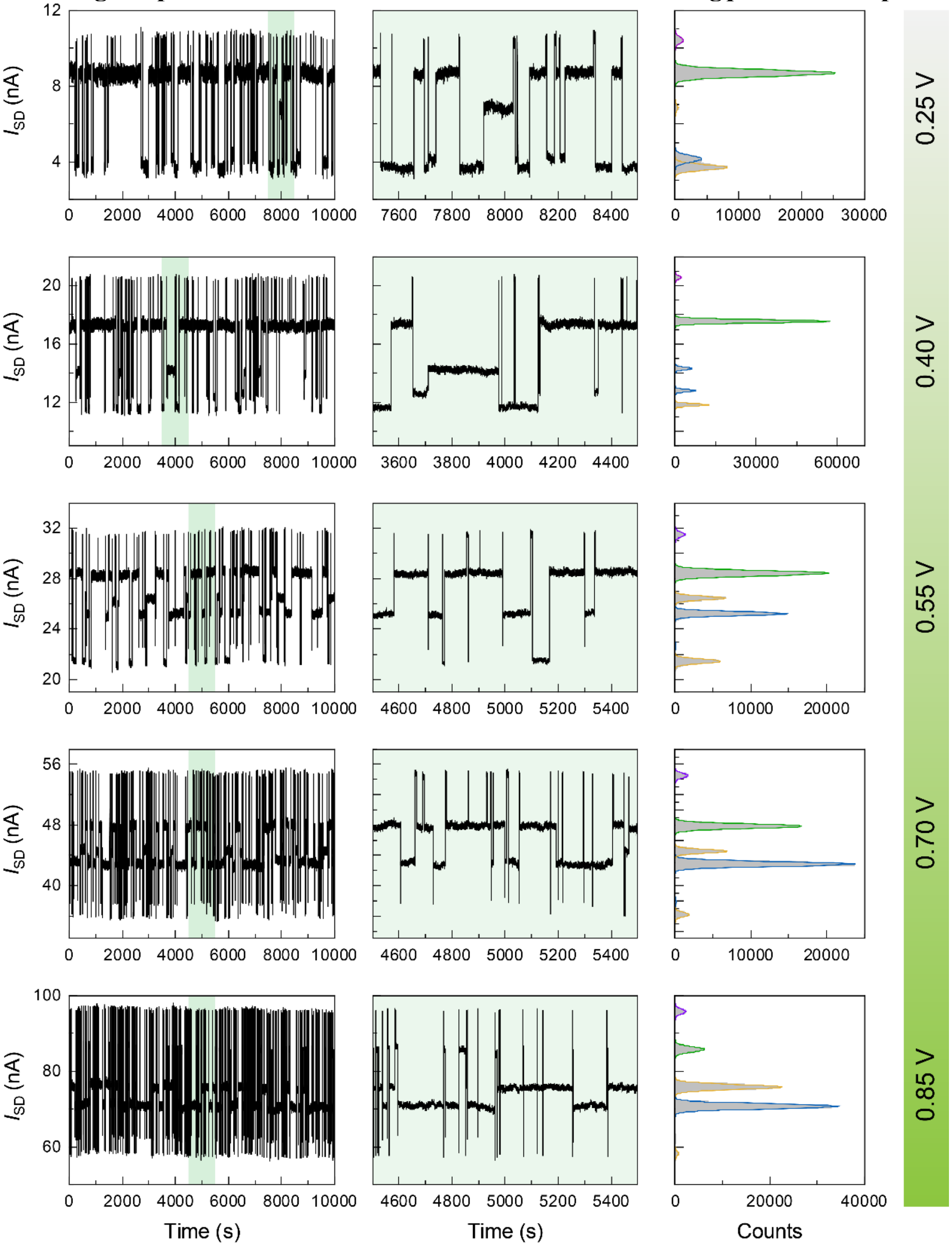

Fig. S45. The positive bias voltage dependent measurements by the C^+ molecular bridge. The Diels-Alder cycloaddition between acrylic acid (1 mM) and isoprene (1 mM) in trifluoroacetate was monitored at 100 K. The occupancy of *m*-PS and *p*-PS increased with higher positive biases, showing the driving effect on the reaction by the EEF.

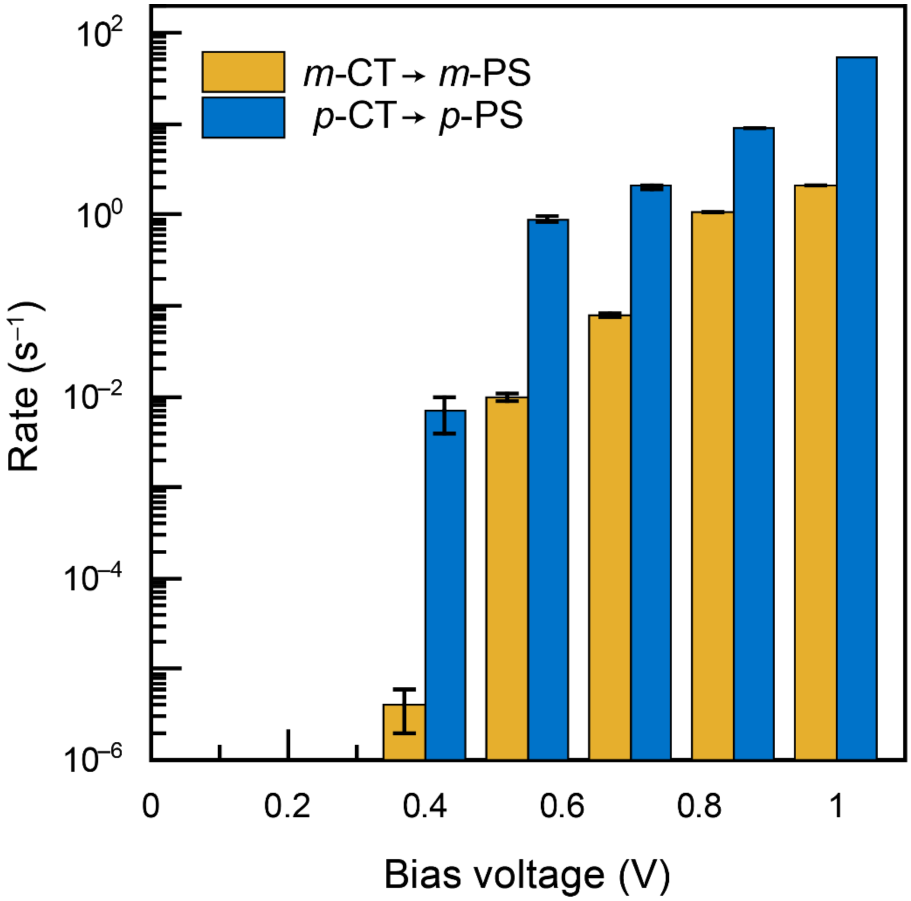

Fig. S46. The conversion rates from CT to corresponding PS at different positive bias voltages. Increased rates with higher biases show the reduction of the TS cycloaddition by the EEF.

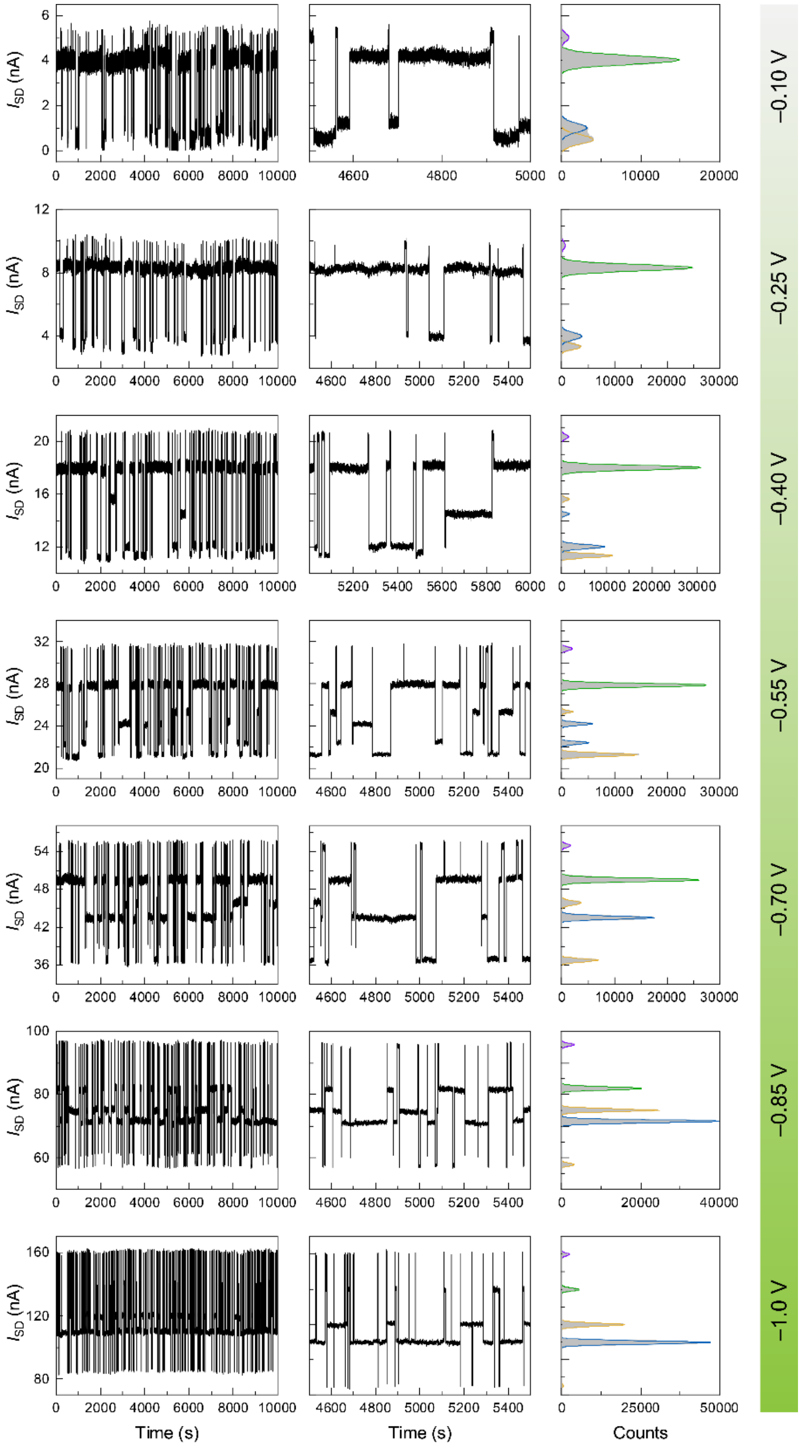

Fig. S47. The negative bias voltage dependent measurements by the C⁺ molecular bridge. The Diels-Alder cycloaddition between acrylic acid (1 mM) and isoprene (1 mM) in trifluoroacetate was monitored at 100 K. The occupancy of *m*-PS and *p*-PS increased with higher negative biases, showing the driving effect on the reaction by the EEF.

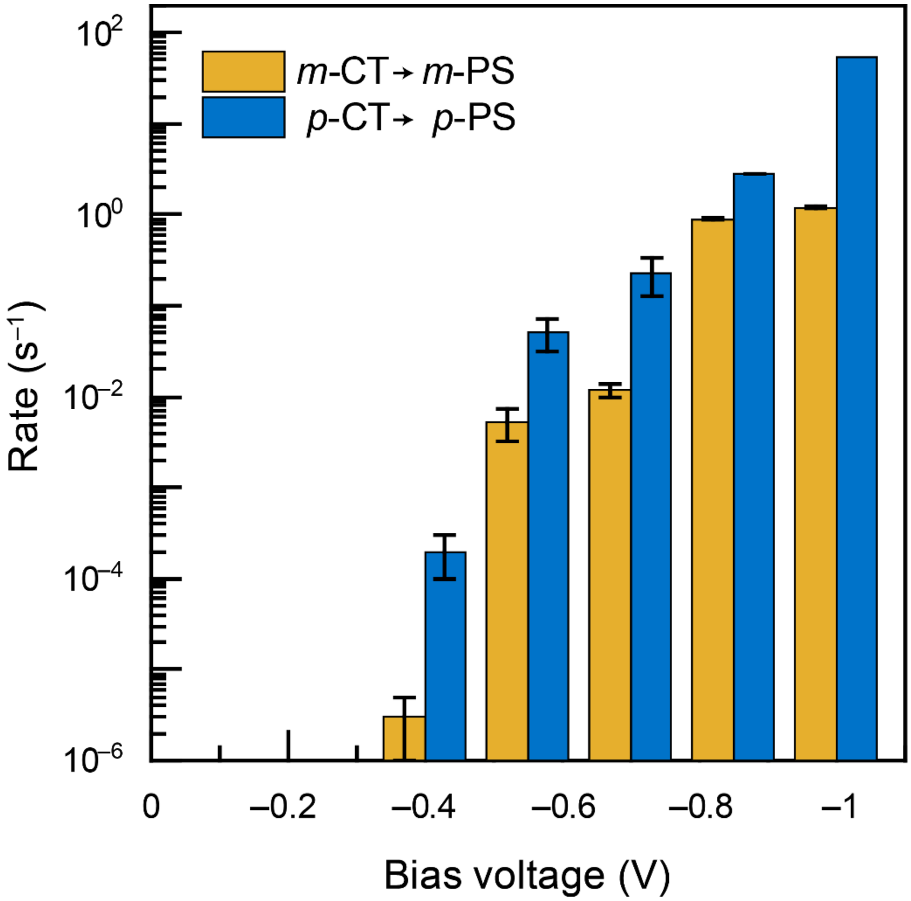

Fig. S48. The conversion rates from CT to corresponding PS at different negative bias voltages. Increased rates with higher biases show the reduction of the TS cycloaddition by the EEF.

10. Bias voltage dependent measurements of the reaction including *para* & *meta* and *R* & *S* paths

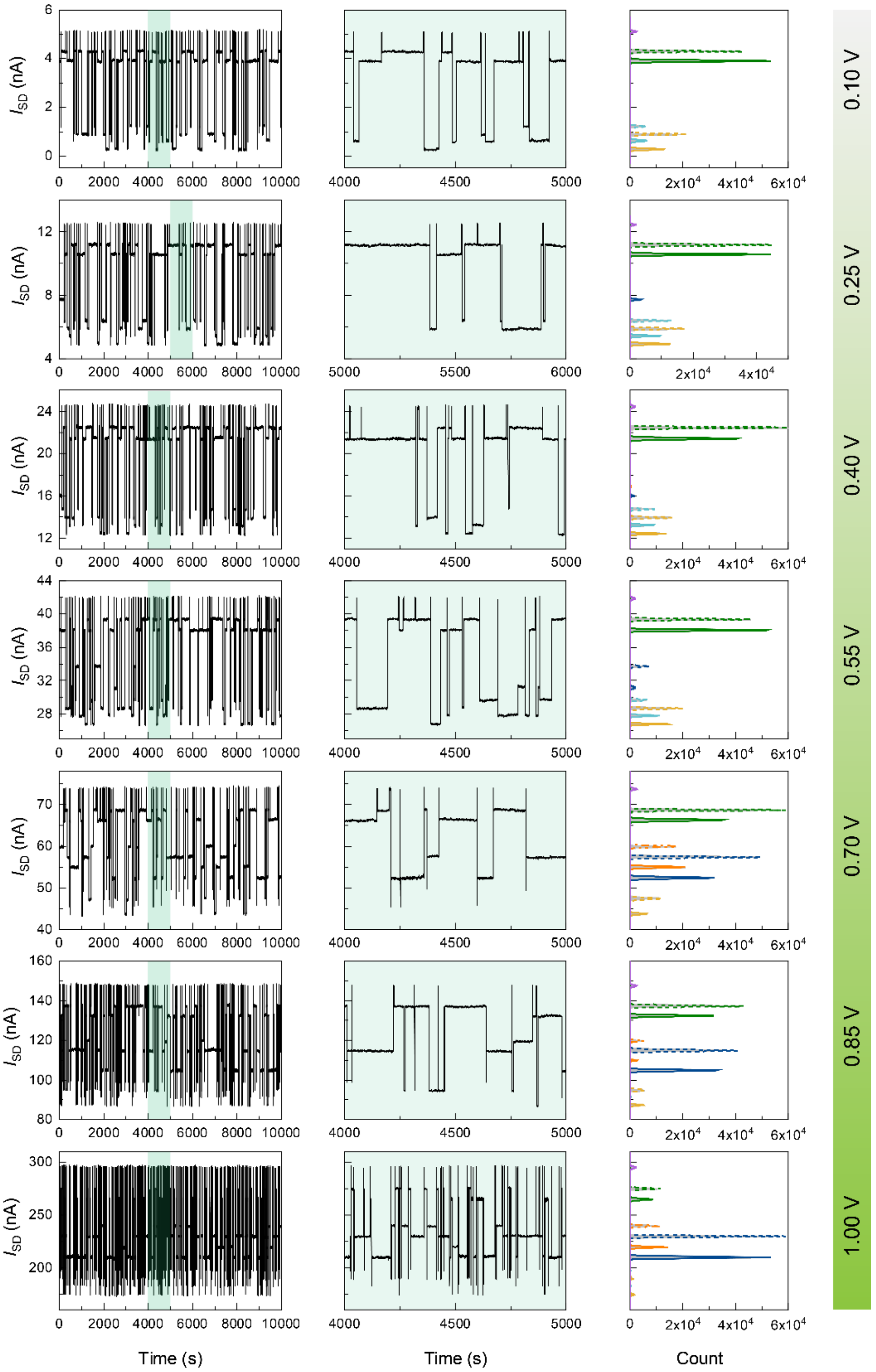

Fig. S49. The positive bias voltage dependent measurements by the C^{+} molecular bridge and a +2 T magnetized Ni electrode. The Diels-Alder cycloaddition between acrylic acid (1 mM) and isoprene (1 mM) in trifluoroacetate was monitored at 100 K. The occupancy of *S*-*m*-PS, *S*-*p*-PS, *R*-*m*-PS, and *R*-*p*-PS increased with higher positive biases, showing the driving effect on the reaction by the EEF.

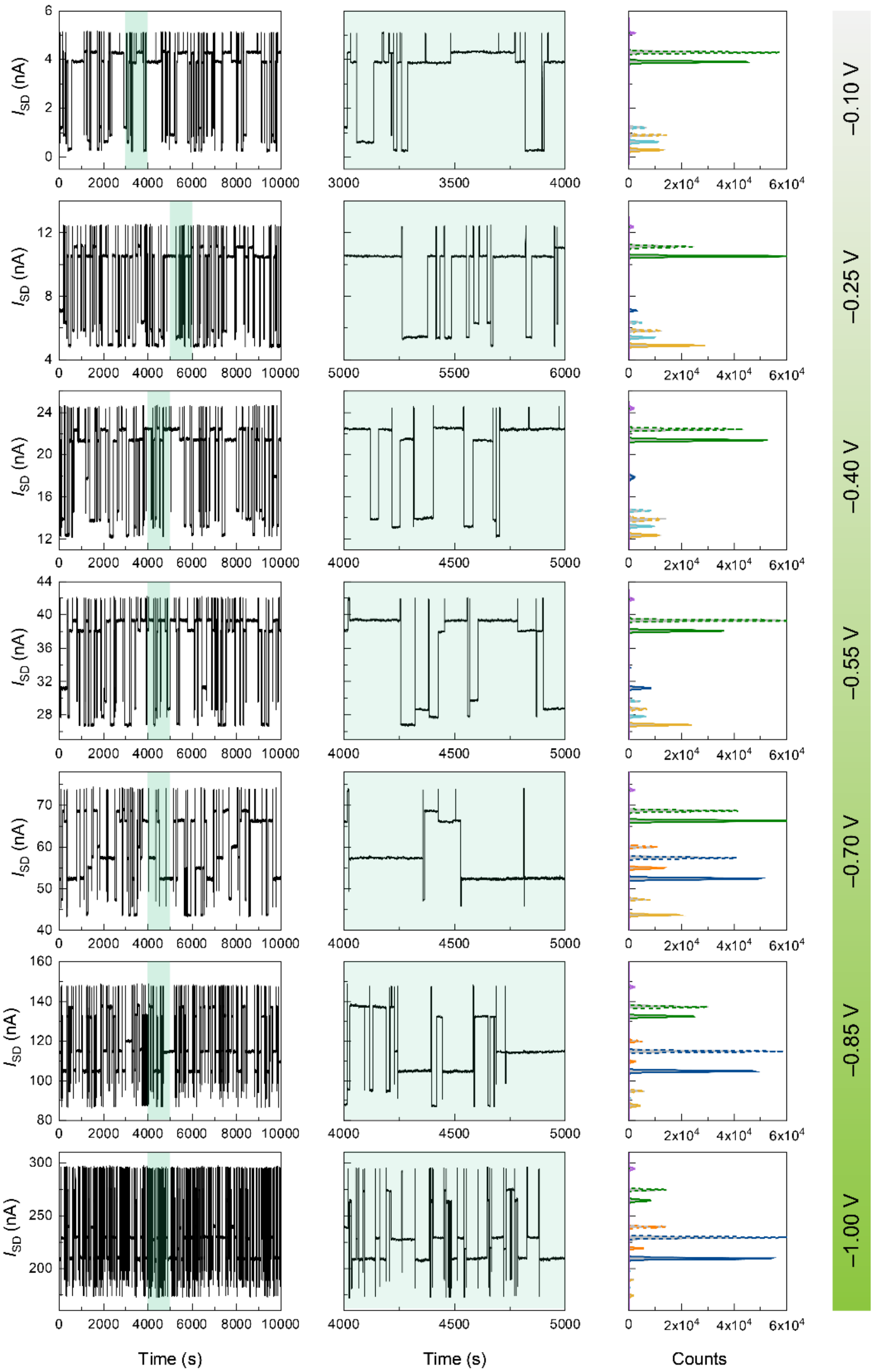

Fig. S50. The negative bias voltage dependent measurements by the C^{+} **molecular bridge and a +2 T magnetized Ni electrode.** The Diels-Alder cycloaddition between acrylic acid (1 mM) and isoprene (1 mM) in trifluoroacetate was monitored at 100 K. The occupancy of *S*-*m*-PS, *S*-*p*-PS, *R*-*m*-PS, and *R*-*p*-PS increased with higher positive biases, showing the driving effect on the reaction by the EEF.

11. The evolution of the reaction at 110 K

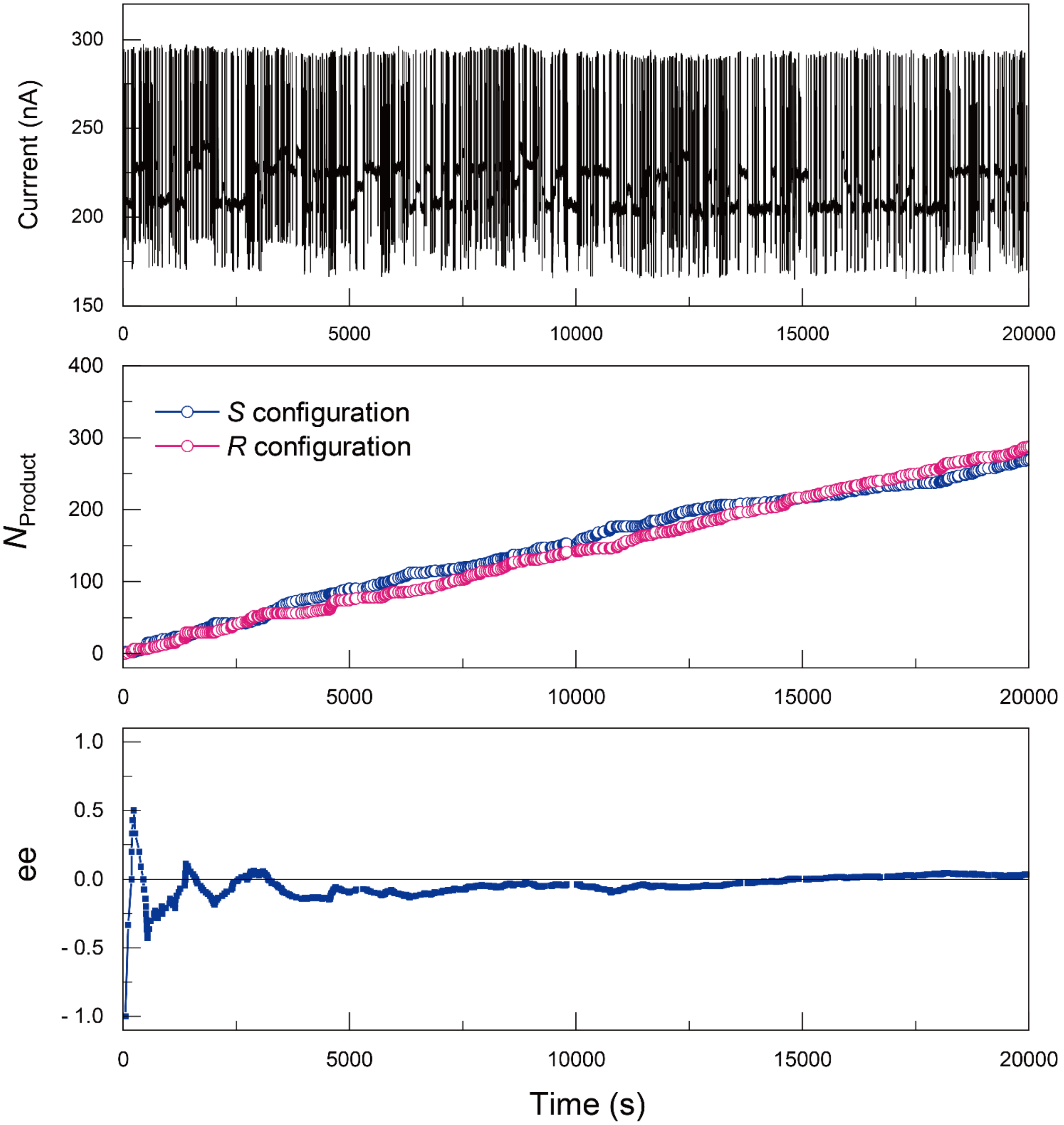

Fig. S51. The evolution of the reaction at 110 K. Top panel: *I*–*t* curves of the Diels-Alder reaction at 110 K. Middle panel: plot of cumulative chiral product number versus time. Bottom panel: The evolution trajectory indicates that the reactions tend to racemize at 110 K.

12. The assignments of the conductance states in *Z* & *E* paths

The only addition of maleic (*Z*) acid or fumaric (*E*) acid provided the assignments of the IS states, and the only addition of *E* and *Z* products also provided the assignments of the corresponding PS states. The detection of the conductance of the *Z*- (*E*-) PS with the initial addition of *E*- (*Z*-) acid showed the crossover of the two reaction pathways via zwitterionic intermediates, which was in line with our previous report[2].

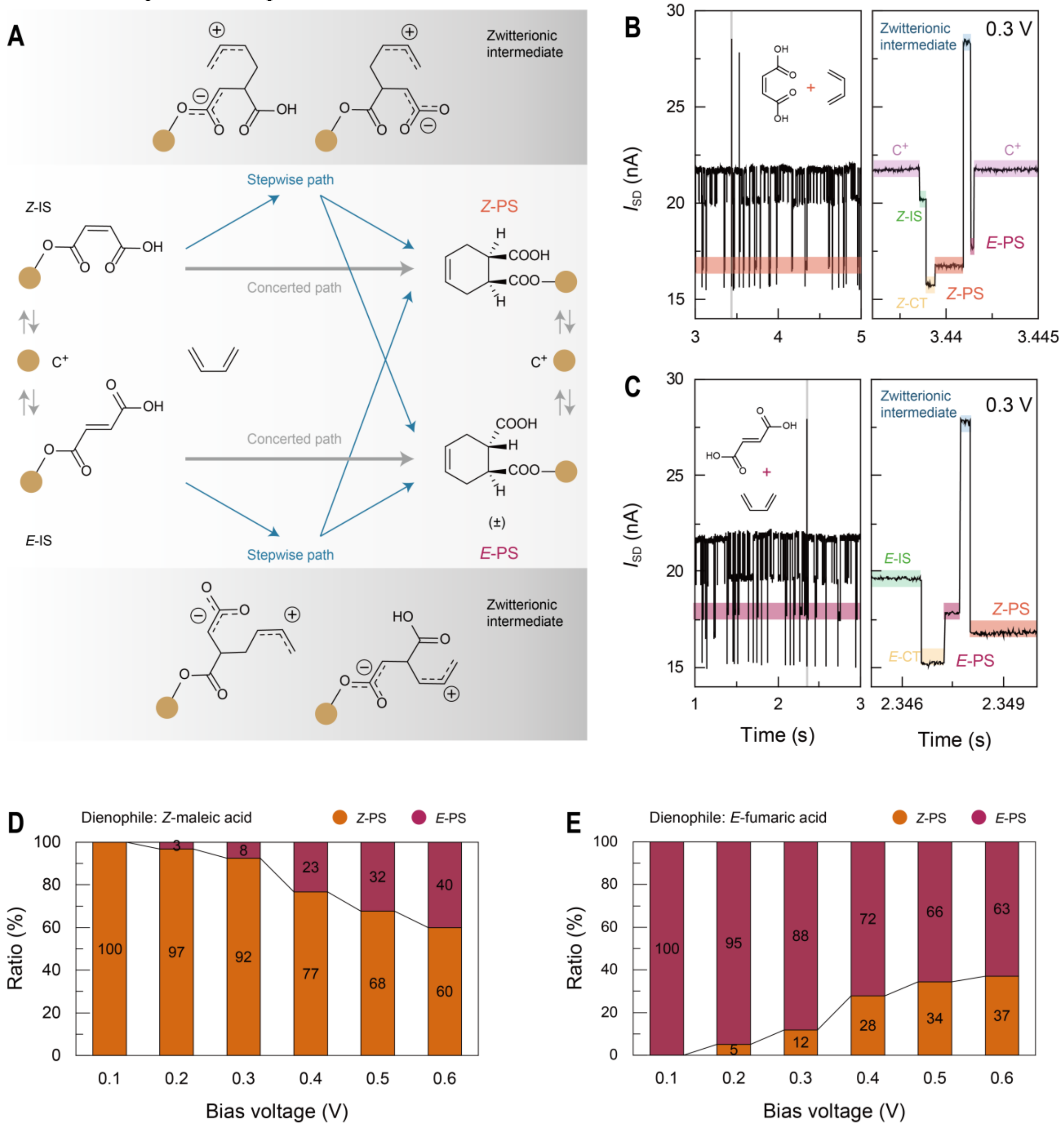

Fig. S52. Mechanistic crossover between the *Z*- and *E*-paths. A. Detected species and corresponding conversion relationship in the reaction scenarios of the concerted and stepwise paths, using fumaric acid or maleic acid as a dienophile and butadiene as a diene. **B.** *I*–*t* curves, enlarged image, and corresponding assignment of the reaction between maleic acid and diene at 393 K and 0.3 V. **C.** *I*–*t* curves, enlarged image and corresponding assignment of the reaction between fumaric

acid and diene at 393 K and 0.3 V. **D.** Statistical results of the reaction between maleic acid and diene at 393 K from 0.1 to 0.6 V with an interval of 0.1 V. **E.** Statistical results of the reaction between fumaric acid and diene at 393 K from 0.1 to 0.6 V with an interval of 0.1 V.

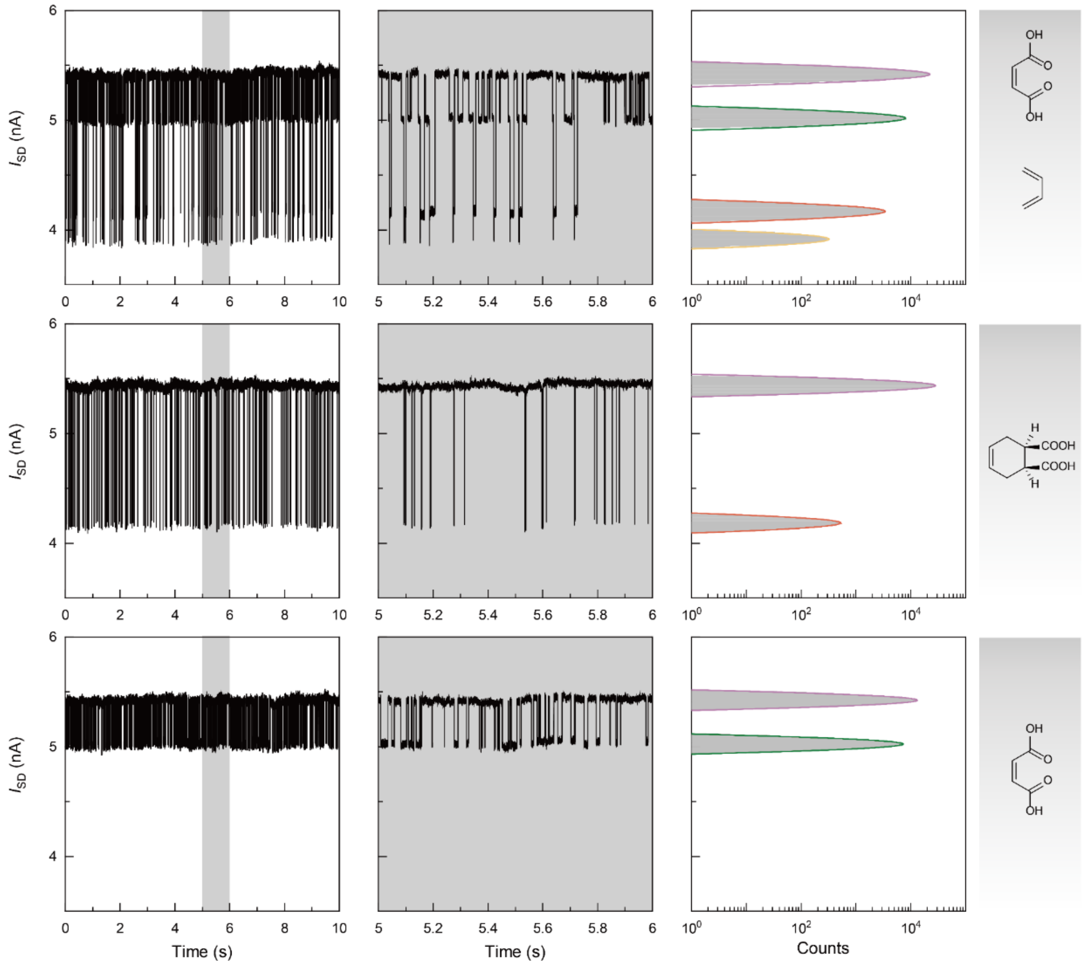

Fig. S53. Control experiments of the reaction between maleic acid and butadiene in trifluoromethanesulfonic acid. By comparing *I*–*t* curves at 0.1 V and 393 K with the addition of only *Z*-PSs (1 mM, middle panel) and maleic acid (1 mM, bottom panel), the IS and *Z*-PS can be assigned in reaction signals (top panel).

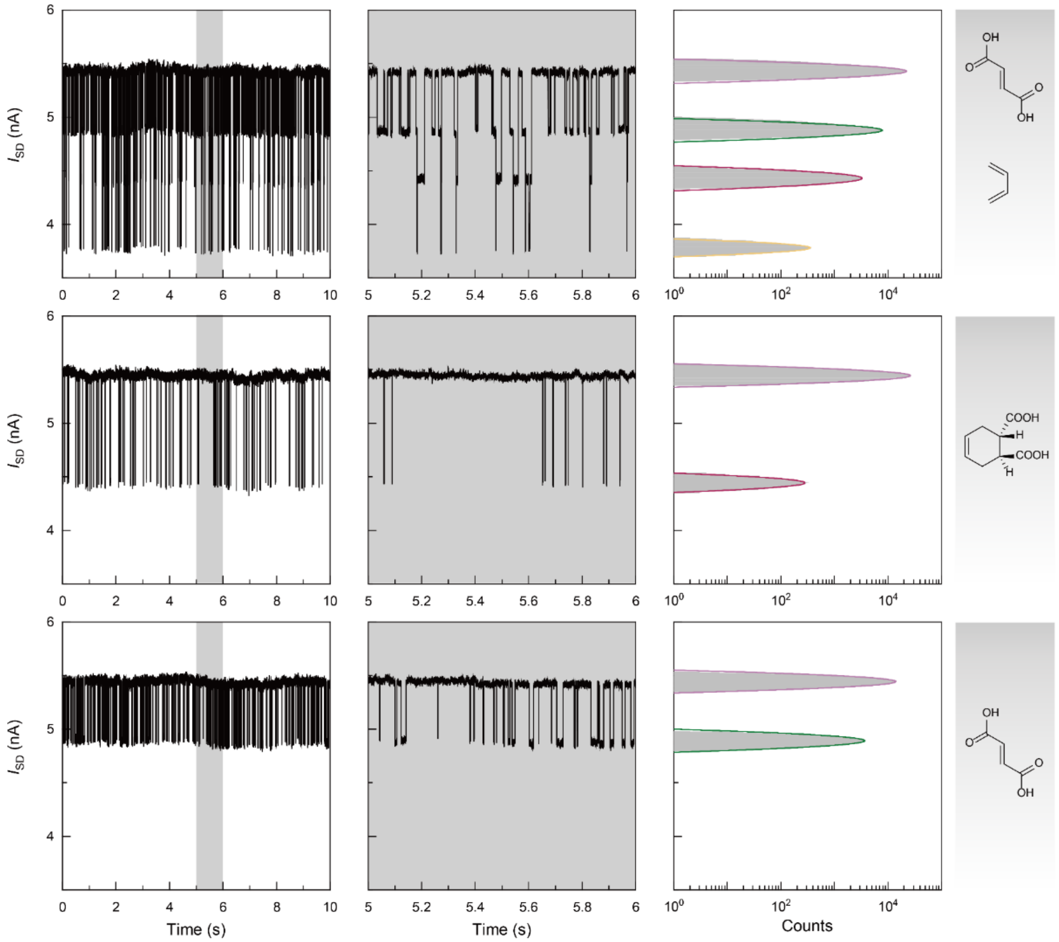

Fig. S54. Control experiments of the reaction between fumaric acid and butadiene in trifluoromethanesulfonic acid. By comparing *I*–*t* curves at 0.1 V and 393 K with the addition of only *E*-PSs (1 mM, middle panel) and maleic acid (1 mM, bottom panel), the IS and *E*-PS can be assigned in reaction signals (top panel).

13. Bias voltage-dependent measurements of the reaction including *Z* & *E* paths

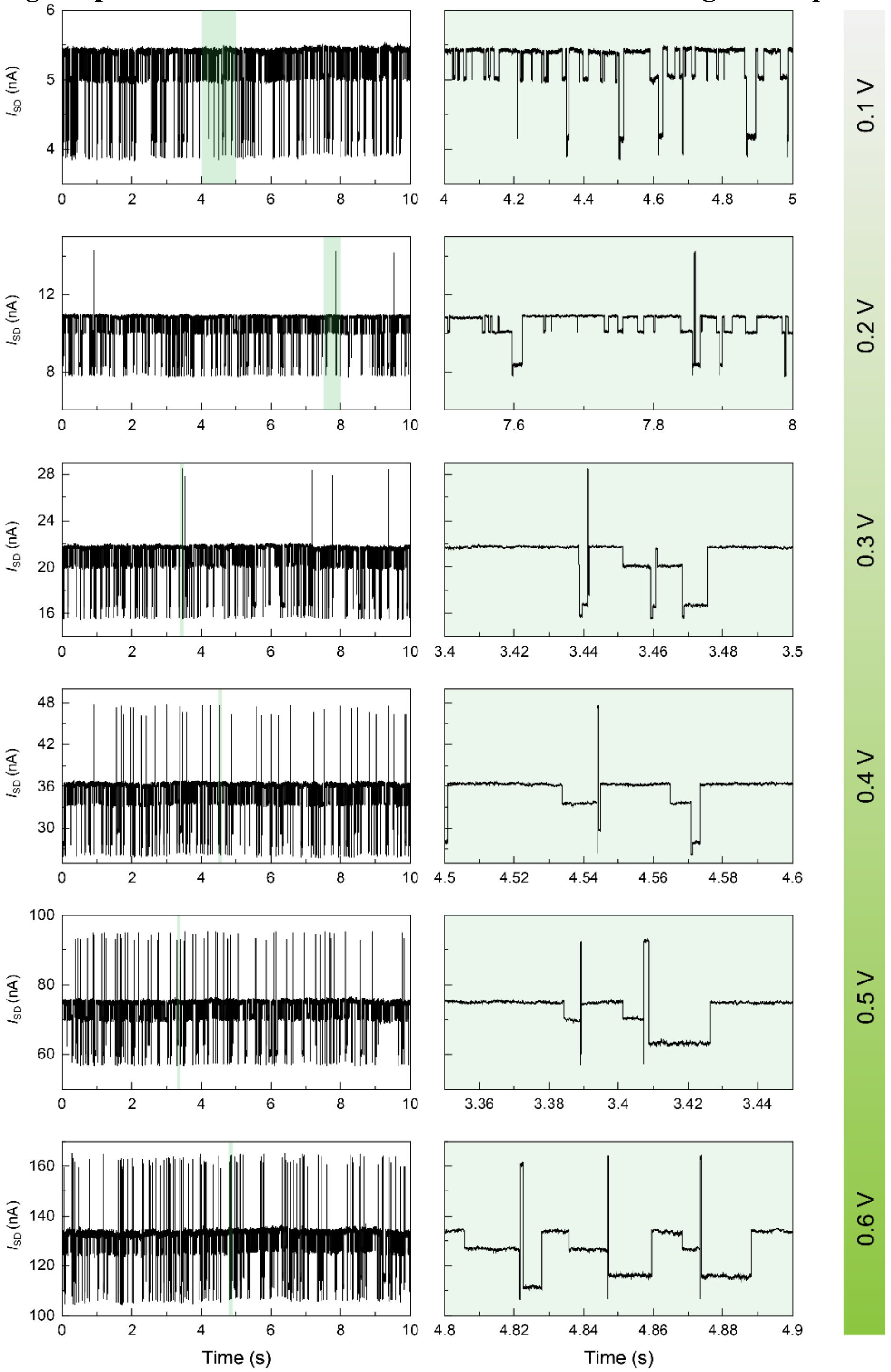

Fig. S55. The bias voltage dependent measurements of the reaction between maleic acid and butadiene by the C$^+$ molecular bridge. The Diels-Alder cycloaddition between maleic acid (1 mM) in trifluoromethanesulfonic acid and butadiene (1 atm) was monitored at 393 K. The occupancy of zwitterionic intermediates and *E*-PSs increased with higher bias voltages, showing the mechanistic crossover triggered by the EEF.

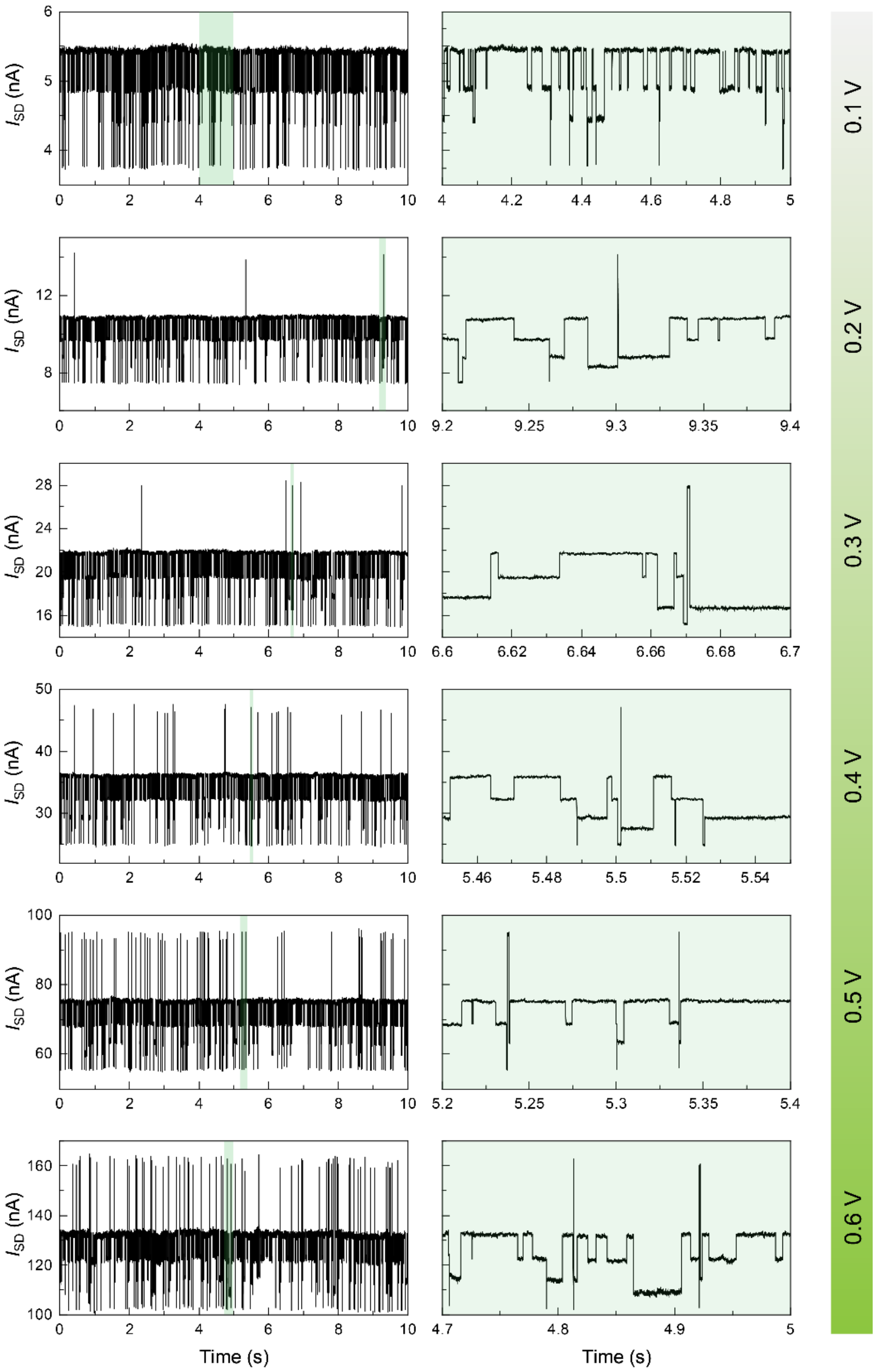

Fig. S56. The bias voltage dependent measurements of the reaction between fumaric acid and butadiene by the C^+ molecular bridge. The Diels-Alder cycloaddition between fumaric acid (1 mM) in trifluoromethanesulfonic acid and butadiene (1 atm) was monitored at 393 K. The occupancy of zwitterionic intermediates and *Z*-PSs increased with higher bias voltages, showing the mechanistic crossover triggered by the EEF.

14. The theoretical studies to control the chirality

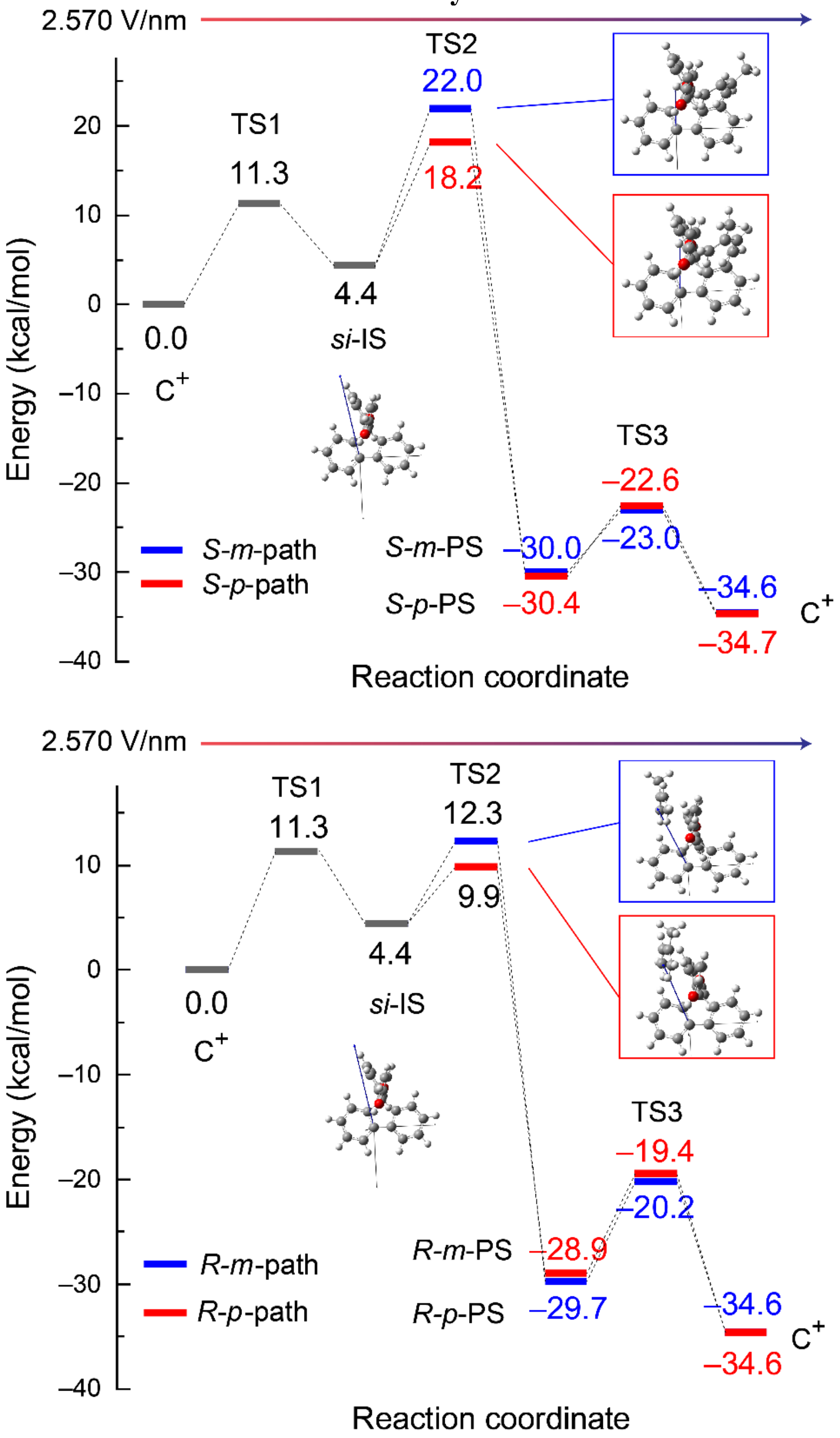

Fig. S57. The theoretical energy potential surface involving chirality with the EEF of 2.57 V/nm. For the acrylic acid located at the outside (*si*-IS), the energy potential surface indicates a large preference of the *R*-path, regardless of the regio-selectivity. The cycloaddition of isoprene from the left side will result in a larger dipole moment in the opposite direction to the EEF and further leads to a lower TS as well as the products with *R* configuration (bottom panel). Mirrored (*re*-) IS at the inside causes a flipping of the chirality preference.

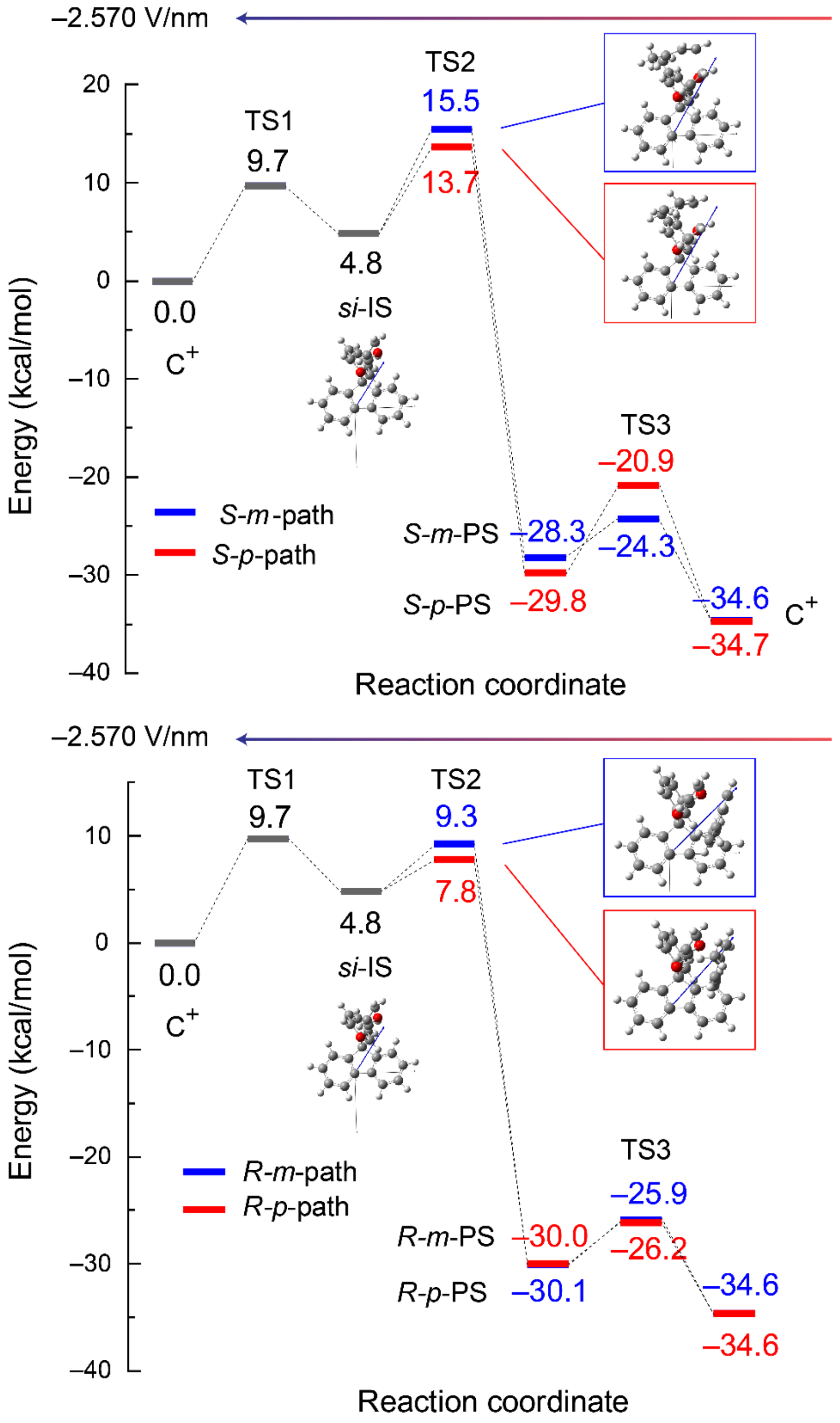

Fig. S58. The theoretical energy potential surface involving chirality with the EEF of −2.57 V/nm. For the acrylic acid located at the outside (*si*-IS), the energy potential surface indicates a large preference of the *R*-path, regardless of the regio-selectivity. The cycloaddition of isoprene from the right side will result in a larger dipole moment in the opposite direction to the EEF and further leads to a lower TS as well as the products with *R* configuration (bottom panel). Mirrored (*re*-) IS at the inside causes a flipping of the chirality preference.

15. Concept of on-line dynamic asymmetric synthesis.

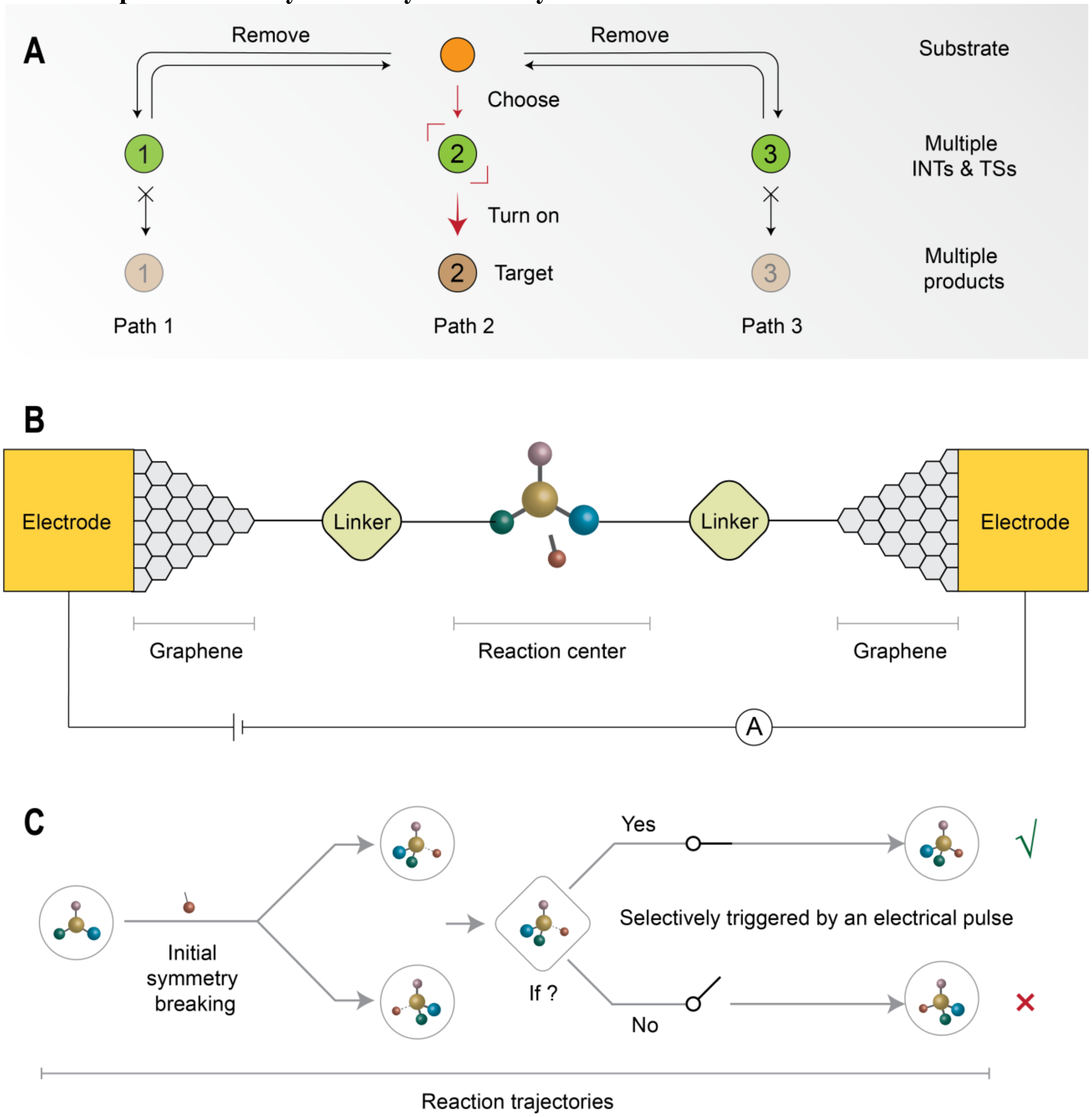

Fig. S59. Concept of on-line dynamic asymmetric synthesis. **A.** Unconventional paradigm of screening target pathways via choosing the desired intermediate or transition state and removing others. INT: intermediate; TS: transition state. **B.** Schematic of a graphene-molecule-graphene single-molecule junction (SMJ) capable of real-time monitoring the asymmetric reaction with high time resolution. **C.** Schematic of on-line controlling the asymmetric reaction based on determining whether the currently detected intermediate is the desired one. The expected reaction will be turned on by an electrical pulse (*vide infra*).

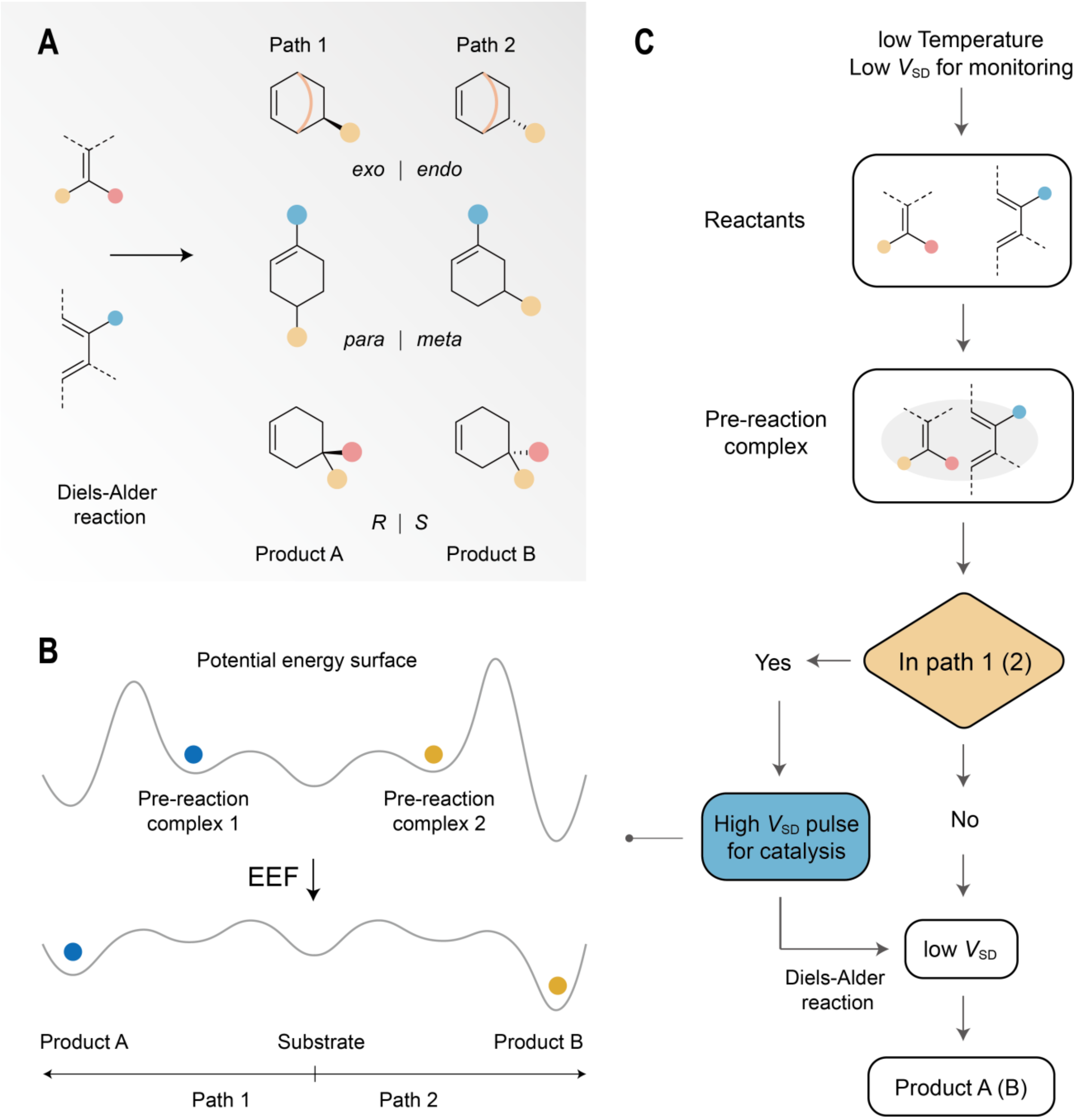

Fig. S60. Schematic demonstration of on-line precise synthesis of a Diels-Alder reaction. **A.** Three types of asymmetries in Diels–Alder cycloaddition: *endo* and *exo*, *para* and *meta*, and *R* and *S*. **B.** Schematic of the potential energy surface with the asymmetric paths in the Diels–Alder reaction. The EEF reduces the energy barrier of the reaction. **C.** Strategy for on-line control of the asymmetric Diels-Alder reaction.

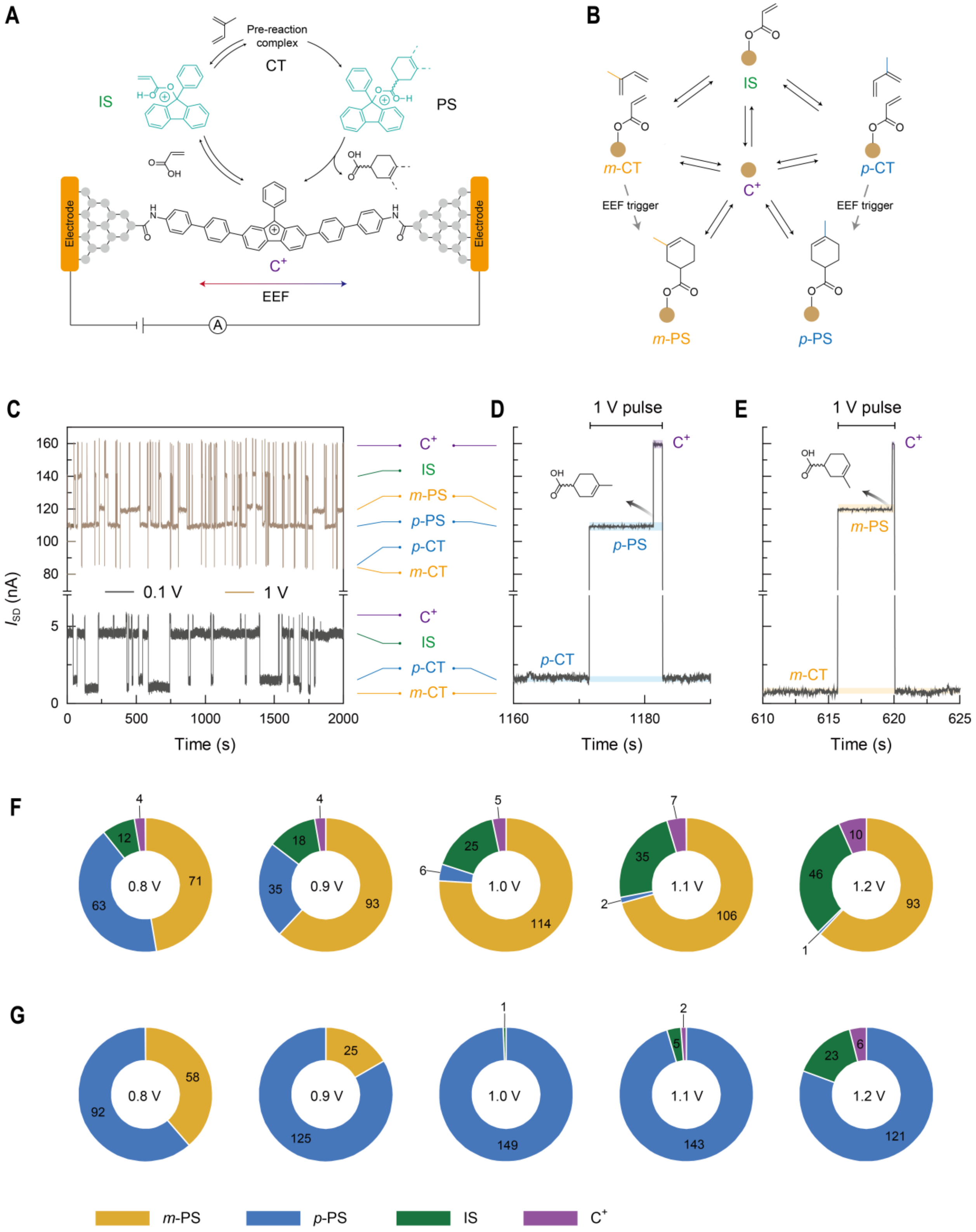

Fig. S61. On-line asymmetric synthesis of *para*- and *meta*-products. A. Schematic of a 9-phenyl-9-fluorenyl cation SMJ to monitor and regulate the reaction between acrylic acid and isoprene, involving the regio-selectivity between *para* and *meta* isomers and the stereo-selectivity

between *R* and *S* isomers. **B.** Detected species and corresponding conversion relationship in the reaction scenario: association/dissociation of the carboxyl group and C^+, and cycloaddition between acrylic acid and isoprene. **C.** Monitoring of the Diels–Alder reaction at 100 K, 0.1 V, and 1 V. **D.** *I*–*t* curves of the reaction, where a 1 V pulse was applied at the *p*-CT state and removed at the C^+ state. **e.** *I*–*t* curves of the reaction, where a 1 V pulse was applied at the *m*-CT state and removed at the C^+ state. **F.** Statistics of 150-times electric stimuli to the *m*-CT with different amplitudes to synthesize a target *m*-PS. **G.** Statistics of 150-times electric stimuli to the *p*-CT with different amplitudes to synthesize a target *p*-PS.

16. The multiple-time control of the regio-selectivity

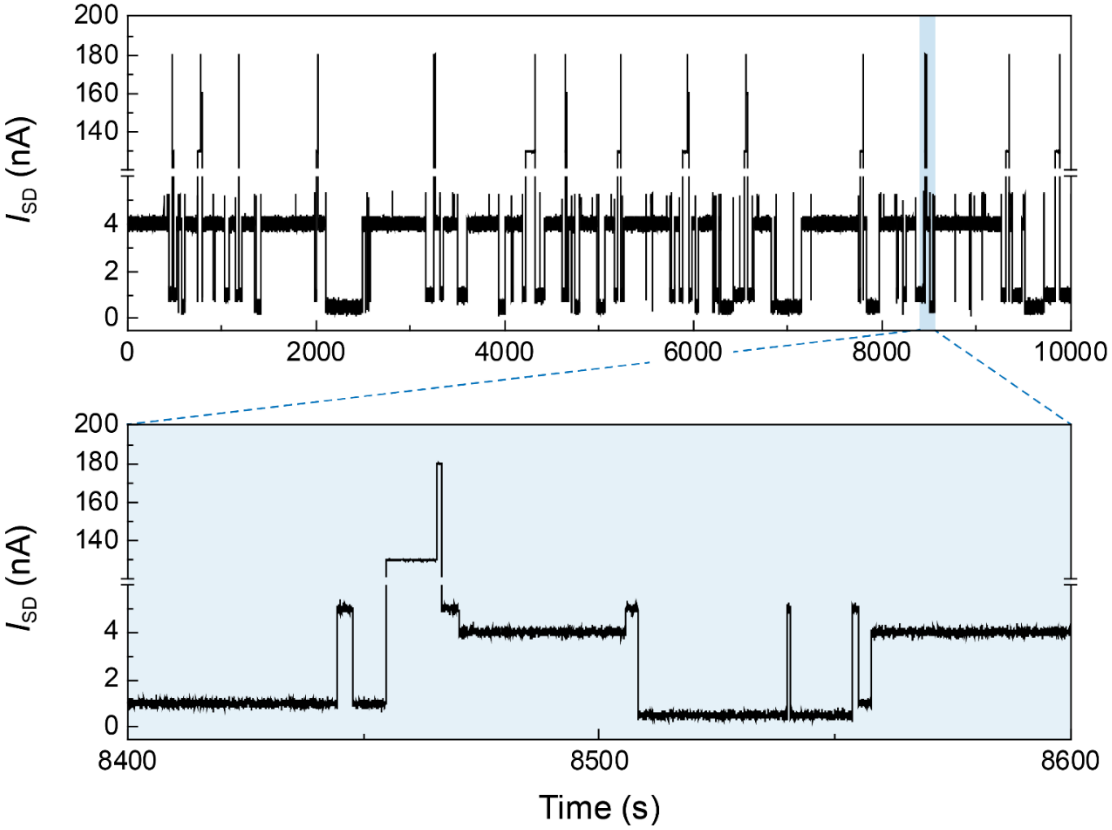

Fig. S62. Multiple-time direct synthesis of the *p*-PS. The Diels-Alder cycloaddition between acrylic acid (1 mM) and isoprene (1 mM) in trifluoroacetate was monitored at 100 K and 0.1 V bias voltage by the C^+ molecular bridge. A 1 V bias voltage was applied at the *p*-CT and removed at the C^+ state to prepare a *p*-PS. The corresponding enlarged image was provided in the bottom panel.

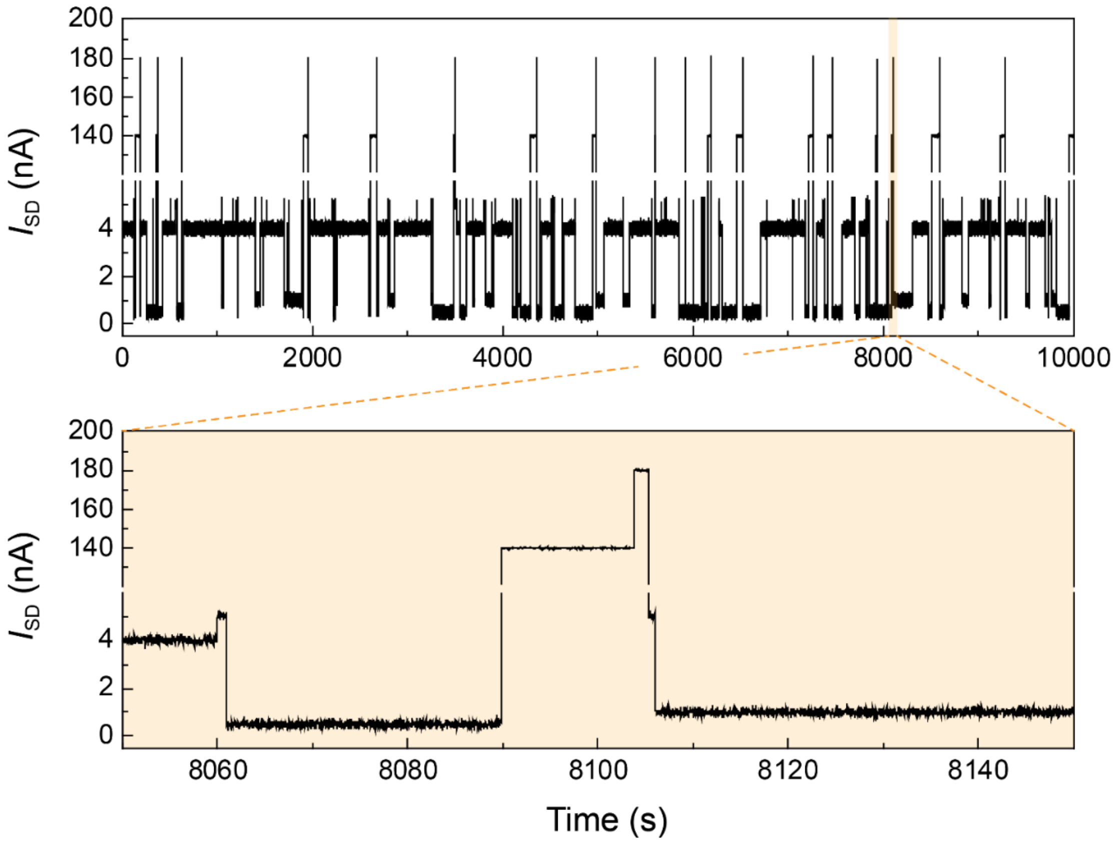

Fig. S63. Multiple-time direct synthesis of the *m*-PS. The Diels-Alder cycloaddition between acrylic acid (1 mM) and isoprene (1 mM) in trifluoroacetate was monitored at 100 K and 0.1 V bias voltage by the C^+ molecular bridge. A 1 V bias voltage was applied at the *m*-CT and removed at the C^+ state to prepare a *m*-PS. The corresponding enlarged image was provided in the bottom panel.

17. On-line operation on other 20 single-molecule devices to synthesize *p*-PS and *m*-PS at 100 K.

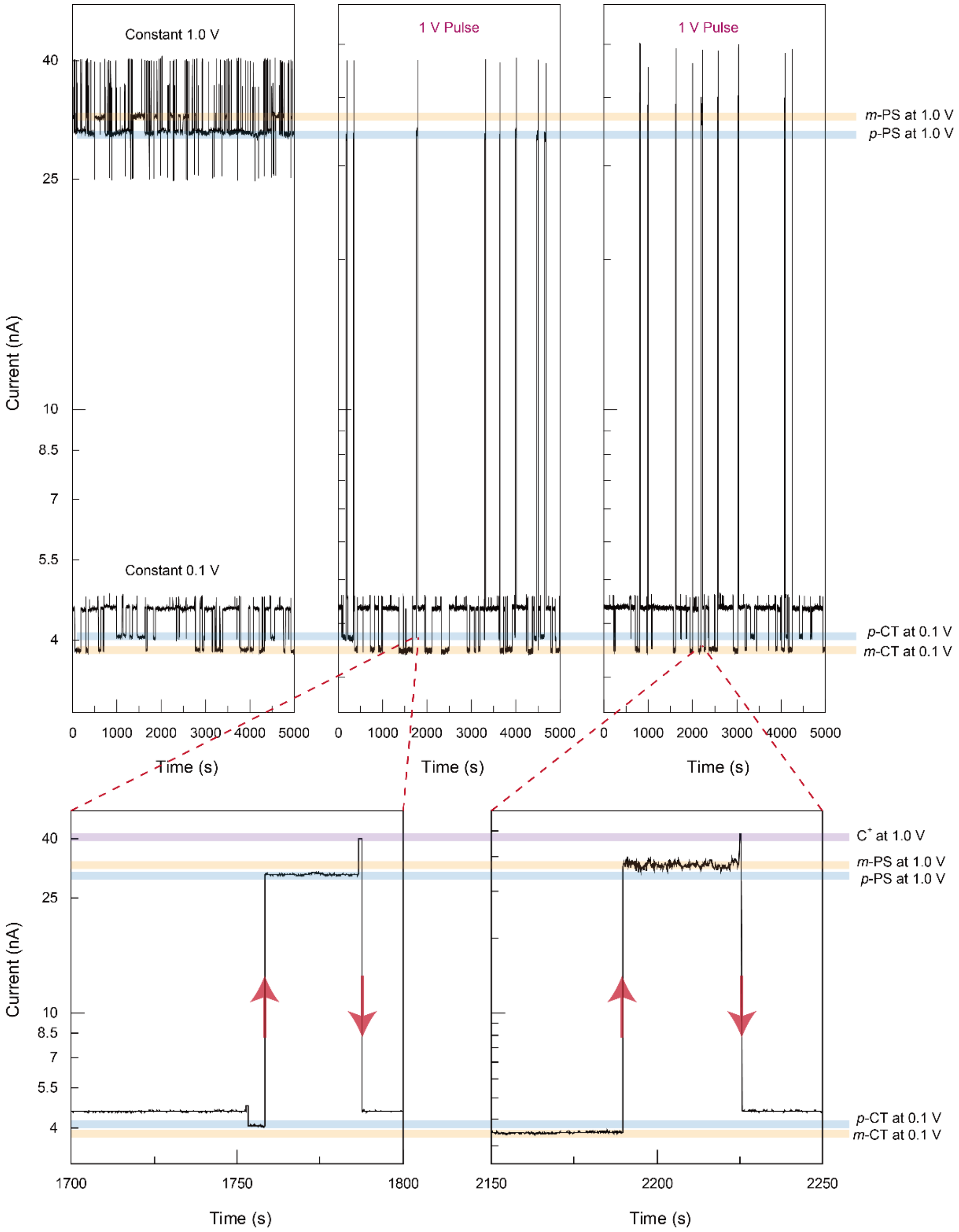

Fig. S64. Preparation of *p*-PS and *m*-PS on device 1#. The *I*–*t* measurements with constant 0.1 V and 1 V bias voltages were provided at the left panel as a reference. For the preparation of the *p*-PS (middle panel), a 1 V bias voltage was applied at the *p*-CT state measured by 0.1 V, and removed at the C^+ state measured by 1 V. For the preparation of the *m*-PS (right panel), a 1 V bias voltage was applied at the *m*-CT state measured by 0.1 V, and removed at the C^+ state measured by 1 V.

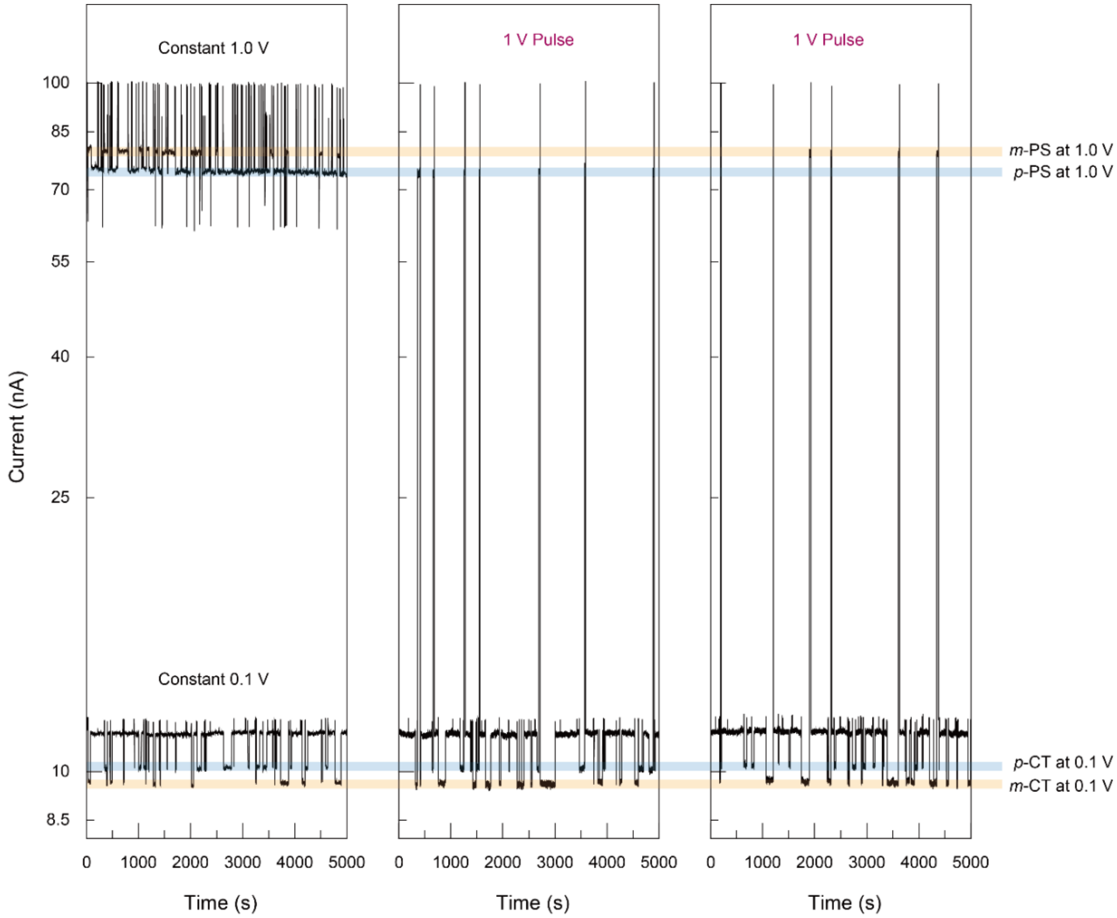

Fig. S65. Preparation of *p*-PS and *m*-PS on device 2#.

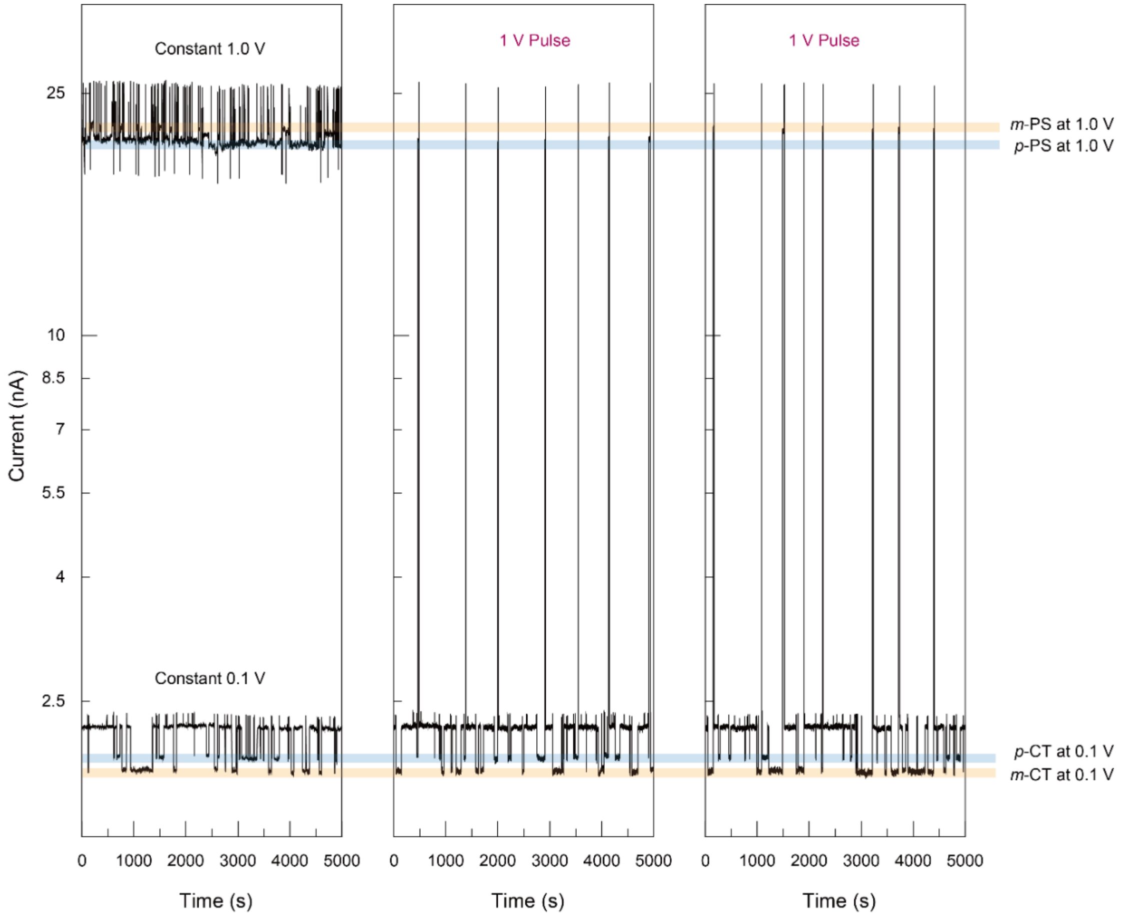

Fig. S66. Preparation of *p*-PS and *m*-PS on device 3#.

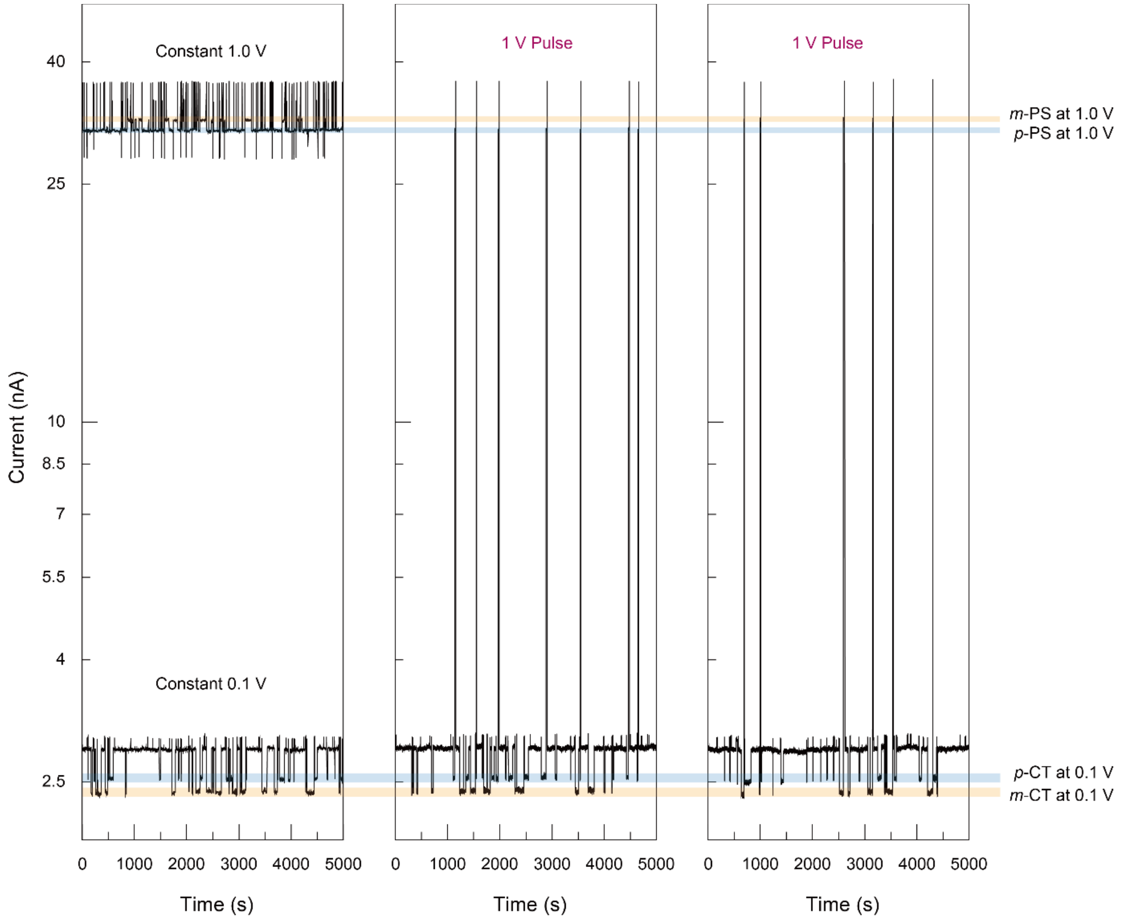

Fig. S67. Preparation of *p*-PS and *m*-PS on device 4#.

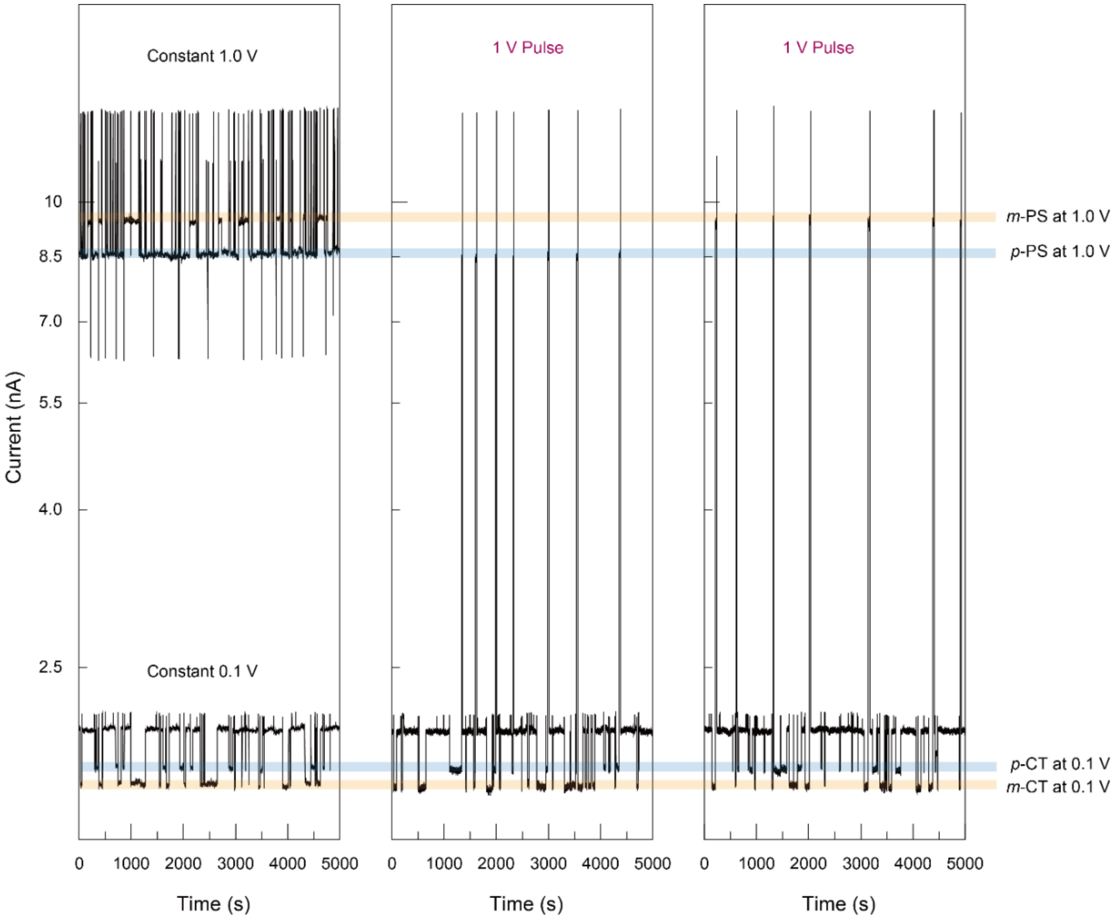

Fig. S68. Preparation of *p*-PS and *m*-PS on device 5#.

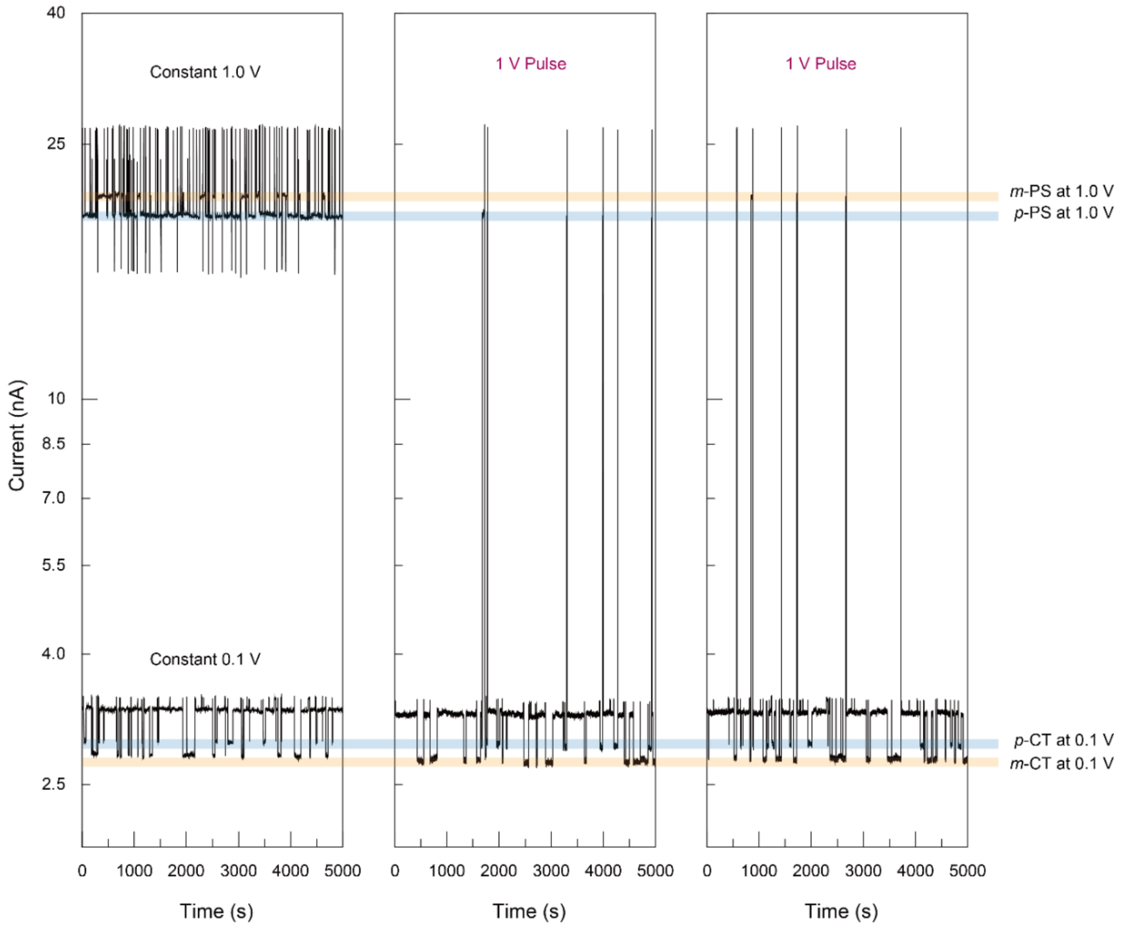

Fig. S69. Preparation of *p*-PS and *m*-PS on device 6#.

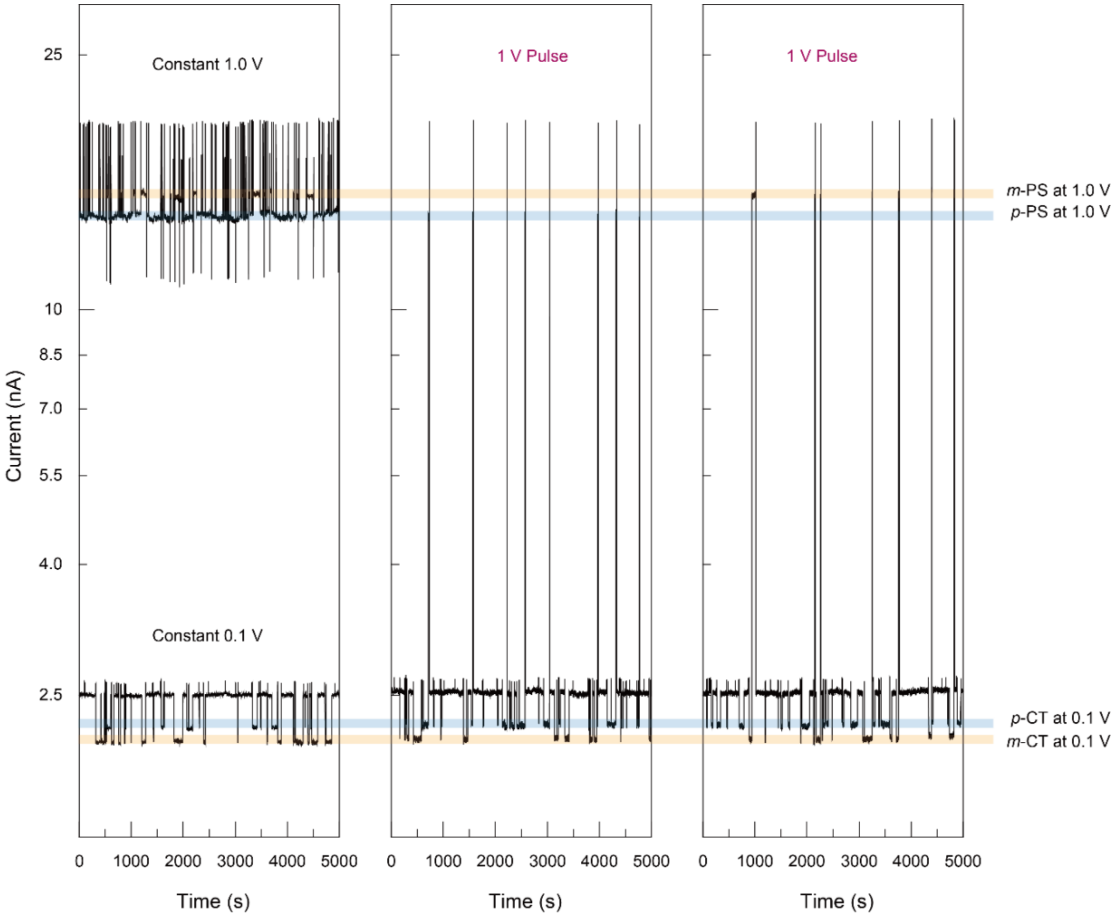

Fig. S70. Preparation of *p*-PS and *m*-PS on device 7#.

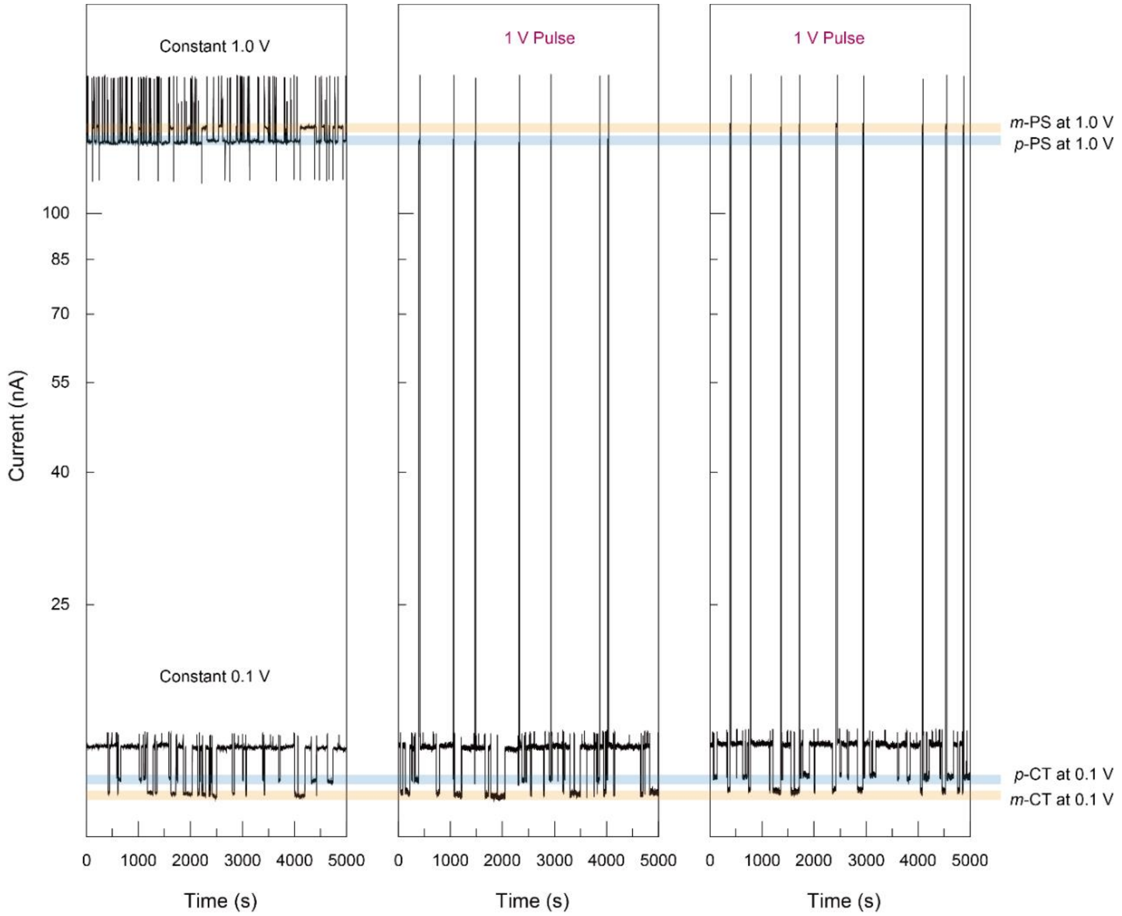

Fig. S71. Preparation of *p*-PS and *m*-PS on device 8#.

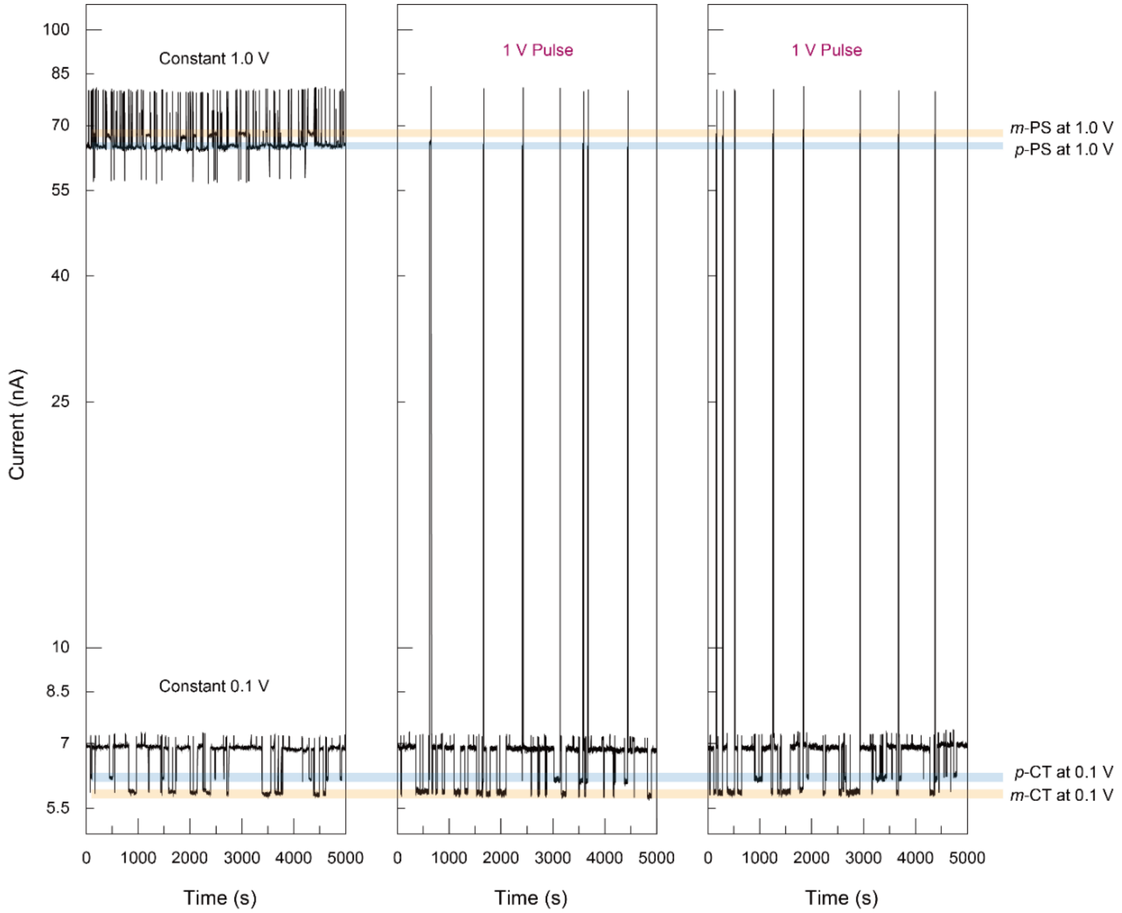

Fig. S72. Preparation of *p*-PS and *m*-PS on device 9#.

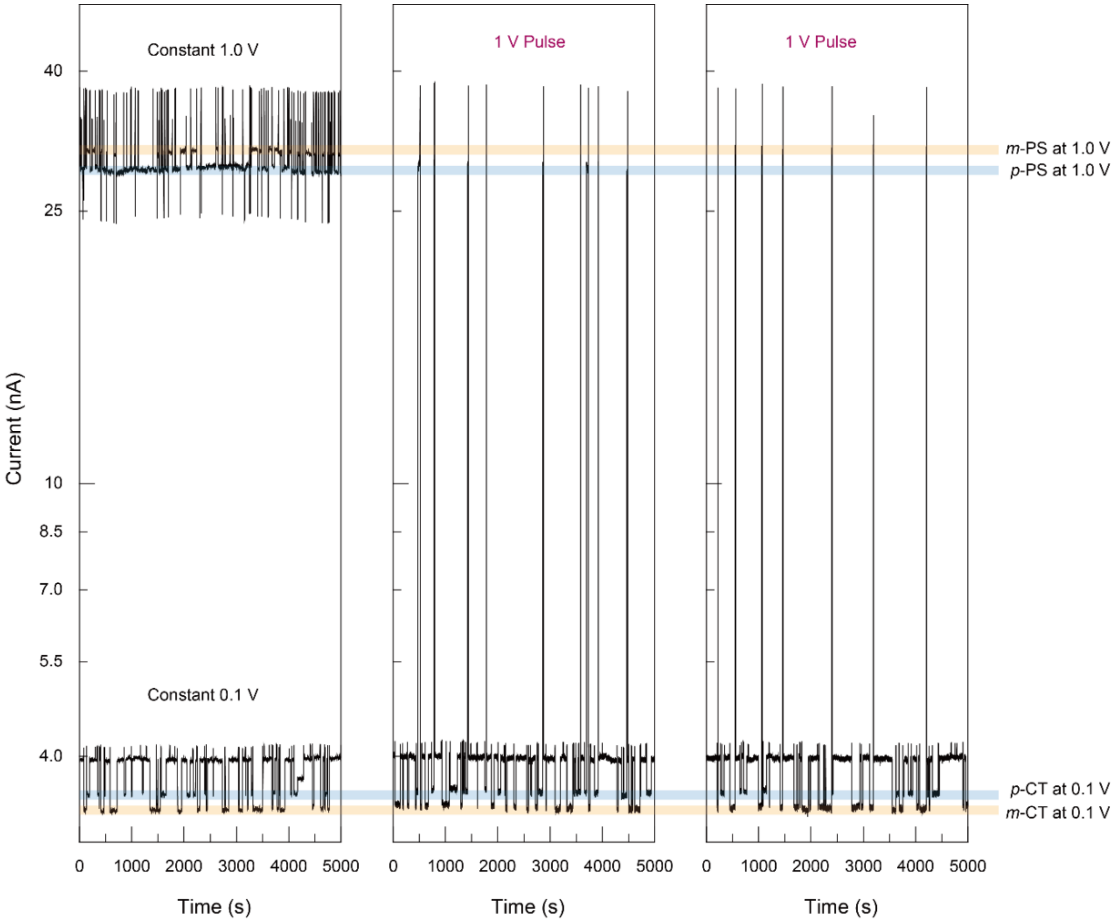

Fig. S73. Preparation of *p*-PS and *m*-PS on device 10#.

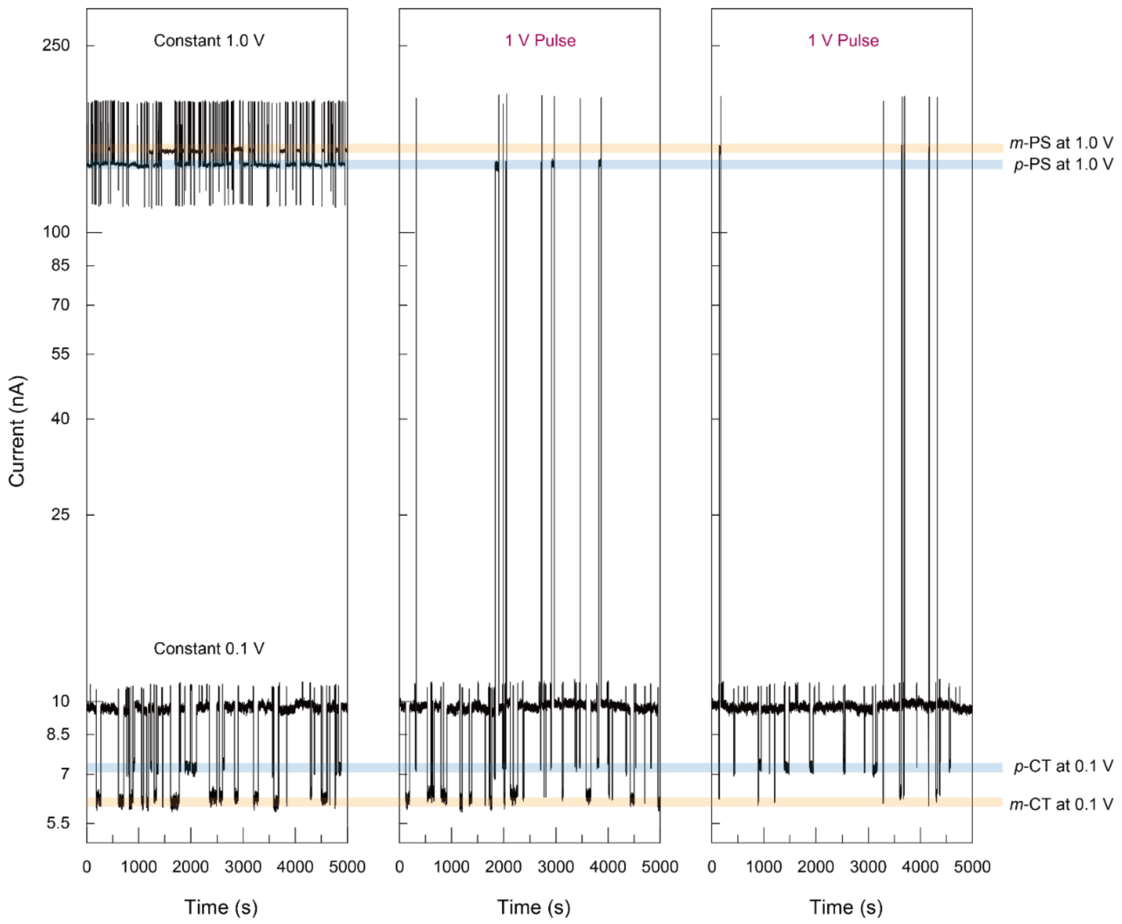

Fig. S74. Preparation of *p*-PS and *m*-PS on device 11#.

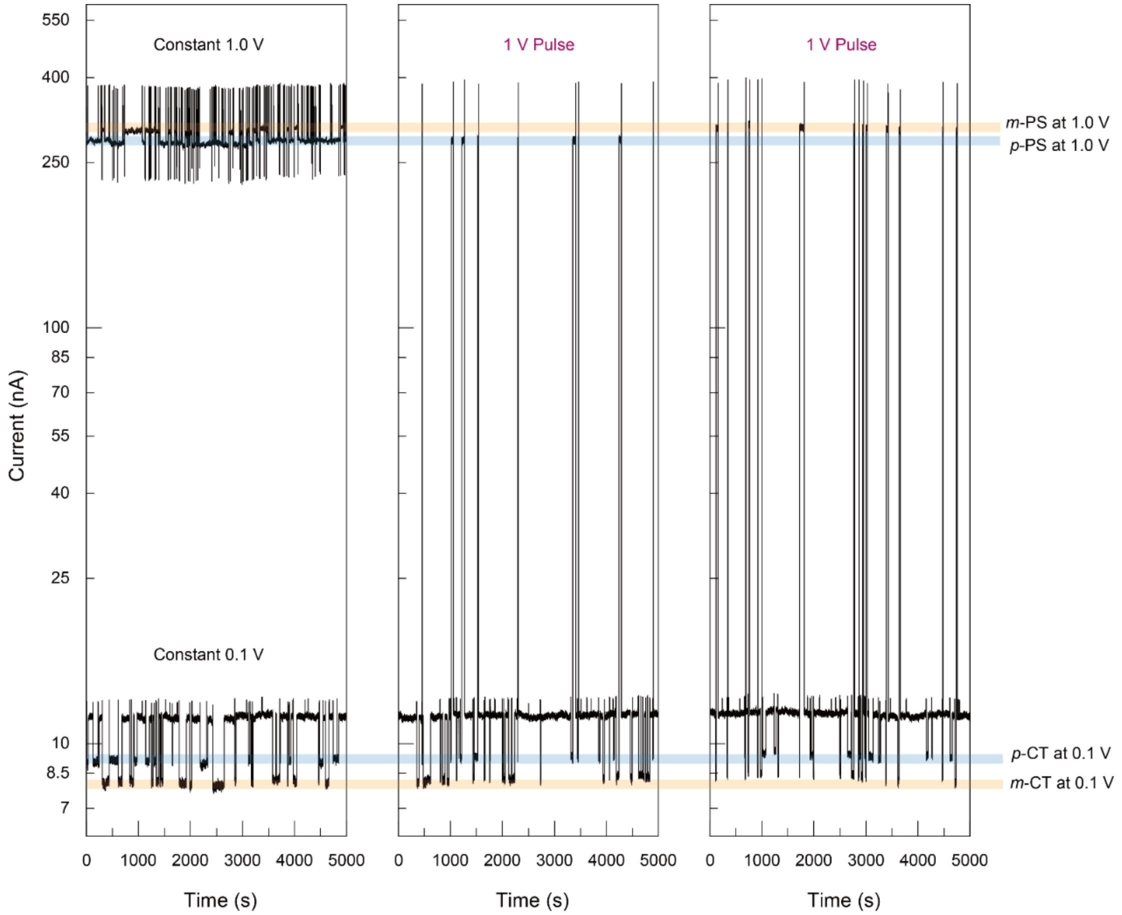

Fig. S75. Preparation of *p*-PS and *m*-PS on device 12#.

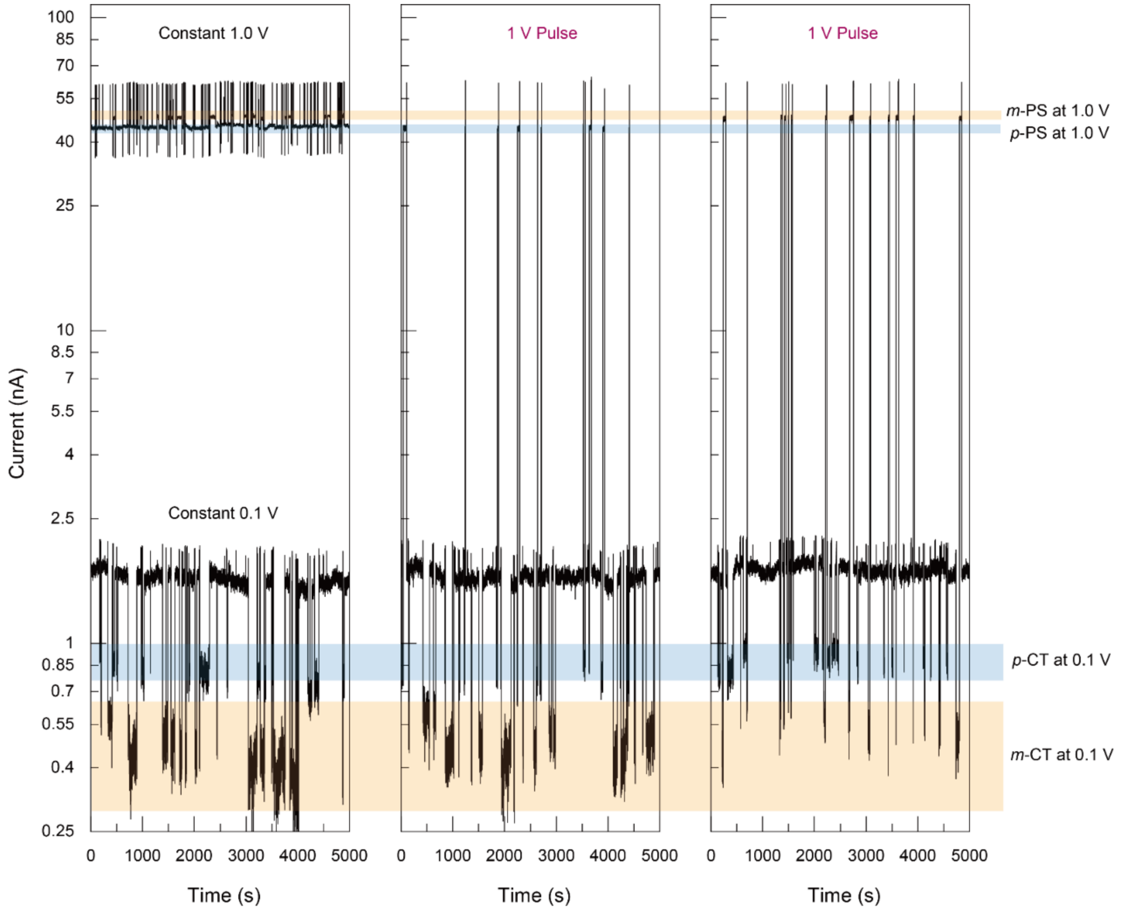

Fig. S76. Preparation of *p*-PS and *m*-PS on device 13#.

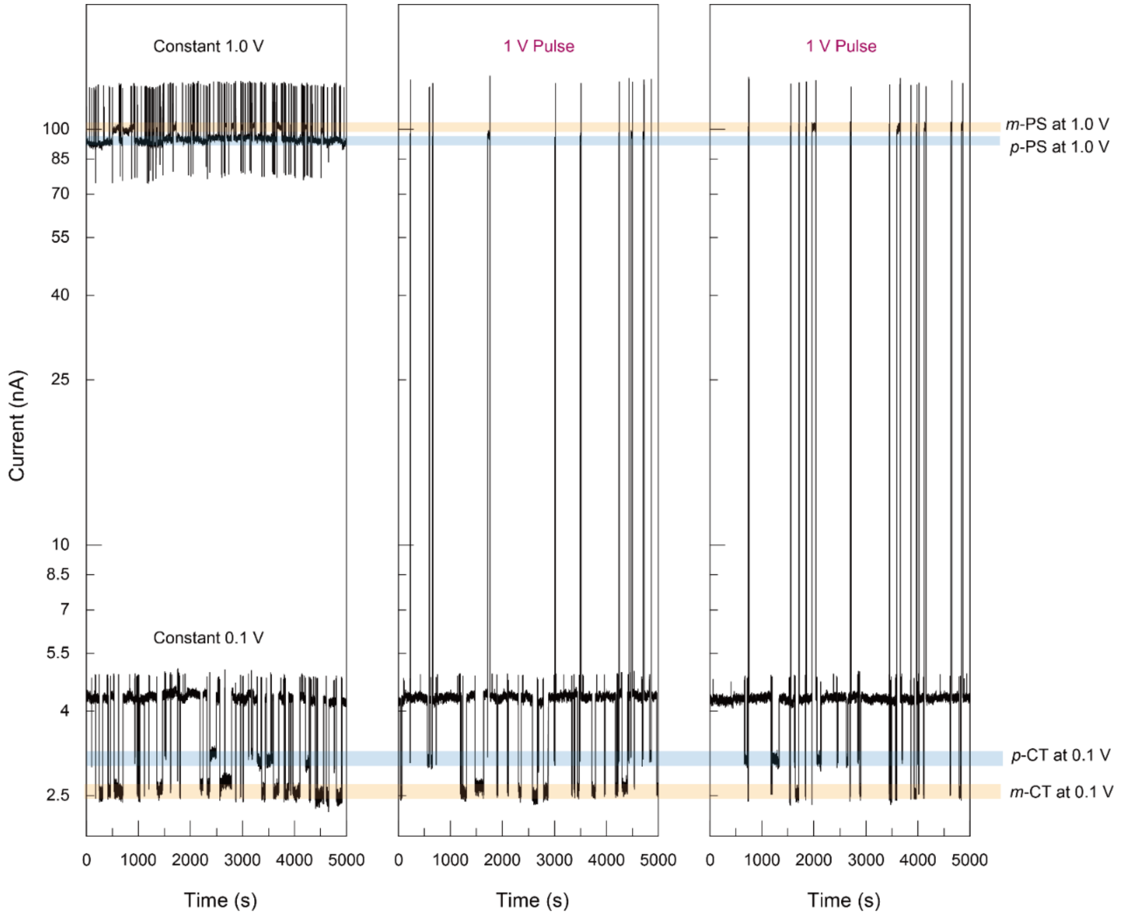

Fig. S77. Preparation of *p*-PS and *m*-PS on device 14#.

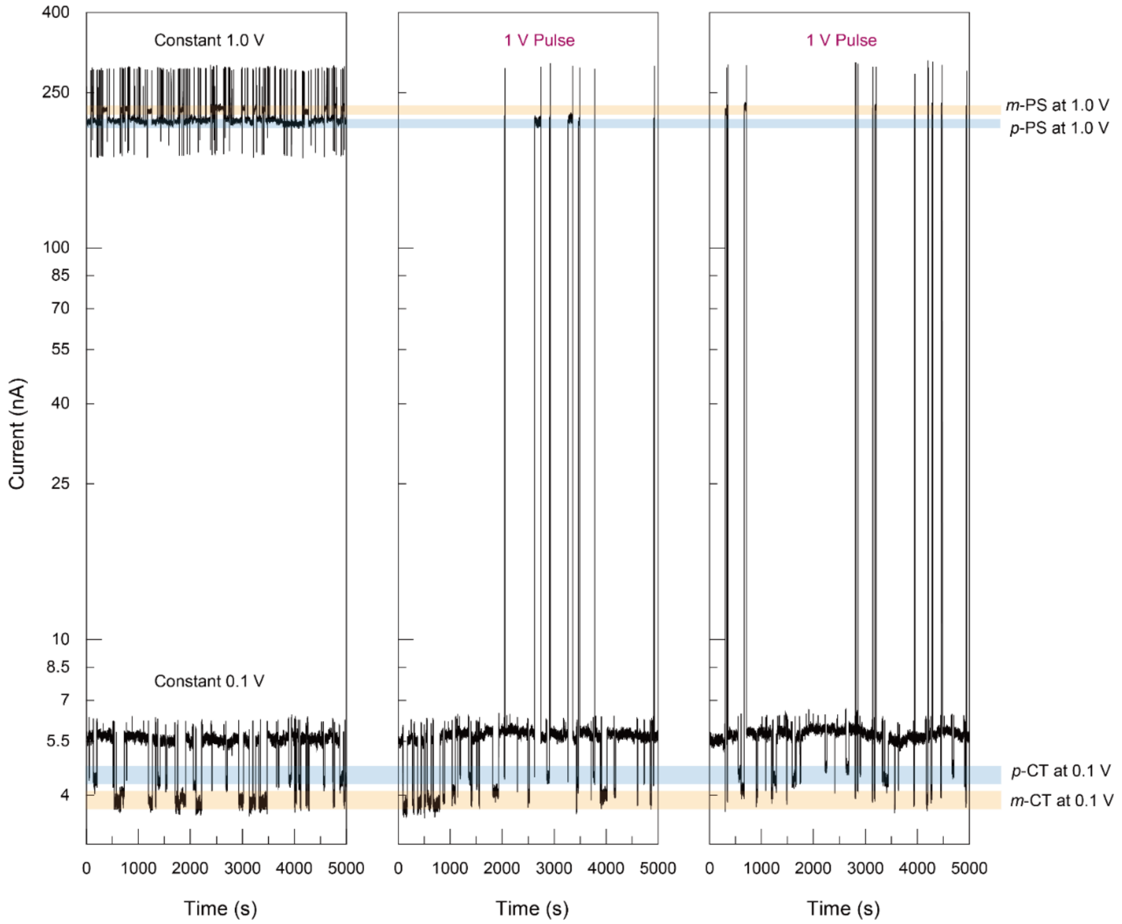

Fig. S78. Preparation of *p*-PS and *m*-PS on device 15#.

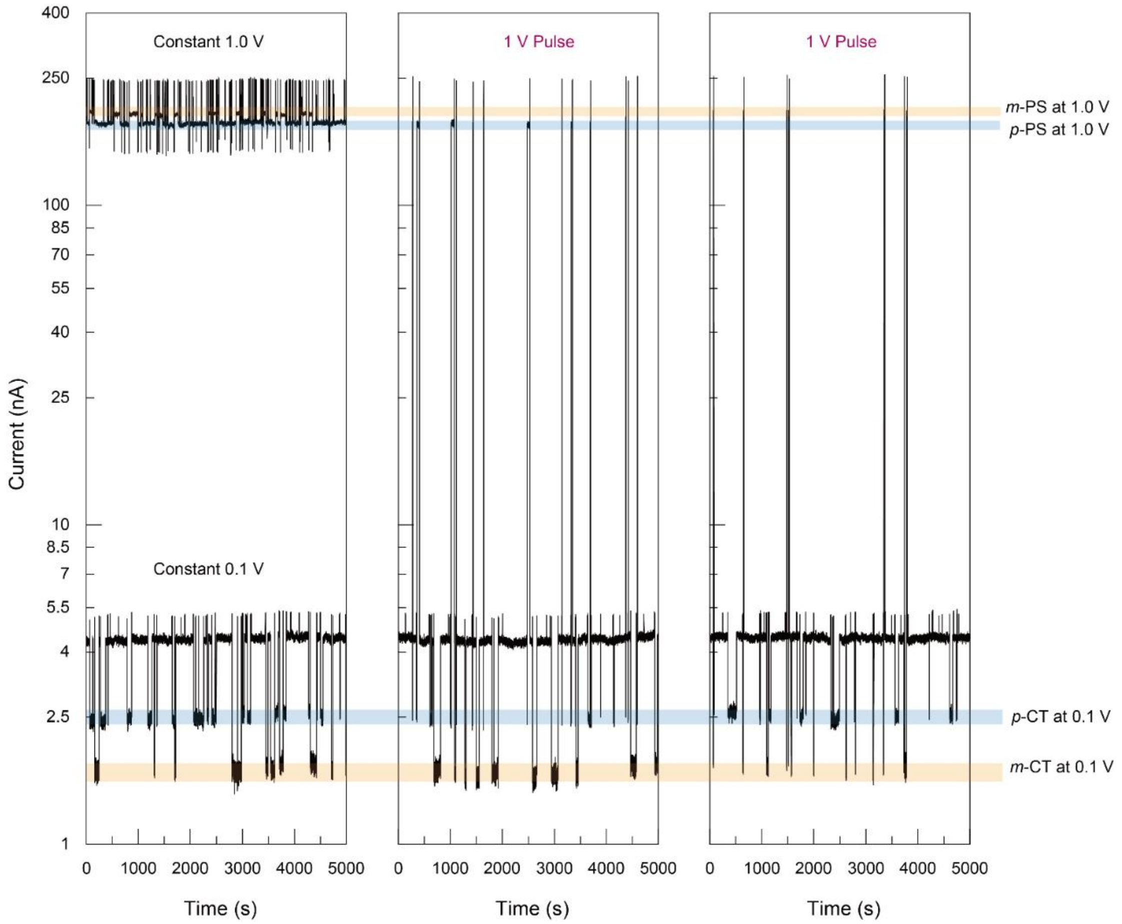

Fig. S79. Preparation of *p*-PS and *m*-PS on device 16#.

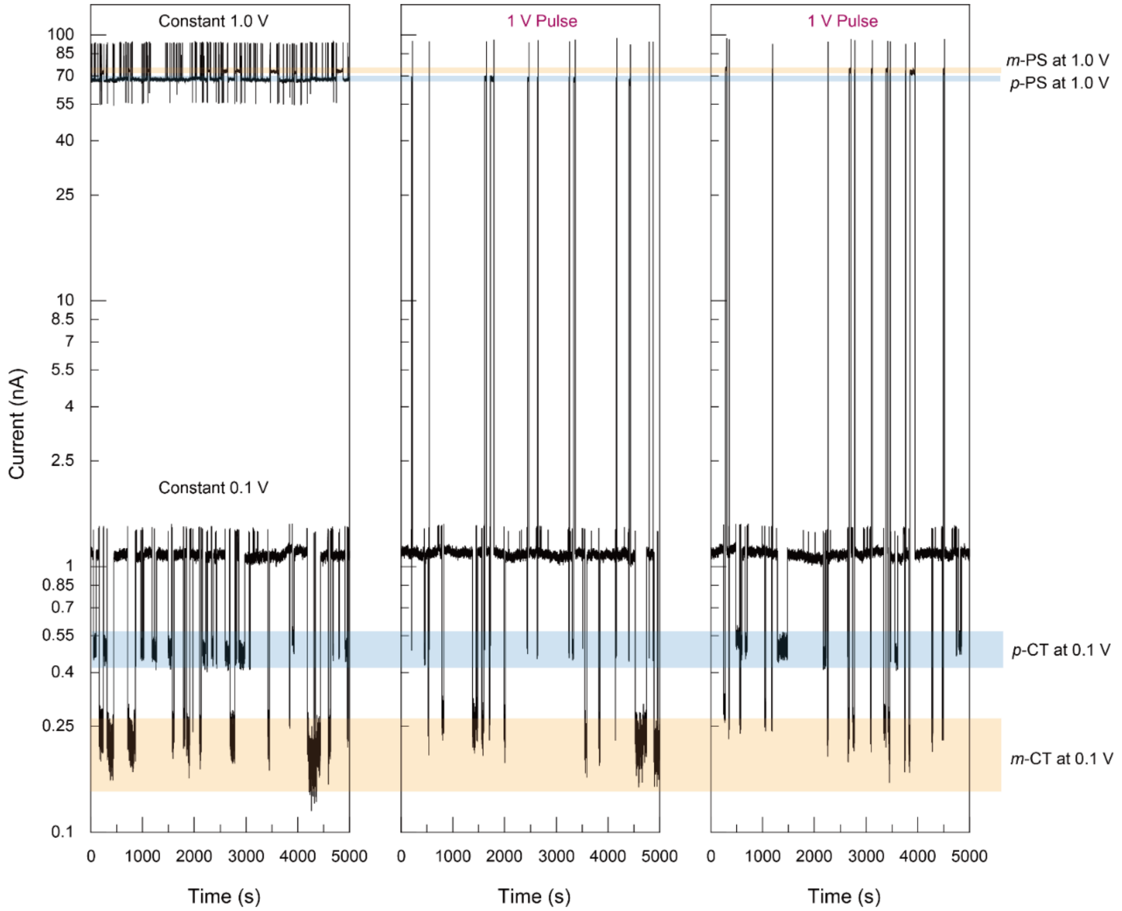

Fig. S80. Preparation of *p*-PS and *m*-PS on device 17#.

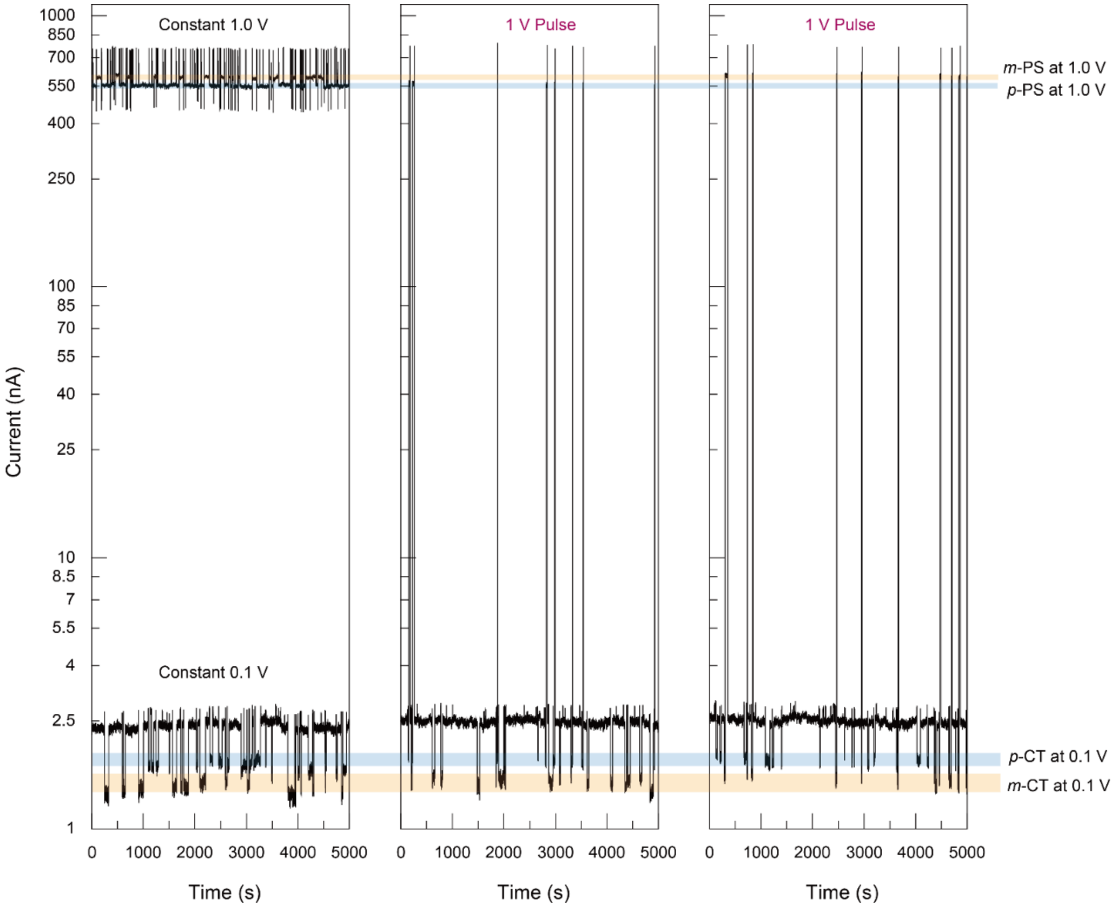

Fig. S81. Preparation of *p*-PS and *m*-PS on device 18#.

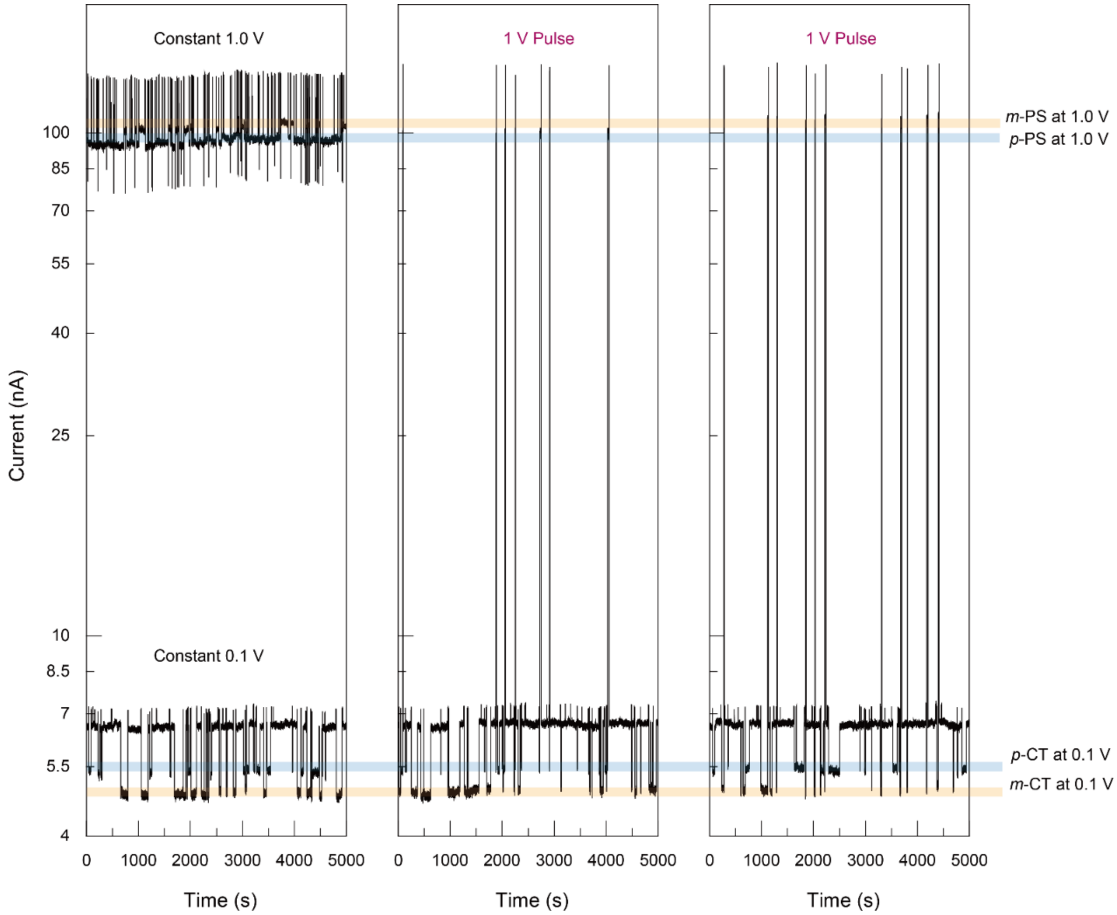

Fig. S82. Preparation of *p*-PS and *m*-PS on device 19#.

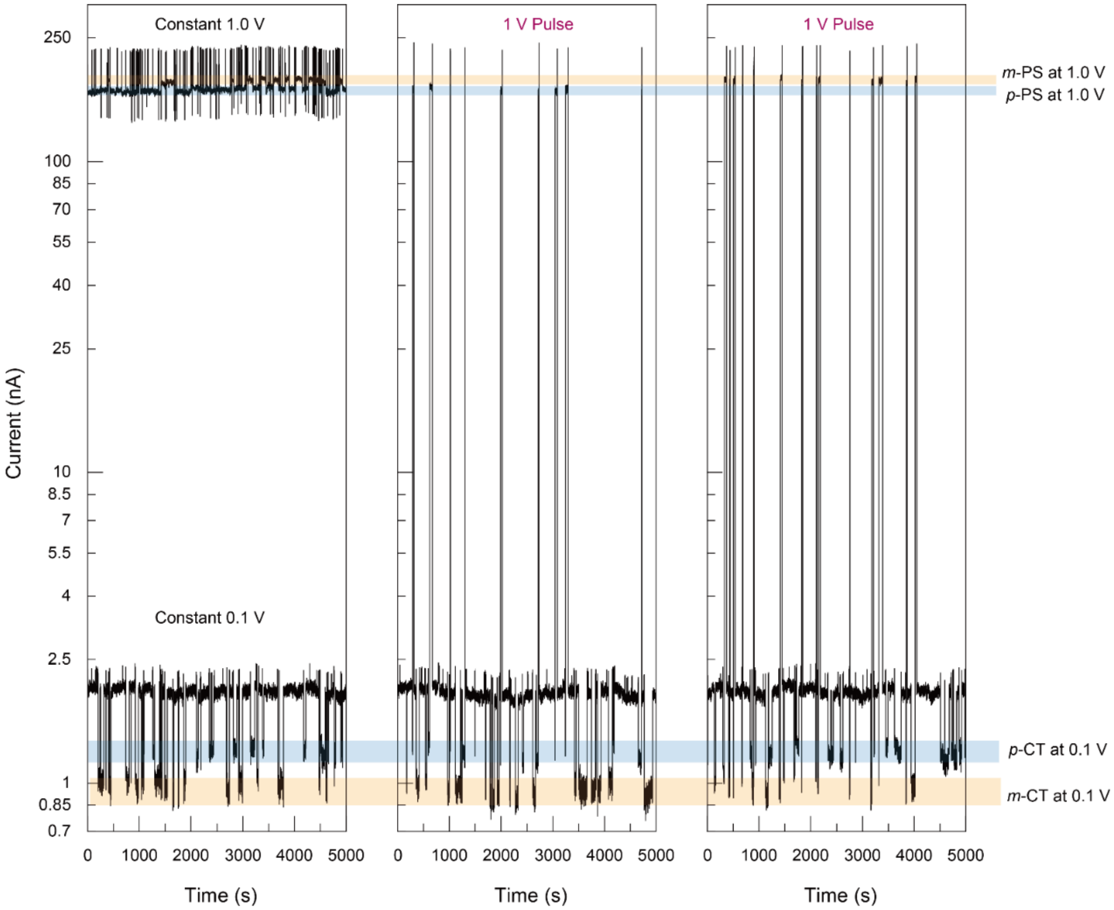

Fig. S83. Preparation of *p*-PS and *m*-PS on device 20#.

18. The multiple-time control of the regio- and stereo-selectivity

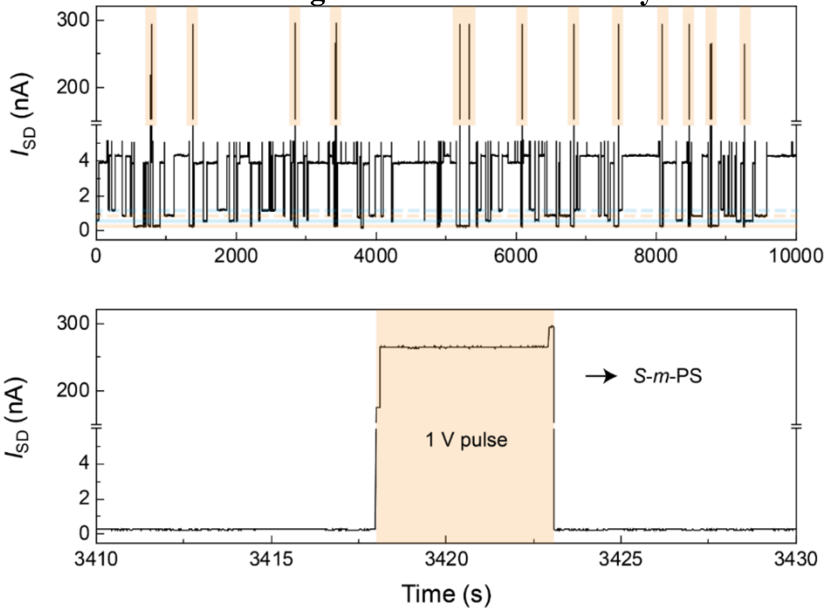

Fig. S84. Multiple-time direct synthesis of the *S-m*-PS. The Diels-Alder cycloaddition between acrylic acid (1 mM) and isoprene (1 mM) in trifluoroacetate was monitored at 100 K and 0.1 V bias voltage by the C^+ molecular bridge. A 1 V bias voltage was applied at the *S-m*-CT and removed at the C^+ state to prepare a *S-m*-PS. The corresponding enlarged image was provided in the bottom panel.

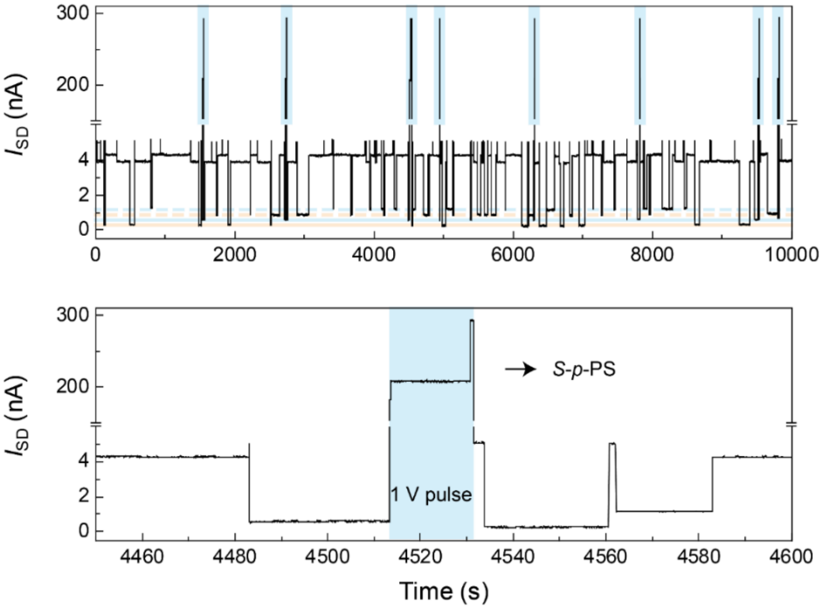

Fig. S85. Multiple-time direct synthesis of the *S-p*-PS. The Diels-Alder cycloaddition between acrylic acid (1 mM) and isoprene (1 mM) in trifluoroacetate was monitored at 100 K and 0.1 V bias voltage by the C^+ molecular bridge. A 1 V bias voltage was applied at the *S-p*-CT and removed at the C^+ state to prepare a *S-p*-PS. The corresponding enlarged image was provided in the bottom panel.

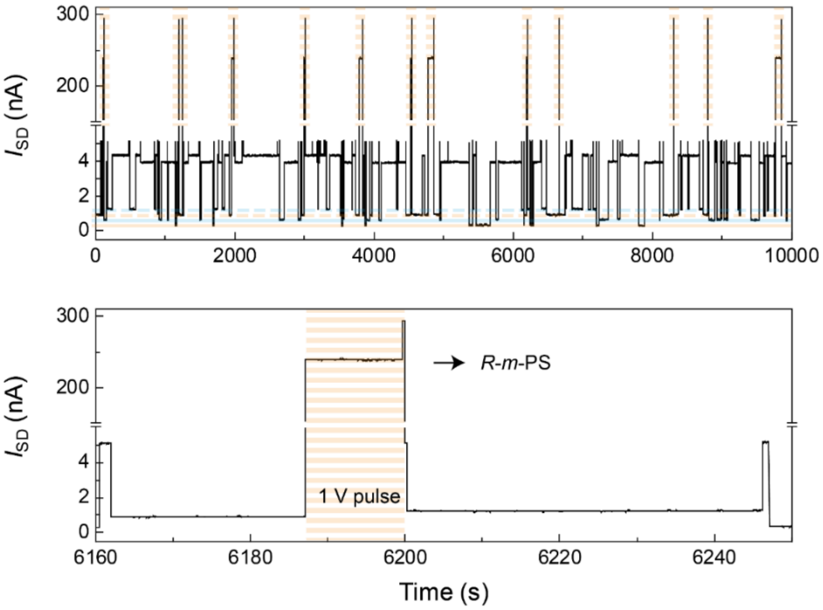

Fig. S86. Multiple-time direct synthesis of the *R-m*-PS. The Diels-Alder cycloaddition between acrylic acid (1 mM) and isoprene (1 mM) in trifluoroacetate was monitored at 100 K and 0.1 V bias voltage by the C^+ molecular bridge. A 1 V bias voltage was applied at the *R-m*-CT and removed at the C^+ state to prepare a *R-m*-PS. The corresponding enlarged image was provided in the bottom panel.

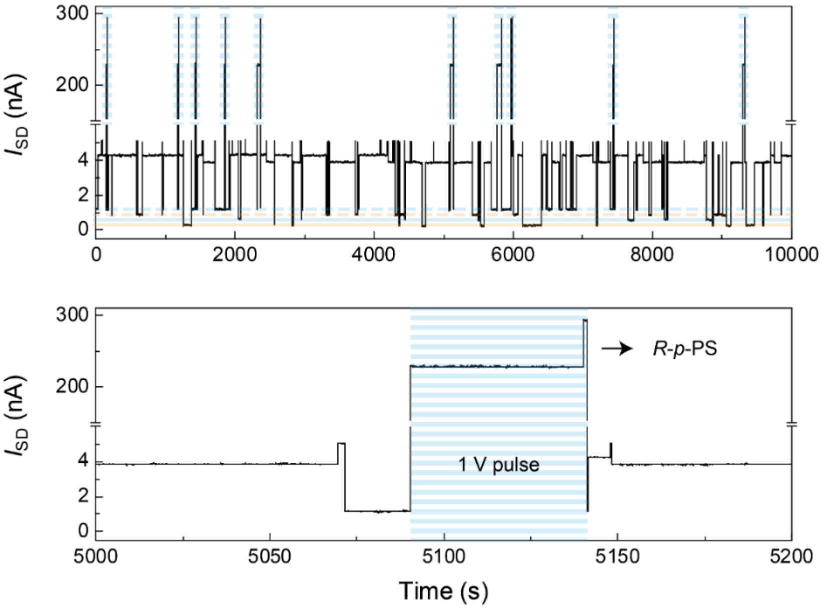

Fig. S87. Multiple-time direct synthesis of the *R-p*-PS. The Diels-Alder cycloaddition between acrylic acid (1 mM) and isoprene (1 mM) in trifluoroacetate was monitored at 100 K and 0.1 V bias voltage by the C^+ molecular bridge. A 1 V bias voltage was applied at the *R-p*-CT and removed at the C^+ state to prepare a *R-p*-PS. The corresponding enlarged image was provided in the bottom panel.

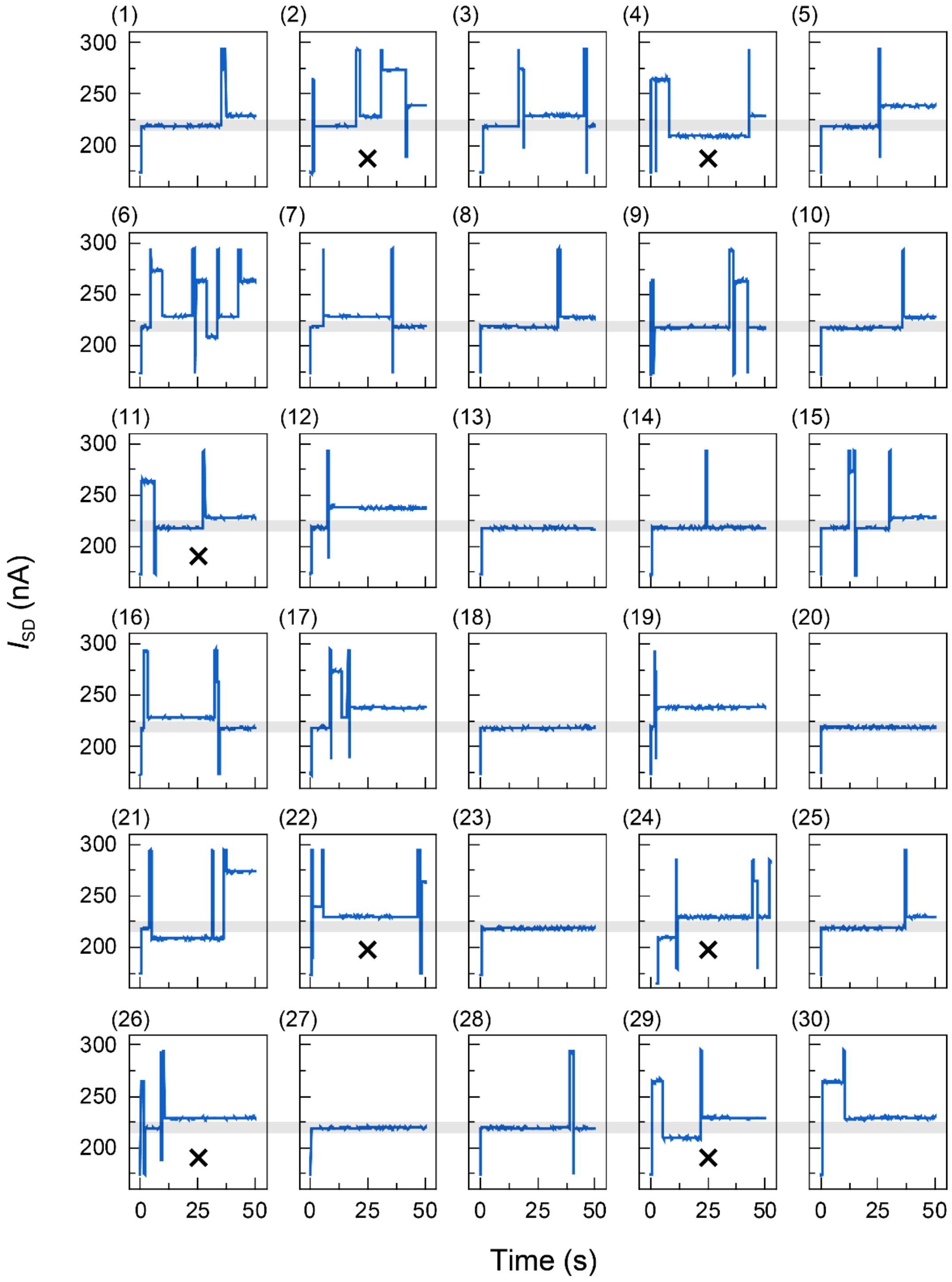

Fig. S88. Statistics of 30 times of a 1 V bias voltage applied at the *S*-*m*-CT. 50 s pulses were applied at the *S*-*m*-CT and the corresponding *I*–*t* curves were provided. There are 6 times failures to form PSs and direct conversion to C^+ or IS (marked as Numbers 2, 4, 11, 22, 26, and 29). This does not affect the asymmetry of the final product. However, in rare cases, it (Number 24) produces *S*-*p*-PSs directly due to the switching of the paths at the entrance. The 1 V bias voltage should be removed timely when the target product is not formed in a practical on-line asymmetric synthesis scenario.

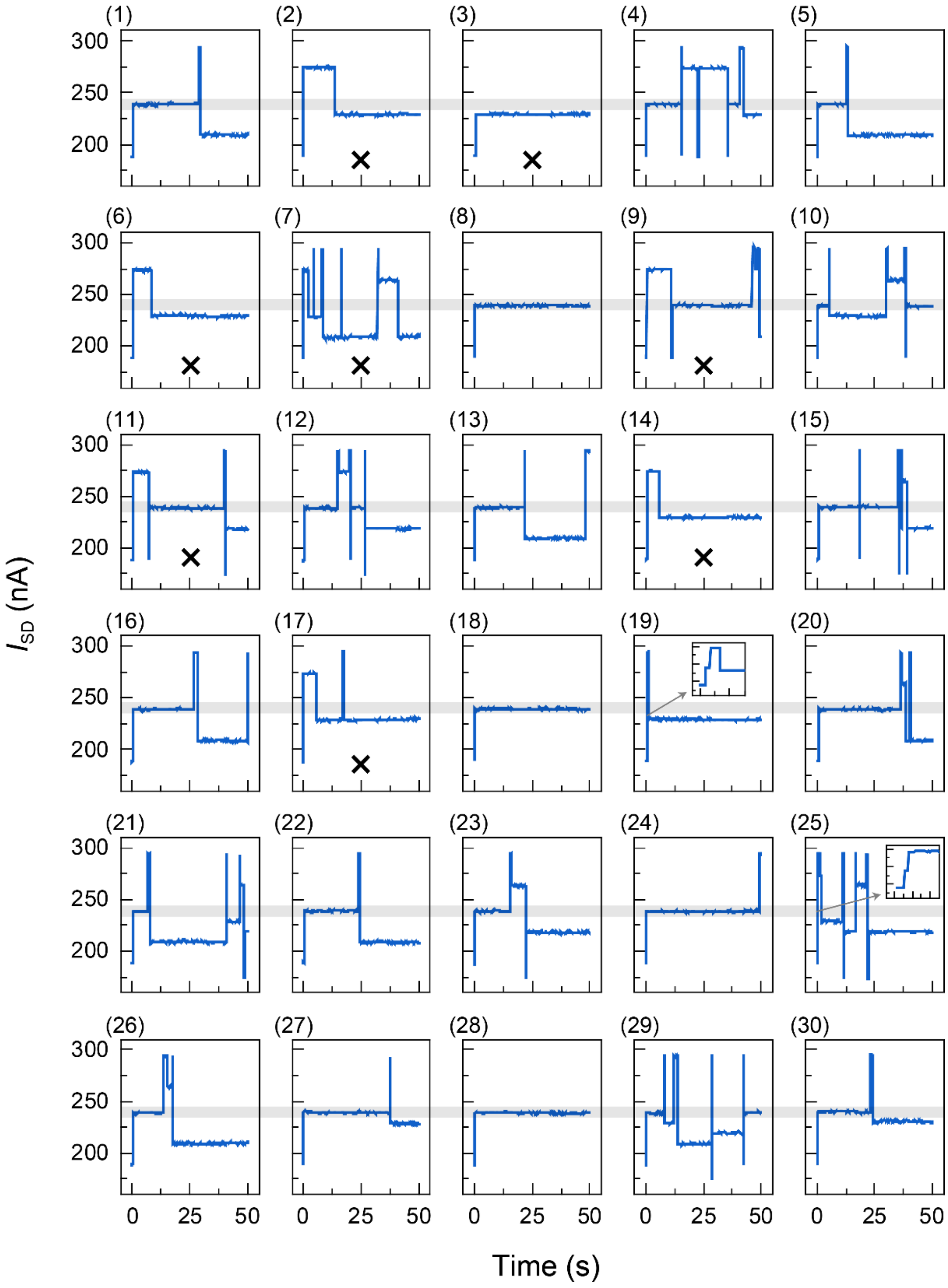

Fig. S89. Statistics of 30 times of a 1 V bias voltage applied at the *R*-*m*-CT. 50 s pulses were applied at the *R*-*m*-CT and the corresponding *I*–*t* curves were provided. There are 7 times failures to form PS and direct conversion to C^+ or IS (marked as Numbers 2, 6, 7, 11, 14, and 17). This does not affect the asymmetry of the final product. However, in rare cases, it (Number 3) produces *R*-*p*-PSs directly due to the switching of the paths at the entrance. The 1 V bias voltage should be removed timely when the target product is not formed in a practical on-line asymmetric synthesis scenario.

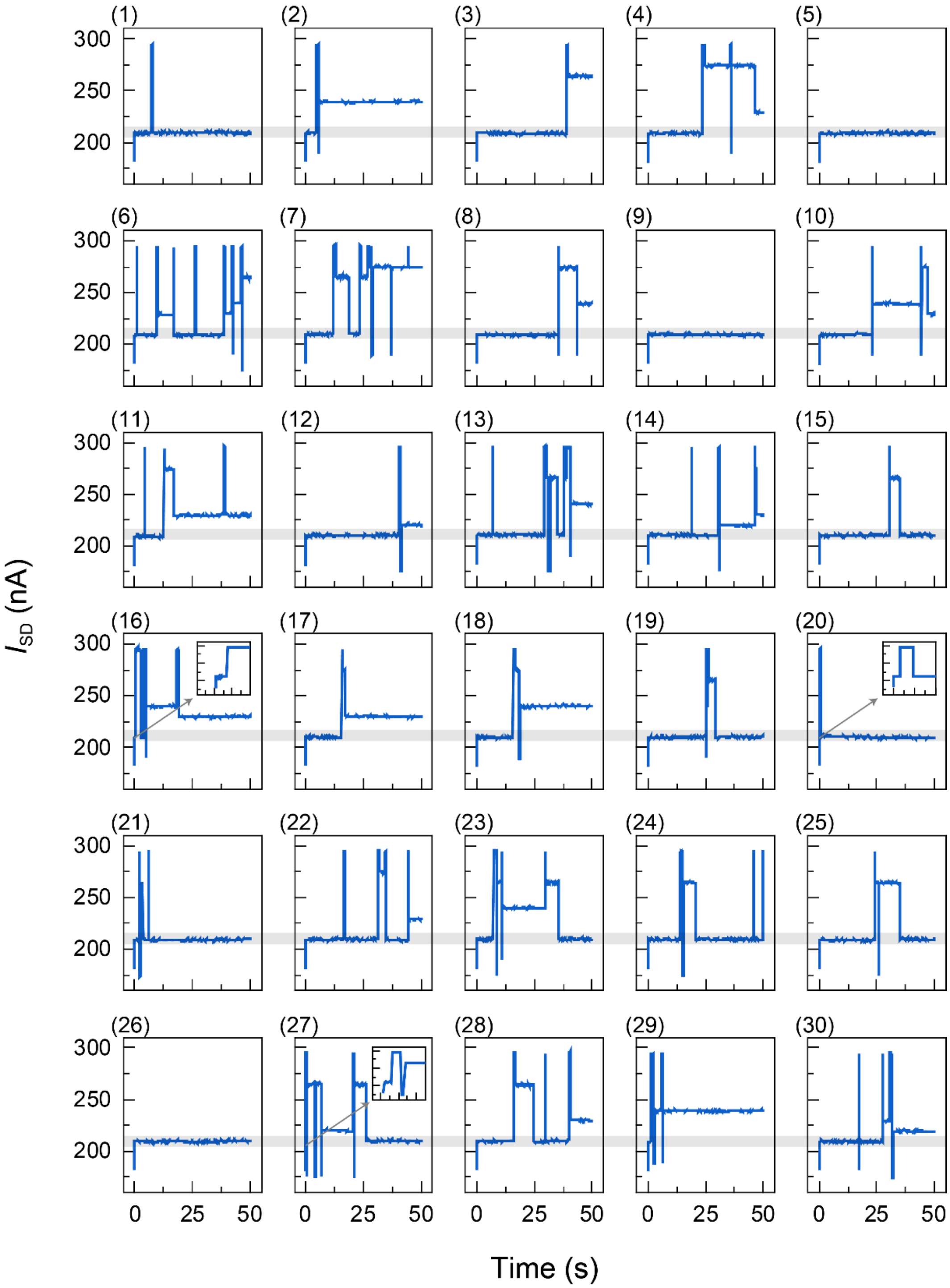

Fig. S90. Statistics of 30 times of a 1 V bias voltage applied at the *S*-*p*-CT. 50 s pulses were applied at the *S*-*p*-CT and the corresponding *I*–*t* curves were provided. All the operations produce *S*-*p*-PSs due to the relative lower TSs at the *para*-path.

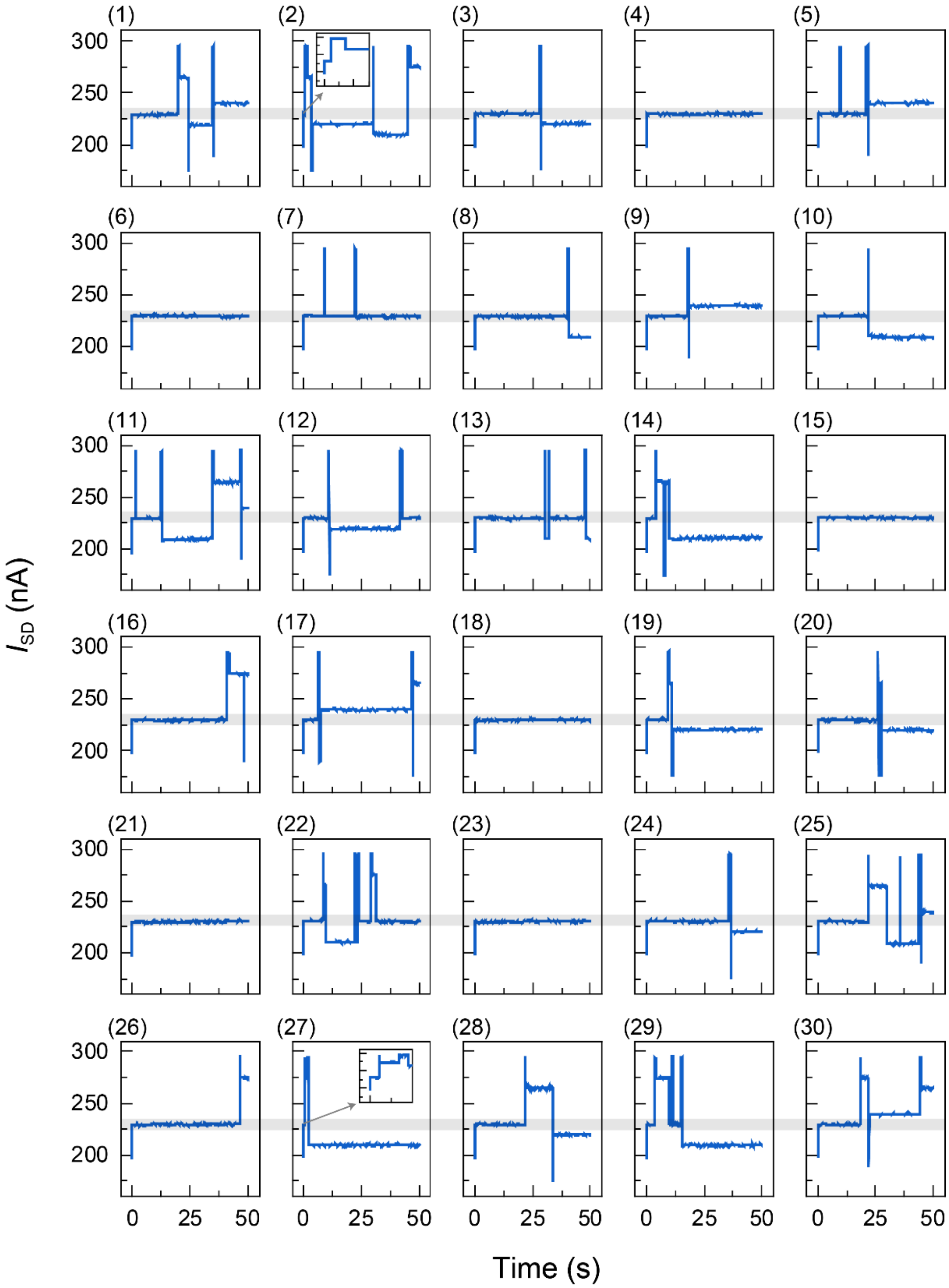

Fig. S91. Statistics of 30 times of a 1 V bias voltage applied at the *R*-*p*-CT. 50 s pulses were applied at the *R*-*p*-CT and the corresponding *I*–*t* curves were provided. All the operations produce *R*-*p*-PSs due to the relative lower TSs at the *para*-path.

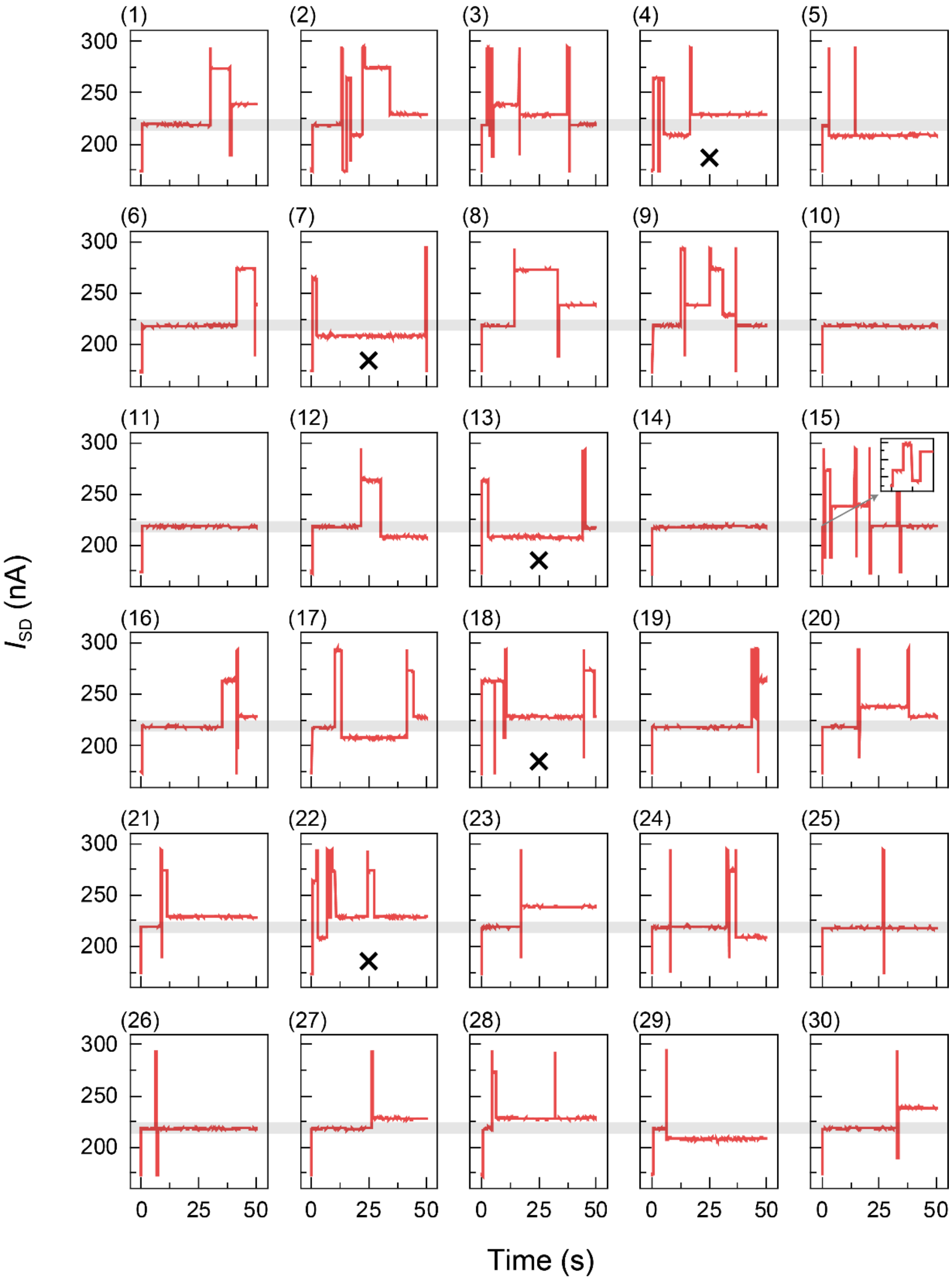

Fig. S92. Statistics of 30 times of a −1 V bias voltage applied at the *S*-*m*-CT. 50 s pulses were applied at the *S*-*m*-CT and the corresponding *I*–*t* curves were provided. There are 5 times failures to form PS and direct conversion to C^+ or IS (marked as Numbers 4, 7, 13, 18, and 22). This does not affect the asymmetry of the final product. The 1 V bias voltage should be removed timely when the target product is not formed in a practical on-line asymmetric synthesis scenario.

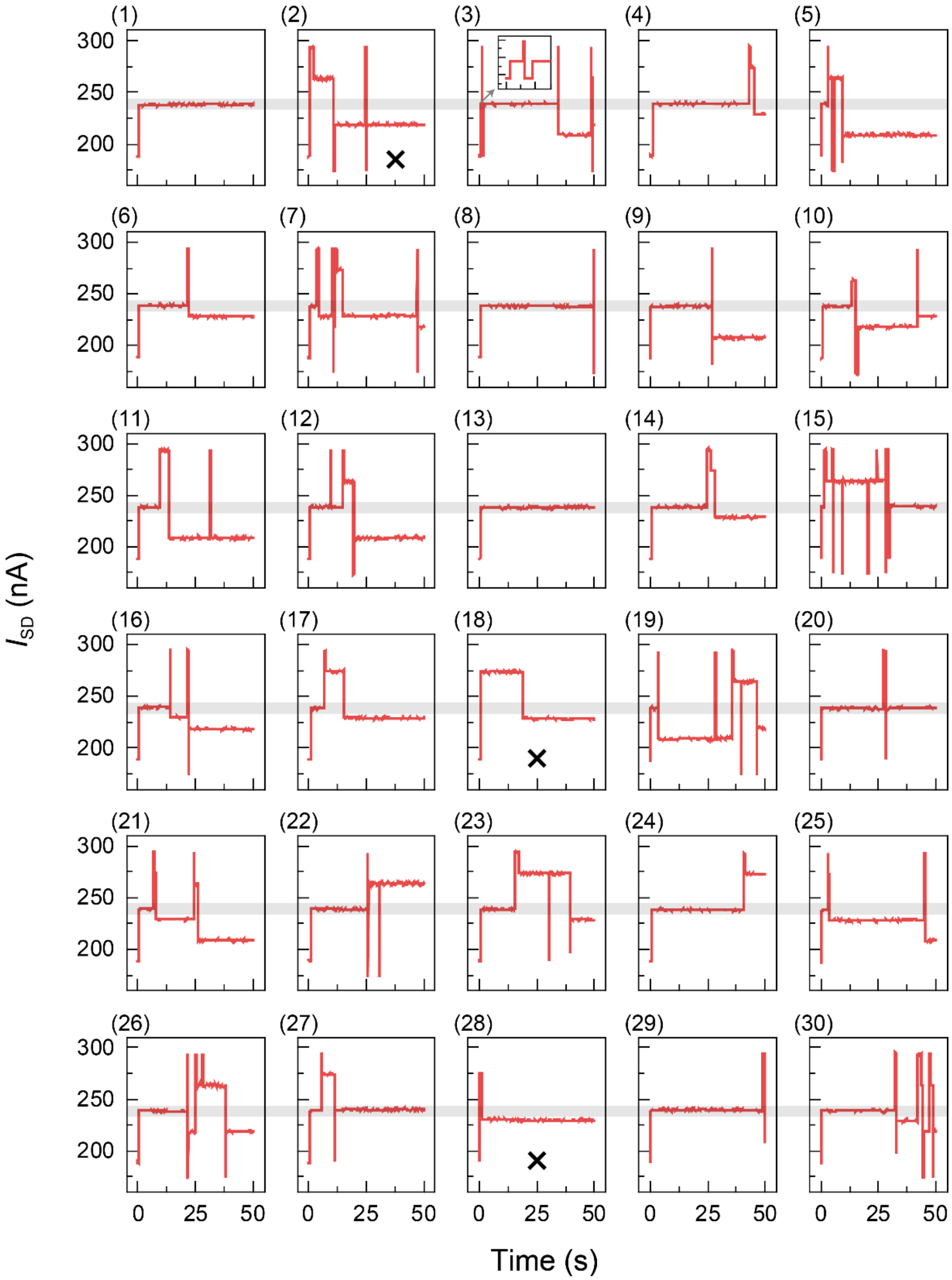

Fig. S93. Statistics of 30 times of a −1 V bias voltage applied at the *R*-*m*-CT. 50 s pulses were applied at the *R*-*m*-CT and the corresponding *I*–*t* curves were provided. There are 3 times failures to form PS and direct conversion to C^+ or IS (marked as Numbers 2, 18, and 28). This does not affect the asymmetry of the final product. The 1 V bias voltage should be removed timely when the target product is not formed in a practical on-line asymmetric synthesis scenario.

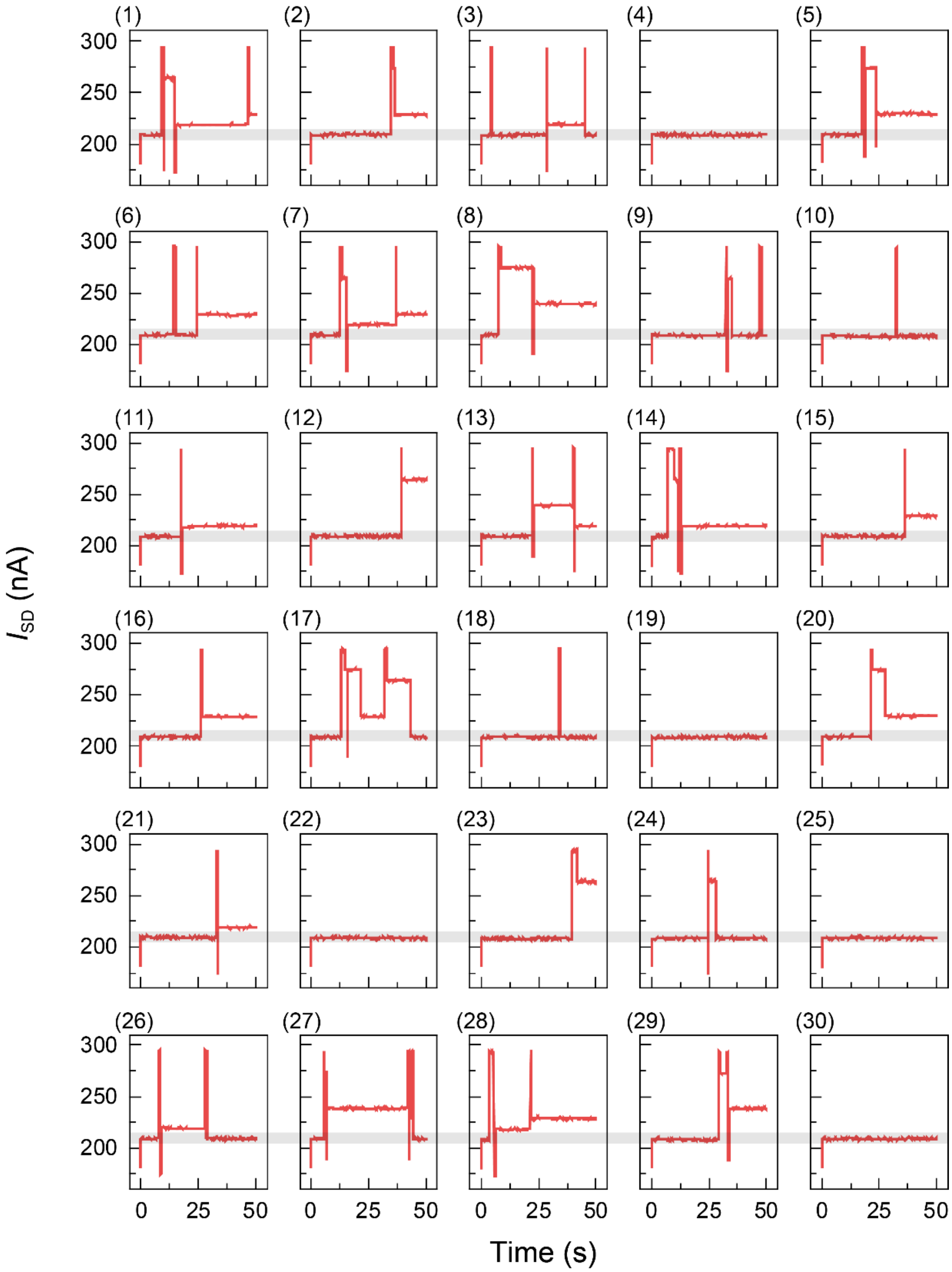

Fig. S94. Statistics of 30 times of a −1 V bias voltage applied at the *S*-*p*-CT. 50 s pulses were applied at the *S*-*p*-CT and the corresponding *I*–*t* curves were provided. All the operations produce *S*-*p*-PSs due to the relative lower TSs at the *para*-path.

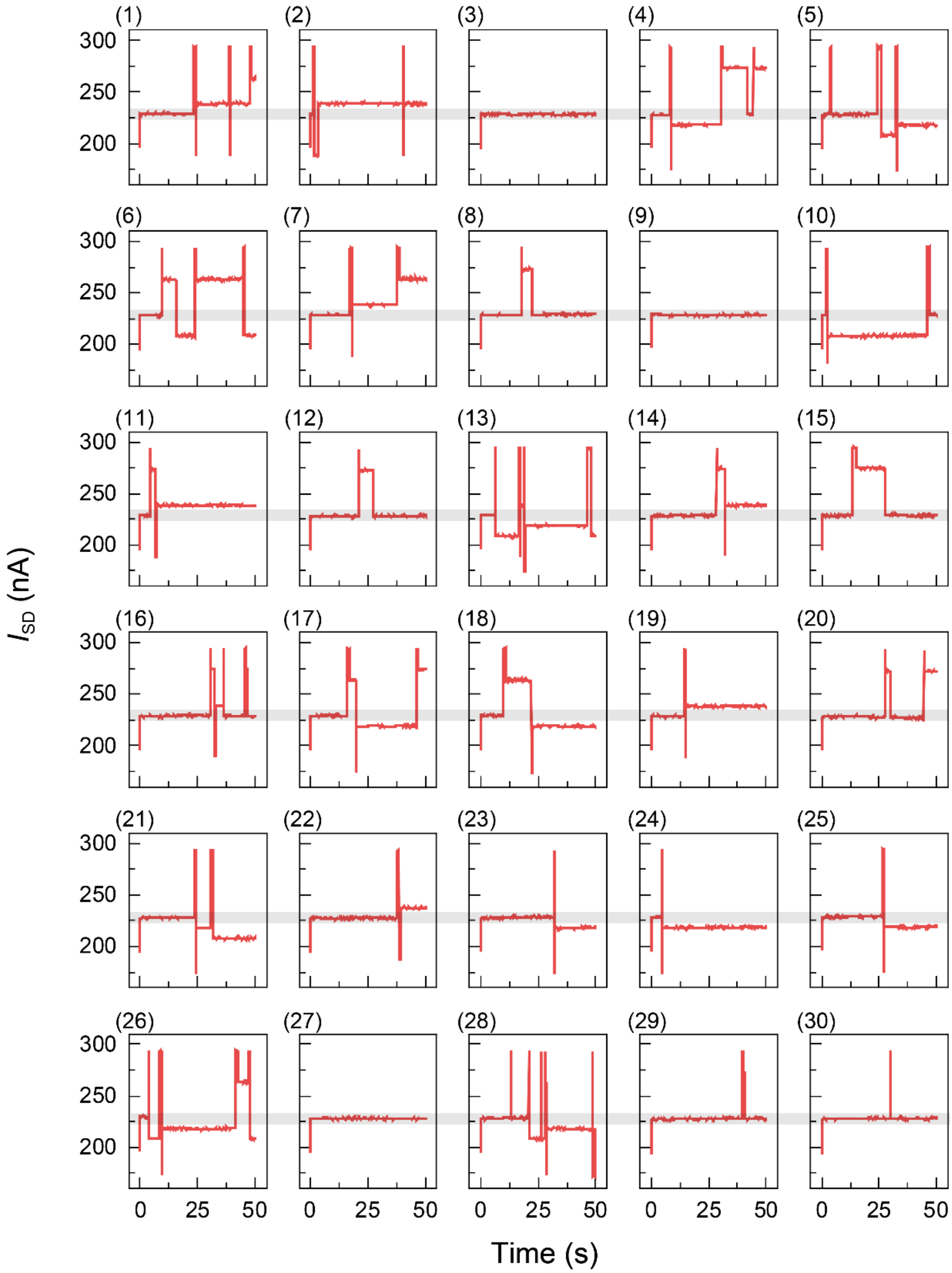

Fig. S95. Statistics of 30 times of a −1 V bias voltage applied at the *R*-*p*-CT. 50 s pulses were applied at the *R*-*p*-CT and the corresponding *I*–*t* curves were provided. All the operations produce *R*-*p*-PSs may to the relative lower TSs at the *para*-path.

19. The regulation of the reaction between maleimide and furan

It is well known that the selective synthesis of *endo* (kinetic) and *exo* (thermodynamic) products of the cycloaddition between maleimide and furan requires careful control of temperature and reaction duration, which highlights the possibility of direct precise asymmetric synthesis. A previous study[2] has reported the reaction trajectories between maleimide and furan by real-time electrical monitoring. Owing to the excellent temporal resolution and electrical sensitivity, the pre-reaction CT complex and corresponding product state, as well as the transformation relationship involving the *endo* and *exo* channels, were accurately recorded (Fig. S96A).
For *endo* & *exo* on-line asymmetric synthesis, the Diels-Alder reaction between maleimide and furan shows 5 conductance states (Fig. S97). This is a well-studied reaction system in our previous work[2] and all the conductance can be assigned accurately. Specifically, the conductance of the maleimide state (RS) can be assigned without the addition of furan. The pre-reaction complexes (*endo*-CT and *exo*-CT) can be assigned by furan concentration-dependent measurements at low temperatures (at which the Diels-Alder cannot occur). The occupancies of the CT complexes increase with the increased concentrations of furan and the occupancy difference between *endo* and *exo* configurations depends on their thermodynamic stability. The *endo* configuration is more stable than the *exo* configuration due to the secondary orbital interaction, leading to a higher occupancy of the *endo* configuration than that of the *exo* configuration. In addition, by replacing furan with tetrahydrofuran (Fig. S98) or replacing maleimide with succinimide (Fig. S99), these control experiments indicate the formation of the CT complexes between maleimide and furan, as well as the subsequent cycloaddition. Next, the newly appeared conductance states at high temperatures can be assigned to the product states, where *endo* and *exo* products can be distinguished by temperature-dependent measurements (the *endo* pathway is a kinetically controlled process and *exo* is thermodynamical) and the conversion time sequence. The assignments of the 5 conductance states are in line with the theoretical studies of the transmission spectra. Here, consistent results were observed during the monitoring with the constant bias voltage, and we push forward this ability to realize on-line asymmetric synthesis.
Both cycloaddition channels can be turned on by strong EEF (1 V bias) at low temperature (200 K) (Fig. S96B). Long-term monitoring at constant voltages of 0.1 and 1 V, that is, the single-molecule behaviors in an equilibrium state, is shown in Fig. S96C. Only conversion among the maleimide reactant substrate (RS), *endo*-CT complex, and *exo*-CT complex was detected at 0.1 V, while the reaction smoothly proceeded at 1 V owing to EEF catalysis. Note that the stepwise pathway with the zwitterionic intermediate could not be observed here because of the low temperature. According to the statistical results (Fig. S96C, right-hand panel), there was no obvious selectivity, which was attributed to elimination of the energetic differences between the *endo* and *exo* channels by the strong EEF[2].
To realize asymmetric synthesis of the *endo*- (*exo*-) PS, a 1 V bias voltage pulse (1 s) was applied to the current *endo*- (*exo*-) CT state monitored at 0.1 V (Fig. S96D and 96E). For the assignment of the current levels, refer to the constant-bias experiment (Fig. S96C). Obvious polarization of the PS between *endo* and *exo* was found according to the statistical results (right-hand panels in Figs. S96D and 96E), which shows the feasibility of on-line regulation. The electrical pulse of 1 V was chosen by considering two factors: low voltage to ensure the stability of the SMJ and high voltage to minimize cycloaddition of the TS, against switching between the two paths at the entrance, that is, the conversion between the CT states. In addition, according to the period of the reaction, the width of the pulse determines the polarization between the PSs (Fig. S96F and 96G).

The detailed operations with different-width pulses are shown in Figs. S100 and 101. A shorter width leads to the system being farther away from the equilibrium and causes higher polarization. Repeated pulses can be applied to prepare a new PS with determined configuration at the next corresponding CT state owing to the reversibility of cycloaddition. Instantaneous removal of 1 V bias in the product state can reserve single *endo*- and *exo*- products in the junction at low temperatures and biases, respectively. Other 20 prepared single-product devices are provided in Figs. S102−121, showing the reproducibility. In addition, the reversibility and the restrictiveness of the potential energy surface can be changed by changing the substituents on the furan ring (e.g., adding a 3-methoxyl group), which leads to permanent preparation of the desired configuration (Fig. S122). The substituent effectively prevents switching between the two paths, which will inspire single-selectivity synthesis in the future.

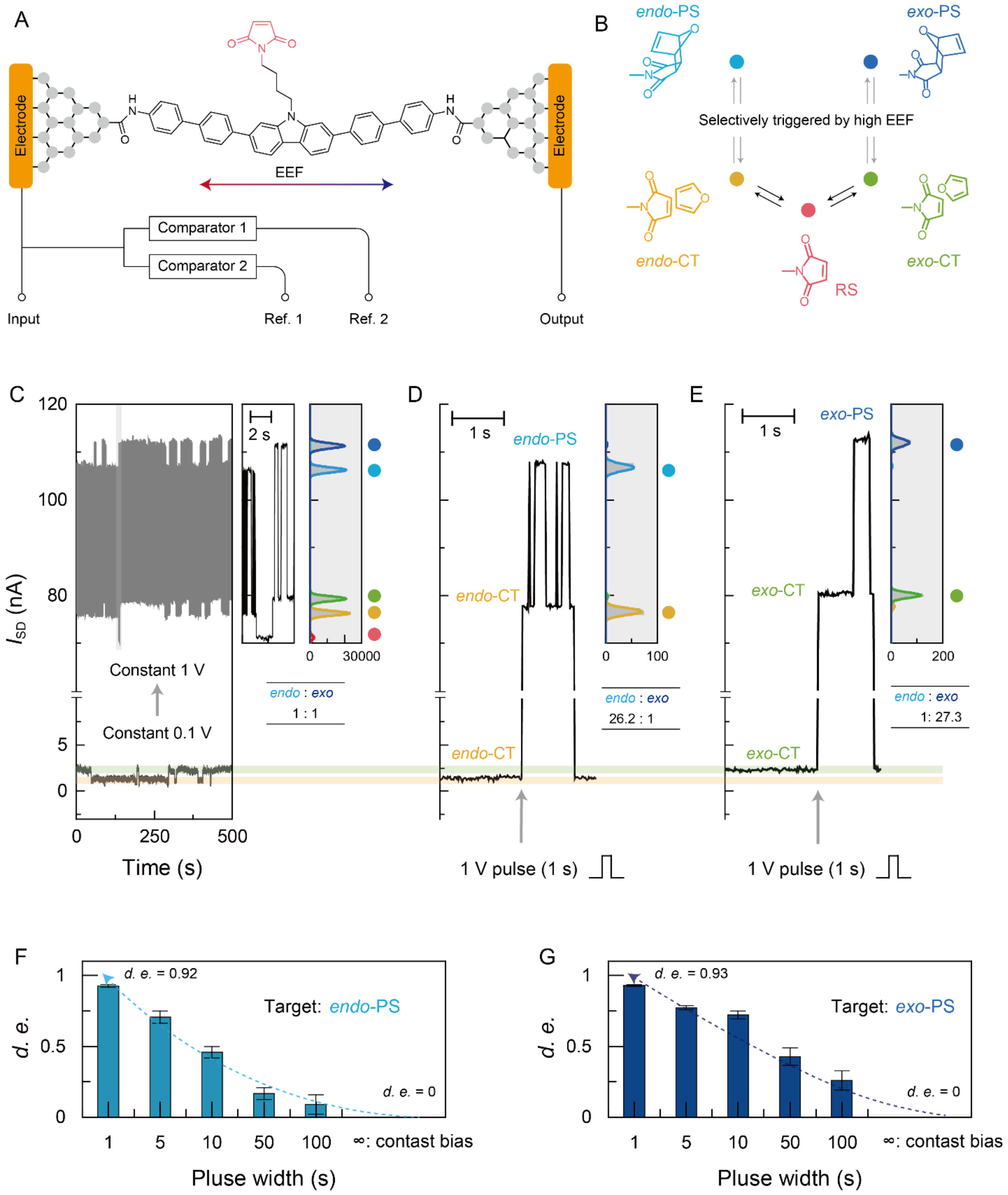

Fig. S96. On-line asymmetric synthesis of *endo*- and *exo*-products. A. Schematic of a single-maleimide SMJ and its reaction with furan, involving the asymmetric *endo* and *exo*-paths. **B.** Detected species and corresponding conversion relationship in the reaction. **C.** Monitoring of the Diels–Alder reaction at 200 K, 0.1 V, and 1 V. The right-hand panel shows the enlarged *I*–*t* curve and statistical frequency distributions from the 10000 s *I*–*t* curve. The ratio of the occupancy between the *endo*- and *exo*-PSs is shown in the table. **D.** *I*–*t* curves with a constant 1 V pulse

applied to the *endo*-CT state for 1 s monitored at 0.1 V. The right-hand panel shows the statistics of the *I*–*t* curves at several 1 V pulse plateaus, which are shown in the Supplementary Materials. The ratio of the occupancy between the *endo*- and *exo*-PSs is shown in the table. **E.** *I*–*t* curves with a constant 1 V pulse applied to the *exo*-CT state for 1 s monitored at 0.1 V. The right-hand panel shows the statistics of the *I*–*t* curves at several 1 V pulse plateaus, which are shown in the Supplementary Materials. The ratio of the occupancy between the *endo*- and *exo*-PSs is shown in the table. **F.** The diastereomeric excess (*d.e.*) of *endo*-PS versus the width of the 1 V pulse, where the error bars were derived from five devices. **G.** The *d.e.* of *exo*-PS versus the width of the 1 V pulse, where the error bars were derived from five devices.

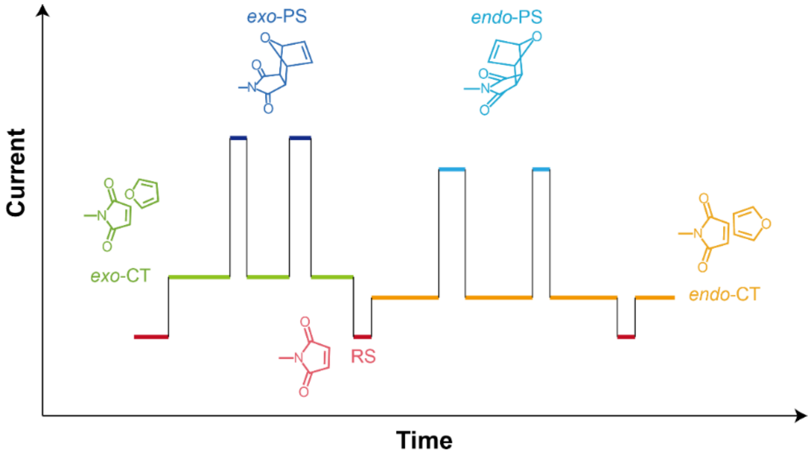

Fig. S97. Idealized *I*–*t* curves and corresponding assignments of the conductance states. Note that the zwitterionic intermediate cannot be observed here due to the low temperature (200 K).

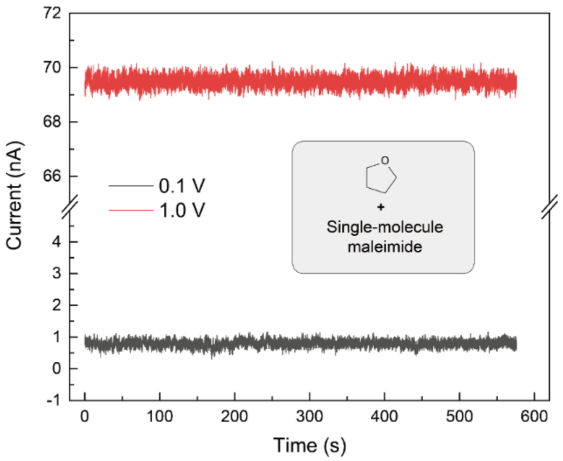

Fig. S98. Control experiment of replacing furan with tetrahydrofuran. No obvious fluctuations were observed, indicating that the chemical reactions or the formation of a charge transfer complex between furan and maleimide result in the variations of the measured conductance.

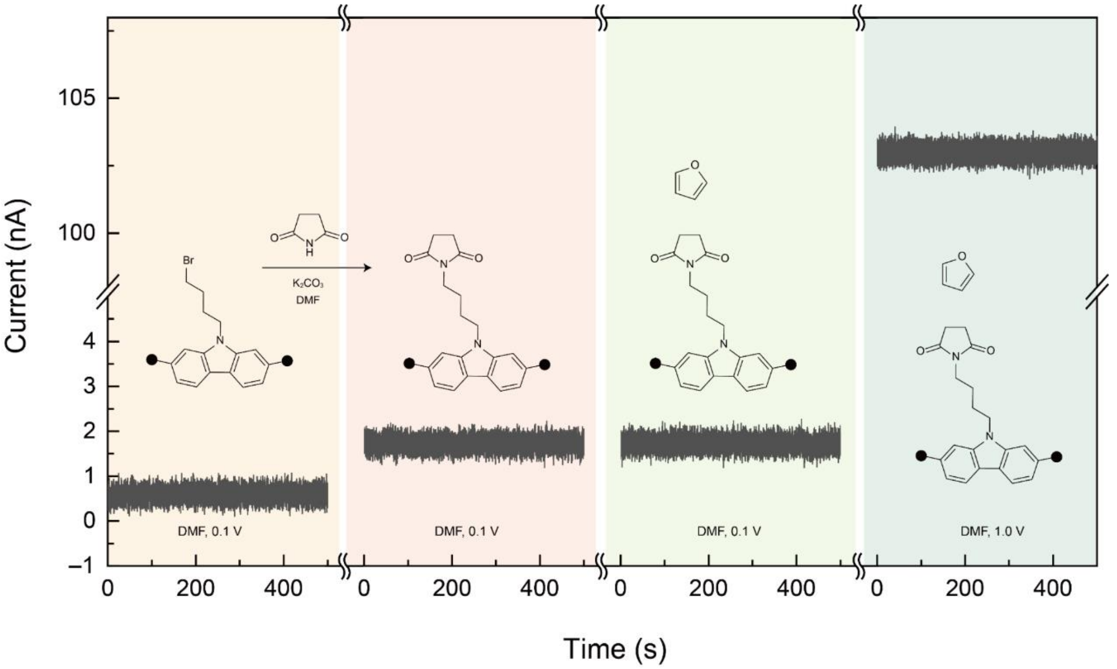

Fig. S99. Control experiment of replacing maleimide with succinimide. No obvious fluctuations were observed, indicating that the chemical reactions or the formation of a charge-transfer complex between furan and maleimide result in the variations of the measured conductance.

20. The pulse width dependence in the synthesis of *endo* & *exo* products

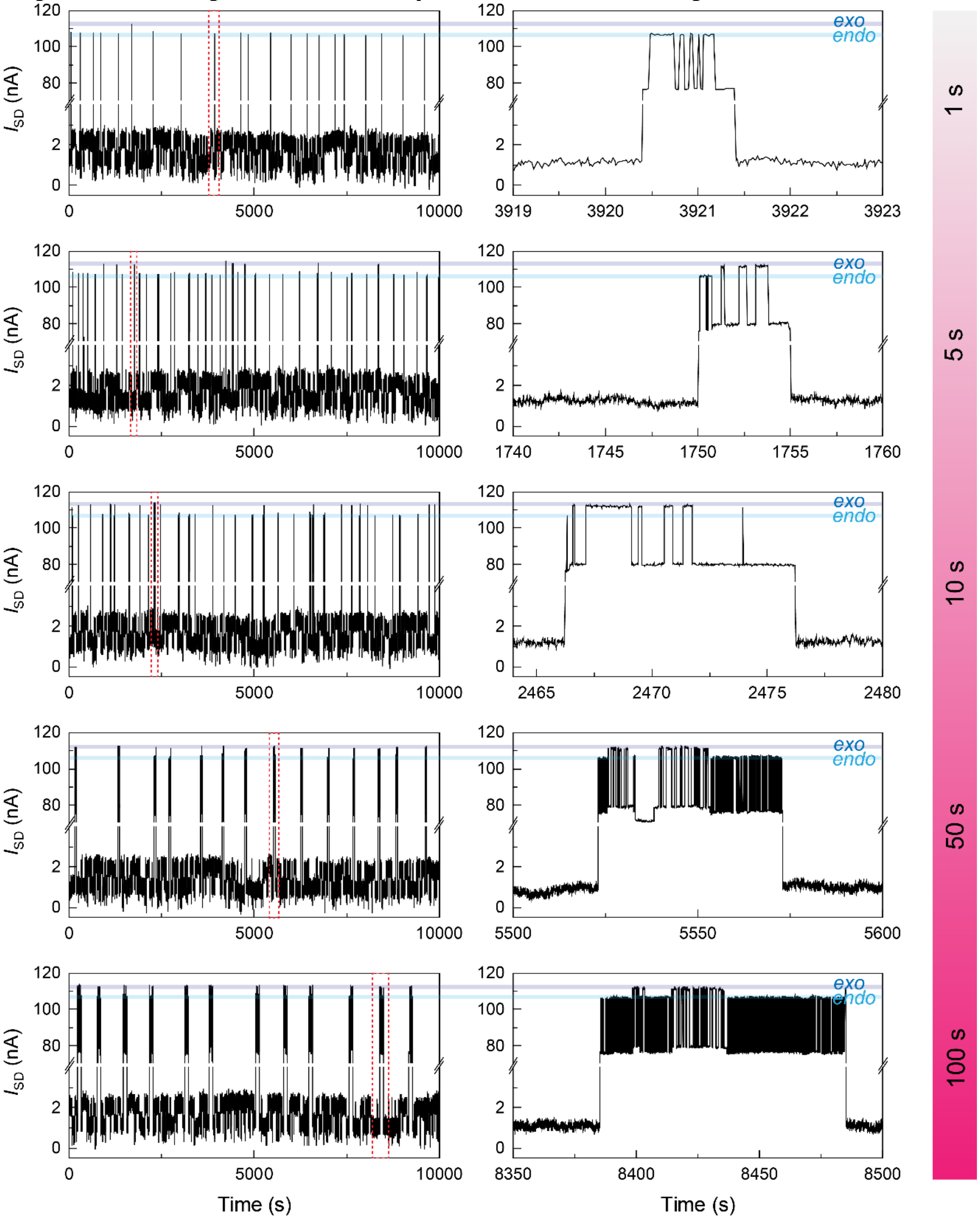

Fig. S100. The pulse width dependence in the synthesis of *endo*-products. The Diels-Alder cycloaddition between a single maleimide and furan (1 mM) in trifluoroacetate was monitored at 200 K and 0.1 V bias voltage. Multiple times (1 s, 5 s, 10 s, 50 s, and 100 s) pulses (1 V) were applied at the *endo*-CT state. The trajectories of the *exo*-path increased with widening the pulse. The statistical results at the 1 V plateau were provided in Fig. S96F. The corresponding enlarged images were provided in the right panel, respectively.

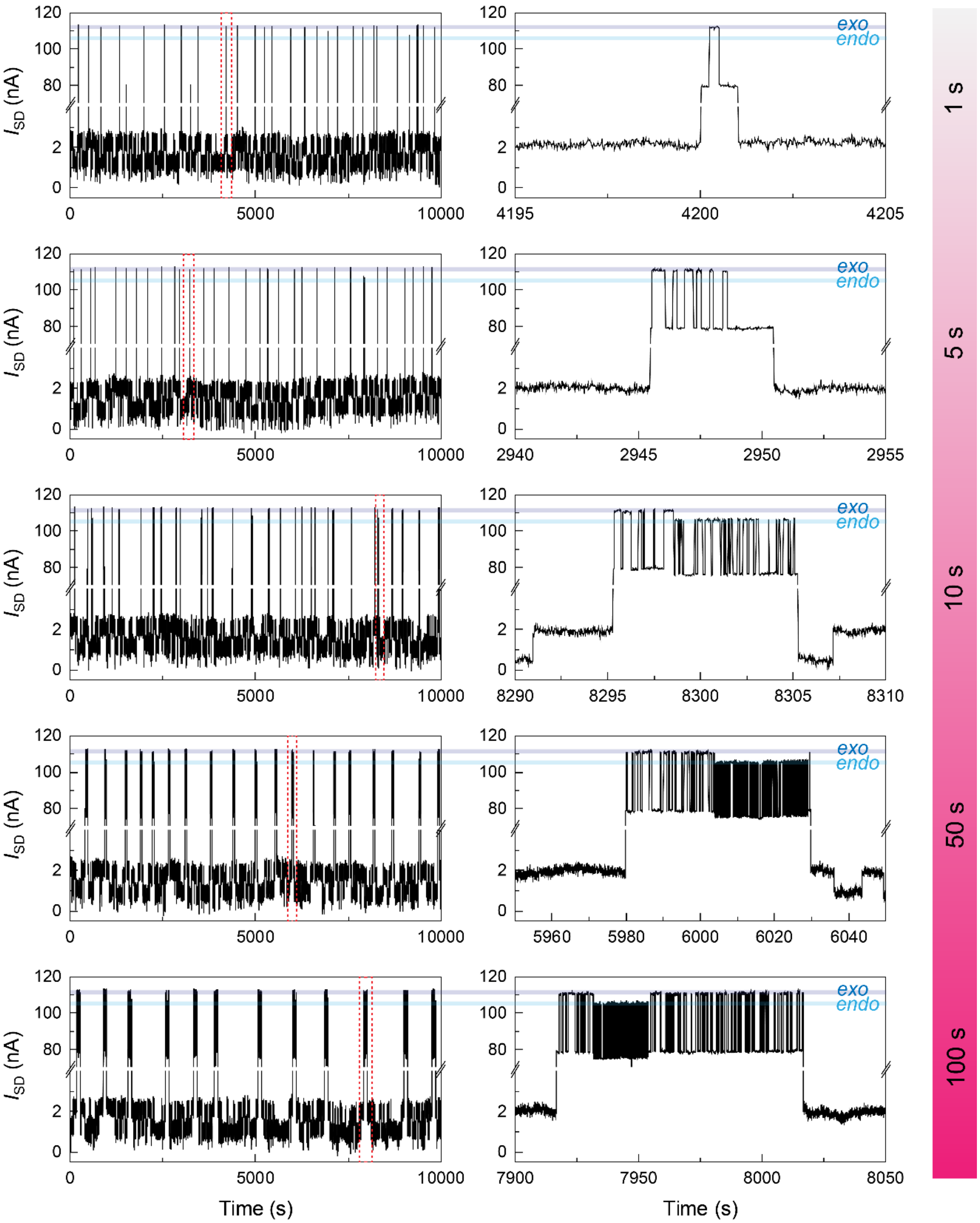

Fig. S101. The pulse width dependence in the synthesis of *exo*-products. The Diels-Alder cycloaddition between a single maleimide and furan (1 mM) in trifluoroacetate was monitored at 200 K and 0.1 V bias voltage. Multiple times (1 s, 5 s, 10 s, 50 s, and 100 s) pulses (1 V) were applied at the *exo*-CT state. The trajectories of the *endo* path increased with widening the pulse. The statistical results at the 1 V plateau were provided in Fig. S96G. The corresponding enlarged images were provided in the right panel, respectively.

21. The preparation and retention of *endo* and *exo* PSs on other 20 single-molecule devices

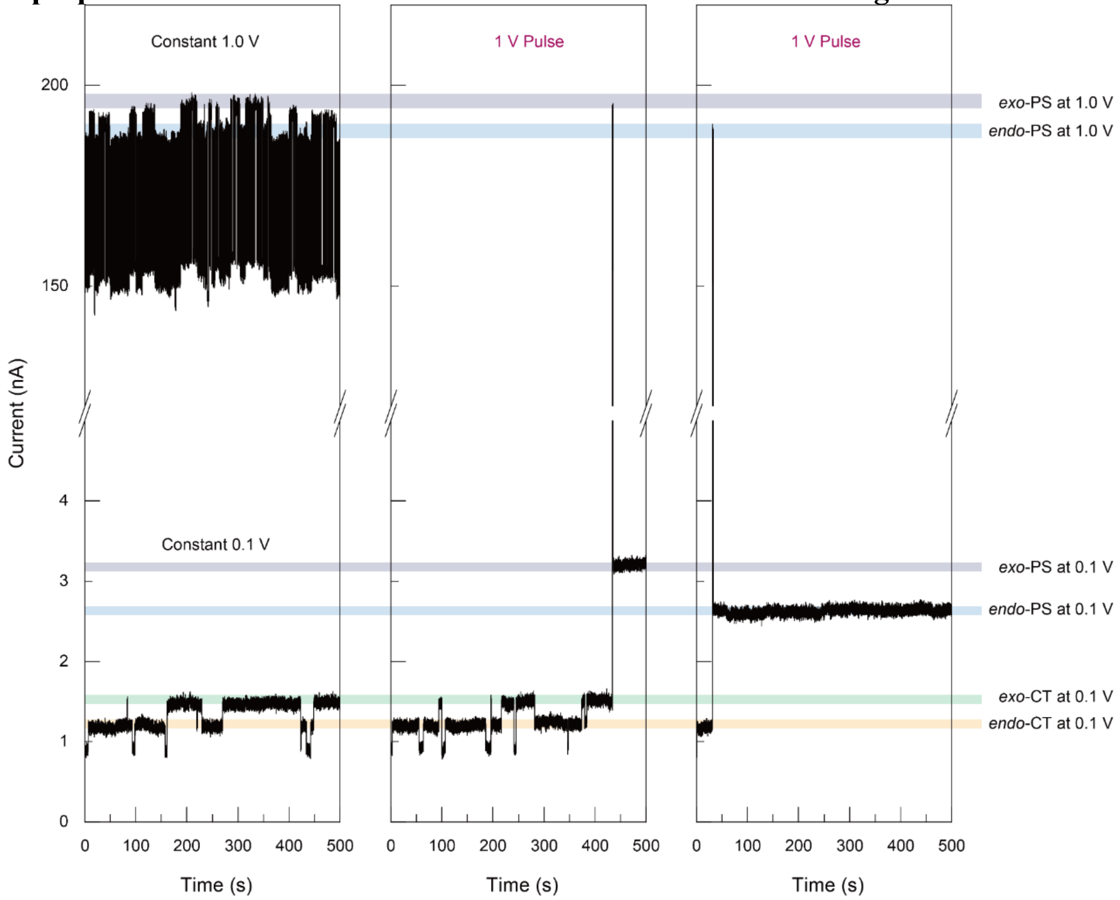

Fig. S102. Preparation and retention of *endo* and *exo* PSs on device 21#. The *I*–*t* measurements with constant 0.1 V and 1 V bias voltages were provided at the left panel as a reference. For the preparation of the *exo*-PS (middle panel), a 1 V bias voltage was applied at the *exo*-CT measured by 0.1 V, and instantaneously removed at the *exo*-PS measured by 1 V. Due to the low temperature (200 K) and relative high energy of the retro-Diels-Alder reaction at 0.1 V, the *exo*-PS could be reserved. For the preparation of the *endo*-PS (right panel), the RS and CT could be re-prepared by a high voltage and the retro-Diels-Alder reaction. Then, based on the monitoring of the conversion between RS and CT, a 1 V bias voltage was applied at the *endo*-CT state measured by 0.1 V, and instantaneously removed at the *endo*-PS measured by 1 V. Due to the low temperature (200 K) and relative high energy of the retro-Diels-Alder reaction at 0.1 V, the *endo*-PS could be reserved.

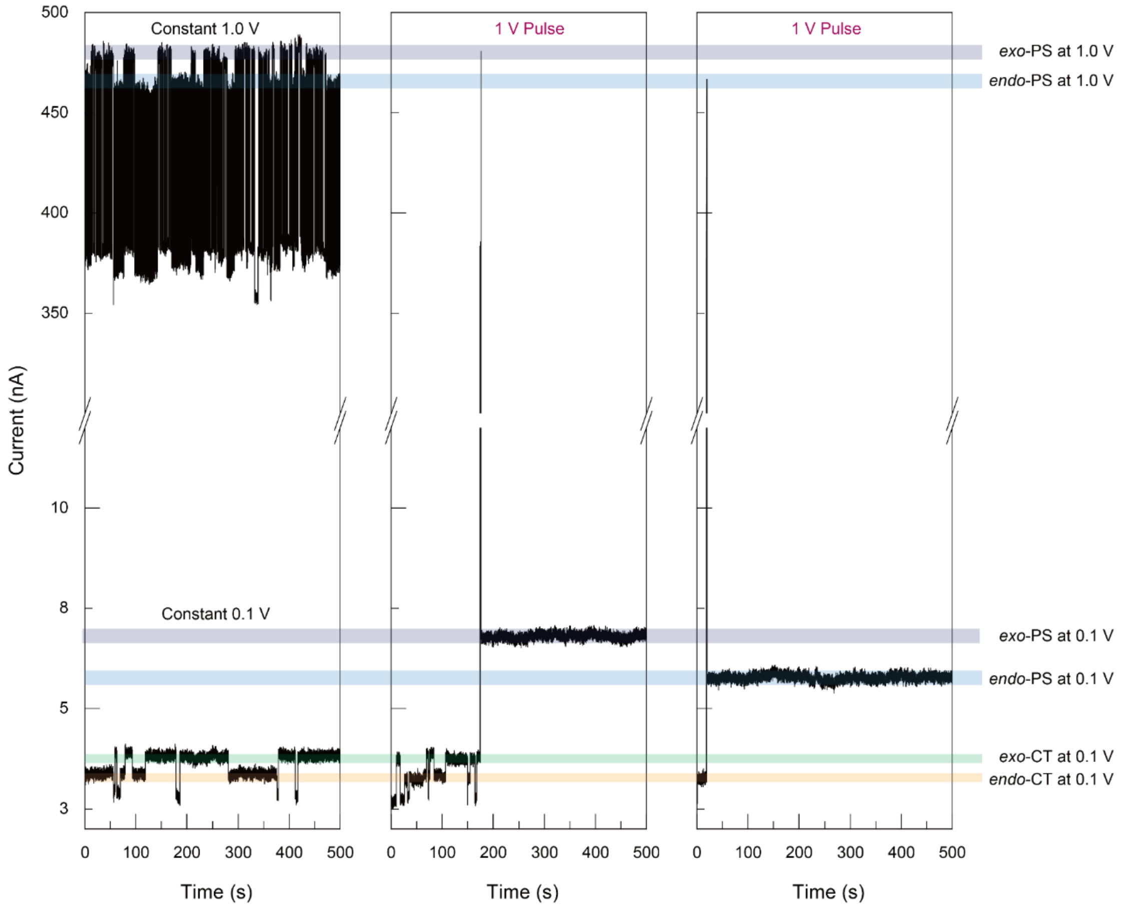

Fig. S103. Preparation and retention of *endo* and *exo* PSs on device 22#.

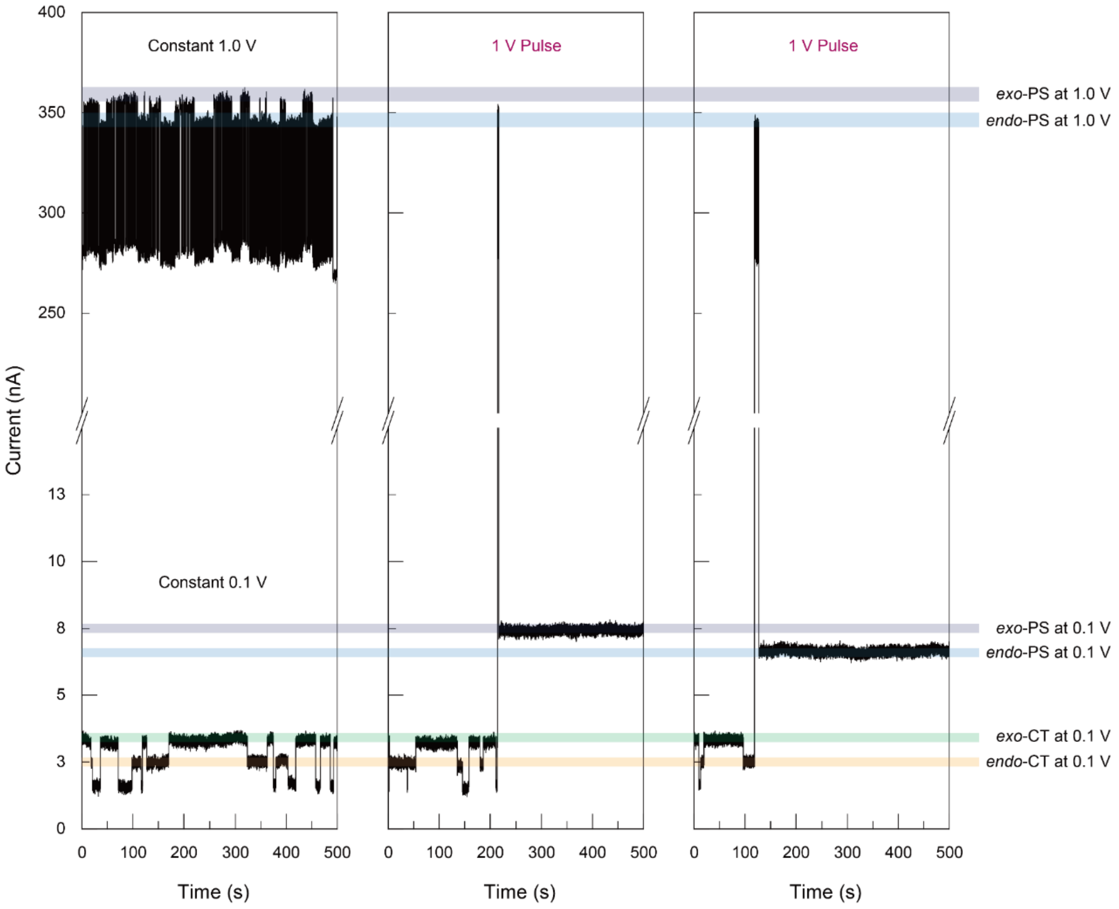

Fig. S104. Preparation and retention of *endo* and *exo* PSs on device 23#.

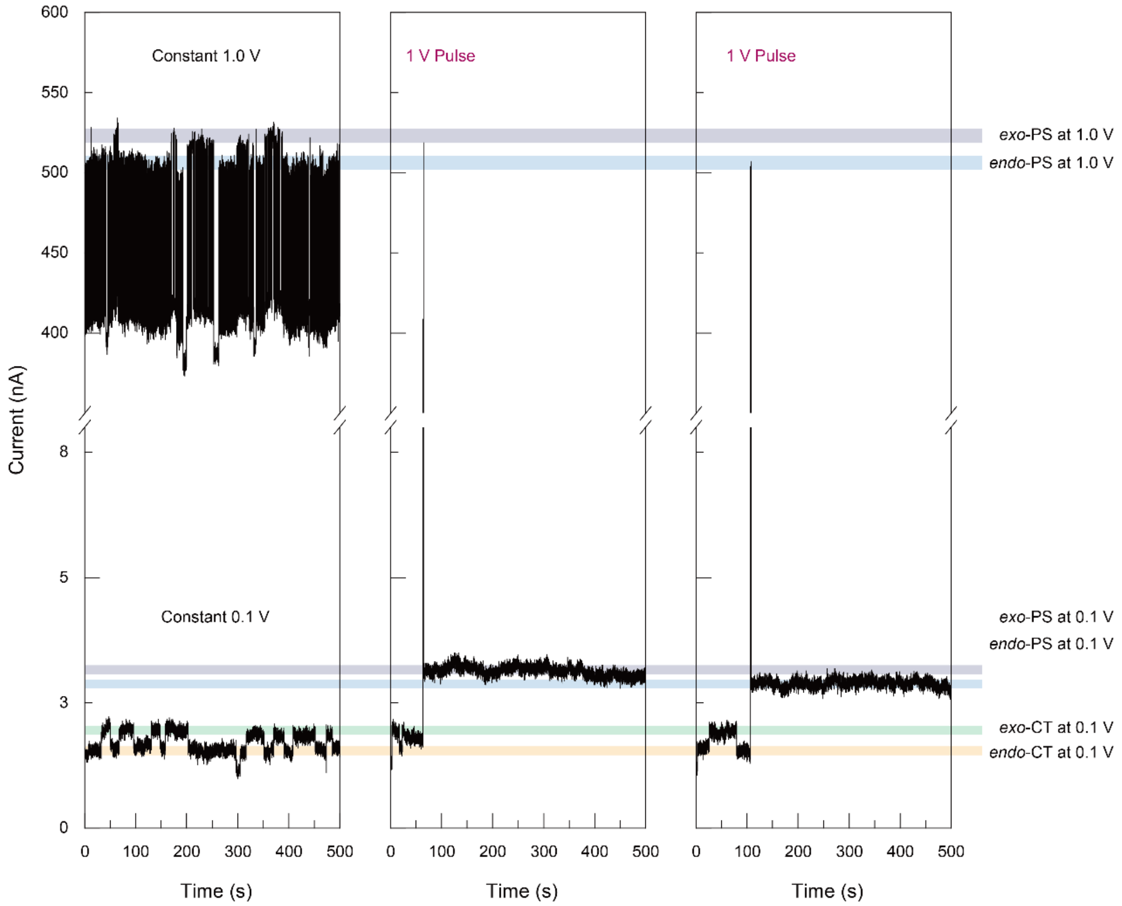

Fig. S105. Preparation and retention of *endo* and *exo* PSs on device 24#.

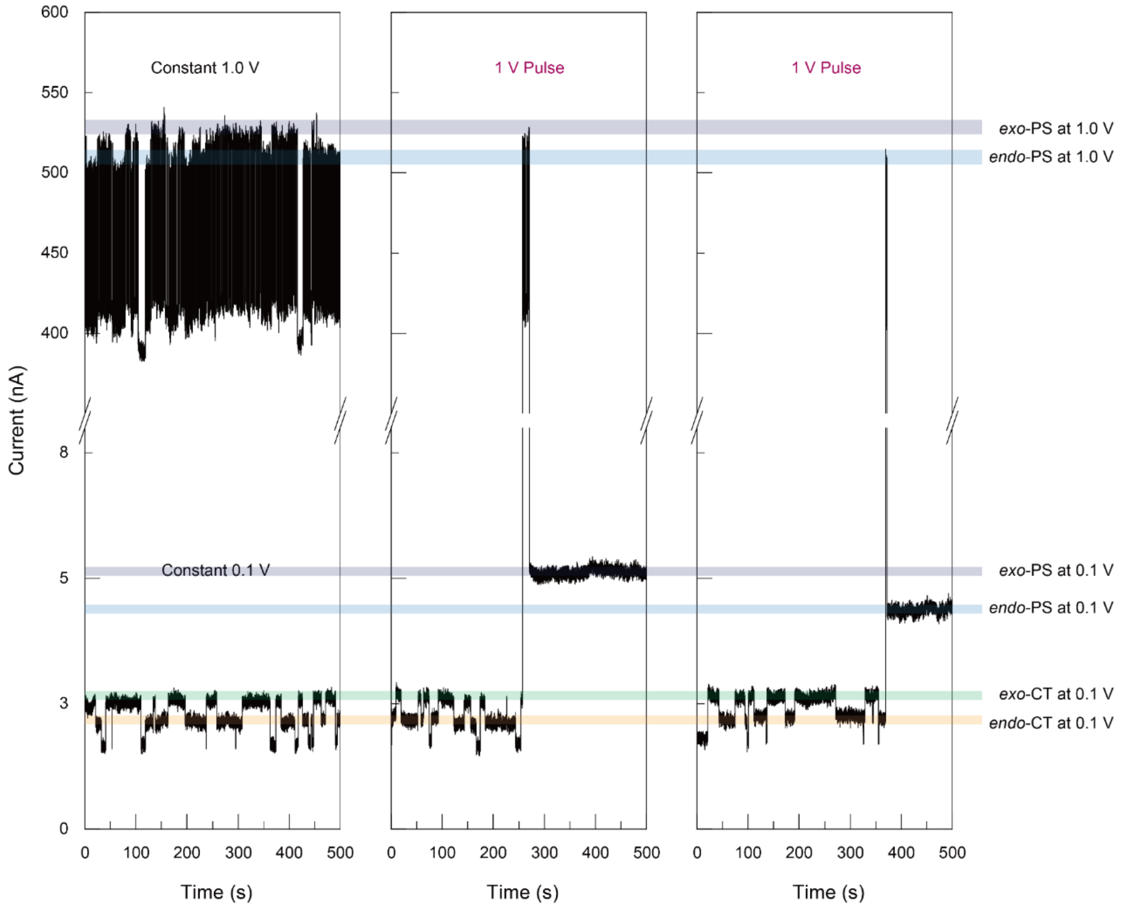

Fig. S106. Preparation and retention of *endo* and *exo* PSs on device 25#.

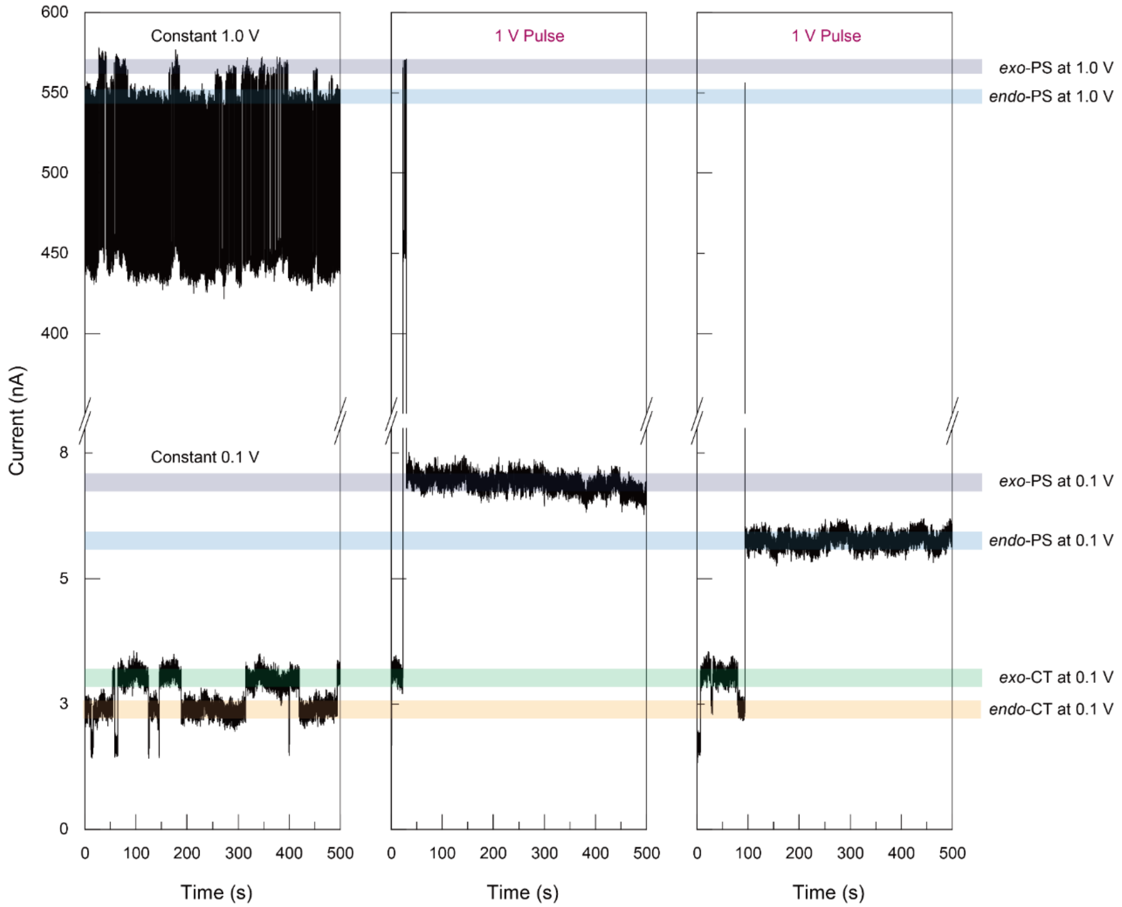

Fig. S107. Preparation and retention of *endo* and *exo* PSs on device 26#.

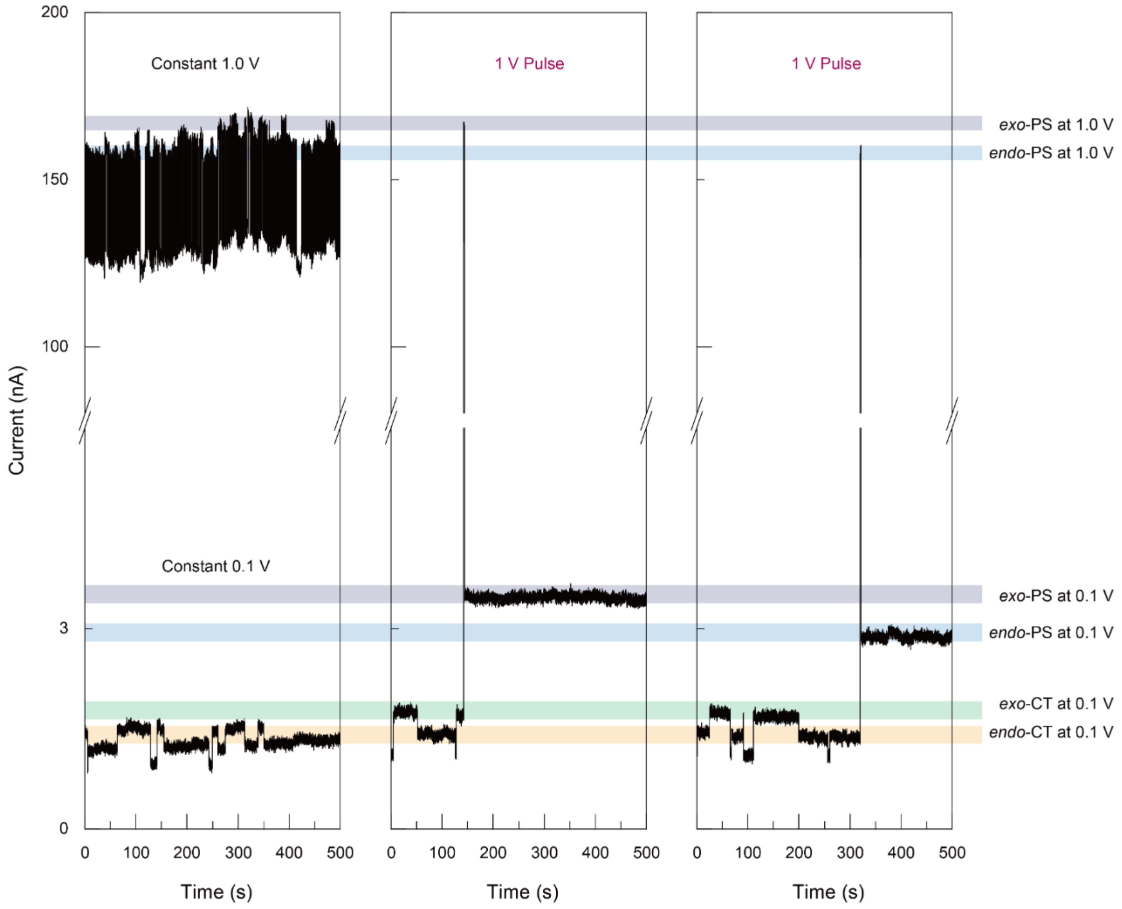

Fig. S108. Preparation and retention of *endo* and *exo* PSs on device 27#.

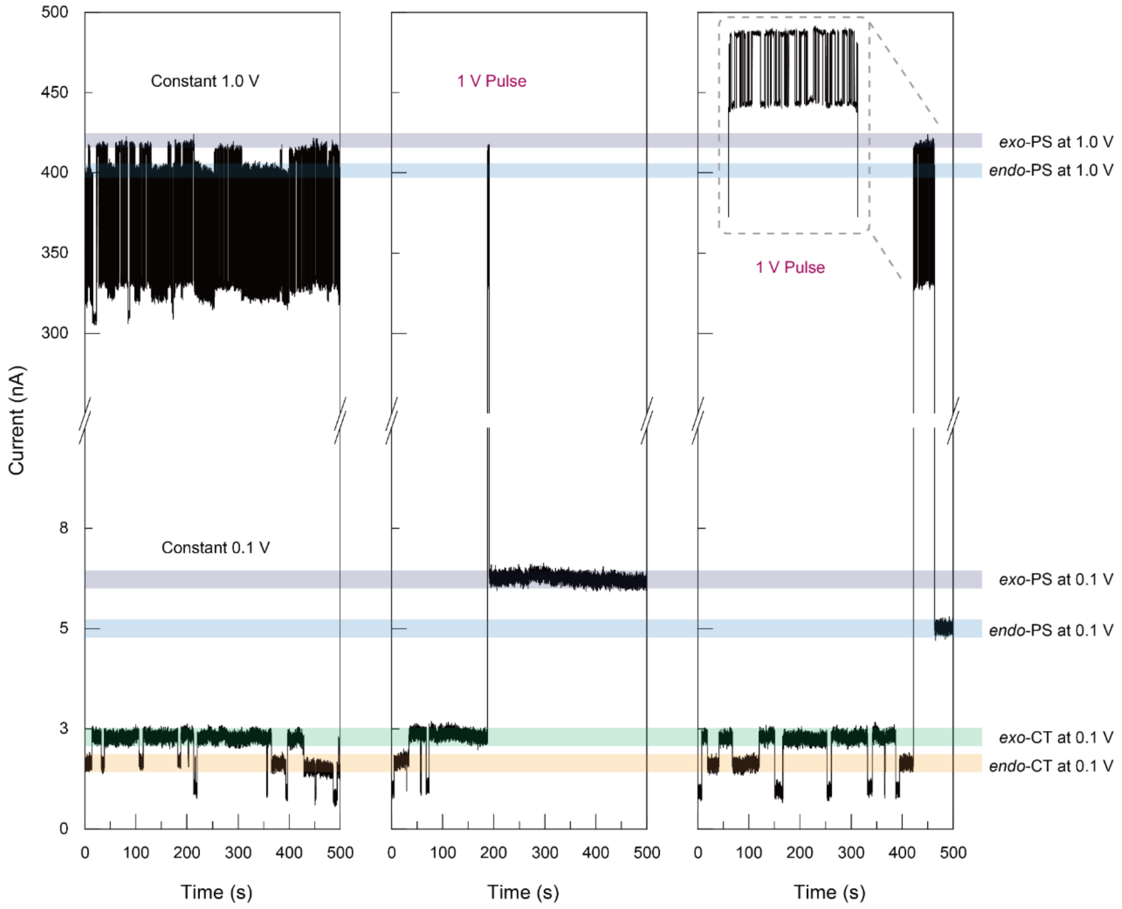

Fig. S109. Preparation and retention of *endo* and *exo* PSs on device 28#.

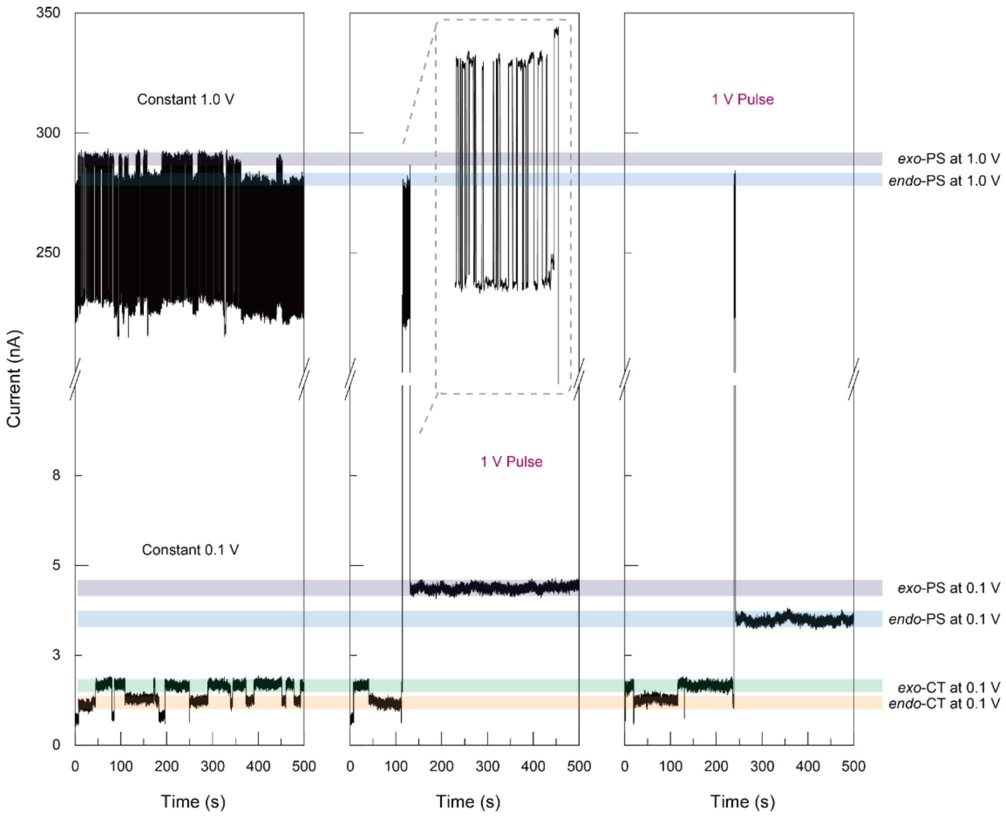

Fig. S110. Preparation and retention of *endo* and *exo* PSs on device 29#.

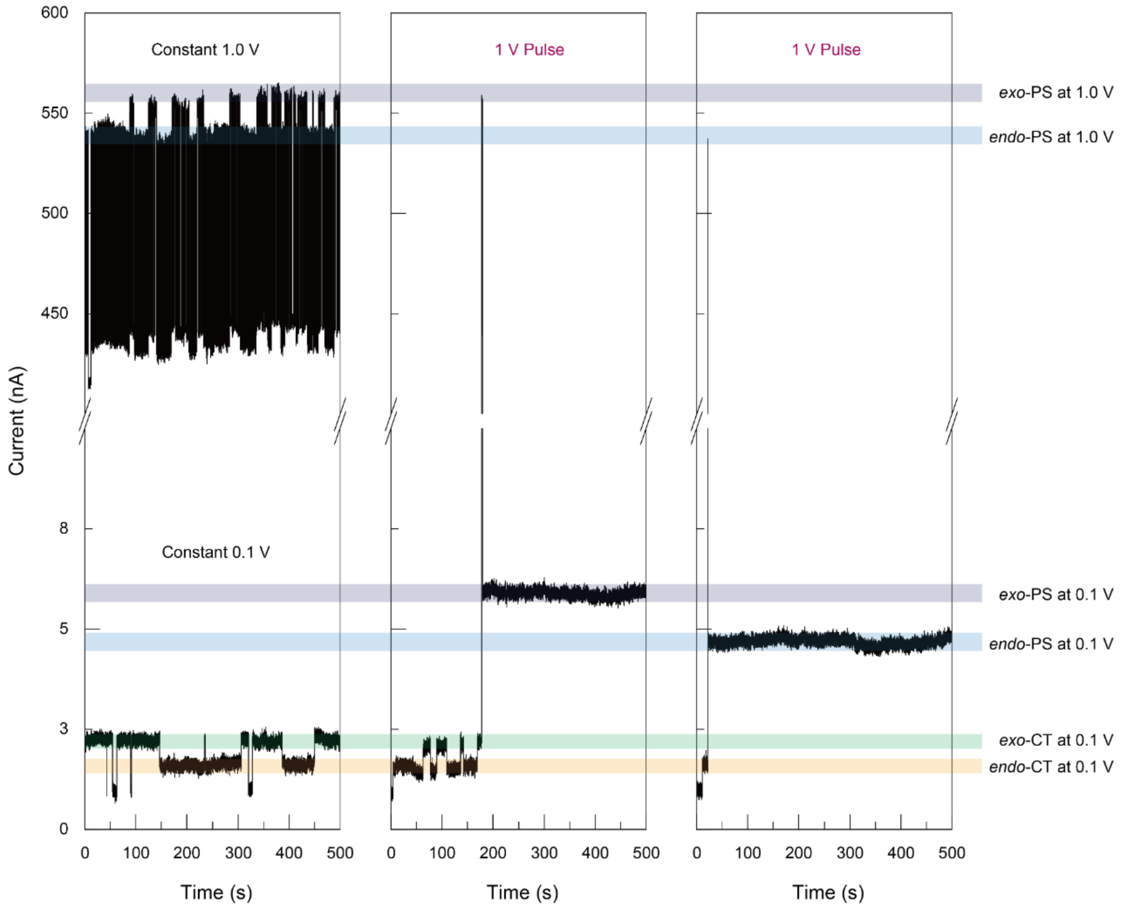

Fig. S111. Preparation and retention of *endo* and *exo* PSs on device 30#.

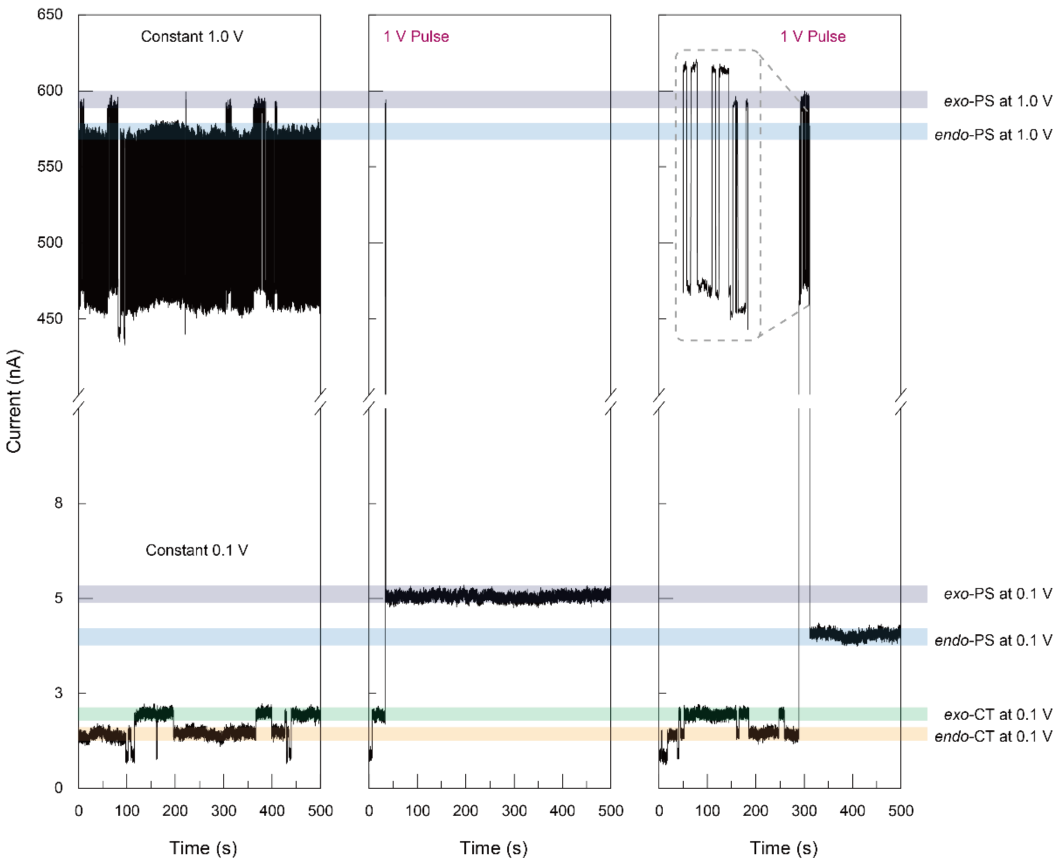

Fig. S112. Preparation and retention of *endo* and *exo* PSs on device 31#.

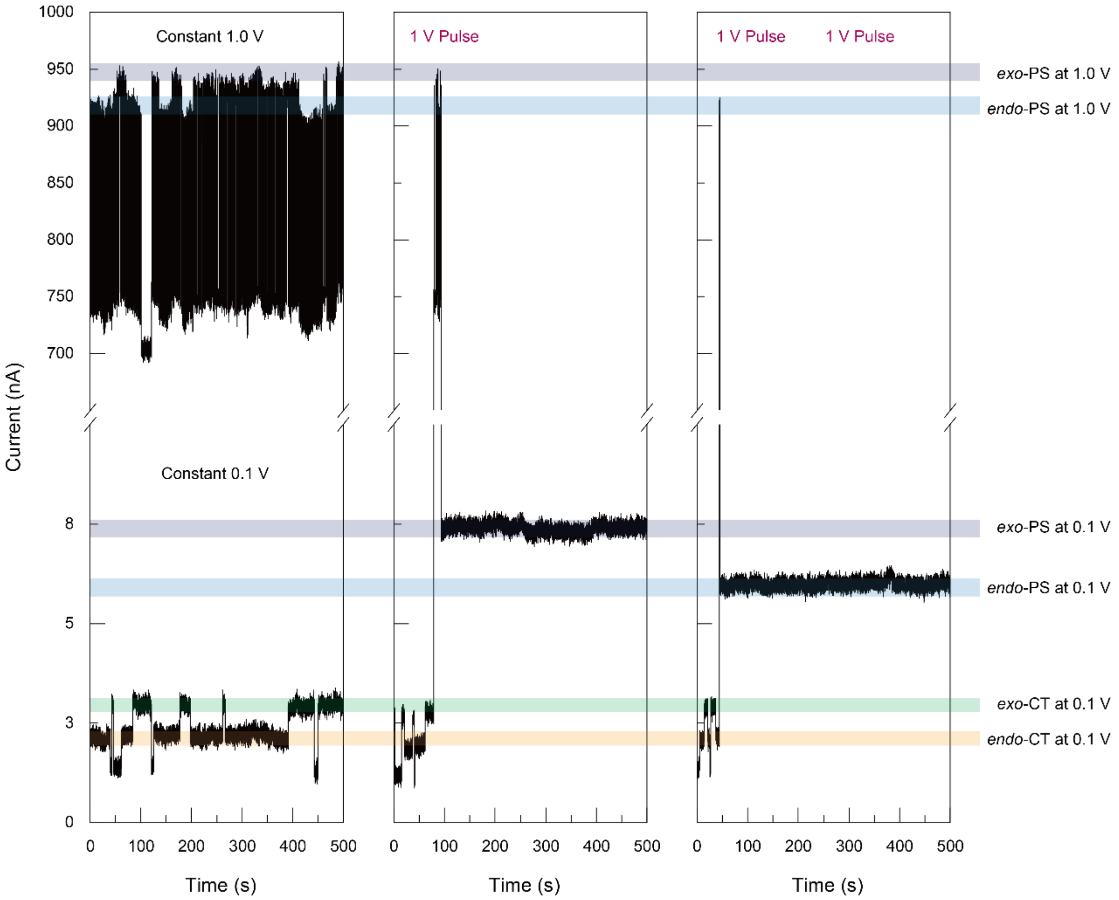

Fig. S113. Preparation and retention of *endo* and *exo* PSs on device 32#.

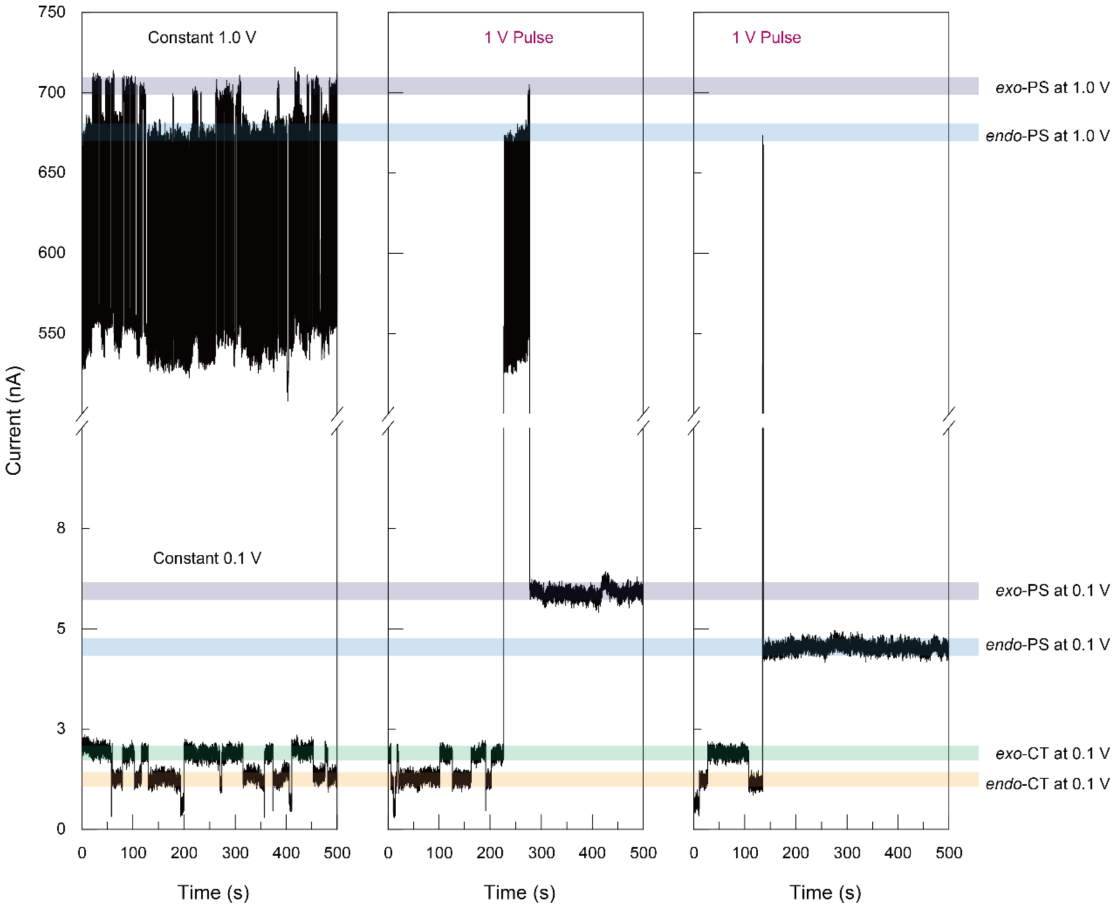

Fig. S114. Preparation and retention of *endo* and *exo* PSs on device 33#.

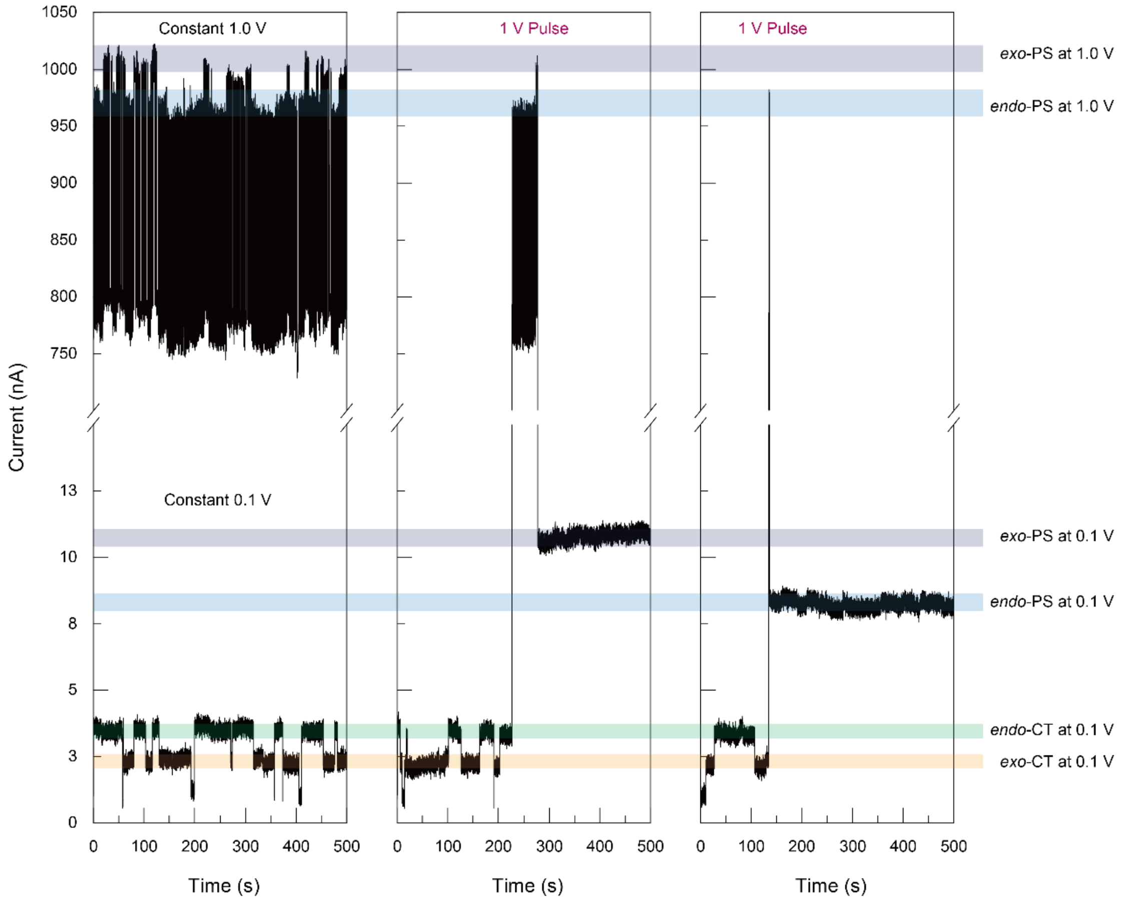

Fig. S115. Preparation and retention of *endo* and *exo* PSs on device 34#.

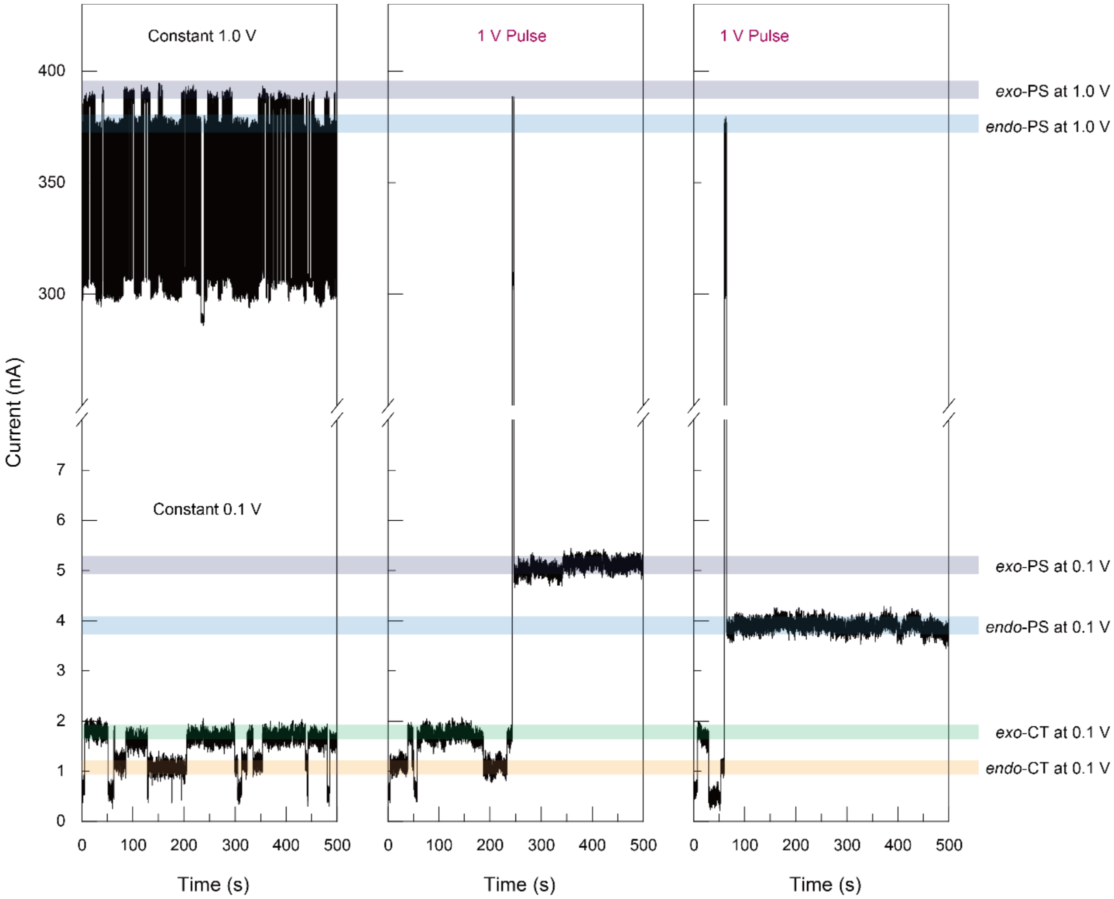

Fig. S116. Preparation and retention of *endo* and *exo* PSs on device 35#.

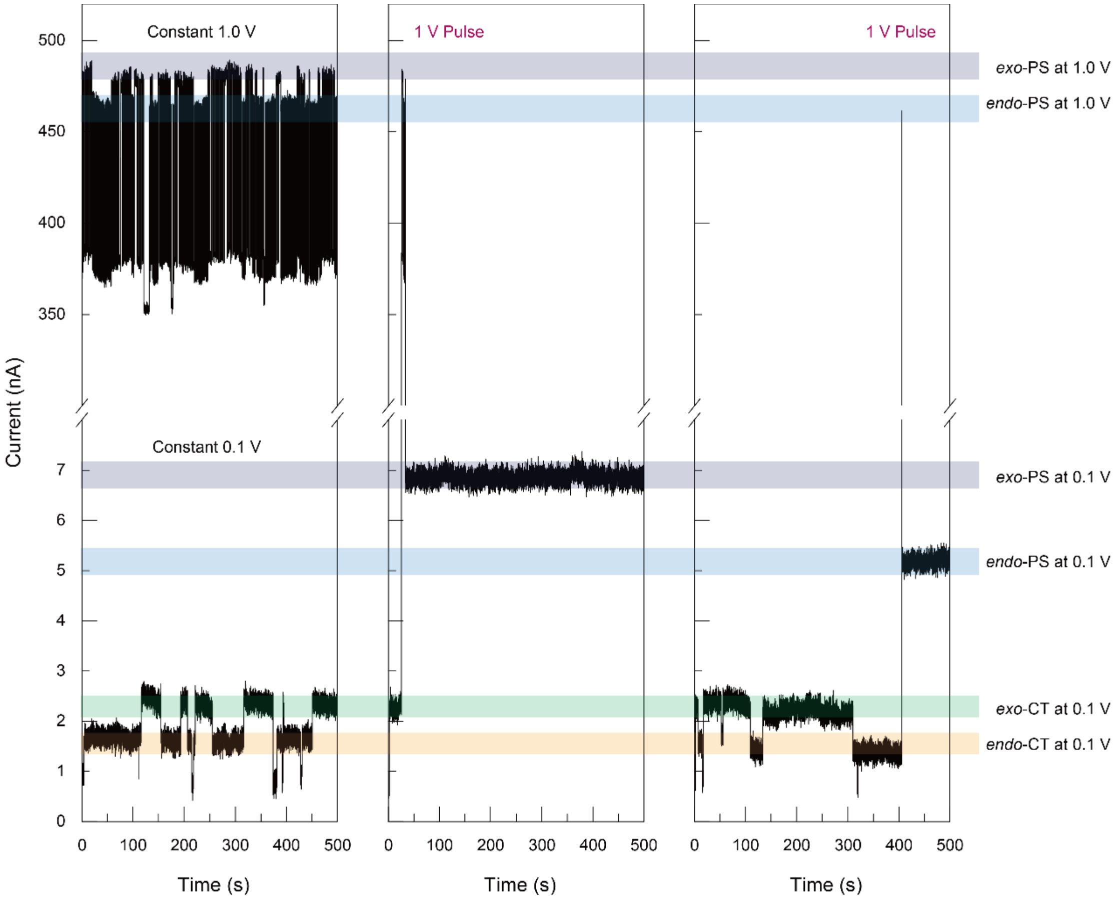

Fig. S117. Preparation and retention of *endo* and *exo* PSs on device 36#.

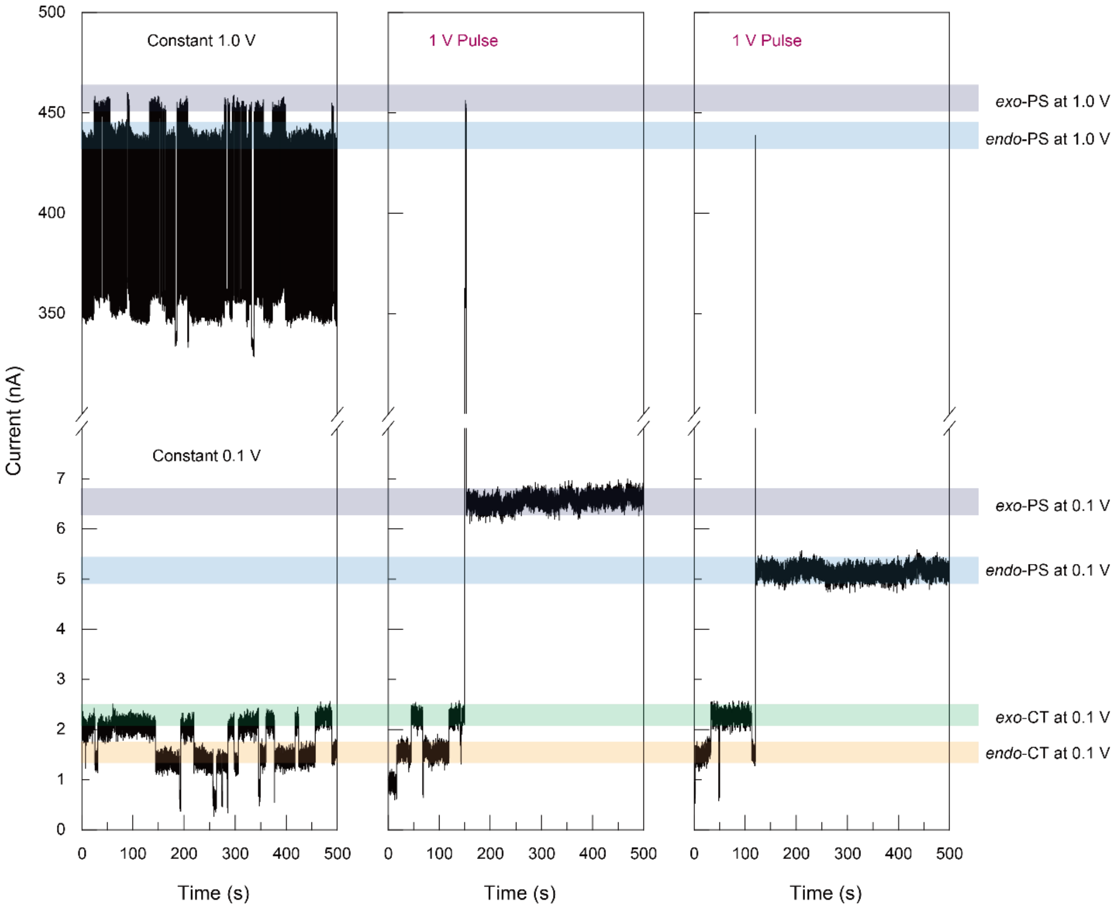

Fig. S118. Preparation and retention of *endo* and *exo* PSs on device 37#.

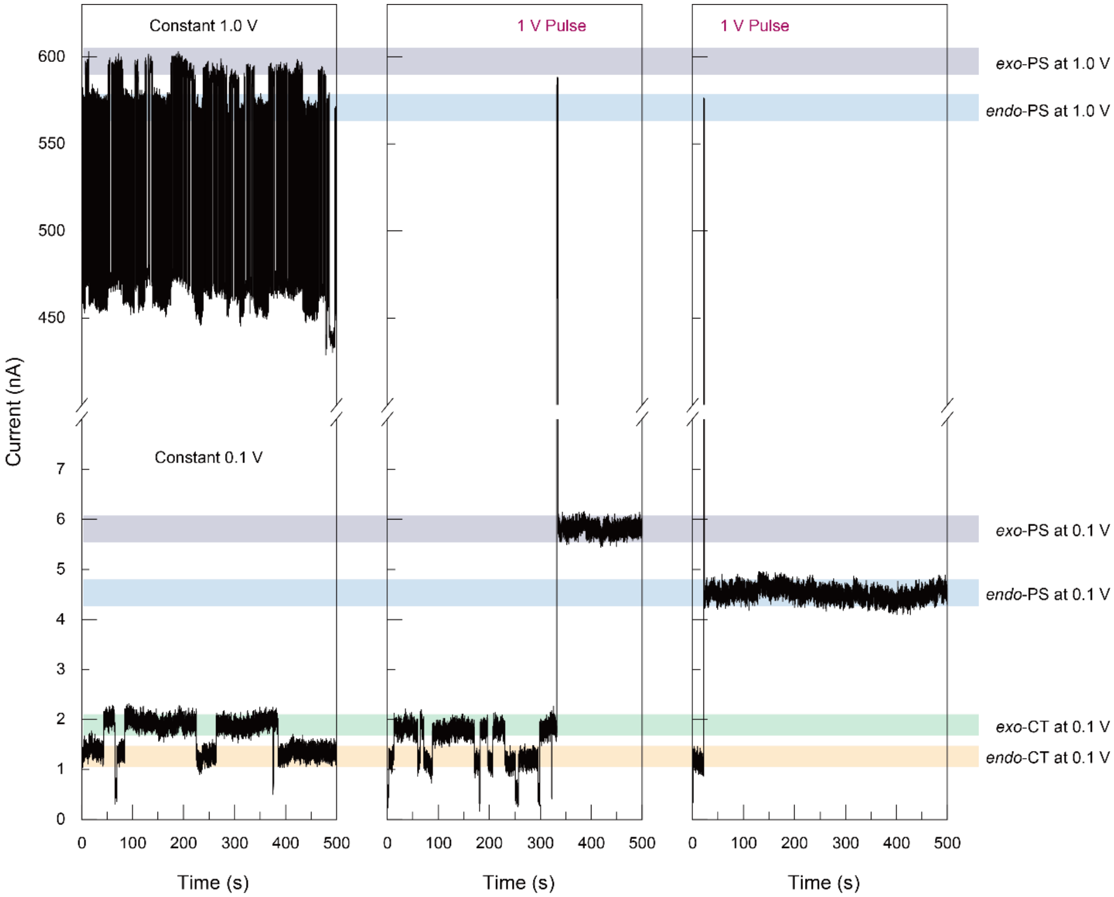

Fig. S119. Preparation and retention of *endo* and *exo* PSs on device 38#.

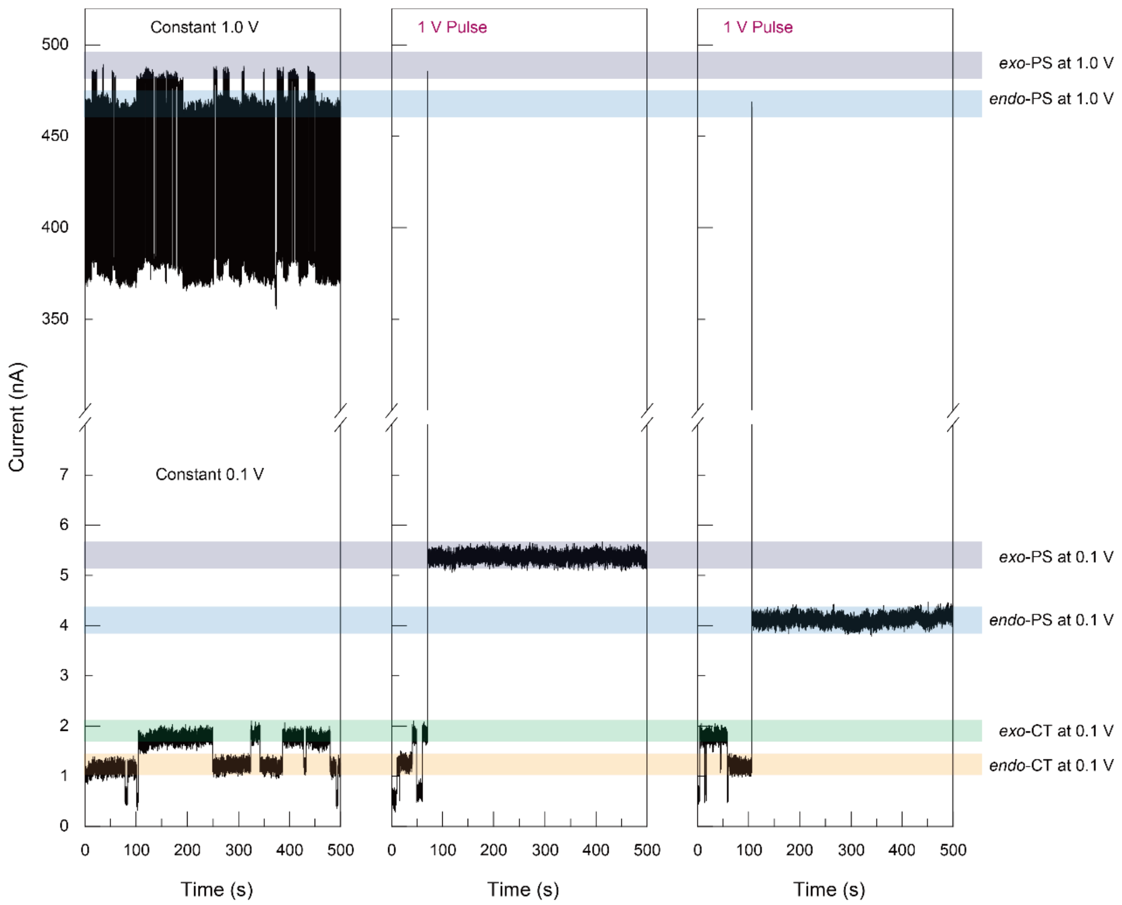

Fig. S120. Preparation and retention of *endo* and *exo* PSs on device 39#.

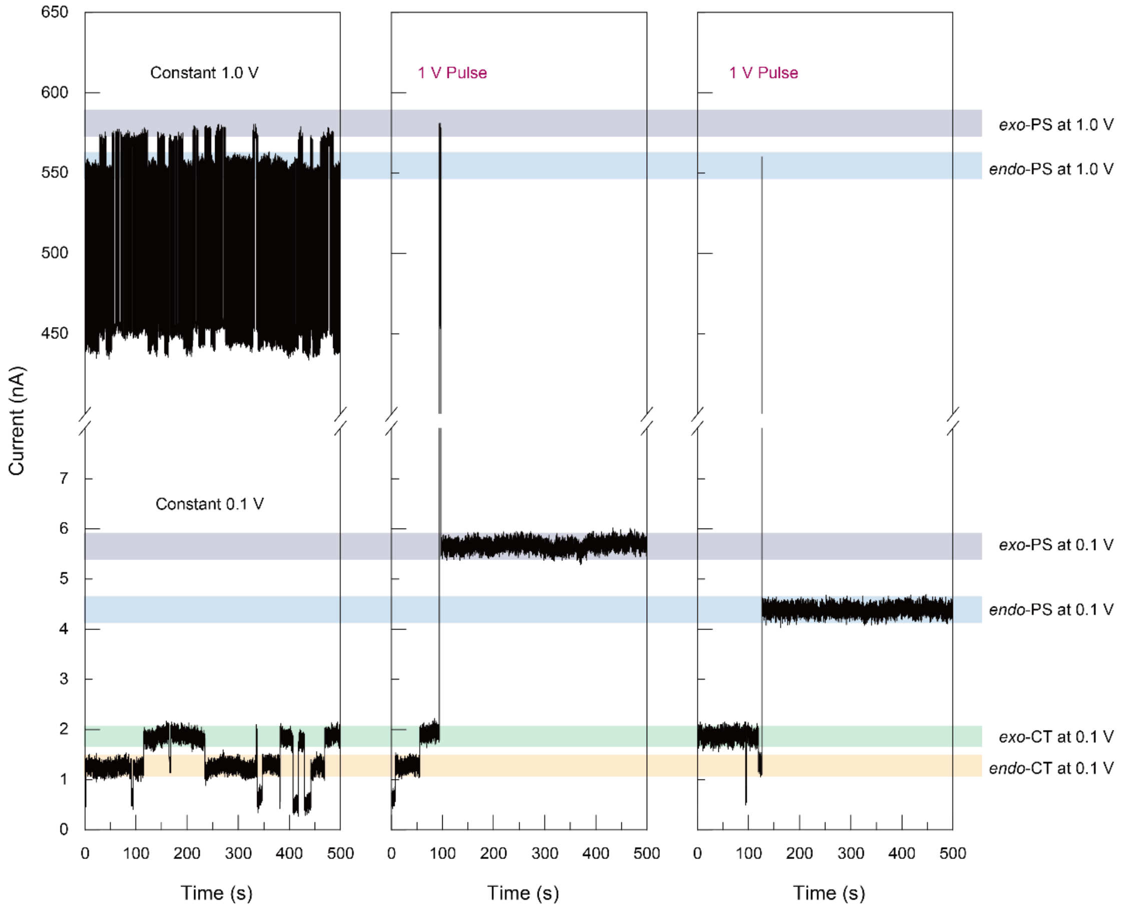

Fig. S121. Preparation and retention of *endo* and *exo* PSs on device 40#.

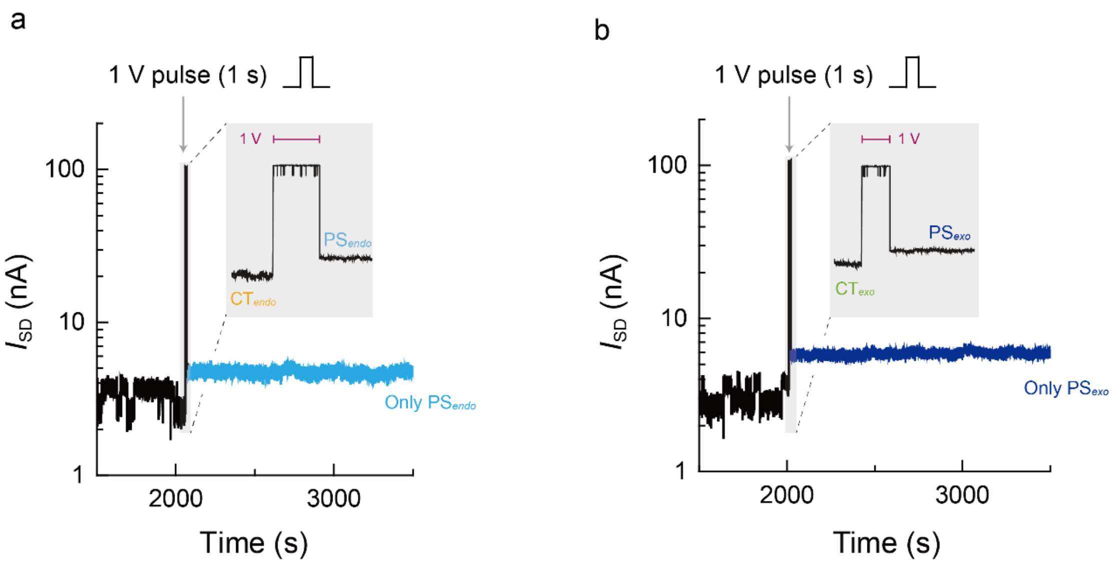

Fig. S122. On-line asymmetric synthesis of substituted *endo*- and *exo*-products. A. *I*–*t* curves of the reaction between 3-methoxyfuran and maleimide, where a 1 V pulse (gray area, and enlarged in the inset) was applied to the *endo*-CT state for 1 s. The switching between *endo*-CT and *endo*-PS was observed at a 1 V plateau. A higher occupancy of PS indicates a thermodynamic preference. **B.** *I*–*t* curves of the reaction between 3-methoxyfuran and maleimide, where a 1 V pulse (gray area, and enlarged in the inset) was applied to the *exo*-CT state for 1 s. The switching between *exo*-CT and *exo*-PS was observed at a 1 V plateau. A higher occupancy of PS indicates a thermodynamic preference.

22. On-line precise regulation of the regio-selectivity of Suzuki-Miyaura cross-coupling.

Suzuki-Miyaura cross-coupling between dihaloarenes and phenylboronic acids was studied here (Fig. S123), where the regio-selectivity of the product depends on the mode of oxidative addition. According to our on-line dynamic asymmetric synthesis strategy proposed in the main text, the screening of oxidative addition intermediates is possible to control the reaction pathway based on real-time monitoring of the catalytic cycle. Specifically, we anchored a previously studied Pd catalyst molecular bridge between graphene electrodes with amide bonds[3]. The pre-activated Pd catalyst could be used to study Suzuki-Miyaura cross-coupling. According to previous studies, Pd(0), oxidative addition & ligand exchange intermediate, *pre*-transmetalation intermediate, and transmetalation intermediate can be accurately detected. Therefore, precise regulation of two oxidative addition intermediates affords the ability to control the reaction pathways. In previous studies[4], we found that the gate voltage can effectively control the valence state of the catalyst, thereby tuning intermediates. Here, we achieved asymmetric synthesis by applying a positive back-gate voltage to reduce the unwanted oxidative addition intermediate while retaining the desired intermediate. During the experiments, we observed two catalytic cycles through synchronous characterization of electric and optical signals (Fig. S124). Referring to the previous assignment of conductance states in the catalytic cycle (intermediate control experiments and IETS), the idealized current curve is provided in Fig. S125. Then, in order to synthesize only product A (i.e., only catalytic cycle 1 exists), we apply a +5 V back-gate voltage when the catalyst enters cycle 2 (Fig. S126, at the corresponding oxidative addition intermediate), so that the catalyst returns to Pd(0). When the catalyst enters cycle 1, no operation is conducted, producing product A. In order to only synthesize product B (i.e., only catalytic cycle 2 exists), we apply a +5 V back gate voltage when the catalyst enters cycle 1 (Fig. S127, at the corresponding oxidation addition intermediate), so that the catalyst returns to Pd(0). Similarly, no operation is performed when the catalyst enters cycle 2, producing product B. Multiple operations could only synthesize product A or B alone. The comparison of the single-molecule resolved fluorescent spectra with the spectra of macroscopic standard samples (Figs. S128 and 129) proves the smooth proceeding of precise single-molecule asymmetric catalysis.

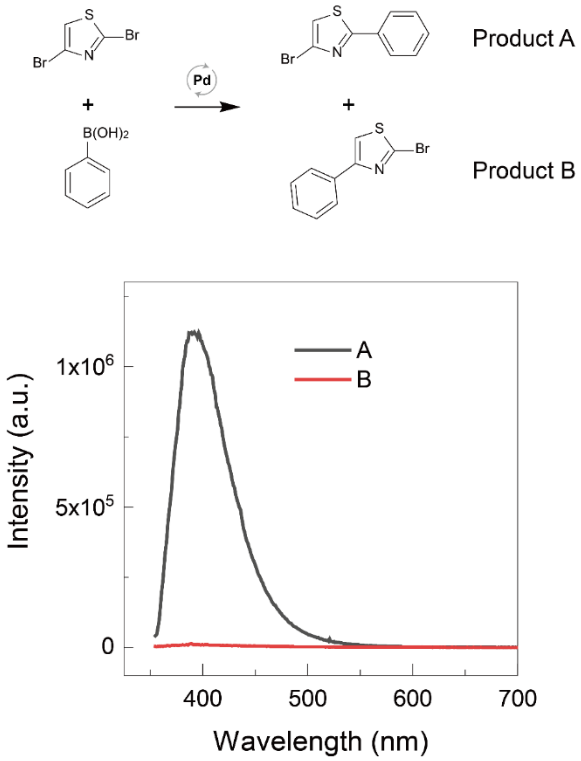

Fig. S123. Suzuki-Miyaura cross-coupling with regio-selectivity. Top panel: Schematic of the Suzuki-Miyaura cross-coupling studied here. Bottom panel: the macroscopic fluorescent spectra of the standard product samples.

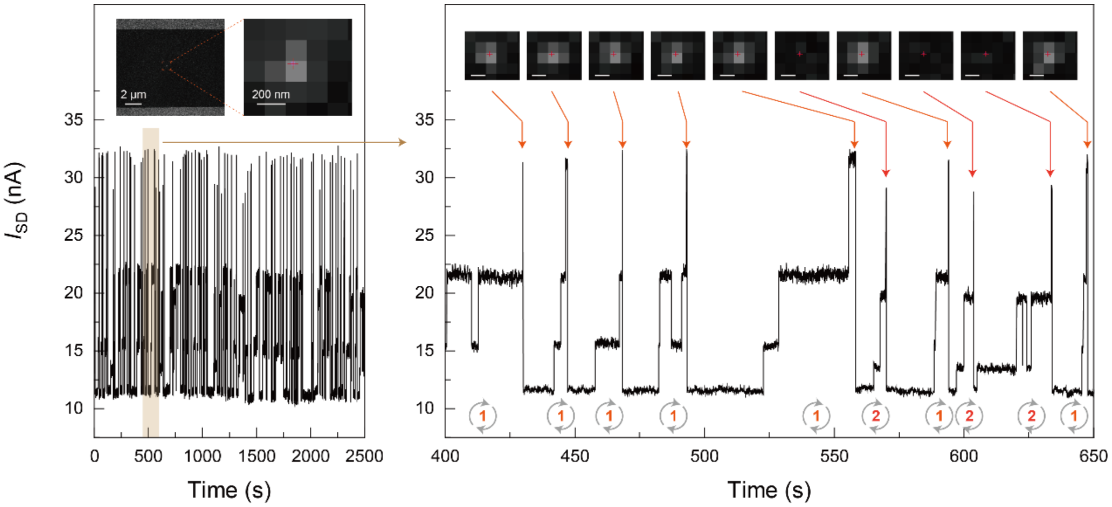

Fig. S124. Synchronous characterization of electric and optical signals of single-molecule Suzuki-Miyaura cross-coupling. The 300-mV bias voltage was applied to measure the *I*−*t* curves (at the bottom left panel and enlarged at the bottom right panel). Due to the multiple catalytic cycles with the formation of fluorescent products (blinking at the single-molecule catalyst site), the single molecule could be located precisely according to the stochastic optical reconstruction, which was provided at the top left panel. Simultaneous collection of the optical images shows a strong fluorescent product (A) (light pixel) was obtained after cycle 1, while a weak fluorescent product (B) (dark pixel) was obtained after cycle 2. The synchronous characterization provides an assignment of the two catalytic cycles.

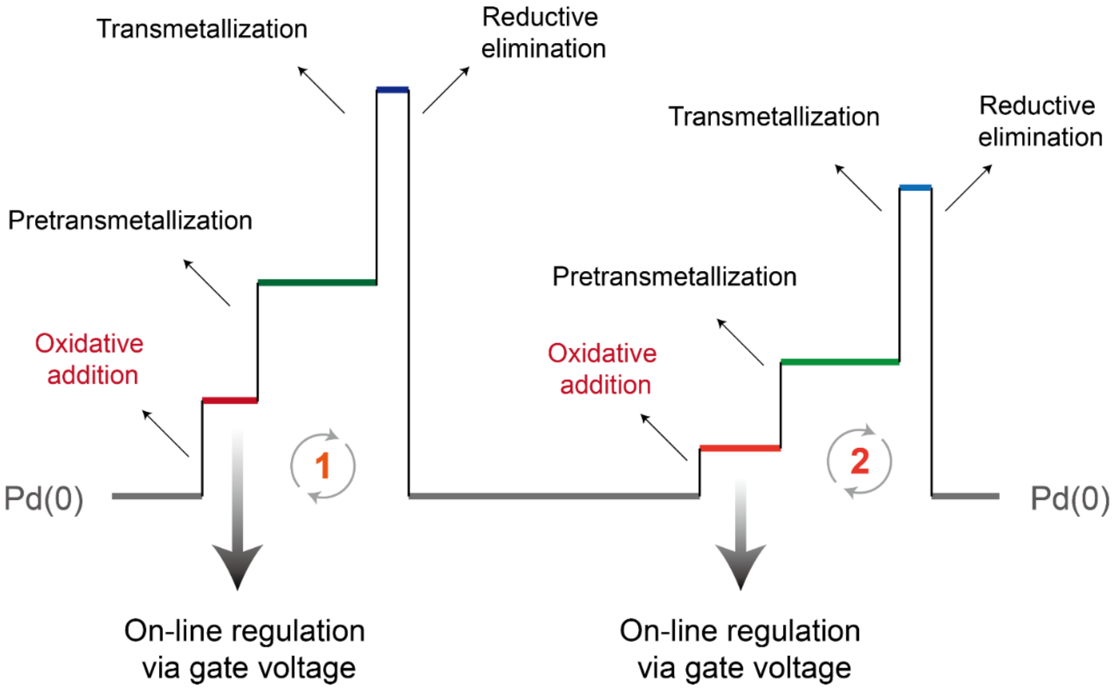

Fig. S125. Idealization of the above *I*−*t* curves and the strategy of controlling the regio-selectivity.

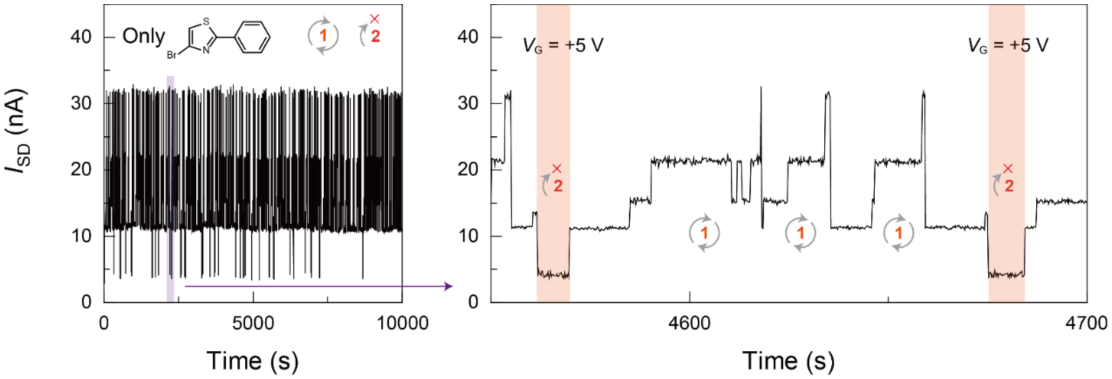

Fig. S126. On-line precise asymmetric synthesis of Product A. We applied a +5 V back-gate voltage when the catalyst entered cycle 2 (at the corresponding oxidative addition intermediate), so that the catalyst returned to Pd(0). When the catalyst entered cycle 1, no operation was conducted.

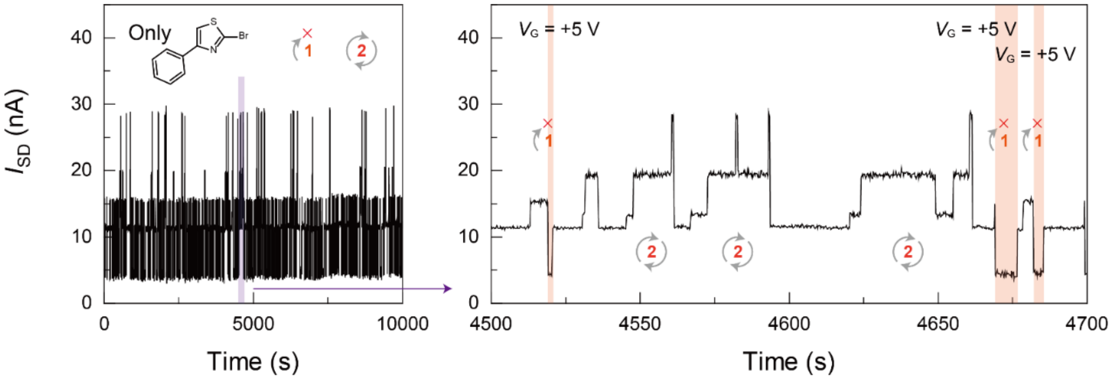

Fig. S127. On-line precise asymmetric synthesis of Product B. We applied a +5 V back-gate voltage when the catalyst entered cycle 1 (at the corresponding oxidative addition intermediate), so that the catalyst returned to Pd(0). When the catalyst entered cycle 2, no operation was conducted.

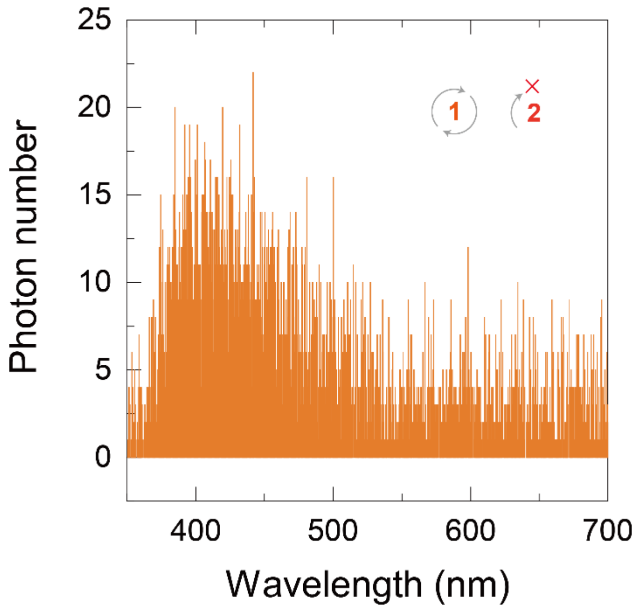

Fig. S128. Energy-resolved single-photon counting after on-line synthesis of product A. With a 60-s exposure time, the fluorescent spectroscopy of the synthesized product A (only catalytic cycle 1) was collected.

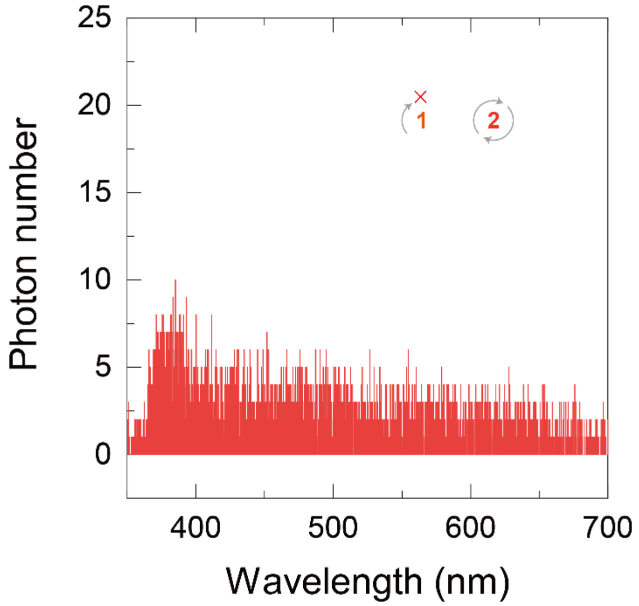

Fig. S129. Energy-resolved single-photon counting after on-line synthesis of products B. With a 60-s exposure time, the fluorescent spectroscopy of the synthesized product B (only catalytic cycle 2) was collected. The fluorescence intensity of the synthesized product B is much lower than that of the synthesized product A, consistent with the result in Fig. S123.

23. Synthesis of chiral 3-methylcyclohex-3-ene-1-carboxylic acids for ex-situ measurements

23.1 Materials and Methods

Except for the first step of the Diels-Alder reaction, which is carried out in a high-pressure resistant glass tube, all other reactions are conducted in Schlenk tubes. The reactions were stirred with Teflon-coated magnetic stirring bars. All the reagents were purchased from Shanghai Aladdin Biochemical Technology Co., Ltd. and Shanghai Macklin Biochemical Technology Co., Ltd, and were used as received without further purification. All solvents (hexanes, petroleum ether, ethyl acetate, dichloromethane, dry dichloromethane, tetrahydrofuran, methanol) were purchased from Shanghai Titan Scientific Co., Ltd. and Meryer (Shanghai) Biochemical Technology Co., Ltd. TLC analysis of reaction mixtures was performed on huanghai silica gel 60 F254 TLC plates and visualized by UV, I_2/silica stain. Reactions that require heating are using a silicon oil bath, which were controlled by an electronic temperature modulator from GLB. Flash chromatography was carried out with silica gel 200–300 mesh. 3-Nitrobenzyl (S)-3-methylcyclohex-3-ene-1-carboxylate and 3-nitrobenzyl (R)-3-methylcyclohex-3-ene-1-carboxylate were separated on YMC K-Prep LAB100G HPLC workstation (Daicel chiral columns CHIRALPAK® IF (0.46 cm I.D. × 15 cm L)). ^{1}H and ^{13}C NMR spectra were recorded with Bruker AV-400 spectrometers and were referenced to residual ^{1}H and ^{13}C signals of the deuterated solvents respectively (δ H 7.26, δ C 77.10 for chloroform). Abbreviations are as follows: s (singlet), d (doublet), t (triplet), m (multiplet), br (broad). HR-MS was performed on an Agilent 6545-QTOF instrumental. Optical rotation was determined on an IP-digi 300/2 apparatus.

23.2 Synthetic procedures of (S)- and (R)-3-methylcyclohex-3-ene-1-carboxylic acid

3- and 4-methyl-3-cyclohexenecarboxylic acid[5].

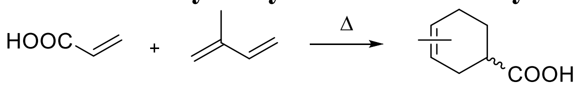

A sealed tube containing isoprene (4.5 g, 0.066 mol) and acrylic acid (4.8 g, 0.066 mol) was heated to 120℃ for 8 hr. After cooling down the mixture to room temperature, the unreacted reactants were evaporated under reduced pressure to afford the crude product that can be directly proceeded to the next step without further treatment.

3- and 4-nitrobenzyl 4-methylcyclohex-3-ene-1-carboxylate.

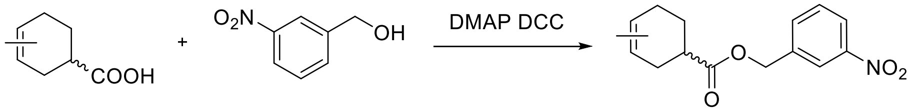

To a mixture of (3-nitrophenyl)methanol (0.57 g, 3.7 mmol), DMAP (0.086 g, 0.7 mmol), and the crude acid (0.516 g, 3.7 mmol) in dry dichloromethane (10 mL) was added DCC (0.92 g, 4.5 mmol). The solution was then stirred at room temperature for 4–5 h until thin layer chromatography (TLC) indicated that no alcohol remained. The suspension was then filtered. The filtrate was diluted with 50 mL of hexanes, filtered, and then concentrated. The residue was then purified by using flash chromatography to get ester with 20:1 petroleum ether/ethyl acetate. The mixed ester was obtained as a colorless oily liquid (~0.98 g, ~96%). DMAP: 4-dimethylaminopyridine; DCC: N, N'-dicyclohexylcarbodiimide. ^{1}H NMR (400 MHz, Chloroform-d, ppm) δ 8.20 (s, 1H), 8.16 (d, J = 8.2 Hz, 1H), 7.66 (d, J = 4.5 Hz, 1H), 7.53 (t, J = 7.9 Hz, 1H), 5.37 (s, 1H), 5.20 (d, J = 2.9 Hz, 2H), 2.73–2.48 (m, 1H), 2.32–1.90 (m, 5H), 1.77–1.60 (m, 4H).

^{13}C NMR (101 MHz, Chloroform-d, ppm) δ 175.46, 175.36, 148.37, 138.45, 138.42, 133.80, 133.70, 133.67, 132.07, 129.58, 129.57, 123.03, 123.00, 122.60, 122.56, 120.66, 118.99, 64.59, 64.55, 39.72, 39.12, 32.02, 29.11, 27.61, 25.38, 24.86, 24.38, 23.51, 23.42. HR-MS (ESI): m/z = 276.1232 $[M+H]^+$, calcd. for $C_{15}H_{18}NO_4$: 276.1236.

3-Nitrobenzyl (S)-3-methylcyclohex-3-ene-1-carboxylate and 3-nitrobenzyl (R)-3-methylcyclohex-3-ene-1-carboxylate were separated through two steps. Firstly, separate 3- and 4-methyl-3-cyclohexenecarboxylic acid on YMC K-Prep LAB100G HPLC workstation (Daicel chiral columns CHIRALPAK® IF (0.46 cm I.D. × 15 cm L)) using 97/3 (v/v) n-Hexane /EtOH as elute in 25 °C. Secondly, separate 3-Nitrobenzyl (S)-3-methylcyclohex-3-ene-1-carboxylate and 3-nitrobenzyl (R)-3-methylcyclohex-3-ene-1-carboxylate within 3-methyl-3-cyclohexenecarboxylic acid on YMC K-Prep LAB100G HPLC workstation (Daicel chiral columns CHIRALPAK® IF (0.46 cm I.D. × 15 cm L)) using 97/3 (v/v) n-Hexane /EtOH as elute in 25 °C. 3-nitrobenzyl (S)-3-methylcyclohex-3-ene-1-carboxylate was obtained as a colorless oily liquid. [α]20 D = –28.227 (c. 0.548, Chloroform); ^1H NMR (400 MHz, Chloroform-d, ppm) δ 8.21 (s, 1H), 8.17 (d, J = 8.3 Hz, 1H), 7.67 (d, J = 7.6 Hz, 1H), 7.53 (t, J = 7.9 Hz, 1H), 5.38 (s, 1H), 5.21 (s, 2H), 2.72–2.60 (m, 1H), 2.27–1.92 (m, 5H), 1.72–1.59 (m, 4H). The peak at 3.73 ppm is attributed to the solvent ethanol remaining in the product. ^{13}C NMR (101 MHz, Chloroform-d, ppm) δ 175.47, 148.48, 138.46, 133.76, 132.15, 129.64, 123.13, 122.70, 120.73, 64.68, 39.81, 32.11, 24.94, 24.46, 23.60. ESI-QTOF, m/z: calcd, 275.1152, found:276.1232 ($[M+H]^+$). 3-Nitrobenzyl (R)-3-methylcyclohex-3-ene-1-carboxylate was obtained as a colorless oily liquid. [α]20 D =+33.029 (c. 0.335, Chloroform); ^1H NMR (400 MHz, Chloroform-d, ppm) δ 8.20 (s, 1H), 8.16 (d, J = 8.3 Hz, 1H), 7.67 (d, J = 7.5 Hz, 1H), 7.53 (t, J = 7.9 Hz, 1H), 5.38 (s, 1H), 5.21 (s, 2H), 2.71–2.60 (m, 1H), 2.27–1.92 (m, 5H), 1.70–1.58 (m, 4H). The peak at 3.72 ppm is attributed to the solvent ethanol remaining in the product. ^{13}C NMR (101 MHz, Chloroform-d, ppm) δ 175.48, 148.47, 138.46, 133.76, 132.15, 129.65, 123.13, 122.70, 120.73, 64.68, 39.81, 32.11, 24.94, 24.46, 23.60. HR-MS (ESI): m/z = 276.1230 $[M+H]^+$, calcd. for $C_{15}H_{18}NO_4$: 276.1236.

(S)-3-methylcyclohex-3-ene-1-carboxylic acid.

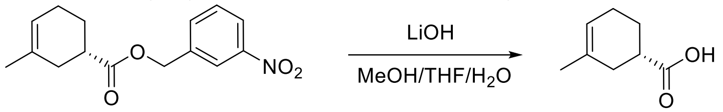

To 3-nitrobenzyl (S)-3-methylcyclohex-3-ene-1-carboxylate (80 mg, 0.29 mmol) in THF/MeOH/H_2O (1: 1: 2, 4.3 mL) at 0 °C was added LiOH (27.8 mg, 1.16 mmol), and the reaction mixture was stirred overnight at room temperature. The solvent was removed by rotary evaporation under reduced pressure, then H_2O (30 mL) was added to the reaction mixture. Then, the combined mixture was extracted with CH_2Cl_2 and the aqueous mixture was collected. The aqueous mixture was acidified to pH 2 with 1 N HCl, the residue was extracted with CH_2Cl_2 (3x30 mL) and washed with H_2O and brine, and the organic layer was dried over Na_2SO_4, filtered, and concentrated in vacuo. (S)-3-methylcyclohex-3-ene-1-carboxylic acid was obtained as a colorless oily liquid (~30 mg, ~74%). [α]20 D = –72.257 (c. 0.665, Chloroform); ^1H NMR (400 MHz, Chloroform-d, ppm) δ 11.48 (br, 1H), 5.38 (s, 1H), 2.60 (m, 1H), 2.26–1.93 (m, 5H), 1.72–1.57 (m, 4H). ^{13}C NMR (101 MHz, Chloroform-d, ppm) δ 182.56, 132.19, 120.73, 39.73, 31.91, 24.76, 24.49, 23.61. HR-MS (ESI): m/z = 139.0766 $[M-H]^+$, calcd. for $C_8H_{11}O_2$: 139.0759.

(R)-3-methylcyclohex-3-ene-1-carboxylic acid.

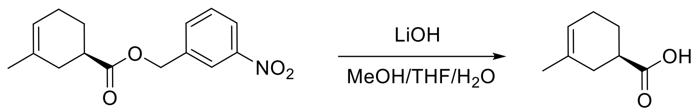

The synthesis method for (R)-3-methylcyclohex-3-ene-1-carboxylic acid is same as for (S)-3-methylcyclohex-3-ene-1-carboxylic acid. (R)-3-methylcyclohex-3-ene-1-carboxylic acid was obtained as a colorless oily liquid (~51 mg, ~78%). [α]20 D = +71.297 (c. 0.665, Chloroform); ^{1}H NMR (400 MHz, Chloroform-d, ppm) δ 11.74 (br, 1H), 5.39 (s, 1H), 2.60 (m, 1H), 2.27–1.95 (m, 5H), 1.72–1.57(m, 4H). ^{13}C NMR (101 MHz, Chloroform-d, ppm) δ 182.71, 132.18, 120.73, 39.74, 31.90, 24.75, 24.48, 23.60. HR-MS (ESI): m/z = 139.0764 [M-H]$^+$, calcd. for $C_8H_{11}O_2$: 139.0759.

**Structure**	**Test Conditions**	**Configuration**	**Specific rotation degrees mL/g·dm**	**Reference**
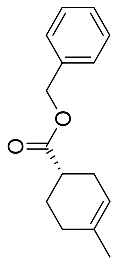	c: 6.0 g/100mL;<br>Solvent: Dichloromethane;<br>λ: 589.3 nm;<br>Temp: 23 °C	*S*	−59.7	6
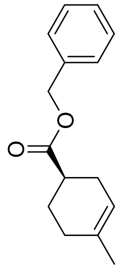	c: 2.5 g/100mL;<br>Solvent: Dichloromethane;<br>λ: 589.3 nm;<br>Temp: 23 °C	*R*	+60.7	6
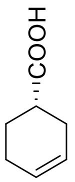	c: 1.00 g/100mL;<br>Solvent: Chloroform ;<br>λ: 589.3 nm;<br>Temp: 25 °C	*S*	−104	7

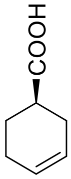	c: 1.00 g/100mL;<br>Solvent: Chloroform ;<br>λ: 589.3 nm;<br>Temp: 26 °C	*R*	+89.6	8
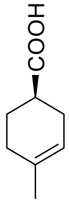	c: 2.22 g/100mL;<br>Solvent: Dichloromethane;<br>λ: 589.3 nm;<br>Temp: 25 °C	*R*	+71.2	9
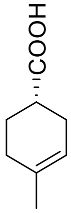	c: 4.00 g/100mL;<br>Solvent: Ethanol;<br>Temp: 20 °C	*S*	−107	10
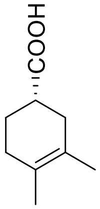	c: 1.8 g/100mL;<br>Solvent: Ethanol;<br>λ: 589.3 nm;<br>Temp: 20 °C	*S*	−85	11
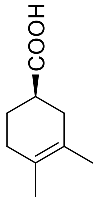	c: 1.5 g/100mL;<br>Solvent: Ethanol;<br>λ: 589.3 nm;<br>Temp: 25 °C	*R*	+77	12
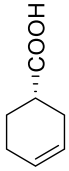	c: 0.525 g/100mL;<br>Solvent: Chloroform;<br>λ: 589.44 nm;<br>Temp: 20 °C	*S*	−89.642	

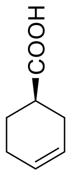	c: 0.908 g/100mL; Solvent: Chloroform; λ: 589.44 nm; Temp: 20 °C	*R*	+92.249
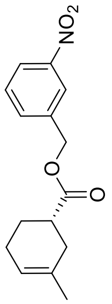	c: 0.548 g/100mL; Solvent: Chloroform; λ: 589.44 nm; Temp: 20 °C	*S*	−28.227
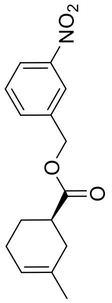	c: 0.335g/100mL; Solvent: Chloroform; λ: 589.44 nm; Temp: 20 °C	*R*	+33.029
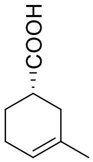	c: 0.665g/100mL; Solvent: Chloroform; λ: 589.44 nm; Temp: 20 °C	*S*	−72.257
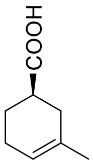	c: 0.665g/100mL; Solvent: Chloroform; λ: 589.44 nm; Temp: 20 °C	*R*	+71.297

23.3 NMR Spectra and HPLC

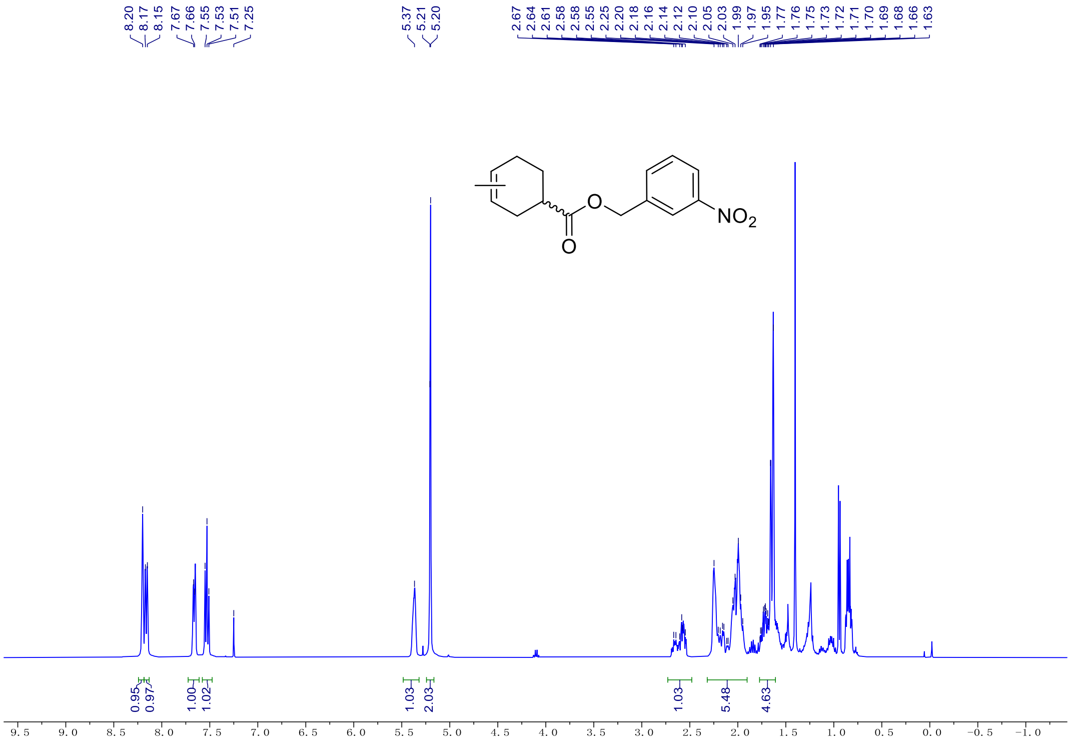
8.20
8.17
8.15
7.67
7.66
7.55
7.53
7.51
7.25
5.37
5.21
5.20
2.67
2.64
2.61
2.58
2.58
2.55
2.25
2.20
2.18
2.16
2.14
2.12
2.10
2.05
2.03
1.99
1.97
1.95
1.77
1.76
1.75
1.73
1.72
1.71
1.70
1.69
1.68
1.66
1.63
NO_2
O
O
0.95
0.97
1.00
1.02
1.03
2.03
1.03
5.48
4.63

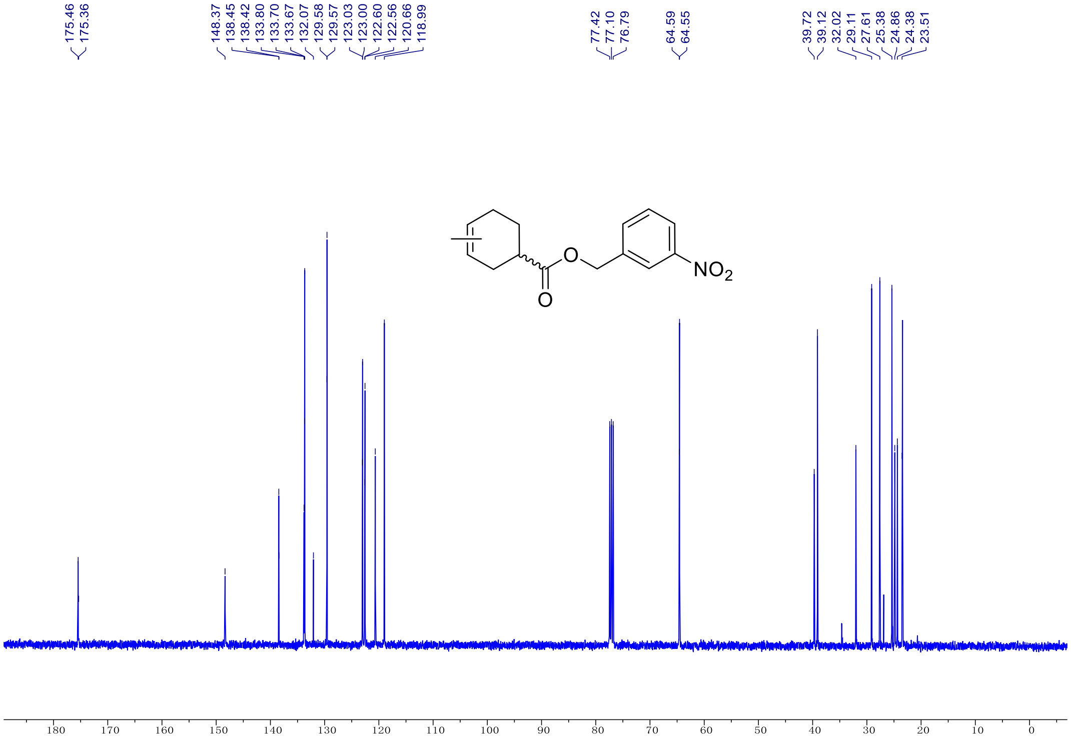
175.46
175.36
148.37
138.45
138.42
133.80
133.70
133.67
132.07
129.58
129.57
123.03
123.00
122.60
122.56
120.66
118.99
77.42
77.10
76.79
64.59
64.55
39.72
39.12
32.02
29.11
27.61
25.38
24.86
24.38
23.51
NO_2
O
O

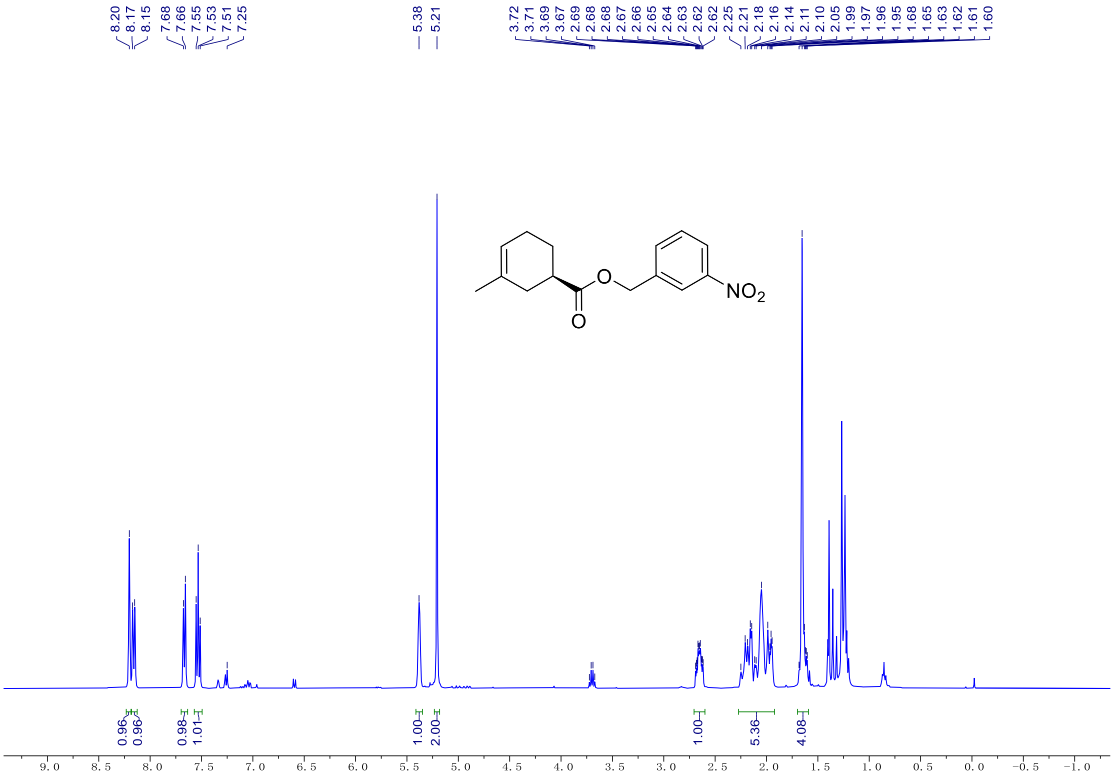

8.20
8.17
8.15
7.68
7.66
7.55
7.53
7.51
7.25
5.38
5.21
3.72
3.71
3.69
3.67
2.69
2.68
2.67
2.66
2.65
2.64
2.63
2.62
2.62
2.25
2.21
2.18
2.16
2.14
2.11
2.10
2.05
1.99
1.97
1.96
1.95
1.68
1.65
1.63
1.62
1.61
1.60
NO2
O
O
0.96
0.96
0.98
1.01
1.00
2.00
1.00
5.36
4.08
9.0 8.5 8.0 7.5 7.0 6.5 6.0 5.5 5.0 4.5 4.0 3.5 3.0 2.5 2.0 1.5 1.0 0.5 0.0 -0.5 -1.0

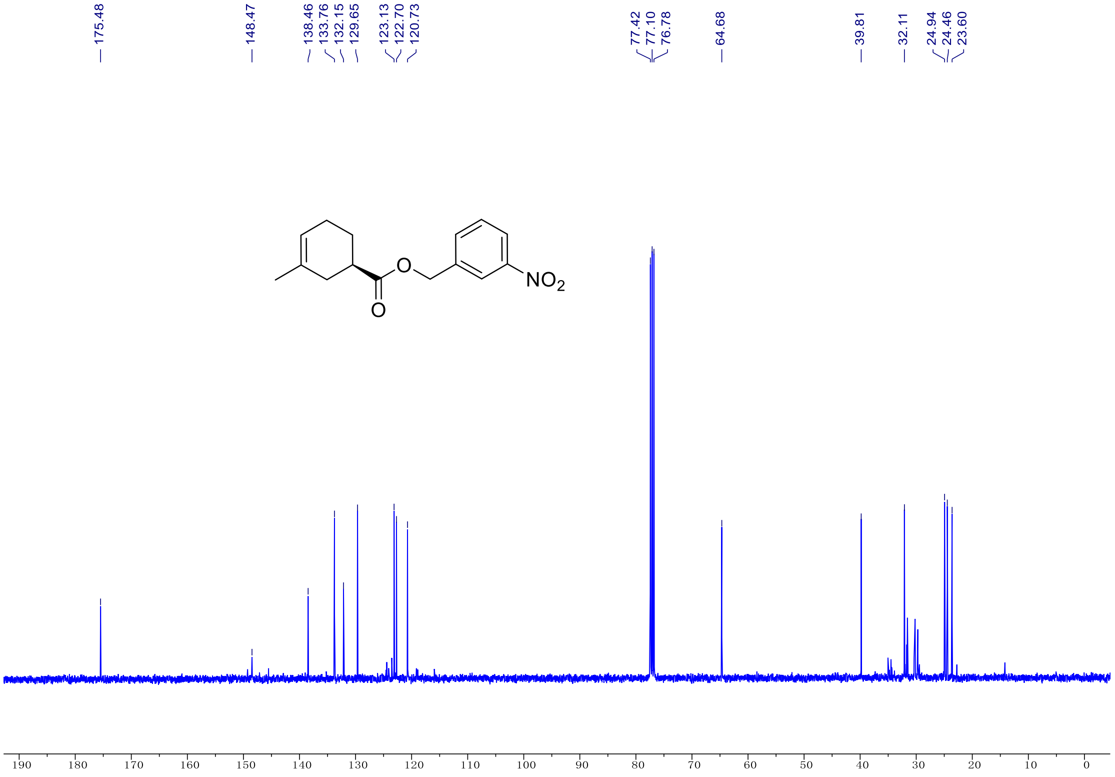

175.48
148.47
138.46
133.76
132.15
129.65
123.13
122.70
120.73
77.42
77.10
76.78
64.68
39.81
32.11
24.94
24.46
23.60
NO2
O
O
190 180 170 160 150 140 130 120 110 100 90 80 70 60 50 40 30 20 10 0

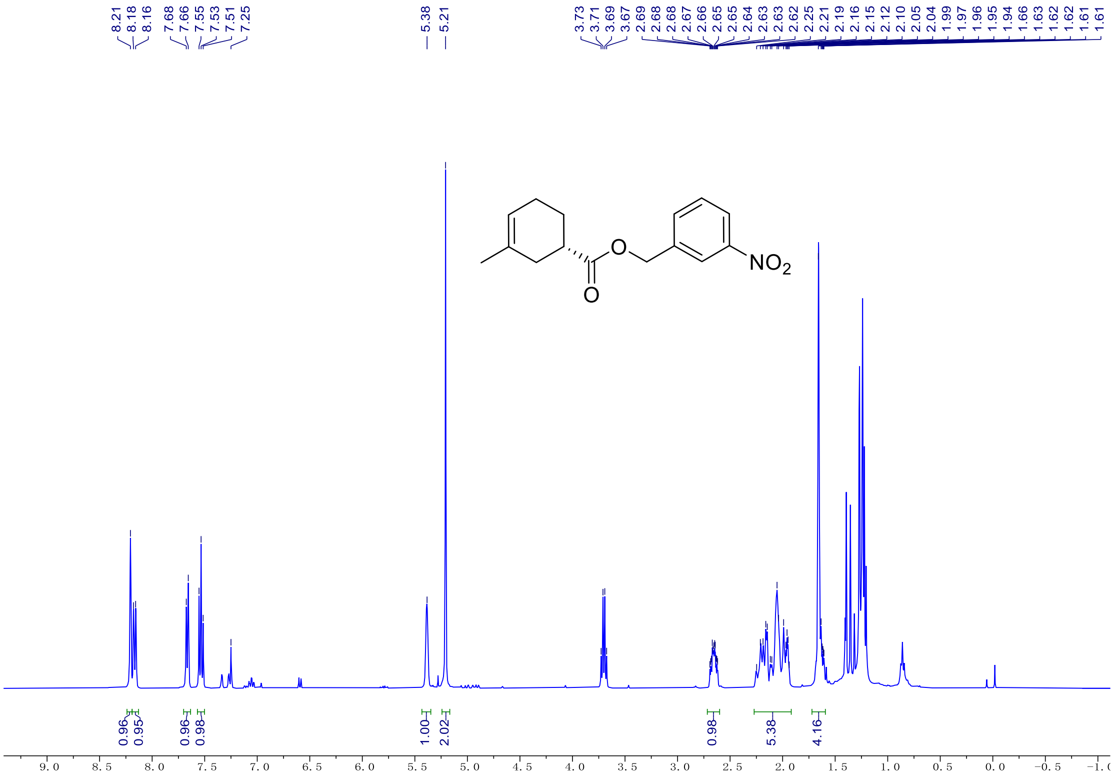
8.21
8.18
8.16
7.68
7.66
7.55
7.53
7.51
7.25
5.38
5.21
3.73
3.71
3.69
3.67
2.69
2.68
2.68
2.67
2.66
2.65
2.65
2.64
2.63
2.63
2.62
2.25
2.21
2.19
2.16
2.15
2.12
2.10
2.05
2.04
1.99
1.97
1.96
1.95
1.94
1.66
1.63
1.62
1.61
1.61
NO2
O
O
0.96
0.95
0.96
0.98
1.00
2.02
0.98
5.38
4.16

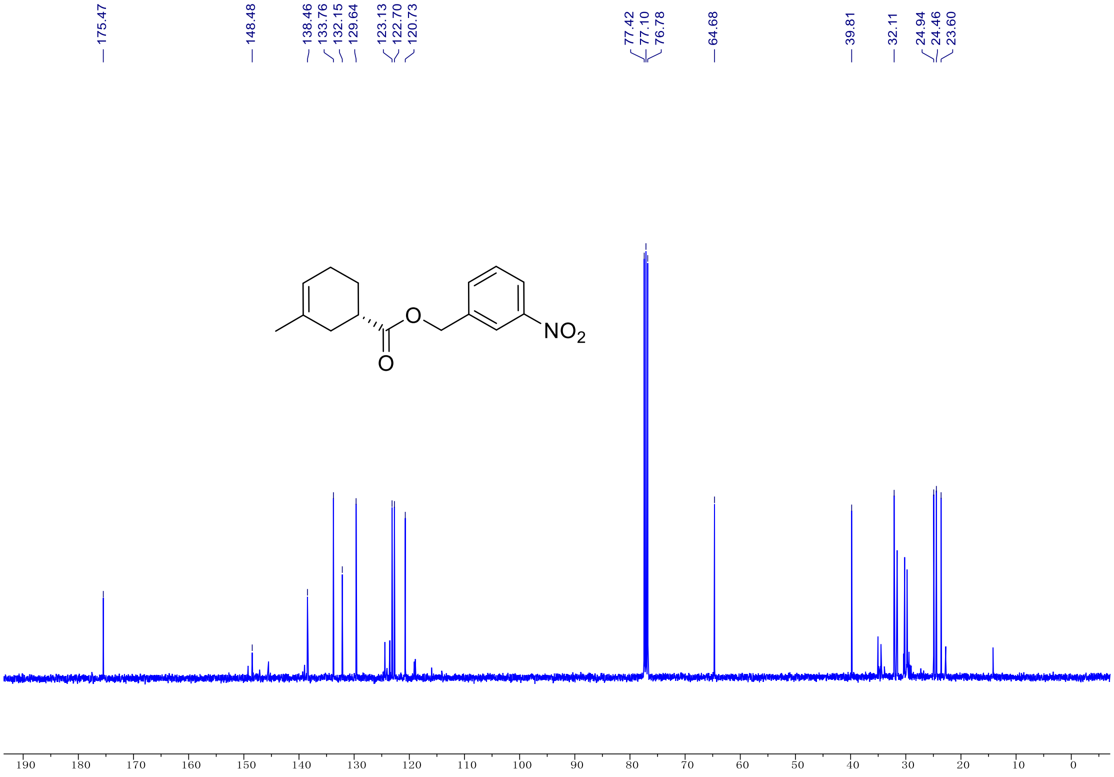
175.47
148.48
138.46
133.76
132.15
129.64
123.13
122.70
120.73
77.42
77.10
76.78
64.68
39.81
32.11
24.94
24.46
23.60
NO2
O
O

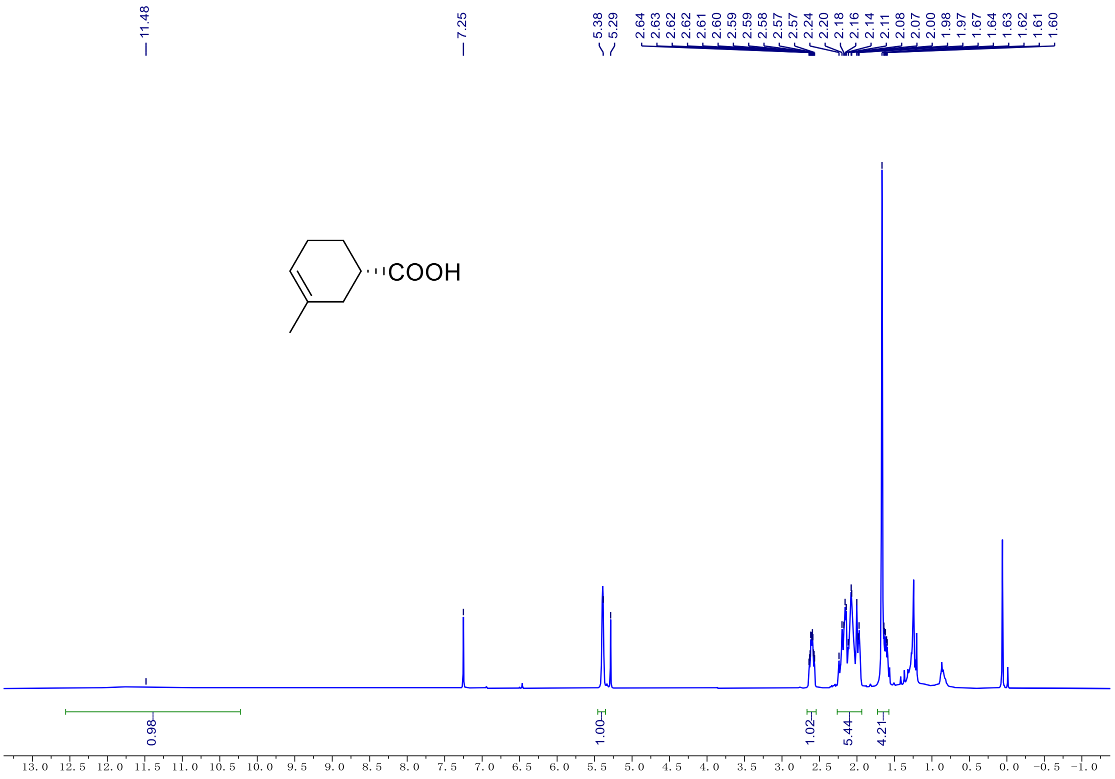
COOH
11.48
7.25
5.38
5.29
0.98
1.00
1.02
5.44
4.21

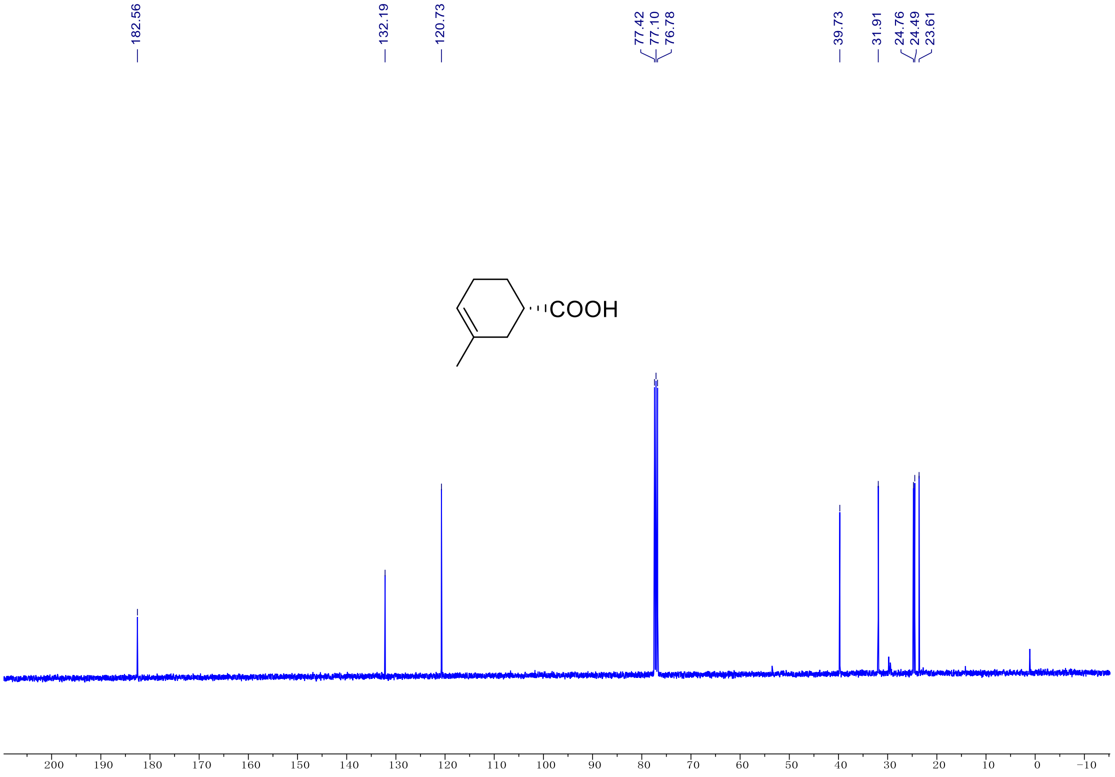
COOH
182.56
132.19
120.73
77.42
77.10
76.78
39.73
31.91
24.76
24.49
23.61

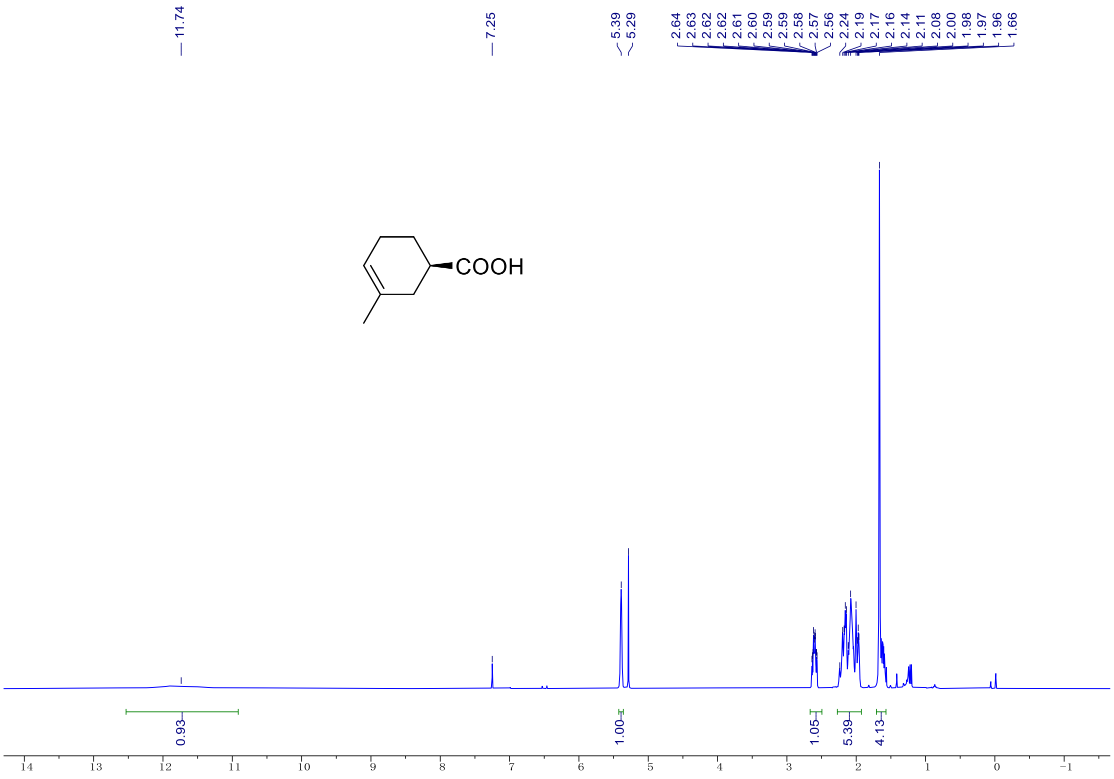
11.74
7.25
5.39
5.29
2.64 2.63 2.62 2.62 2.61 2.60 2.59 2.59 2.58 2.57 2.56 2.24 2.19 2.17 2.16 2.14 2.11 2.08 2.00 1.98 1.97 1.96 1.66
COOH
0.93
1.00
1.05
5.39
4.13
14 13 12 11 10 9 8 7 6 5 4 3 2 1 0 −1

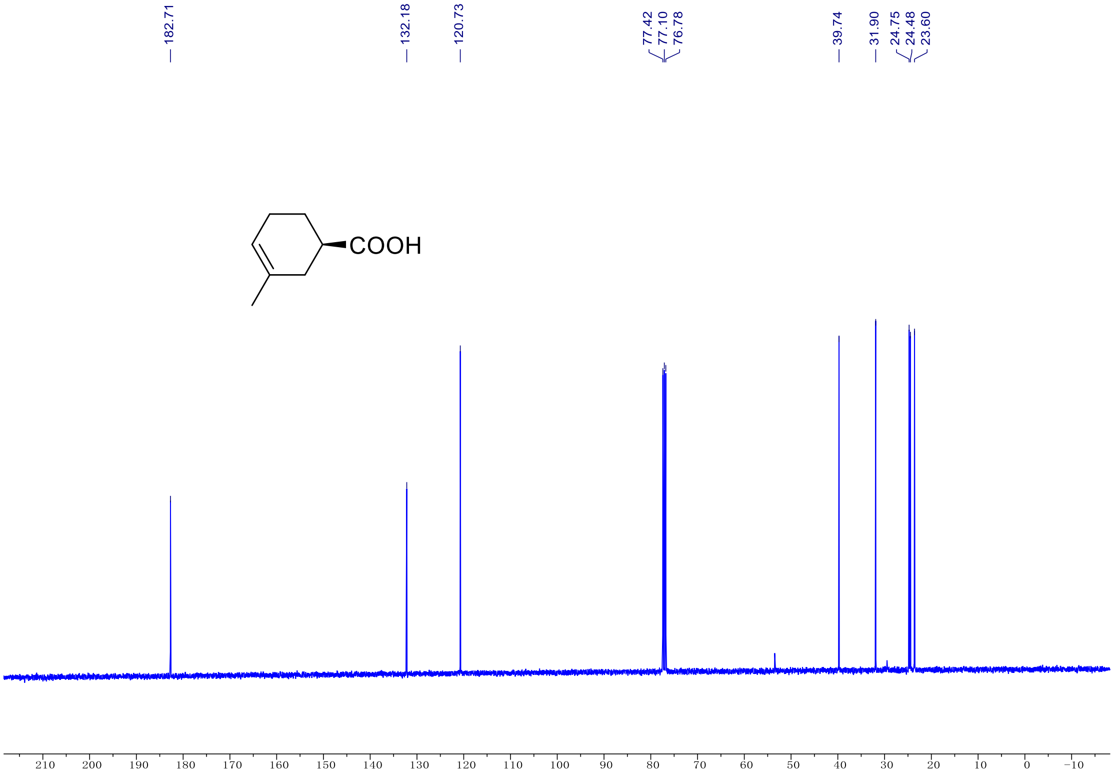
182.71
132.18
120.73
77.42 77.10 76.78
39.74
31.90
24.75 24.48 23.60
COOH
210 200 190 180 170 160 150 140 130 120 110 100 90 80 70 60 50 40 30 20 10 0 −10

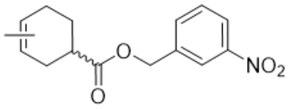

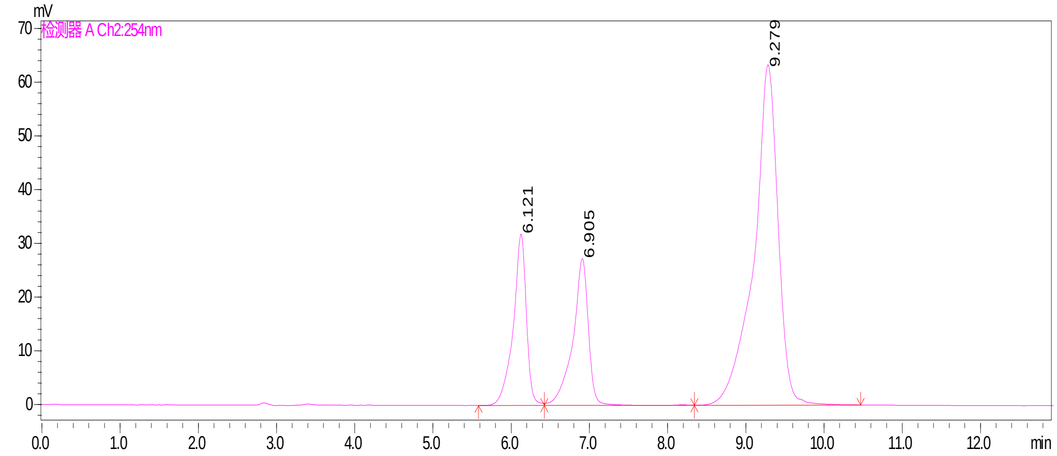

Peak#	Time	Area	Area%	height	Height%
1	6.121	372654	17.6397	31891	26.0291
2	6.905	375893	17.7930	27318	22.2969
3	9.279	1364038	64.5642	63310	51.6740

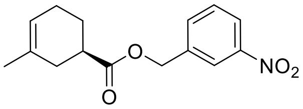

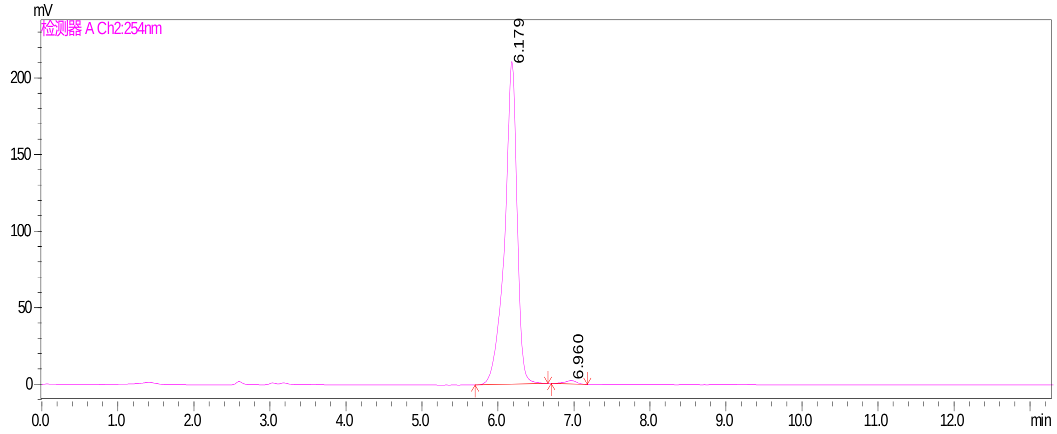

Peak#	Time	Area	Area%	height	Height%
1	6.179	2395514	99.0512	210684	98.9931
2	6.960	22947	0.9488	2143	1.0069

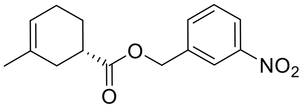

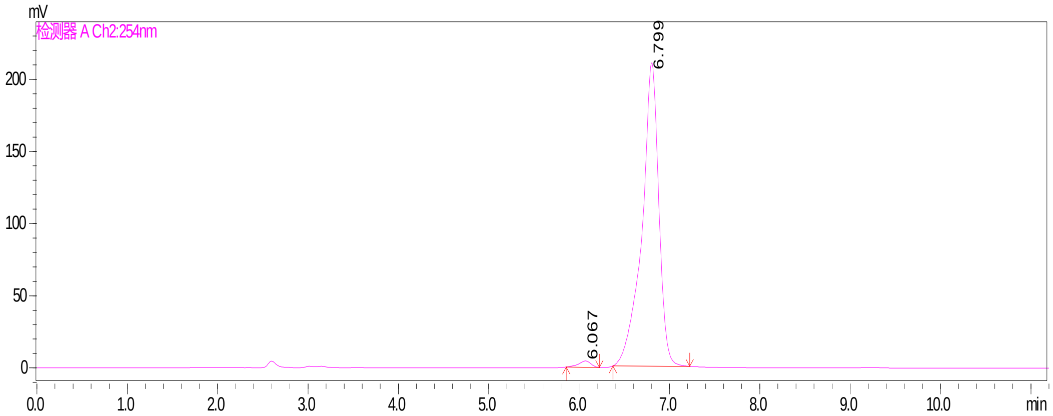

Peak#	Time	Area	Area%	height	Height%
1	6.067	41425	1.4963	4364	2.0344
2	6.799	2727028	98.5037	210125	97.9656

24. Atomic coordinates

No field:

Carbonic cation:

C	-0.02921	-0.01220	-0.01792
C	-0.06906	-0.04142	1.46277
C	0.55360	-0.20574	3.75708
C	-0.77768	-0.15730	4.15074
C	-1.79322	-0.04859	3.19832
C	-1.42850	0.04294	1.85613
C	0.92643	-0.16177	2.39863
H	1.32677	-0.29944	4.51129
H	-1.03125	-0.21961	5.20150
H	-2.83183	-0.05639	3.50559
H	1.96918	-0.23350	2.11274
C	-2.24803	0.10442	0.66557
C	-1.36698	0.06422	-0.48116
C	-1.64779	0.19719	-1.83991
C	-0.57870	0.18278	-2.73829
C	0.72674	0.07354	-2.27627
C	1.02053	-0.01166	-0.90058
H	-2.66070	0.32875	-2.20088
H	-0.76883	0.27331	-3.80036
H	1.54371	0.07377	-2.98900
H	2.04805	-0.06244	-0.56037
C	-3.67843	0.19045	0.62691
C	-4.40463	-0.53757	-0.33893
C	-4.36792	1.00097	1.55329
C	-5.78614	-0.46771	-0.35860
H	-3.88354	-1.20362	-1.01596
C	-5.74674	1.09476	1.49332
H	-3.81217	1.60059	2.26401
C	-6.45577	0.35475	0.54714
H	-6.34519	-1.05356	-1.07792
H	-6.27301	1.74456	2.18182
H	-7.53741	0.41903	0.51587

TS-1:

C	-0.04844	-0.11701	0.06835
C	-0.04435	-0.00417	1.54308
C	0.67263	0.21515	3.81028
C	-0.62058	-0.02881	4.26365
C	-1.65756	-0.26433	3.35781
C	-1.35594	-0.23377	2.00430
C	0.97941	0.22622	2.44250
H	1.46334	0.39100	4.53042

H	-0.82351	-0.04481	5.32740
H	-2.66115	-0.48417	3.70455
H	1.99388	0.40571	2.10580
C	-2.22531	-0.52155	0.84429
C	-1.35604	-0.43628	-0.34848
C	-1.67129	-0.54512	-1.69505
C	-0.64505	-0.37630	-2.62672
C	0.65009	-0.08187	-2.21163
C	0.96227	0.06263	-0.85444
H	-2.68126	-0.74643	-2.02893
H	-0.86356	-0.46431	-3.68395
H	1.42929	0.05375	-2.95282
H	1.96784	0.32010	-0.54237
C	-3.65856	-0.22246	0.81799
C	-4.52959	-0.89075	-0.05792
C	-4.14762	0.81344	1.62497
C	-5.86472	-0.52973	-0.11288
H	-4.16605	-1.71755	-0.65521
C	-5.48515	1.18449	1.54354
H	-3.48037	1.35588	2.28305
C	-6.34366	0.51243	0.68257
H	-6.53798	-1.06070	-0.77526
H	-5.85280	1.99758	2.15785
H	-7.38853	0.79587	0.62913
C	-1.71821	-3.39753	1.37765
C	-2.18065	-4.79098	1.31462
H	-3.16906	-4.92182	0.89216
C	-1.43325	-5.80339	1.74465
H	-0.44533	-5.64710	2.16379
H	-1.79986	-6.82170	1.68433
O	-2.44762	-2.49058	0.96237
O	-0.52261	-3.21528	1.88702
H	-0.28209	-2.27514	1.94042

IS:

C	0.06503	0.16967	-0.08600
C	0.00565	0.04239	1.38343
C	0.64816	-0.10590	3.67903
C	-0.68387	-0.29402	4.04889
C	-1.69467	-0.30629	3.08672
C	-1.32877	-0.13771	1.76362
C	1.00935	0.06515	2.34308
H	1.41555	-0.09170	4.44448
H	-0.93651	-0.42418	5.09452
H	-2.73437	-0.43664	3.36790

H	2.04650	0.21164	2.06360
C	-2.22970	-0.07765	0.54482
C	-1.23574	0.06472	-0.60096
C	-1.49139	0.13586	-1.96035
C	-0.40599	0.29938	-2.82121
C	0.89064	0.40722	-2.31717
C	1.14119	0.34674	-0.94708
H	-2.50393	0.06862	-2.34466
H	-0.57280	0.35193	-3.89037
H	1.71783	0.54294	-3.00425
H	2.15128	0.43780	-0.56426
C	-3.25792	1.03831	0.54925
C	-4.46189	0.90014	-0.14238
C	-2.95357	2.24552	1.17749
C	-5.35879	1.96251	-0.19244
H	-4.70854	-0.03296	-0.63472
C	-3.85159	3.30565	1.11787
H	-2.01756	2.36273	1.71235
C	-5.05656	3.16625	0.43615
H	-6.29620	1.84576	-0.72388
H	-3.60905	4.23957	1.61141
H	-5.75789	3.99169	0.39665
C	-2.61657	-2.49668	0.19973
C	-3.58528	-3.56778	0.33275
H	-4.55585	-3.27362	0.71032
C	-3.26814	-4.81754	-0.00584
H	-2.28629	-5.07566	-0.38757
H	-3.99758	-5.61282	0.09358
O	-3.04086	-1.32113	0.46460
O	-1.42177	-2.78431	-0.16422
H	-0.84775	-2.01735	-0.36352

m-CT:

C	-0.09467	-0.16901	-0.00772
C	-0.05860	-0.08840	1.46602
C	0.73150	0.02997	3.71712
C	-0.58058	-0.01431	4.18938
C	-1.65421	-0.08804	3.30077
C	-1.37162	-0.12194	1.94716
C	1.00863	-0.00688	2.35103
H	1.54969	0.09325	4.42530
H	-0.76787	0.01488	5.25623
H	-2.67780	-0.11114	3.65966
H	2.03089	0.02637	1.99183
C	-2.35123	-0.19040	0.79089
C	-1.43291	-0.25326	-0.42314

C	-1.77586	-0.31910	-1.76321
C	-0.74508	-0.33534	-2.70328
C	0.58783	-0.26459	-2.29741
C	0.92836	-0.17428	-0.94823
H	-2.81412	-0.34319	-2.07472
H	-0.98296	-0.39198	-3.75883
H	1.37241	-0.27050	-3.04527
H	1.96580	-0.10300	-0.64201
C	-3.34528	0.95039	0.70263
C	-4.58773	0.76443	0.09527
C	-2.97601	2.21740	1.15296
C	-5.45894	1.83967	-0.04563
H	-4.88421	-0.21637	-0.25919
C	-3.84890	3.28968	1.00296
H	-2.00889	2.37119	1.61902
C	-5.09213	3.10332	0.40685
H	-6.42627	1.68717	-0.51028
H	-3.55599	4.27076	1.35837
H	-5.77372	3.93885	0.29716
O	-3.19743	-1.40377	0.95027
C	-2.77961	-2.61484	0.95514
C	-3.70168	-3.60863	1.45932
H	-4.68810	-3.24202	1.71287
C	-3.31974	-4.87925	1.60328
H	-2.31489	-5.20615	1.36129
H	-4.01142	-5.61917	1.98984
C	-2.65573	-5.66474	-1.50356
H	-2.24722	-6.66431	-1.60560
H	-1.96216	-4.86699	-1.25252
C	-3.95673	-5.43112	-1.67906
H	-4.62396	-6.26565	-1.88764
C	-4.60013	-4.10803	-1.55395
C	-3.97577	-2.97692	-1.89895
H	-4.46892	-2.01541	-1.79103
H	-2.98472	-2.98749	-2.34256
C	-6.00406	-4.12449	-1.01656
H	-6.66441	-4.65583	-1.70957
H	-6.04650	-4.66211	-0.06381
H	-6.39248	-3.11482	-0.87350
O	-1.61210	-2.98854	0.57520
H	-1.07008	-2.29684	0.14548

p-CT:

C	-0.17764	-0.19304	-0.19473
C	-0.12358	-0.29467	1.27733
C	0.69063	-0.51843	3.51421

C	-0.61363	-0.46160	4.00751
C	-1.69537	-0.31537	3.13908
C	-1.43107	-0.24105	1.78152
C	0.95182	-0.43348	2.14736
H	1.51619	-0.62754	4.20801
H	-0.78707	-0.52587	5.07502
H	-2.71213	-0.26082	3.51389
H	1.96896	-0.47061	1.77445
C	-2.41778	-0.08537	0.63900
C	-1.51755	-0.08054	-0.58648
C	-1.87915	0.06171	-1.91296
C	-0.86289	0.06256	-2.86961
C	0.47249	-0.06687	-2.48971
C	0.83222	-0.19112	-1.14720
H	-2.91943	0.16833	-2.20276
H	-1.11365	0.16629	-3.91884
H	1.24516	-0.06184	-3.24991
H	1.87355	-0.27677	-0.85795
C	-3.30936	1.13920	0.72191
C	-4.57694	1.14587	0.13921
C	-2.81013	2.30368	1.30667
C	-5.34261	2.30777	0.15751
H	-4.97329	0.25022	-0.32303
C	-3.57739	3.46302	1.31467
H	-1.82158	2.31192	1.75258
C	-4.84634	3.46743	0.74354
H	-6.32991	2.30339	-0.28976
H	-3.18194	4.36217	1.77258
H	-5.44543	4.37059	0.75562
O	-3.36268	-1.23280	0.56435
C	-3.09033	-2.47268	0.72153
C	-4.20411	-3.38911	0.57632
H	-5.11666	-2.95990	0.18375
C	-4.08821	-4.65983	0.96599
H	-3.16575	-5.04749	1.38360
H	-4.93391	-5.33329	0.89222
C	-2.03005	-3.21731	-2.47263
H	-2.27314	-2.49569	-3.24391
H	-1.38863	-2.87835	-1.66254
C	-2.48443	-4.46940	-2.54470
H	-3.13918	-4.73935	-3.37176
C	-2.21568	-5.54607	-1.56848
C	-1.12217	-5.56026	-0.80101
H	-0.95699	-6.35122	-0.07619
H	-0.35459	-4.79781	-0.88909
C	-3.25220	-6.63744	-1.52081

H	-3.24255	-7.20155	-2.45944
H	-3.06769	-7.33270	-0.70040
H	-4.25784	-6.21960	-1.41546
O	-1.93631	-2.95215	1.00950
H	-1.24969	-2.29501	1.24162

m-TS2:

C	-0.02757	0.00019	0.01044
C	-0.02483	0.00054	1.48739
C	0.71726	0.01019	3.75877
C	-0.59871	-0.13796	4.19761
C	-1.65039	-0.20703	3.28217
C	-1.34331	-0.13624	1.93561
C	1.02002	0.08091	2.39936
H	1.51784	0.06964	4.48719
H	-0.80661	-0.19290	5.25976
H	-2.67779	-0.30803	3.61635
H	2.04561	0.19243	2.06572
C	-2.30086	-0.15481	0.75717
C	-1.35022	-0.13429	-0.43605
C	-1.66207	-0.20857	-1.78357
C	-0.61362	-0.15140	-2.70237
C	0.70470	-0.00904	-2.26688
C	1.01282	0.06858	-0.90942
H	-2.69157	-0.30172	-2.11485
H	-0.82481	-0.21033	-3.76353
H	1.50358	0.04093	-2.99780
H	2.04034	0.17705	-0.58087
C	-3.25535	1.03002	0.71237
C	-4.54079	0.89487	0.18978
C	-2.80712	2.28507	1.12666
C	-5.37369	2.00693	0.09462
H	-4.89998	-0.07393	-0.13519
C	-3.64083	3.39233	1.02467
H	-1.80622	2.40115	1.52900
C	-4.92760	3.25631	0.51066
H	-6.37460	1.89179	-0.30549
H	-3.28442	4.36196	1.35265
H	-5.57876	4.11972	0.43766
O	-3.16285	-1.33026	0.78876
C	-2.72242	-2.56609	0.83287
C	-3.59930	-3.56388	1.24208
H	-4.60472	-3.25404	1.48975
C	-3.19323	-4.89798	1.28832
H	-2.13369	-5.11594	1.37352
H	-3.84935	-5.60960	1.77343

C	-3.08964	-5.73090	-0.57839
H	-2.68527	-6.69954	-0.30712
H	-2.36741	-4.99909	-0.92675
C	-4.41224	-5.64755	-0.93229
H	-5.04484	-6.51206	-0.74068
C	-5.07597	-4.44563	-1.36480
C	-4.37617	-3.30976	-1.57696
H	-4.88713	-2.38207	-1.81481
H	-3.29276	-3.29539	-1.61337
C	-6.57925	-4.48122	-1.43866
H	-6.90839	-5.26042	-2.13125
H	-7.00704	-4.71419	-0.45918
H	-6.98154	-3.52602	-1.77601
O	-1.50003	-2.88553	0.48694
H	-1.00875	-2.16876	0.04725

p-TS2:

C	-0.21017	-0.12409	-0.12624
C	-0.07671	-0.10028	1.34382
C	0.86117	-0.12554	3.54324
C	-0.41843	-0.11822	4.10049
C	-1.54886	-0.09401	3.28322
C	-1.35907	-0.08716	1.91148
C	1.04758	-0.11756	2.16174
H	1.72565	-0.13864	4.19712
H	-0.53453	-0.12617	5.17768
H	-2.54734	-0.07743	3.70751
H	2.04547	-0.12262	1.73802
C	-2.41429	-0.04561	0.81571
C	-1.57174	-0.12467	-0.45048
C	-2.00671	-0.14751	-1.76217
C	-1.04347	-0.20139	-2.77131
C	0.31531	-0.20680	-2.45595
C	0.74818	-0.16239	-1.13040
H	-3.06544	-0.12048	-1.99868
H	-1.35322	-0.22876	-3.80958
H	1.04707	-0.24023	-3.25496
H	1.80632	-0.15912	-0.89405
C	-3.27305	1.21006	0.84021
C	-4.60870	1.18021	0.44187
C	-2.68322	2.42675	1.18737
C	-5.34908	2.35954	0.40426
H	-5.07801	0.24379	0.16786
C	-3.42516	3.60063	1.14192
H	-1.64243	2.46158	1.49190
C	-4.76169	3.57033	0.75229

H	-6.38902	2.32652	0.10016
H	-2.95773	4.53967	1.41487
H	-5.34095	4.48595	0.72214
O	-3.36052	-1.15643	0.90081
C	-3.02841	-2.42180	0.89288
C	-4.03675	-3.36917	0.71461
H	-5.00435	-3.00416	0.40077
C	-3.78097	-4.70981	0.95215
H	-2.91210	-4.98301	1.54065
H	-4.61379	-5.40199	0.98449
C	-3.21154	-3.25328	-2.27479
H	-3.47818	-2.38056	-2.86023
H	-2.29878	-3.18955	-1.69026
C	-3.95310	-4.36890	-2.35052
H	-4.82872	-4.37528	-2.99474
C	-3.72563	-5.57345	-1.57252
C	-2.71842	-5.66593	-0.65417
H	-2.54901	-6.60848	-0.14539
H	-1.90262	-4.95208	-0.63112
C	-4.73779	-6.66573	-1.71735
H	-4.79717	-6.98295	-2.76300
H	-4.49595	-7.52949	-1.09855
H	-5.73201	-6.29462	-1.44469
O	-1.79486	-2.82531	1.05422
H	-1.19626	-2.15105	1.42481

m-PS:

C	-0.11562	0.21936	-0.30366
C	-0.01709	0.04543	1.15943
C	0.86942	-0.23873	3.36134
C	-0.42075	-0.37055	3.87676
C	-1.53241	-0.28721	3.03833
C	-1.31046	-0.08262	1.68686
C	1.08723	-0.02991	2.00023
H	1.71815	-0.30004	4.03271
H	-0.56015	-0.53362	4.93876
H	-2.53960	-0.37587	3.43183
H	2.09317	0.07377	1.60981
C	-2.33460	0.06054	0.57629
C	-1.46686	0.18835	-0.66698
C	-1.87068	0.29270	-1.98560
C	-0.88182	0.42562	-2.96176
C	0.46668	0.46668	-2.60712
C	0.86607	0.36585	-1.27466
H	-2.92231	0.28184	-2.25301
H	-1.16484	0.50736	-4.00450

H	1.21699	0.57978	-3.38112
H	1.91618	0.39719	-1.00729
C	-3.29720	1.22215	0.74219
C	-4.59428	1.16431	0.23253
C	-2.83788	2.39804	1.33673
C	-5.42781	2.27474	0.33136
H	-4.96284	0.25917	-0.23406
C	-3.67297	3.50568	1.42452
H	-1.82813	2.45367	1.72919
C	-4.97070	3.44618	0.92491
H	-6.43750	2.22006	-0.05900
H	-3.30838	4.41445	1.88896
H	-5.62258	4.30890	0.99892
C	-2.81452	-2.34672	0.36392
C	-3.82664	-3.34351	-0.06023
C	-3.99602	-3.17361	-1.58922
C	-3.44610	-4.78100	0.28396
H	-4.76646	-3.06062	0.42437
H	-3.01265	-3.16319	-2.07806
H	-4.46679	-2.20850	-1.80075
H	-2.54432	-5.05210	-0.27299
H	-3.22314	-4.87443	1.34889
C	-4.59586	-5.70373	-0.11160
H	-4.25961	-6.74310	-0.06868
H	-5.41360	-5.61924	0.61440
C	-4.83713	-4.28498	-2.17164
C	-5.11093	-5.40045	-1.49293
H	-5.74308	-6.15231	-1.95915
C	-5.33390	-4.03002	-3.56575
H	-4.49668	-3.82052	-4.23995
H	-5.88468	-4.88793	-3.95437
H	-5.99007	-3.15392	-3.58956
O	-3.20693	-1.13727	0.45431
O	-1.61805	-2.74745	0.55791
H	-0.98826	-2.07385	0.88898

p-PS:

C	0.03158	0.32876	-0.16306
C	-0.02447	0.07880	1.29210
C	0.62626	-0.28563	3.56171
C	-0.70324	-0.51764	3.91562
C	-1.71719	-0.44256	2.95979
C	-1.35689	-0.14623	1.65739
C	0.98194	0.01483	2.24747
H	1.39645	-0.34115	4.32242
H	-0.95115	-0.75018	4.94444

H	-2.75531	-0.60461	3.23035
H	2.01757	0.19238	1.98081
C	-2.25994	0.03411	0.45325
C	-1.26947	0.25757	-0.68173
C	-1.52794	0.42223	-2.03127
C	-0.44479	0.65346	-2.87970
C	0.85238	0.73174	-2.37171
C	1.10612	0.57188	-1.01019
H	-2.54127	0.37881	-2.41725
H	-0.61381	0.78240	-3.94212
H	1.67737	0.92163	-3.04847
H	2.11697	0.63676	-0.62403
C	-3.26455	1.16626	0.56622
C	-4.47572	1.11742	-0.12422
C	-2.92906	2.30507	1.29824
C	-5.34956	2.19878	-0.06894
H	-4.74766	0.23885	-0.69651
C	-3.80360	3.38519	1.34310
H	-1.98650	2.35321	1.83236
C	-5.01638	3.33384	0.66292
H	-6.29301	2.15074	-0.60031
H	-3.53644	4.26542	1.91617
H	-5.69961	4.17423	0.70440
C	-2.66798	-2.34676	-0.03372
C	-3.68988	-3.40405	-0.21870
C	-4.24652	-3.24299	-1.65366
C	-3.14037	-4.81591	-0.01946
H	-4.49483	-3.19353	0.49121
H	-3.41435	-3.16831	-2.36522
H	-4.81247	-2.31020	-1.72322
H	-2.39733	-5.02016	-0.79585
H	-2.64419	-4.90254	0.94946
C	-5.17734	-5.55700	-1.32241
C	-6.11302	-6.67219	-1.68861
H	-5.55093	-7.57065	-1.96280
H	-6.74073	-6.93986	-0.83212
H	-6.76197	-6.39937	-2.52241
C	-4.29030	-5.81279	-0.12770
H	-3.88843	-6.82852	-0.19487
H	-4.90289	-5.78934	0.78219
C	-5.13100	-4.40995	-2.00276
H	-5.77344	-4.27431	-2.86910
O	-3.09327	-1.17881	0.24281
O	-1.44248	-2.66287	-0.20799
H	-0.79367	-1.93327	-0.18063

m-TS3:

C	0.00825	0.01395	0.00879
C	0.00213	0.00998	1.48811
C	0.70677	0.00232	3.76903
C	-0.60387	-0.19909	4.19379
C	-1.64288	-0.29730	3.26575
C	-1.32583	-0.17571	1.92057
C	1.02806	0.10330	2.40880
H	1.49876	0.07298	4.50580
H	-0.81867	-0.28708	5.25172
H	-2.66187	-0.48233	3.58707
H	2.05500	0.24670	2.09327
C	-2.20059	-0.30523	0.73709
C	-1.31452	-0.16883	-0.44063
C	-1.62088	-0.16364	-1.79359
C	-0.57241	-0.01626	-2.70403
C	0.73647	0.14627	-2.25914
C	1.04166	0.17535	-0.89280
H	-2.63960	-0.26615	-2.14674
H	-0.78279	-0.01807	-3.76672
H	1.53375	0.26665	-2.98361
H	2.06099	0.32684	-0.55686
C	-3.61131	0.09356	0.73539
C	-4.52390	-0.43044	-0.19432
C	-4.02921	1.08868	1.62888
C	-5.83011	0.02826	-0.21462
H	-4.21805	-1.22221	-0.86630
C	-5.33648	1.55999	1.58325
H	-3.32834	1.52438	2.32967
C	-6.23767	1.02771	0.66989
H	-6.53617	-0.39346	-0.92002
H	-5.64717	2.34102	2.26668
H	-7.25955	1.38813	0.64404
C	-1.67798	-3.15316	0.56622
C	-1.88324	-4.33360	-0.32654
C	-1.46589	-3.87278	-1.73896
C	-1.10279	-5.58034	0.08057
H	-2.95779	-4.53912	-0.33349
H	-0.47832	-3.39120	-1.70276
H	-2.16841	-3.11564	-2.10316
H	-0.03163	-5.36956	0.00679
H	-1.31852	-5.84802	1.11741
C	-1.46862	-6.72637	-0.86042
H	-0.77223	-7.55714	-0.71625
H	-2.46234	-7.11722	-0.60890
C	-1.42425	-5.02775	-2.71152

C	-1.44428	-6.29822	-2.30373
H	-1.43691	-7.08724	-3.05236
C	-1.36043	-4.63205	-4.15881
H	-0.50020	-3.97874	-4.34091
H	-1.27602	-5.50458	-4.80867
H	-2.25432	-4.06873	-4.44622
O	-2.51602	-2.24434	0.61208
O	-0.54037	-3.12981	1.21297
H	-0.45177	-2.34621	1.78316

p-TS3:

C	0.04445	0.02257	0.04269
C	-0.00828	-0.04149	1.51927
C	0.61994	-0.12255	3.81797
C	-0.71478	-0.23611	4.19802
C	-1.72730	-0.24952	3.23682
C	-1.35768	-0.13371	1.90529
C	0.99214	-0.02714	2.47042
H	1.38981	-0.11320	4.58116
H	-0.96902	-0.31943	5.24761
H	-2.76738	-0.36081	3.52211
H	2.03596	0.05347	2.19006
C	-2.20053	-0.16422	0.69976
C	-1.27625	-0.05323	-0.45163
C	-1.54245	0.02119	-1.81137
C	-0.45894	0.12562	-2.68727
C	0.84380	0.17636	-2.20137
C	1.11132	0.13648	-0.82635
H	-2.55499	0.00135	-2.19527
H	-0.63735	0.17459	-3.75440
H	1.66868	0.26210	-2.89932
H	2.12896	0.20247	-0.45924
C	-3.58649	0.30412	0.65912
C	-4.48864	-0.18045	-0.30084
C	-3.99107	1.30899	1.54668
C	-5.77413	0.32865	-0.36142
H	-4.18968	-0.98337	-0.96419
C	-5.27714	1.83163	1.46139
H	-3.29424	1.71140	2.27141
C	-6.16861	1.34002	0.51623
H	-6.47465	-0.06304	-1.08932
H	-5.57960	2.62052	2.13944
H	-7.17450	1.74002	0.46079
C	-2.12057	-3.12585	0.34017
C	-2.77079	-4.46011	0.54555
C	-3.92977	-4.56784	-0.46473

C	-1.82303	-5.64753	0.39271
H	-3.19458	-4.44198	1.55436
H	-3.56745	-4.31086	-1.46915
H	-4.70410	-3.83828	-0.21118
H	-1.47395	-5.69465	-0.64326
H	-0.94575	-5.52701	1.03209
C	-3.92643	-7.02051	0.08827
C	-4.57014	-8.37694	0.10407
H	-3.96096	-9.09903	-0.44922
H	-4.64910	-8.75022	1.13070
H	-5.56950	-8.35658	-0.33404
C	-2.56951	-6.93200	0.74496
H	-1.97288	-7.80019	0.44682
H	-2.69630	-7.01261	1.83216
C	-4.51194	-5.95612	-0.46502
H	-5.48090	-6.07048	-0.94519
O	-2.66438	-2.10033	0.75681
O	-0.99682	-3.14739	-0.33519
H	-0.63405	-2.25835	-0.49140

Diene (isoprene):

C	-0.11361	0.23361	0.00382
H	-0.16971	0.12590	1.08157
H	0.76558	0.72457	-0.40296
C	-1.09277	-0.20661	-0.78492
H	-1.98153	-0.64570	-0.33381
C	-1.09919	-0.11112	-2.26135
C	0.01045	-0.28462	-2.98401
H	-0.00477	-0.19521	-4.06551
H	0.95733	-0.53411	-2.51637
C	-2.43875	0.17009	-2.88656
H	-3.15505	-0.61251	-2.61701
H	-2.84586	1.11646	-2.51753
H	-2.36959	0.21835	-3.97405

Dienophile (acrylic acid):

C	-3.96817	-1.36638	1.71549
C	-4.72224	-2.62256	1.52491
H	-4.41711	-3.19842	0.65901
C	-5.69343	-3.01978	2.34052
H	-5.98851	-2.43331	3.20370
H	-6.22046	-3.95028	2.16137
O	-3.08109	-1.00396	0.97483
O	-4.34724	-0.65374	2.78242
H	-3.80708	0.15032	2.83017

+2.57 V/nm:
Carbonic cation:

C	0.00025	0.00456	0.00350
C	0.00120	0.00383	1.48468
C	0.70424	0.00365	3.76427
C	-0.60644	-0.17054	4.18891
C	-1.64850	-0.26308	3.25842
C	-1.33778	-0.14295	1.91187
C	1.02719	0.07980	2.39851
H	1.50056	0.06420	4.49803
H	-0.82563	-0.25001	5.24724
H	-2.66382	-0.44146	3.59056
H	2.05742	0.18342	2.07834
C	-2.18823	-0.26211	0.71889
C	-1.34813	-0.16506	-0.42557
C	-1.67790	-0.11856	-1.78997
C	-0.64580	0.02398	-2.71318
C	0.66923	0.15235	-2.27976
C	1.00902	0.15956	-0.90552
H	-2.70605	-0.16578	-2.12811
H	-0.86814	0.05275	-3.77230
H	1.45718	0.27155	-3.01506
H	2.04040	0.29422	-0.60178
C	-3.60955	-0.46367	0.72801
C	-4.20879	-1.32274	-0.21524
C	-4.41780	0.20908	1.66883

C	-5.58102	-1.50903	-0.20824
H	-3.58806	-1.87645	-0.90908
C	-5.79033	0.04409	1.63939
H	-3.96905	0.91302	2.35894
C	-6.37134	-0.81937	0.70832
H	-6.03611	-2.18241	-0.92409
H	-6.41615	0.59825	2.32878
H	-7.44745	-0.94865	0.69085

TS-1:

C	-0.00145	-0.01942	-0.00278
C	-0.00208	-0.00515	1.47627
C	0.70577	0.00955	3.75666
C	-0.61860	-0.02506	4.18588
C	-1.66353	-0.04530	3.25791
C	-1.33752	-0.01443	1.91317
C	1.03151	0.01379	2.39548
H	1.50435	0.02802	4.49035
H	-0.84151	-0.03953	5.24659
H	-2.69786	-0.09409	3.57951
H	2.06596	0.03157	2.07266
C	-2.22782	-0.06292	0.72740
C	-1.34532	-0.02186	-0.44513
C	-1.66024	0.03633	-1.79819
C	-0.60792	0.04008	-2.71680
C	0.71268	0.00505	-2.28148
C	1.03323	-0.01235	-0.91408
H	-2.68482	0.07864	-2.14678
H	-0.82307	0.07459	-3.77766
H	1.51199	0.00889	-3.01359
H	2.06865	-0.00483	-0.59407
C	-3.60347	0.43969	0.72179
C	-4.55115	-0.05467	-0.18698
C	-3.94551	1.49685	1.57498
C	-5.81971	0.49854	-0.23506
H	-4.29923	-0.88954	-0.83093
C	-5.21419	2.06123	1.50258
H	-3.20876	1.91245	2.25141
C	-6.15111	1.56167	0.60515
H	-6.54850	0.10965	-0.93604
H	-5.46452	2.90088	2.14041
H	-7.13901	2.00393	0.54738
C	-2.38469	-2.98474	0.29121
C	-3.18823	-4.21002	0.39118
H	-4.05685	-4.13300	1.03311
C	-2.88214	-5.31027	-0.29216

H	-2.01706	-5.35607	-0.94471
H	-3.50647	-6.19385	-0.22462
O	-2.72519	-1.97399	0.92339
O	-1.32683	-3.06278	-0.47382
H	-0.83185	-2.22781	-0.52862

IS:

C	-0.00544	0.00279	-0.01166
C	0.00026	0.00729	1.46437
C	0.74268	-0.00668	3.73891
C	-0.57993	0.01592	4.18337
C	-1.63343	0.04349	3.26742
C	-1.32292	0.03874	1.91983
C	1.04814	-0.01082	2.37814
H	1.54982	-0.01814	4.46338
H	-0.79085	0.02184	5.24703
H	-2.66621	0.07965	3.59854
H	2.07812	-0.02503	2.04039
C	-2.27574	0.10649	0.73986
C	-1.33708	0.04185	-0.45572
C	-1.65865	0.04513	-1.80403
C	-0.60973	-0.01280	-2.72290
C	0.71568	-0.05588	-2.28933
C	1.03408	-0.04423	-0.93126
H	-2.69018	0.09137	-2.13755
H	-0.82644	-0.01869	-3.78432
H	1.51116	-0.09511	-3.02422
H	2.06832	-0.06932	-0.60749
C	-3.17976	1.32394	0.71035
C	-4.42538	1.26701	0.08525
C	-2.71282	2.53762	1.21644
C	-5.20287	2.41618	-0.02013
H	-4.78984	0.33462	-0.32908
C	-3.49204	3.68372	1.10217
H	-1.73340	2.59649	1.67877
C	-4.73891	3.62564	0.48660
H	-6.16387	2.36653	-0.51854
H	-3.11528	4.62719	1.48024
H	-5.33849	4.52264	0.38376
C	-2.94227	-2.27486	0.56842
C	-4.01262	-3.21904	0.82815
H	-4.89613	-2.80645	1.29722
C	-3.90404	-4.48971	0.43837
H	-3.01689	-4.86081	-0.06361
H	-4.71979	-5.18437	0.60062
O	-3.21314	-1.04769	0.81033

O	-1.82446	-2.71775	0.13081
H	-1.20613	-2.04062	-0.21868

m-complex:

C	-0.00964	-0.03225	0.01488
C	-0.02529	-0.03006	1.49049
C	0.68240	-0.02560	3.77607
C	-0.64692	-0.04905	4.20009
C	-1.68633	-0.05585	3.26790
C	-1.35476	-0.04512	1.92548
C	1.00861	-0.01571	2.42014
H	1.47819	-0.01280	4.51295
H	-0.87442	-0.05403	5.26032
H	-2.72504	-0.05964	3.58207
H	2.04353	0.00446	2.09813
C	-2.29535	-0.02704	0.73287
C	-1.33567	-0.04793	-0.44860
C	-1.63434	-0.03222	-1.80138
C	-0.57054	-0.03654	-2.70540
C	0.74865	-0.03635	-2.25192
C	1.04481	-0.02683	-0.88865
H	-2.66071	-0.00392	-2.15125
H	-0.77146	-0.03044	-3.77001
H	1.55642	-0.03371	-2.97440
H	2.07402	-0.00943	-0.54865
C	-3.25350	1.14696	0.68400
C	-4.51075	1.01611	0.09591
C	-2.82553	2.39585	1.13612
C	-5.33777	2.12849	-0.03092
H	-4.84438	0.05360	-0.27367
C	-3.65423	3.50403	1.00238
H	-1.83774	2.50977	1.57027
C	-4.91207	3.37327	0.41998
H	-6.30754	2.02158	-0.50290
H	-3.30825	4.47507	1.33774
H	-5.55015	4.24107	0.29986
O	-3.17335	-1.22061	0.82082
C	-2.81023	-2.44315	0.66941
C	-3.74678	-3.44493	1.12539
H	-4.70735	-3.06875	1.45230
C	-3.42384	-4.74099	1.10025
H	-2.44909	-5.08175	0.77136
H	-4.13522	-5.49087	1.42655
C	-3.20124	-5.35101	-1.90664
H	-2.93609	-6.38836	-2.07934
H	-2.38833	-4.65378	-1.72155

C	-4.47139	-4.94524	-1.95830
H	-5.25737	-5.67767	-2.13246
C	-4.92076	-3.55467	-1.75997
C	-4.17225	-2.50386	-2.11532
H	-4.54052	-1.49101	-1.98327
H	-3.22337	-2.62884	-2.62816
C	-6.28988	-3.40080	-1.15707
H	-7.04420	-3.80290	-1.84069
H	-6.36966	-3.97052	-0.22446
H	-6.52730	-2.35367	-0.96036
O	-1.68089	-2.82188	0.19503
H	-1.14352	-2.11458	-0.22165

p-complex:

C	0.07467	0.05814	0.05338
C	0.02328	0.03932	1.52769
C	0.67677	0.06717	3.82896
C	-0.65830	-0.03965	4.22105
C	-1.67353	-0.10307	3.26451
C	-1.31293	-0.06147	1.93016
C	1.03283	0.10830	2.48114
H	1.45291	0.12190	4.58467
H	-0.90969	-0.06657	5.27553
H	-2.71697	-0.17347	3.55433
H	2.07176	0.19371	2.18329
C	-2.22376	-0.09967	0.71504
C	-1.23549	-0.03856	-0.44300
C	-1.50044	-0.02156	-1.80270
C	-0.41683	0.06106	-2.67909
C	0.88753	0.14634	-2.19226
C	1.14829	0.15308	-0.82176
H	-2.51600	-0.06555	-2.18083
H	-0.59190	0.06864	-3.74818
H	1.71033	0.21644	-2.89404
H	2.16499	0.23553	-0.45450
C	-3.26542	0.99929	0.64027
C	-4.48398	0.77934	-0.00219
C	-2.95117	2.27171	1.11807
C	-5.38561	1.82856	-0.15519
H	-4.73142	-0.20155	-0.39253
C	-3.85460	3.31642	0.95747
H	-1.99316	2.45422	1.59374
C	-5.07369	3.09722	0.32220
H	-6.32491	1.65504	-0.66739
H	-3.59708	4.30740	1.31354
H	-5.77045	3.91544	0.18178

O	-3.01586	-1.35248	0.78095
C	-2.60380	-2.54757	0.55252
C	-3.50009	-3.60831	0.94097
H	-4.39950	-3.29265	1.45351
C	-3.24453	-4.87872	0.60875
H	-2.35148	-5.15998	0.06071
H	-3.92571	-5.66998	0.89775
C	-4.11185	-2.62628	-2.24946
H	-4.12836	-1.59069	-2.57090
H	-3.15085	-3.13640	-2.24720
C	-5.24578	-3.26083	-1.94299
H	-6.18096	-2.70309	-1.95605
C	-5.34962	-4.68101	-1.56062
C	-4.52214	-5.61199	-2.04875
H	-4.61527	-6.65294	-1.75490
H	-3.77227	-5.37228	-2.79506
C	-6.45054	-5.01004	-0.58994
H	-6.46903	-6.07475	-0.35125
H	-6.33089	-4.43631	0.33725
H	-7.42146	-4.73359	-1.01238
O	-1.46297	-2.84834	0.04905
H	-0.95194	-2.09132	-0.30700

m-TS2 (R-route):

C	0.00687	0.02126	-0.00509
C	0.00149	0.02590	1.47106
C	0.72862	0.02660	3.75084
C	-0.59644	-0.03193	4.18442
C	-1.64350	-0.05126	3.26022
C	-1.32549	-0.02213	1.91506
C	1.04265	0.05639	2.39216
H	1.53013	0.05037	4.48130
H	-0.81497	-0.05399	5.24646
H	-2.67918	-0.07965	3.58337
H	2.07421	0.10199	2.06153
C	-2.27739	0.01489	0.72803
C	-1.32069	-0.01680	-0.45811
C	-1.62928	-0.04937	-1.80859
C	-0.57254	-0.06068	-2.72019
C	0.75029	-0.02353	-2.27708
C	1.05548	0.02149	-0.91721
H	-2.65900	-0.05928	-2.15085
H	-0.78045	-0.09045	-3.78315
H	1.55249	-0.02677	-3.00573
H	2.08707	0.05511	-0.58546
C	-3.15333	1.26024	0.69974

C	-4.49218	1.20094	0.32135
C	-2.56709	2.49793	0.97577
C	-5.23976	2.37392	0.22413
H	-4.95531	0.25009	0.08867
C	-3.31541	3.66302	0.87401
H	-1.51703	2.55326	1.24617
C	-4.65616	3.60438	0.49797
H	-6.27601	2.32034	-0.08931
H	-2.84565	4.62057	1.06786
H	-5.23426	4.51575	0.39815
O	-3.20791	-1.10385	0.76824
C	-2.88591	-2.36734	0.62993
C	-3.87071	-3.32083	0.89520
H	-4.85567	-2.94166	1.12988
C	-3.60982	-4.67203	0.74461
H	-2.58502	-5.02525	0.74907
H	-4.35997	-5.38584	1.05963
C	-3.52603	-5.24624	-1.34925
H	-3.29346	-6.29748	-1.22129
H	-2.68143	-4.58532	-1.51506
C	-4.79950	-4.87942	-1.66874
H	-5.57213	-5.64551	-1.64406
C	-5.24456	-3.52455	-1.91224
C	-4.36739	-2.50302	-1.97687
H	-4.71709	-1.48481	-2.11682
H	-3.29397	-2.65898	-1.99779
C	-6.73036	-3.30737	-2.00474
H	-7.14904	-3.89857	-2.82333
H	-7.22480	-3.63187	-1.08333
H	-6.96856	-2.25880	-2.18310
O	-1.68911	-2.76005	0.28480
H	-1.13958	-2.06557	-0.12590

p-TS2 (R route):

C	-0.00883	-0.00950	-0.00876
C	0.01037	-0.00183	1.46690
C	0.77629	-0.00887	3.73368
C	-0.54134	-0.06456	4.18991
C	-1.60417	-0.07815	3.28382
C	-1.30888	-0.04541	1.93347
C	1.06735	0.02307	2.37004
H	1.59024	0.00938	4.45040
H	-0.74164	-0.08993	5.25543
H	-2.63416	-0.10523	3.62466
H	2.09329	0.06476	2.02199
C	-2.27818	-0.00780	0.76328

C	-1.34366	-0.05101	-0.43945
C	-1.67451	-0.09139	-1.78424
C	-0.63287	-0.10351	-2.71309
C	0.69688	-0.05927	-2.29238
C	1.02438	-0.00929	-0.93779
H	-2.70940	-0.10726	-2.11089
H	-0.85829	-0.13889	-3.77227
H	1.48685	-0.06171	-3.03428
H	2.06115	0.02850	-0.62315
C	-3.16518	1.22719	0.72716
C	-4.42531	1.17137	0.13225
C	-2.67313	2.44771	1.19159
C	-5.19054	2.32799	0.01293
H	-4.80882	0.23172	-0.24792
C	-3.44029	3.60067	1.06555
H	-1.68283	2.50604	1.63063
C	-4.70101	3.54392	0.47811
H	-6.16277	2.27858	-0.46351
H	-3.04364	4.54839	1.41152
H	-5.29077	4.44616	0.36445
O	-3.21170	-1.13262	0.83152
C	-2.89477	-2.39084	0.69320
C	-3.87845	-3.34158	1.01542
H	-4.84844	-2.95073	1.29038
C	-3.63034	-4.68278	0.87523
H	-2.61616	-5.04384	0.75404
H	-4.36917	-5.39960	1.21146
C	-4.37695	-2.59553	-1.97386
H	-4.67365	-1.58504	-2.23334
H	-3.31171	-2.79245	-1.90365
C	-5.29599	-3.56233	-1.84790
H	-6.34439	-3.30959	-1.98413
C	-5.01471	-4.94535	-1.48194
C	-3.75014	-5.41336	-1.31781
H	-3.58527	-6.45960	-1.08430
H	-2.88214	-4.84258	-1.62915
C	-6.20582	-5.80580	-1.19087
H	-5.91720	-6.81772	-0.90655
H	-6.80631	-5.35902	-0.39001
H	-6.84945	-5.85958	-2.07409
O	-1.72145	-2.79210	0.30093
H	-1.17294	-2.09883	-0.11542

m-TS2 (S route):

C	0.00519	-0.01079	0.03443
C	0.02502	0.02951	1.50914

C	0.79113	0.08837	3.77554
C	-0.52718	0.12026	4.23229
C	-1.58916	0.11237	3.32537
C	-1.29498	0.05601	1.97569
C	1.08242	0.04766	2.41191
H	1.60577	0.10383	4.49156
H	-0.72768	0.16236	5.29736
H	-2.61926	0.15900	3.66433
H	2.10900	0.03492	2.06361
C	-2.26601	0.05530	0.80717
C	-1.33128	-0.01632	-0.39493
C	-1.66352	-0.06417	-1.73875
C	-0.62288	-0.12118	-2.66764
C	0.70753	-0.11518	-2.24795
C	1.03747	-0.05724	-0.89376
H	-2.69898	-0.05828	-2.06307
H	-0.85023	-0.16399	-3.72610
H	1.49656	-0.15353	-2.98986
H	2.07501	-0.04744	-0.57970
C	-3.17731	1.26921	0.73769
C	-4.43488	1.17480	0.14149
C	-2.71041	2.51043	1.17233
C	-5.22165	2.31301	-0.00830
H	-4.79929	0.22018	-0.21837
C	-3.49909	3.64512	1.01570
H	-1.72220	2.59891	1.61111
C	-4.75686	3.54954	0.42708
H	-6.19086	2.23236	-0.48664
H	-3.12119	4.60871	1.33794
H	-5.36337	4.43704	0.28837
O	-3.18105	-1.08878	0.91767
C	-2.84681	-2.34678	0.80456
C	-3.77393	-3.31261	1.21384
H	-4.66048	-2.95167	1.71518
C	-3.60629	-4.65505	0.84649
H	-2.97781	-4.86492	-0.01458
H	-4.46936	-5.30248	0.93793
C	-2.31454	-5.59727	1.98840
H	-2.26511	-6.54926	1.47169
H	-1.49965	-4.91496	1.76784
C	-2.95632	-5.55352	3.21104
H	-3.56318	-6.40980	3.49883
C	-3.07797	-4.37464	4.00381
C	-2.45094	-3.22765	3.63313
H	-2.64899	-2.30054	4.16076
H	-1.64113	-3.21014	2.91305

C	-4.07182	-4.39290	5.13933
H	-5.08481	-4.56493	4.76386
H	-3.84041	-5.20172	5.83840
H	-4.06267	-3.44884	5.68634
O	-1.69612	-2.74321	0.33907
H	-1.19418	-2.05549	-0.14101

p-TS2 (S route):

C	-0.00554	-0.02716	0.02555
C	-0.00215	-0.01312	1.50100
C	0.73610	0.00390	3.77762
C	-0.58751	0.01937	4.21895
C	-1.63881	0.02332	3.29920
C	-1.32771	-0.00273	1.95274
C	1.04404	-0.00799	2.41674
H	1.54215	0.00859	4.50349
H	-0.80058	0.03737	5.28237
H	-2.67354	0.05360	3.62585
H	2.07492	-0.00962	2.08110
C	-2.28638	0.01309	0.77389
C	-1.33726	-0.03223	-0.41893
C	-1.65346	-0.06033	-1.76733
C	-0.60183	-0.09703	-2.68496
C	0.72359	-0.09241	-2.25005
C	1.03746	-0.05509	-0.89142
H	-2.68456	-0.05442	-2.10515
H	-0.81706	-0.12390	-3.74648
H	1.52127	-0.11606	-2.98329
H	2.07136	-0.04721	-0.56534
C	-3.19581	1.23026	0.72349
C	-4.47133	1.14273	0.16690
C	-2.70966	2.46894	1.14529
C	-5.25636	2.28600	0.04282
H	-4.85069	0.19074	-0.18378
C	-3.49625	3.60776	1.01473
H	-1.70805	2.55100	1.55428
C	-4.77249	3.51930	0.46518
H	-6.24040	2.21110	-0.40530
H	-3.10342	4.56892	1.32625
H	-5.37829	4.41023	0.34670
O	-3.19892	-1.13040	0.84897
C	-2.84324	-2.38999	0.76900
C	-3.75456	-3.35732	1.18306
H	-4.64194	-3.00971	1.69220
C	-3.54428	-4.70540	0.86767
H	-2.89089	-4.92855	0.02913

H	-4.38283	-5.38317	0.97879
C	-2.35926	-3.26301	3.79260
H	-2.53348	-2.38715	4.40742
H	-1.60082	-3.16969	3.02233
C	-3.00092	-4.41681	4.05910
H	-3.70175	-4.44639	4.89037
C	-2.93448	-5.59609	3.23437
C	-2.22276	-5.60833	2.05424
H	-2.13994	-6.54355	1.51144
H	-1.42403	-4.89571	1.88017
C	-3.83881	-6.73303	3.59637
H	-4.88359	-6.40336	3.56632
H	-3.71861	-7.57987	2.92150
H	-3.63739	-7.06089	4.62123
O	-1.67358	-2.76972	0.32514
H	-1.19840	-2.08539	-0.18484

p-product (R):

C	-0.00480	-0.04112	0.03766
C	-0.04023	-0.04968	1.51272
C	0.63718	-0.05829	3.80710
C	-0.69726	-0.09746	4.21354
C	-1.72433	-0.10648	3.26774
C	-1.37499	-0.08692	1.92964
C	0.98113	-0.03100	2.45580
H	1.42301	-0.04496	4.55457
H	-0.93852	-0.11212	5.27059
H	-2.76666	-0.11547	3.56922
H	2.01980	0.00432	2.14752
C	-2.29770	-0.07876	0.72390
C	-1.32346	-0.08930	-0.44457
C	-1.60353	-0.09699	-1.80157
C	-0.52708	-0.07125	-2.68976
C	0.78483	-0.02401	-2.21774
C	1.06174	-0.00631	-0.85057
H	-2.62502	-0.11789	-2.16674
H	-0.71267	-0.08019	-3.75703
H	1.60199	0.00299	-2.92907
H	2.08520	0.03851	-0.49599
C	-3.27942	1.07412	0.67057
C	-4.52416	0.92800	0.05929
C	-2.88528	2.32286	1.15298
C	-5.37292	2.02481	-0.05732
H	-4.83407	-0.03238	-0.33501
C	-3.73555	3.41560	1.02835
H	-1.90736	2.44922	1.60544

C	-4.98170	3.26901	0.42546
H	-6.33325	1.90647	-0.54539
H	-3.41571	4.38666	1.38859
H	-5.63800	4.12436	0.31477
C	-2.76751	-2.50567	0.66648
C	-3.67103	-3.54602	1.22279
C	-3.43756	-4.94038	0.64800
C	-3.45315	-3.54961	2.75508
H	-4.69216	-3.20498	1.02524
H	-2.36623	-5.16539	0.62151
H	-3.78768	-4.97999	-0.38637
H	-2.44402	-3.92353	2.95805
H	-3.52840	-2.53521	3.15290
C	-4.64219	-5.78178	2.69205
C	-5.39034	-6.84382	3.44691
H	-4.87255	-7.09532	4.37973
H	-6.39010	-6.48796	3.72055
H	-5.50061	-7.75392	2.85521
C	-4.49305	-4.45819	3.40290
H	-4.21332	-4.63945	4.44728
H	-5.46716	-3.95307	3.42909
C	-4.15454	-5.98086	1.46616
H	-4.28113	-6.95223	0.99555
O	-3.15654	-1.29760	0.80292
O	-1.65396	-2.86849	0.16814
H	-1.10988	-2.15128	-0.22935

m-product (R):

C	-0.01819	-0.07419	-0.01281
C	-0.01923	-0.10106	1.46297
C	0.70970	-0.16072	3.74084
C	-0.61547	-0.15195	4.17817
C	-1.66365	-0.11222	3.25660
C	-1.34476	-0.09224	1.91092
C	1.02346	-0.13244	2.38215
H	1.51247	-0.18615	4.46974
H	-0.83276	-0.16807	5.24039
H	-2.69837	-0.08582	3.58228
H	2.05539	-0.13403	2.05026
C	-2.29436	-0.04556	0.72717
C	-1.34886	-0.06957	-0.46376
C	-1.66119	-0.04253	-1.81372
C	-0.60571	-0.03842	-2.72718
C	0.71775	-0.04557	-2.28636
C	1.02728	-0.06080	-0.92610
H	-2.69100	-0.01972	-2.15435

H	-0.81627	-0.02171	-3.78973
H	1.51855	-0.03441	-3.01648
H	2.05973	-0.05657	-0.59578
C	-3.25157	1.12805	0.70472
C	-4.50706	1.01365	0.10861
C	-2.82598	2.36274	1.19593
C	-5.33517	2.12815	0.01539
H	-4.84006	0.06390	-0.29275
C	-3.65609	3.47354	1.09503
H	-1.83949	2.46412	1.63579
C	-4.91261	3.35867	0.50715
H	-6.30381	2.03460	-0.46152
H	-3.31228	4.43394	1.46166
H	-5.55276	4.22832	0.41465
C	-2.82350	-2.46178	0.67228
C	-3.73343	-3.48194	1.25768
C	-3.48114	-4.89479	0.73990
C	-3.55036	-3.42237	2.79263
H	-4.75160	-3.15319	1.02414
H	-2.40877	-5.11879	0.74725
H	-3.80493	-4.97410	-0.30195
H	-2.54284	-3.77840	3.03343
H	-3.64426	-2.39363	3.14604
C	-4.59773	-4.31536	3.45137
H	-4.33879	-4.45209	4.50669
H	-5.57213	-3.81127	3.43653
C	-4.20328	-5.93120	1.56880
C	-4.70458	-5.65649	2.77568
H	-5.23628	-6.43932	3.31232
C	-4.31152	-7.28608	0.93395
H	-3.32035	-7.66021	0.65807
H	-4.78467	-8.00736	1.60250
H	-4.89390	-7.23209	0.00939
O	-3.18208	-1.24539	0.81665
O	-1.73447	-2.84894	0.13982
H	-1.19163	-2.14557	-0.28276

p-product (S):

C	-0.00966	-0.01106	-0.01513
C	-0.00745	-0.02407	1.46052
C	0.72660	-0.05315	3.73747
C	-0.59745	-0.08707	4.17706
C	-1.64795	-0.08195	3.25720
C	-1.33170	-0.05237	1.91116
C	1.03713	-0.01966	2.37820
H	1.53105	-0.05085	4.46495

H	-0.81227	-0.10971	5.23965
H	-2.68268	-0.09120	3.58436
H	2.06815	0.00884	2.04459
C	-2.28414	-0.02963	0.72914
C	-1.34056	-0.04696	-0.46310
C	-1.65618	-0.04791	-1.81187
C	-0.60362	-0.02907	-2.72826
C	0.72054	0.00574	-2.29085
C	1.03325	0.01756	-0.93134
H	-2.68698	-0.05720	-2.15017
H	-0.81707	-0.03332	-3.79037
H	1.51910	0.02812	-3.02314
H	2.06601	0.05314	-0.60383
C	-3.25614	1.13257	0.70386
C	-4.51697	0.99765	0.12371
C	-2.84054	2.37658	1.18014
C	-5.36023	2.10099	0.03181
H	-4.84332	0.04078	-0.26589
C	-3.68567	3.47596	1.08037
H	-1.85051	2.49386	1.60801
C	-4.94769	3.34061	0.50869
H	-6.33340	1.99125	-0.43231
H	-3.34970	4.44346	1.43547
H	-5.60014	4.20120	0.41741
C	-2.78138	-2.45019	0.68509
C	-3.25367	-4.91029	0.93232
H	-3.10605	-5.05218	-0.13920
H	-2.29303	-5.08963	1.42589
C	-4.74214	-5.57462	2.86702
C	-5.48318	-6.66848	3.58091
H	-6.35853	-6.97647	3.00000
H	-4.84942	-7.55482	3.68838
H	-5.81696	-6.35087	4.57156
C	-4.29838	-5.88968	1.45989
H	-5.17797	-5.89656	0.80479
H	-3.89581	-6.90667	1.42903
C	-4.50084	-4.39027	3.43384
H	-4.83172	-4.20982	4.45482
O	-3.15443	-1.23824	0.82440
O	-1.67819	-2.82747	0.17603
H	-1.13453	-2.11963	-0.23888
C	-3.80762	-3.24151	2.75238
H	-4.35217	-2.31148	2.93532
H	-2.80123	-3.10698	3.17225
C	-3.70309	-3.48374	1.23017
H	-4.68223	-3.28735	0.77692

m-product (S):

C	-0.01101	-0.01197	-0.00673
C	-0.01937	-0.02466	1.46886
C	0.69920	-0.04459	3.75088
C	-0.62741	-0.09395	4.18121
C	-1.67142	-0.10122	3.25403
C	-1.34626	-0.06865	1.91013
C	1.01864	-0.00769	2.39379
H	1.49850	-0.03249	4.48391
H	-0.84940	-0.11857	5.24229
H	-2.70821	-0.12184	3.57412
H	2.05151	0.03308	2.06718
C	-2.29053	-0.05503	0.72143
C	-1.33816	-0.06396	-0.46420
C	-1.64414	-0.07109	-1.81522
C	-0.58536	-0.04090	-2.72407
C	0.73510	0.01119	-2.27720
C	1.03801	0.02848	-0.91552
H	-2.67237	-0.09398	-2.16073
H	-0.79122	-0.04964	-3.78765
H	1.53847	0.04242	-3.00389
H	2.06795	0.07683	-0.58077
C	-3.27147	1.09969	0.68961
C	-4.52914	0.95491	0.10506
C	-2.86657	2.34750	1.16531
C	-5.37997	2.05206	0.00843
H	-4.84762	-0.00473	-0.28417
C	-3.71904	3.44065	1.06058
H	-1.87900	2.47266	1.59665
C	-4.97809	3.29533	0.48473
H	-6.35064	1.93458	-0.45902
H	-3.39121	4.41112	1.41516
H	-5.63644	4.15102	0.38976
C	-2.76927	-2.47932	0.66604
C	-4.47829	-4.45155	3.41325
C	-4.70934	-5.61456	2.80152
H	-5.24161	-6.39399	3.34311
C	-4.88873	-4.17834	4.83367
H	-5.61367	-3.35843	4.87736
H	-5.33534	-5.06201	5.29448
H	-4.02305	-3.87206	5.43258
O	-3.15110	-1.27067	0.81033
O	-1.66031	-2.84672	0.16274
H	-1.11733	-2.13236	-0.24194
C	-3.78522	-3.30179	2.72325

H	-4.32664	-2.37235	2.92450
H	-2.77821	-3.17823	3.14618
C	-3.68862	-3.52224	1.19761
H	-4.66942	-3.31756	0.75211
C	-3.24109	-4.94370	0.87409
H	-2.27856	-5.13186	1.36066
H	-3.09931	-5.06706	-0.20048
C	-4.28545	-5.92868	1.39307
H	-3.88279	-6.94397	1.35027
H	-5.16375	-5.92667	0.73718

p-TS3 (R route):

C	0.00126	0.01136	-0.01495
C	0.00951	0.00617	1.46465
C	0.72857	0.00413	3.74293
C	-0.59368	-0.02587	4.17883
C	-1.64310	-0.04973	3.25506
C	-1.32265	-0.02119	1.90946
C	1.04745	0.01749	2.37974
H	1.53123	0.01463	4.47233
H	-0.81156	-0.04254	5.24060
H	-2.67615	-0.10627	3.58022
H	2.08033	0.03797	2.05181
C	-2.22125	-0.11920	0.72707
C	-1.34165	-0.04903	-0.45055
C	-1.66471	0.00705	-1.80220
C	-0.61778	0.07472	-2.72416
C	0.70509	0.11227	-2.29274
C	1.03051	0.09307	-0.92822
H	-2.69311	-0.00115	-2.14383
H	-0.83836	0.11121	-3.78391
H	1.49929	0.17367	-3.02787
H	2.06499	0.15264	-0.61040
C	-3.60279	0.38124	0.72618
C	-4.56545	-0.15632	-0.13764
C	-3.92647	1.47741	1.53377
C	-5.83511	0.39609	-0.18929
H	-4.32101	-1.01879	-0.74732
C	-5.19743	2.03785	1.46075
H	-3.17700	1.92076	2.17824
C	-6.15091	1.49812	0.60532
H	-6.57629	-0.02334	-0.85901
H	-5.43606	2.90542	2.06494
H	-7.13938	1.93871	0.54494
C	-1.92183	-2.97090	0.78139
C	-1.87253	-4.08203	1.78313

C	-1.64705	-5.46926	1.18719
C	-0.73408	-3.74039	2.76864
H	-2.82171	-4.04744	2.32523
H	-0.82256	-5.44273	0.46704
H	-2.53249	-5.78814	0.63110
H	0.21936	-3.83659	2.23931
H	-0.82325	-2.70842	3.11978
C	-0.97056	-6.14737	3.51352
C	-0.70989	-7.18343	4.57111
H	0.33182	-7.14054	4.90817
H	-1.33902	-7.00220	5.45101
H	-0.91190	-8.19129	4.20398
C	-0.78215	-4.71168	3.94512
H	0.14620	-4.63066	4.52246
H	-1.59439	-4.43278	4.63044
C	-1.34263	-6.46775	2.27216
H	-1.43992	-7.51642	2.00191
O	-2.63874	-1.98048	0.99672
O	-1.12355	-3.09838	-0.23620
H	-1.17928	-2.35964	-0.87071

m-TS3 (R route):

C	-0.00937	0.00316	0.02577
C	-0.03591	-0.02131	1.50480
C	0.62950	-0.06192	3.79860
C	-0.70132	-0.15204	4.20204
C	-1.72723	-0.17820	3.25376
C	-1.37598	-0.09838	1.91688
C	0.97972	0.00181	2.44551
H	1.41373	-0.04655	4.54772
H	-0.94260	-0.21103	5.25725
H	-2.76626	-0.27296	3.55074
H	2.01883	0.06368	2.14349
C	-2.24648	-0.17558	0.71358
C	-1.33906	-0.09593	-0.44465
C	-1.62617	-0.05075	-1.80380
C	-0.55758	0.05319	-2.69703
C	0.75161	0.13196	-2.23223
C	1.04164	0.11843	-0.85964
H	-2.64271	-0.08931	-2.17598
H	-0.75170	0.08294	-3.76214
H	1.56221	0.22078	-2.94627
H	2.06459	0.20924	-0.51295
C	-3.61949	0.34483	0.68632
C	-4.55939	-0.15249	-0.22628
C	-3.95820	1.41438	1.52295

C	-5.82085	0.41549	-0.29791
H	-4.30579	-0.99698	-0.85684
C	-5.22028	1.99137	1.42929
H	-3.22580	1.82580	2.20702
C	-6.15071	1.49261	0.52504
H	-6.54516	0.02761	-1.00412
H	-5.46988	2.83945	2.05625
H	-7.13222	1.94609	0.44858
C	-1.99163	-3.01160	0.98281
C	-2.29657	-4.16877	1.88464
C	-1.29428	-4.09264	3.05525
C	-3.73068	-4.14642	2.40087
H	-2.10624	-5.08464	1.31648
H	-1.23230	-3.06030	3.43030
H	-0.29274	-4.35146	2.69864
H	-3.87811	-3.23839	2.99556
H	-4.43478	-4.11091	1.56756
C	-3.97964	-5.37934	3.26572
H	-4.93539	-5.27754	3.78829
H	-4.07548	-6.26848	2.63101
C	-1.67723	-5.00873	4.19308
C	-2.87611	-5.59123	4.26739
H	-3.07780	-6.27050	5.09402
C	-0.60252	-5.22314	5.22199
H	-0.26583	-4.26406	5.63192
H	-0.95530	-5.85107	6.04346
H	0.27369	-5.69960	4.77046
O	-2.73807	-2.02771	0.92673
O	-0.85717	-3.11843	0.34747
H	-0.68753	-2.38642	-0.27335

p-TS3 (S route):

C	-0.00998	-0.02626	0.01126
C	-0.02317	-0.04838	1.49010
C	0.66343	-0.08295	3.77716
C	-0.66498	-0.12695	4.19398
C	-1.70101	-0.13532	3.25594
C	-1.36188	-0.08568	1.91501
C	1.00192	-0.04440	2.41955
H	1.45541	-0.08168	4.51822
H	-0.89721	-0.16319	5.25222
H	-2.73882	-0.19437	3.56514
H	2.03910	-0.01365	2.10650
C	-2.24283	-0.15425	0.71958
C	-1.34645	-0.08985	-0.44502
C	-1.64911	-0.03354	-1.80097
C	-0.58859	0.04163	-2.70658

C	0.72739	0.08383	-2.25561
C	1.03291	0.06156	-0.88618
H	-2.67094	-0.04372	-2.16047
H	-0.79348	0.07832	-3.76946
H	1.53218	0.15023	-2.97869
H	2.06222	0.12329	-0.55243
C	-3.61557	0.36461	0.70342
C	-4.56646	-0.13652	-0.19563
C	-3.94321	1.44206	1.53492
C	-5.82750	0.43355	-0.25792
H	-4.32073	-0.98523	-0.82354
C	-5.20467	2.02153	1.45003
H	-3.20221	1.85829	2.20663
C	-6.14615	1.51775	0.56006
H	-6.55996	0.04199	-0.95361
H	-5.44515	2.87576	2.07219
H	-7.12737	1.97297	0.49071
C	-1.99248	-3.02039	0.84993
C	-1.47505	-5.45631	1.37088
H	-1.73044	-5.73448	0.34709
H	-0.39546	-5.28037	1.40353
C	-1.76621	-6.15723	3.78435
C	-1.77084	-7.27194	4.79201
H	-2.65623	-7.90266	4.65802
H	-0.89925	-7.91977	4.65244
H	-1.76502	-6.89149	5.81649
C	-1.84605	-6.57899	2.33707
H	-2.86157	-6.93917	2.12895
H	-1.18582	-7.43732	2.17744
C	-1.69973	-4.87226	4.14166
H	-1.62245	-4.61634	5.19722
O	-2.72525	-2.02535	0.93665
O	-0.98122	-3.12617	0.03758
H	-0.89882	-2.37602	-0.58034
C	-1.73775	-3.71752	3.17624
H	-2.41397	-2.94247	3.54797
H	-0.74365	-3.25355	3.10256
C	-2.20103	-4.17884	1.78007
H	-3.28164	-4.35263	1.81422

m-TS3 (S route):

C	-0.00459	-0.02314	0.01775
C	-0.01285	-0.02242	1.49715
C	0.68190	-0.02768	3.78292
C	-0.64512	-0.04293	4.20470
C	-1.68463	-0.05466	3.26976

C	-1.35026	-0.03619	1.92768
C	1.01542	-0.01783	2.42304
H	1.47691	-0.02507	4.52081
H	-0.87448	-0.05586	5.26426
H	-2.72176	-0.09658	3.58413
H	2.05181	-0.00703	2.10600
C	-2.23747	-0.13036	0.73562
C	-1.34293	-0.07546	-0.43249
C	-1.65013	-0.02336	-1.78771
C	-0.59263	0.03217	-2.69830
C	0.72576	0.06026	-2.25254
C	1.03563	0.04512	-0.88451
H	-2.67418	-0.02322	-2.14202
H	-0.80153	0.06560	-3.76053
H	1.52849	0.11127	-2.97916
H	2.06689	0.09773	-0.55527
C	-3.61081	0.39200	0.71458
C	-4.56704	-0.13037	-0.16568
C	-3.93168	1.49093	1.51929
C	-5.82820	0.43914	-0.23522
H	-4.32410	-0.99309	-0.77557
C	-5.19365	2.06883	1.42805
H	-3.18632	1.92311	2.17595
C	-6.14127	1.54354	0.55729
H	-6.56465	0.03154	-0.91737
H	-5.42970	2.93854	2.03016
H	-7.12259	1.99770	0.48271
C	-1.97548	-2.97745	0.81511
C	-1.22250	-4.62901	4.15362
C	-1.23084	-5.92388	3.82984
H	-1.07175	-6.66120	4.61507
C	-0.96970	-4.13598	5.55154
H	-1.83812	-3.58677	5.93247
H	-0.75044	-4.96115	6.23327
H	-0.12369	-3.43921	5.56348
O	-2.69019	-1.97824	0.99551
O	-1.13280	-3.11697	-0.16239
H	-1.15817	-2.38490	-0.80703
C	-1.46326	-3.54393	3.13360
H	-2.16402	-2.80941	3.54229
H	-0.52319	-3.00205	2.95186
C	-2.01509	-4.10246	1.80844
H	-3.07472	-4.34380	1.94924
C	-1.24653	-5.34875	1.38136
H	-0.18452	-5.10000	1.29187
H	-1.58576	-5.69483	0.40373

C	-1.43426	-6.44253	2.43126
H	-0.73317	-7.25941	2.23993
H	-2.43677	-6.87903	2.34274

-2.57 V/nm

Carbonic cation:

C	0.00401	-0.00621	-0.00880
C	0.00271	-0.00707	1.47232
C	0.68604	-0.00215	3.75628
C	-0.62596	-0.15824	4.18985
C	-1.66303	-0.24736	3.26579
C	-1.35029	-0.13637	1.90134
C	1.02025	0.06131	2.38209
H	1.48083	0.05319	4.49196
H	-0.83781	-0.22886	5.24917
H	-2.67860	-0.41841	3.60202
H	2.05667	0.15137	2.07910
C	-2.18968	-0.23904	0.75718
C	-1.33445	-0.15831	-0.43560
C	-1.66462	-0.10681	-1.78197
C	-0.62783	0.03601	-2.71225
C	0.68929	0.15289	-2.28796
C	1.02168	0.14665	-0.92223
H	-2.69424	-0.15516	-2.11480
H	-0.85944	0.06868	-3.77041
H	1.47915	0.27045	-3.02175

H	2.04970	0.27013	-0.60195
C	-3.61790	-0.39050	0.74194
C	-4.22298	-1.25167	-0.19785
C	-4.42190	0.29838	1.67252
C	-5.59351	-1.43522	-0.18161
H	-3.60375	-1.82612	-0.87618
C	-5.79704	0.13489	1.65188
H	-3.97046	0.99528	2.36828
C	-6.38104	-0.73730	0.73569
H	-6.05328	-2.13381	-0.87051
H	-6.41345	0.67741	2.35812
H	-7.45527	-0.88229	0.74414

TS1:

C	0.00801	0.00106	0.01042
C	0.01776	0.02721	1.48844
C	0.74822	0.06881	3.75948
C	-0.56855	0.10981	4.20544
C	-1.62404	0.11249	3.29227
C	-1.31983	0.08105	1.93529
C	1.05831	0.02751	2.38989
H	1.55265	0.06678	4.48608
H	-0.77684	0.13528	5.26786
H	-2.64920	0.12192	3.64415
H	2.09139	-0.00244	2.06377
C	-2.21106	0.03228	0.77749
C	-1.33098	0.02202	-0.42185
C	-1.65318	0.07540	-1.76620
C	-0.60712	0.07047	-2.69559
C	0.71642	0.02942	-2.26990
C	1.04047	0.00287	-0.90787
H	-2.68206	0.13069	-2.10014
H	-0.83237	0.10855	-3.75524
H	1.51539	0.03322	-3.00342
H	2.07462	-0.00406	-0.58341
C	-3.58372	0.55602	0.74965
C	-4.53768	0.04586	-0.14761
C	-3.92727	1.61818	1.59709
C	-5.81171	0.58640	-0.18309
H	-4.28814	-0.80959	-0.76492
C	-5.20595	2.16527	1.53974
H	-3.19573	2.03579	2.27774
C	-6.14682	1.64943	0.65838
H	-6.55526	0.16781	-0.85144
H	-5.46484	2.98419	2.19986
H	-7.14877	2.06236	0.63662

C	-3.07167	-2.63465	1.57671
C	-2.77696	-4.06732	1.66967
H	-2.05043	-4.42947	0.95322
C	-3.34061	-4.84456	2.59233
H	-4.05447	-4.45588	3.31048
H	-3.08645	-5.89636	2.65816
O	-2.53302	-1.94182	0.69806
O	-3.91639	-2.16910	2.46124
H	-4.12950	-1.22833	2.34522

IS:

C	0.00488	0.01067	0.01038
C	0.00330	0.00341	1.48188
C	0.78096	-0.00955	3.74144
C	-0.53559	0.01237	4.19615
C	-1.60250	0.03316	3.29648
C	-1.32175	0.01936	1.93751
C	1.06366	-0.00888	2.37657
H	1.59204	-0.02263	4.45997
H	-0.73691	0.01836	5.26064
H	-2.61795	0.06159	3.67741
H	2.08968	-0.01774	2.02644
C	-2.27313	0.01430	0.74778
C	-1.31801	0.00736	-0.44267
C	-1.61954	0.01664	-1.79256
C	-0.56255	0.02440	-2.70655
C	0.75845	0.03343	-2.26076
C	1.05601	0.02823	-0.89839
H	-2.64696	0.02486	-2.13923
H	-0.77326	0.03259	-3.77029
H	1.56845	0.04914	-2.98205
H	2.08404	0.03967	-0.55428
C	-3.30279	1.12454	0.65209
C	-4.39894	0.98984	-0.20933
C	-3.12859	2.31033	1.36697
C	-5.31038	2.02894	-0.34358
H	-4.55519	0.06266	-0.75170
C	-4.04232	3.35143	1.22041
H	-2.28703	2.42391	2.04031
C	-5.13138	3.21330	0.36852
H	-6.16929	1.90895	-0.99392
H	-3.90571	4.26316	1.78964
H	-5.85034	4.01893	0.27416
C	-3.90365	-1.74450	1.40691
C	-4.21931	-3.15438	1.30082
H	-3.58986	-3.72297	0.62928

C	-5.19572	-3.69017	2.03574
H	-5.79320	-3.09548	2.71847
H	-5.40723	-4.75142	1.97620
O	-2.93712	-1.32783	0.67272
O	-4.59468	-1.01961	2.20290
H	-4.40632	-0.06093	2.19735

m-complex:

C	0.00721	0.00051	-0.03703
C	0.01928	-0.01453	1.43408
C	0.82229	-0.04304	3.68499
C	-0.48136	0.09957	4.15238
C	-1.55478	0.17380	3.26307
C	-1.29800	0.09389	1.90283
C	1.08661	-0.09025	2.31626
H	1.63901	-0.10272	4.39478
H	-0.66831	0.15337	5.21831
H	-2.55935	0.29254	3.65637
H	2.10527	-0.17608	1.95485
C	-2.26275	0.10155	0.72310
C	-1.31693	0.09423	-0.47612
C	-1.63064	0.13948	-1.82189
C	-0.58393	0.08915	-2.74694
C	0.73863	-0.00044	-2.31515
C	1.04826	-0.04431	-0.95593
H	-2.65926	0.21641	-2.15709
H	-0.80356	0.12628	-3.80825
H	1.54079	-0.03259	-3.04461
H	2.07777	-0.10949	-0.62206
C	-3.31289	1.19269	0.66162
C	-4.43390	1.03996	-0.16353
C	-3.13971	2.37978	1.37445
C	-5.36986	2.06147	-0.26446
H	-4.58749	0.11636	-0.71256
C	-4.07841	3.40252	1.26288
H	-2.27797	2.51052	2.01836
C	-5.19267	3.24586	0.44704
H	-6.24594	1.92636	-0.88849
H	-3.94118	4.31507	1.83069
H	-5.93025	4.03726	0.37960
C	-3.84922	-1.73452	1.33908
C	-4.21574	-3.10208	1.05928
C	-1.50536	-3.14747	3.05569
C	-5.22266	-3.69249	1.71187
H	-3.59673	-3.60509	0.32834
H	-1.78395	-2.57128	3.93133

H	-0.75833	-2.71965	2.39242
H	-5.82082	-3.16594	2.44745
H	-5.47645	-4.72659	1.50975
C	-3.95046	-4.27845	4.39250
H	-4.01194	-3.19531	4.32471
H	-4.66289	-4.77344	5.04322
C	-2.00141	-4.37254	2.84707
C	-3.01753	-4.96914	3.73347
H	-3.00417	-6.05443	3.81525
C	-1.55533	-5.23893	1.70027
H	-1.06093	-6.13823	2.08135
H	-2.41635	-5.57575	1.11190
H	-0.85894	-4.71012	1.04633
O	-2.91456	-1.24451	0.60561
O	-4.48758	-1.09947	2.25292
H	-4.33855	-0.13740	2.30970

p-complex:

C	0.00069	0.00932	-0.02287
C	-0.00698	-0.01811	1.44845
C	0.76821	-0.04090	3.70967
C	-0.54419	0.07243	4.16154
C	-1.60696	0.13189	3.25898
C	-1.33189	0.06255	1.90056
C	1.05037	-0.07635	2.34447
H	1.57727	-0.08702	4.42921
H	-0.74542	0.11869	5.22516
H	-2.61801	0.23031	3.64178
H	2.07499	-0.13875	1.99537
C	-2.28064	0.05921	0.70622
C	-1.31881	0.08107	-0.47920
C	-1.61598	0.13597	-1.82831
C	-0.55628	0.11714	-2.73960
C	0.76192	0.04926	-2.29084
C	1.05455	-0.00363	-0.92810
H	-2.64142	0.19870	-2.17603
H	-0.76243	0.16285	-3.80328
H	1.57417	0.04242	-3.00971
H	2.08078	-0.05069	-0.58145
C	-3.35332	1.12713	0.63611
C	-4.46815	0.94743	-0.19113
C	-3.20759	2.31971	1.34610
C	-5.42553	1.94847	-0.29808
H	-4.60106	0.01879	-0.73698
C	-4.16745	3.32178	1.22856
H	-2.35119	2.46985	1.99317

C	-5.27583	3.13860	0.40995
H	-6.29713	1.79203	-0.92343
H	-4.05203	4.23858	1.79434
H	-6.03041	3.91345	0.33832
C	-3.81240	-1.82127	1.31446
C	-4.12724	-3.20293	1.04788
C	-1.42749	-3.21479	2.98045
C	-4.99259	-3.87135	1.81864
H	-3.56956	-3.65747	0.23967
H	-2.24361	-2.76154	3.53876
H	-0.56385	-2.59133	2.77059
H	-5.50462	-3.39916	2.65073
H	-5.21524	-4.91300	1.62072
C	-2.55964	-5.43822	2.88673
C	-2.76091	-6.51079	1.85015
H	-1.85633	-7.11990	1.75371
H	-3.59235	-7.16770	2.10985
H	-2.95315	-6.06457	0.86642
C	-3.33338	-5.33911	3.97297
H	-3.14774	-4.59448	4.73998
H	-4.14636	-6.03667	4.14803
C	-1.45578	-4.49862	2.61598
H	-0.61735	-4.90219	2.04967
O	-2.90157	-1.29974	0.57280
O	-4.46685	-1.20546	2.23051
H	-4.32850	-0.24298	2.30660

m-TS2 (R route):

C	-0.00483	0.00077	0.01321
C	-0.00812	0.00659	1.48530
C	0.77143	0.02523	3.74497
C	-0.52684	0.25893	4.19111
C	-1.58646	0.35517	3.28685
C	-1.32323	0.19515	1.93582
C	1.04604	-0.08795	2.38190
H	1.57750	-0.04837	4.46565
H	-0.71882	0.37151	5.25166
H	-2.58594	0.55297	3.65883
H	2.06344	-0.23264	2.03566
C	-2.28010	0.20403	0.74798
C	-1.31846	0.15901	-0.43887
C	-1.61820	0.22231	-1.78638
C	-0.56692	0.12245	-2.70277
C	0.74540	-0.03573	-2.25902
C	1.04063	-0.09696	-0.89670
H	-2.64052	0.35428	-2.12558

H	-0.77380	0.17576	-3.76607
H	1.55157	-0.10566	-2.98144
H	2.06329	-0.21152	-0.55474
C	-3.27695	1.34330	0.65712
C	-4.44872	1.19292	-0.09279
C	-2.99525	2.57332	1.25408
C	-5.33223	2.25858	-0.22845
H	-4.68098	0.23721	-0.55173
C	-3.88040	3.63711	1.10984
H	-2.09236	2.70033	1.84037
C	-5.04943	3.48234	0.37151
H	-6.25004	2.12620	-0.79014
H	-3.66184	4.58171	1.59397
H	-5.74678	4.30733	0.28170
C	-3.79732	-1.61654	1.50720
C	-4.04954	-2.98929	1.42840
C	-1.69040	-2.94946	3.35699
C	-5.02505	-3.58325	2.20228
H	-3.40306	-3.55608	0.77304
H	-2.32793	-2.19139	3.79583
H	-0.74747	-2.61022	2.93637
H	-5.77458	-2.97498	2.69684
H	-5.28916	-4.61645	2.01943
C	-4.31346	-3.91507	4.29280
H	-4.20275	-2.84561	4.43831
H	-5.21655	-4.36240	4.69262
C	-1.99202	-4.25976	3.42584
C	-3.27016	-4.71324	3.94156
H	-3.42716	-5.78977	3.96422
C	-1.06333	-5.32220	2.90303
H	-0.82291	-6.03873	3.69306
H	-1.53888	-5.88222	2.09095
H	-0.13311	-4.88780	2.53529
O	-2.96726	-1.10208	0.62992
O	-4.41812	-0.89682	2.39923
H	-4.38841	0.06457	2.25581

p-TS2 (R route).xyz:

C	-0.03071	-0.05333	0.03214
C	-0.04554	-0.04367	1.50429
C	0.71735	-0.02437	3.77008
C	-0.58153	0.22487	4.20630
C	-1.63242	0.32973	3.29343
C	-1.36115	0.16276	1.94449
C	1.00049	-0.14660	2.40962
H	1.51751	-0.10425	4.49665

H	-0.78014	0.34296	5.26499
H	-2.63458	0.53714	3.65441
H	2.01870	-0.30454	2.07171
C	-2.30817	0.18508	0.74838
C	-1.33791	0.12535	-0.43079
C	-1.62635	0.19024	-1.78067
C	-0.56988	0.06989	-2.68850
C	0.73596	-0.10995	-2.23408
C	1.01982	-0.17226	-0.86937
H	-2.64369	0.33783	-2.12829
H	-0.76769	0.12344	-3.75349
H	1.54609	-0.19671	-2.95019
H	2.03762	-0.30414	-0.51930
C	-3.29360	1.33307	0.65110
C	-4.45800	1.19833	-0.11332
C	-3.00778	2.55668	1.25921
C	-5.33049	2.27244	-0.25131
H	-4.69339	0.24901	-0.58393
C	-3.88166	3.62939	1.11194
H	-2.11016	2.67260	1.85556
C	-5.04388	3.48958	0.36037
H	-6.24291	2.15197	-0.82427
H	-3.65951	4.56893	1.60418
H	-5.73300	4.32120	0.26847
C	-3.88868	-1.61021	1.45542
C	-4.18384	-2.97940	1.33395
C	-1.83732	-2.96358	3.38282
C	-5.16078	-3.56612	2.09258
H	-3.55133	-3.54184	0.66116
H	-2.55187	-2.23208	3.74590
H	-0.86438	-2.58567	3.08268
H	-5.85045	-2.96342	2.67260
H	-5.44694	-4.59225	1.89954
C	-3.37806	-4.88232	3.73678
C	-3.52887	-6.33816	3.41309
H	-2.77395	-6.91947	3.95122
H	-4.51521	-6.71441	3.68402
H	-3.35771	-6.50652	2.34322
C	-4.40925	-4.16496	4.24812
H	-4.28888	-3.14066	4.58252
H	-5.33205	-4.66148	4.52815
C	-2.09801	-4.27762	3.38069
H	-1.32601	-4.96589	3.04474
O	-3.00821	-1.11732	0.62442
O	-4.51877	-0.89229	2.33641
H	-4.42189	0.07137	2.24161

m-TS2 (S route):

C	-0.00131	0.00415	-0.01135
C	-0.02048	-0.02559	1.46055
C	0.72318	-0.07988	3.73116
C	-0.59840	-0.01202	4.16635
C	-1.65201	0.05091	3.25153
C	-1.35084	0.03063	1.89752
C	1.02607	-0.08068	2.37044
H	1.52264	-0.12380	4.46147
H	-0.81335	0.00017	5.22827
H	-2.66984	0.12593	3.61690
H	2.05663	-0.11955	2.03553
C	-2.29112	0.05814	0.69850
C	-1.31910	0.05576	-0.47709
C	-1.60842	0.09738	-1.82840
C	-0.54396	0.08263	-2.73412
C	0.77258	0.03641	-2.27660
C	1.05779	-0.00191	-0.91164
H	-2.63430	0.14677	-2.17853
H	-0.74401	0.11597	-3.79957
H	1.58919	0.03436	-2.99066
H	2.08286	-0.03399	-0.55962
C	-3.27332	1.21498	0.61886
C	-4.42565	1.09342	-0.16560
C	-3.01475	2.41648	1.28013
C	-5.31217	2.15967	-0.27483
H	-4.64006	0.15614	-0.66924
C	-3.90212	3.48217	1.16164
H	-2.12968	2.51904	1.89766
C	-5.05163	3.35582	0.38840
H	-6.21561	2.04932	-0.86407
H	-3.70168	4.40425	1.69458
H	-5.75167	4.18059	0.32022
O	-2.99964	-1.22864	0.57532
C	-3.75063	-1.71874	1.55175
C	-3.77166	-3.08795	1.74951
H	-3.15894	-3.68283	1.08704
C	-4.55450	-3.66319	2.75627
H	-4.84951	-3.04828	3.60154
H	-4.39598	-4.70949	2.98624
C	-6.53862	-3.69194	2.27747
H	-6.91698	-4.10004	3.20783
H	-6.59835	-2.61305	2.17646
C	-6.46072	-4.51176	1.18262
H	-6.60603	-5.57892	1.33801

C	-6.02960	-4.10135	-0.13242
C	-5.76298	-2.80813	-0.40919
H	-5.34422	-2.52476	-1.37022
H	-6.02076	-2.00119	0.26734
C	-5.75508	-5.19754	-1.12900
H	-4.97920	-5.87104	-0.75327
H	-6.65380	-5.79856	-1.29335
H	-5.42793	-4.78806	-2.08609
O	-4.50774	-0.94671	2.29068
H	-4.48828	-0.00328	2.05569

p-TS2 (S route):

C	-0.00470	-0.01929	0.00729
C	-0.02790	-0.03467	1.47917
C	0.71205	-0.06765	3.75152
C	-0.60987	0.01154	4.18377
C	-1.66118	0.06959	3.26628
C	-1.35890	0.03188	1.91294
C	1.01682	-0.08466	2.39139
H	1.51018	-0.10797	4.48348
H	-0.82673	0.03592	5.24507
H	-2.67966	0.15244	3.62855
H	2.04768	-0.13203	2.05852
C	-2.29643	0.05448	0.71090
C	-1.32073	0.03608	-0.46229
C	-1.60643	0.06657	-1.81464
C	-0.53979	0.03669	-2.71733
C	0.77528	-0.01372	-2.25596
C	1.05677	-0.04094	-0.89000
H	-2.63106	0.11838	-2.16801
H	-0.73692	0.06087	-3.78357
H	1.59362	-0.02826	-2.96787
H	2.08071	-0.07690	-0.53511
C	-3.27088	1.21677	0.61632
C	-4.41869	1.09693	-0.17543
C	-3.00850	2.42111	1.27055
C	-5.29687	2.16816	-0.29875
H	-4.63600	0.15680	-0.67242
C	-3.88765	3.49208	1.13751
H	-2.12739	2.52203	1.89394
C	-5.03242	3.36766	0.35728
H	-6.19717	2.05965	-0.89315
H	-3.68445	4.41676	1.66490
H	-5.72627	4.19663	0.27796
O	-3.00948	-1.23010	0.59279
C	-3.82642	-1.69795	1.52118

C	-3.94075	-3.07265	1.66478
H	-3.35251	-3.68275	0.99422
C	-4.78733	-3.62236	2.62379
H	-5.10091	-3.00906	3.46242
H	-4.72353	-4.68617	2.82091
C	-5.94752	-2.54685	-0.56237
H	-5.55421	-2.24791	-1.52853
H	-6.10424	-1.76011	0.16765
C	-6.28150	-3.82508	-0.32714
H	-6.14270	-4.55830	-1.11821
C	-6.71781	-4.34806	0.95597
C	-6.82120	-3.55407	2.06568
H	-7.21175	-3.97767	2.98443
H	-6.83885	-2.47186	1.99359
C	-6.89724	-5.83048	1.05062
H	-5.96231	-6.33912	0.78978
H	-7.20179	-6.13852	2.05002
H	-7.65255	-6.16213	0.33088
O	-4.56497	-0.91219	2.26228
H	-4.48545	0.03828	2.07247

p-product (R route):

C	-0.01072	-0.03375	0.00793
C	-0.02323	-0.04396	1.47979
C	0.73610	-0.06780	3.74555
C	-0.58378	-0.04156	4.19047
C	-1.64354	-0.01194	3.28242
C	-1.35168	-0.01935	1.92561
C	1.02967	-0.06562	2.38289
H	1.54174	-0.08488	4.47013
H	-0.79259	-0.03676	5.25353
H	-2.66247	0.02115	3.65468
H	2.05844	-0.07866	2.04101
C	-2.29293	-0.00479	0.72949
C	-1.33030	-0.01372	-0.45381
C	-1.62460	-0.00688	-1.80506
C	-0.56257	-0.02555	-2.71285
C	0.75567	-0.04050	-2.25903
C	1.04570	-0.04247	-0.89486
H	-2.65018	0.01593	-2.15676
H	-0.76705	-0.02283	-3.77781
H	1.56976	-0.04878	-2.97578
H	2.07194	-0.05268	-0.54549
C	-3.32018	1.10772	0.64053
C	-4.41200	0.98641	-0.22894
C	-3.14995	2.28331	1.37270

C	-5.32364	2.02666	-0.35140
H	-4.56637	0.06862	-0.78782
C	-4.06295	3.32656	1.23680
H	-2.31144	2.38801	2.05111
C	-5.14867	3.20041	0.37895
H	-6.17959	1.91567	-1.00722
H	-3.92839	4.23036	1.81901
H	-5.86776	4.00690	0.29345
C	-3.94631	-1.77151	1.32775
C	-4.26822	-3.21116	1.19990
C	-3.34294	-3.97510	2.17658
C	-5.73037	-3.53695	1.49988
H	-4.00217	-3.50101	0.17894
H	-3.41915	-3.53744	3.17911
H	-2.30230	-3.86968	1.85856
H	-5.93023	-3.33068	2.55459
H	-6.39773	-2.90787	0.90577
C	-4.89533	-5.91144	1.76677
C	-5.22873	-7.37442	1.81289
H	-6.07778	-7.55233	2.48014
H	-5.52236	-7.73383	0.81994
H	-4.38561	-7.97033	2.16524
C	-5.97366	-5.01442	1.20519
H	-6.93725	-5.31556	1.62757
H	-6.05006	-5.18132	0.12207
C	-3.73384	-5.42825	2.21179
H	-3.00401	-6.10457	2.64895
O	-2.96470	-1.34492	0.62931
O	-4.64232	-1.07272	2.13521
H	-4.43936	-0.11696	2.18318

m-product (R route):

C	-0.01076	-0.03583	0.01084
C	-0.02278	-0.04102	1.48274
C	0.73713	-0.05724	3.74839
C	-0.58268	-0.03204	4.19359
C	-1.64271	-0.00640	3.28570
C	-1.35114	-0.01691	1.92886
C	1.03037	-0.05835	2.38564
H	1.54300	-0.07097	4.47279
H	-0.79122	-0.02473	5.25669
H	-2.66162	0.02622	3.65814
H	2.05908	-0.07073	2.04359
C	-2.29274	-0.00720	0.73304
C	-1.33054	-0.01857	-0.45052
C	-1.62532	-0.01659	-1.80167

C	-0.56357	-0.03752	-2.70974
C	0.75483	-0.05013	-2.25634
C	1.04535	-0.04715	-0.89226
H	-2.65104	0.00408	-2.15309
H	-0.76843	-0.03861	-3.77464
H	1.56866	-0.06047	-2.97334
H	2.07172	-0.05559	-0.54324
C	-3.32194	1.10314	0.64121
C	-4.41376	0.97716	-0.22751
C	-3.15394	2.28104	1.37028
C	-5.32759	2.01520	-0.35255
H	-4.56639	0.05733	-0.78343
C	-4.06919	3.32197	1.23187
H	-2.31550	2.38922	2.04825
C	-5.15486	3.19125	0.37460
H	-6.18356	1.90055	-1.00770
H	-3.93647	4.22757	1.81169
H	-5.87568	3.99598	0.28719
C	-3.94157	-1.77502	1.33956
C	-4.25602	-3.21773	1.22502
C	-3.33785	-3.95975	2.22441
C	-5.72055	-3.54674	1.51034
H	-3.97556	-3.51909	0.21140
H	-3.43010	-3.51305	3.22205
H	-2.29224	-3.85150	1.92016
H	-5.93486	-3.32792	2.55970
H	-6.38247	-2.92920	0.89830
C	-5.94874	-5.03054	1.23262
H	-6.91411	-5.33175	1.64815
H	-6.01339	-5.20580	0.15045
C	-3.70227	-5.42440	2.29362
C	-4.86287	-5.88924	1.82660
H	-5.06594	-6.95462	1.90383
C	-2.67188	-6.29663	2.94900
H	-2.45265	-5.93470	3.95833
H	-3.01303	-7.33006	3.02321
H	-1.72998	-6.27370	2.39129
O	-2.96197	-1.34912	0.63788
O	-4.64016	-1.07362	2.14261
H	-4.43959	-0.11707	2.18572

p-product (S route):

C	0.00743	0.04030	0.10477
C	-0.09916	0.04179	1.57623
C	0.46518	-0.03345	3.89650
C	-0.88511	0.04703	4.23994

C	-1.86733	0.13508	3.25233
C	-1.45554	0.12461	1.92870
C	0.87497	-0.03446	2.56303
H	1.20769	-0.09386	4.68364
H	-1.17200	0.04746	5.28452
H	-2.91714	0.21111	3.51593
H	1.92758	-0.09070	2.31007
C	-2.30662	0.23392	0.68039
C	-1.27927	0.11919	-0.43937
C	-1.49683	0.11874	-1.80492
C	-0.38571	0.03055	-2.64595
C	0.90158	-0.04116	-2.11241
C	1.11315	-0.03562	-0.73378
H	-2.50203	0.19338	-2.20761
H	-0.52442	0.02684	-3.72137
H	1.75442	-0.10010	-2.77978
H	2.11606	-0.09119	-0.32585
C	-3.14169	1.49771	0.57691
C	-4.33347	1.51621	-0.14979
C	-2.66709	2.67460	1.15662
C	-5.04811	2.70309	-0.28079
H	-4.72078	0.60620	-0.59156
C	-3.38275	3.85871	1.01540
H	-1.74801	2.67142	1.73219
C	-4.57550	3.87590	0.29952
H	-5.98745	2.70400	-0.82233
H	-3.01585	4.76220	1.48801
H	-5.14333	4.79523	0.21407
C	-3.09086	-2.11576	0.76304
C	-3.69362	-4.45160	0.06255
H	-2.86731	-4.43104	-0.65227
H	-3.32427	-4.90350	0.98684
C	-6.12720	-5.09423	0.32426
C	-7.20995	-6.10076	0.06015
H	-7.46848	-6.11727	-1.00520
H	-6.87090	-7.10799	0.32301
H	-8.11181	-5.88176	0.63361
C	-4.86204	-5.26741	-0.48369
H	-5.06326	-4.99396	-1.52872
H	-4.59194	-6.32840	-0.49339
C	-6.26229	-4.11849	1.22419
H	-7.17416	-4.05439	1.81191
O	-3.28606	-0.87658	0.53120
O	-2.05701	-2.61322	1.31759
H	-1.42913	-1.96682	1.71121
C	-5.22166	-3.07033	1.51169

H	-5.69339	-2.08998	1.61488
H	-4.71791	-3.28559	2.46150
C	-4.18459	-3.03557	0.36598
H	-4.65883	-2.59278	-0.51483

m-product (S route):

C	0.01252	0.01069	0.02738
C	-0.03732	-0.03841	1.49794
C	0.65734	-0.12265	3.78250
C	-0.67492	-0.12559	4.19037
C	-1.70927	-0.07936	3.25391
C	-1.37697	-0.04002	1.90821
C	0.99007	-0.07604	2.42948
H	1.44212	-0.15278	4.52923
H	-0.91257	-0.15689	5.24690
H	-2.73822	-0.06928	3.59726
H	2.02816	-0.06796	2.11705
C	-2.28596	0.00934	0.69112
C	-1.29515	0.03065	-0.46781
C	-1.55588	0.06480	-1.82556
C	-0.47112	0.07557	-2.70623
C	0.83535	0.06306	-2.21911
C	1.09120	0.03221	-0.84841
H	-2.57299	0.08620	-2.20195
H	-0.64846	0.10043	-3.77574
H	1.66722	0.07907	-2.91496
H	2.10839	0.02331	-0.47336
C	-3.30839	1.12673	0.61165
C	-4.37922	1.03378	-0.28684
C	-3.15443	2.27885	1.38322
C	-5.28881	2.07723	-0.39687
H	-4.51941	0.13657	-0.88313
C	-4.06456	3.32616	1.25922
H	-2.33164	2.36259	2.08359
C	-5.13102	3.22720	0.37407
H	-6.12886	1.98786	-1.07618
H	-3.94322	4.21133	1.87211
H	-5.84814	4.03635	0.29791
C	-3.99775	-1.75205	1.14724
C	-5.65257	-4.34259	-1.06088
C	-5.99984	-5.27030	-0.16726
H	-6.46180	-6.18987	-0.52044
C	-5.90116	-4.49123	-2.53614
H	-4.95730	-4.48645	-3.09294
H	-6.43115	-5.42002	-2.75673
H	-6.49979	-3.65349	-2.91229

O	-2.96121	-1.32448	0.53833
O	-4.70966	-1.09007	1.96930
H	-4.48306	-0.14674	2.09600
C	-4.98163	-3.05149	-0.65719
H	-4.16069	-2.82878	-1.34703
H	-5.70285	-2.22681	-0.73835
C	-4.43910	-3.12656	0.78754
H	-3.53954	-3.75046	0.79024
C	-5.49550	-3.69642	1.72972
H	-6.40170	-3.08723	1.66130
H	-5.14893	-3.66086	2.76351
C	-5.80249	-5.13409	1.31742
H	-6.70020	-5.47804	1.83699
H	-4.99468	-5.80061	1.64144

p-TS3 (R route):

C	0.01333	0.06857	0.00890
C	0.01400	0.03355	1.48694
C	0.73049	-0.00447	3.76308
C	-0.58929	-0.02497	4.20122
C	-1.63994	-0.02173	3.28139
C	-1.32713	0.01152	1.92609
C	1.04904	0.02734	2.39517
H	1.53054	-0.01022	4.49459
H	-0.80439	-0.04740	5.26244
H	-2.66725	-0.06021	3.62742
H	2.08428	0.04932	2.07505
C	-2.20959	-0.05082	0.75913
C	-1.32248	0.04157	-0.43158
C	-1.64318	0.11423	-1.77465
C	-0.59420	0.18856	-2.69781
C	0.72823	0.20076	-2.26566
C	1.04847	0.14822	-0.90338
H	-2.67428	0.11924	-2.10801
H	-0.81575	0.24392	-3.75749
H	1.52885	0.26432	-2.99461
H	2.08068	0.17793	-0.57385
C	-3.61308	0.38708	0.74672
C	-4.53882	-0.17083	-0.14965
C	-4.01039	1.42504	1.59857
C	-5.84030	0.30108	-0.18188
H	-4.23991	-1.00442	-0.77463
C	-5.31680	1.90308	1.54602
H	-3.30079	1.87530	2.28194
C	-6.23043	1.34173	0.66365
H	-6.56127	-0.15341	-0.85159

H	-5.61798	2.70396	2.21036
H	-7.25272	1.70139	0.64457
C	-2.60329	-2.74476	1.55639
C	-1.80216	-3.98827	1.75269
C	-0.37859	-3.51499	2.12460
C	-2.34490	-4.93700	2.81587
H	-1.75167	-4.48653	0.77827
H	-0.43572	-2.77358	2.93470
H	0.07256	-3.00591	1.26698
H	-2.31356	-4.43615	3.78767
H	-3.38722	-5.19327	2.61304
C	-0.00055	-5.88904	2.86460
C	0.89507	-7.02184	3.27352
H	0.69272	-7.31202	4.30900
H	0.70437	-7.90582	2.65526
H	1.95014	-6.75574	3.19068
C	-1.47991	-6.19393	2.85956
H	-1.72307	-6.77521	3.75391
H	-1.70650	-6.84602	2.00539
C	0.47316	-4.68151	2.54816
H	1.54283	-4.49542	2.60975
O	-2.39367	-2.00884	0.57648
O	-3.45463	-2.45615	2.49910
H	-3.95632	-1.63781	2.33263

m-TS3 (R route):

C	0.01112	0.05171	-0.03147
C	0.02448	0.03391	1.44677
C	0.76016	0.01939	3.71706
C	-0.55573	-0.01163	4.16644
C	-1.61398	-0.02857	3.25518
C	-1.31268	-0.00028	1.89723
C	1.06713	0.04374	2.34629
H	1.56628	0.02744	4.44197
H	-0.76192	-0.02889	5.22952
H	-2.63776	-0.07976	3.60987
H	2.09952	0.07251	2.01727
C	-2.20456	-0.08468	0.73836
C	-1.32779	0.00398	-0.46054
C	-1.66024	0.05435	-1.80177
C	-0.61970	0.12706	-2.73451
C	0.70601	0.15965	-2.31357
C	1.03787	0.12979	-0.95341
H	-2.69406	0.04232	-2.12643
H	-0.85040	0.16427	-3.79303
H	1.49994	0.22089	-3.05001

H	2.07244	0.17447	-0.63294
C	-3.61167	0.34203	0.73305
C	-4.54092	-0.23670	-0.14632
C	-4.00953	1.38872	1.57363
C	-5.84633	0.22453	-0.17383
H	-4.24098	-1.07729	-0.76137
C	-5.32008	1.85586	1.52558
H	-3.29761	1.85336	2.24493
C	-6.23702	1.27472	0.65952
H	-6.56974	-0.24577	-0.82986
H	-5.62193	2.66372	2.18113
H	-7.26224	1.62608	0.64437
C	-2.55913	-2.77046	1.56739
C	-1.72478	-3.99074	1.77485
C	-0.31411	-3.46447	2.12053
C	-2.22490	-4.93684	2.86067
H	-1.67115	-4.50353	0.80827
H	-0.38160	-2.70767	2.91550
H	0.11476	-2.96102	1.24697
H	-2.19990	-4.41731	3.82291
H	-3.25957	-5.23237	2.67306
C	-1.31403	-6.16193	2.91629
H	-1.52716	-6.73354	3.82310
H	-1.53206	-6.83433	2.07641
C	0.60308	-4.57498	2.57023
C	0.14343	-5.78311	2.90162
H	0.85322	-6.54366	3.21836
C	2.05411	-4.19494	2.64653
H	2.18742	-3.33962	3.31785
H	2.66514	-5.01833	3.01925
H	2.43175	-3.89162	1.66343
O	-2.37451	-2.04348	0.57525
O	-3.40602	-2.48520	2.51445
H	-3.92637	-1.68016	2.33943

p-TS3 (S route):

C	-0.04329	-0.00075	-0.05677
C	-0.01966	-0.02859	1.42054
C	0.72764	-0.06381	3.68620
C	-0.58796	-0.09321	4.13913
C	-1.65123	-0.08866	3.23270
C	-1.34951	-0.03953	1.87999
C	1.02968	-0.03352	2.31665
H	1.53577	-0.06593	4.40859
H	-0.78877	-0.12195	5.20311
H	-2.67704	-0.13088	3.58196

H	2.06065	-0.01064	1.98285
C	-2.26150	-0.07657	0.72454
C	-1.38654	0.00342	-0.47991
C	-1.72233	0.08884	-1.81963
C	-0.68667	0.08450	-2.75924
C	0.64193	0.03576	-2.34535
C	0.98023	0.01448	-0.98726
H	-2.75572	0.14621	-2.14011
H	-0.92140	0.12585	-3.81676
H	1.43289	0.03449	-3.08766
H	2.01781	0.00970	-0.67378
C	-3.61130	0.51580	0.72978
C	-4.59758	0.06724	-0.16018
C	-3.88467	1.58561	1.58610
C	-5.84053	0.67782	-0.18177
H	-4.39880	-0.78800	-0.79699
C	-5.13132	2.20492	1.54588
H	-3.12693	1.95011	2.26882
C	-6.10844	1.75093	0.67031
H	-6.61195	0.30725	-0.84708
H	-5.33835	3.02934	2.21757
H	-7.08575	2.21962	0.66186
C	-2.33169	-2.95646	0.48649
C	-0.63282	-4.42729	-0.70333
H	-0.04088	-4.46663	0.21281
H	-1.27824	-5.31005	-0.71855
C	-0.44688	-4.06882	-3.20511
C	0.30740	-4.32981	-4.47843
H	1.26201	-3.79185	-4.47096
H	0.54503	-5.39451	-4.57562
H	-0.26185	-4.01610	-5.35688
C	0.28009	-4.41969	-1.92848
H	1.09853	-3.70181	-1.78327
H	0.75561	-5.39902	-2.04527
C	-1.67627	-3.54728	-3.19843
H	-2.16775	-3.32658	-4.14441
O	-2.79169	-1.87430	0.87875
O	-2.65173	-4.05191	1.11668
H	-3.20038	-3.86490	1.90152
C	-2.44496	-3.21636	-1.94691
H	-2.95060	-2.25406	-2.06452
H	-3.22731	-3.96461	-1.76764
C	-1.49368	-3.16566	-0.73658
H	-0.84157	-2.29919	-0.84991

m-TS3 (S route):

C	0.07717	0.02839	0.07108
C	0.04314	0.06842	1.54934
C	0.70071	0.15122	3.84106
C	-0.63020	0.18373	4.24534
C	-1.65662	0.16083	3.29973
C	-1.30710	0.11150	1.95541
C	1.05500	0.09349	2.48318
H	1.48158	0.17087	4.59270
H	-0.87042	0.22498	5.30053
H	-2.69389	0.17198	3.61834
H	2.09830	0.07330	2.19059
C	-2.16249	0.05431	0.77300
C	-1.24827	0.04552	-0.40028
C	-1.53564	0.06956	-1.75270
C	-0.46466	0.04299	-2.65288
C	0.84664	0.00809	-2.18956
C	1.13424	0.00897	-0.81889
H	-2.55669	0.11394	-2.11246
H	-0.66034	0.05579	-3.71899
H	1.66515	-0.00832	-2.90101
H	2.15940	0.00154	-0.46704
C	-3.53504	0.57359	0.71334
C	-4.47420	0.04829	-0.18932
C	-3.89268	1.64942	1.53611
C	-5.75043	0.58232	-0.24933
H	-4.21373	-0.80890	-0.79968
C	-5.17137	2.19252	1.45206
H	-3.17162	2.08113	2.21902
C	-6.10052	1.65763	0.56944
H	-6.48293	0.15032	-0.92138
H	-5.43995	3.02323	2.09344
H	-7.10357	2.06638	0.52837
C	-3.20673	-2.67449	1.26164
C	-2.33753	-5.95245	-0.55117
C	-3.53687	-6.53885	-0.55841
H	-3.62784	-7.54536	-0.96196
C	-1.10845	-6.61044	-1.11113
H	-0.37090	-6.78137	-0.32064
H	-1.34227	-7.56943	-1.57802
H	-0.63014	-5.96328	-1.85541
O	-2.46941	-1.90562	0.63961
O	-4.02135	-2.32234	2.22028
H	-4.01222	-1.37495	2.43508
C	-2.11780	-4.57937	0.03848
H	-1.19124	-4.57960	0.62152
H	-1.96616	-3.85851	-0.77446

C	-3.28274	-4.13475	0.91746
H	-3.27727	-4.67878	1.86820
C	-4.62610	-4.40703	0.21419
H	-4.62418	-3.88498	-0.75056
H	-5.45383	-4.01857	0.80990
C	-4.78713	-5.90897	-0.00637
H	-5.62487	-6.09165	-0.68658
H	-5.05407	-6.39467	0.93948

References

1. P. Zhang, V. Nguyen, & J. W. Frost, Synthesis of terephthalic acid from methane. *ACS Sustain. Chem. Eng.* **4**, 5998 (2016).
2. Yang, C. *et al.* Electric field–catalyzed single-molecule Diels-Alder reaction dynamics. *Sci. Adv.* **7**, eabf0689 (2021).
3. Yang, C. *et al.* Unveiling the full reaction path of the Suzuki–Miyaura cross-coupling in a single-molecule junction. *Nat. Nanotechnol.* **16**, 1214−1223 (2021).
4. L. Zhang, *et al.* Precise electrical gating of the single-molecule Mizoroki-Heck reaction. *Nat. Commun.* **13**, 4552 (2022).
5. E. K. Martin, and A. H. Deane, Photochemical cyclization of olefinic N-chloroamides. *J. Org. Chem.* **40**, 1287 (1975).
6. Jin, & R. M. Coates, Enantioselective synthesis of α-terpineol and nephthenol by intramolecular acyloxazolidinone enolate alkylations. *Chem. Commun.* **27**, 2902−2904, (2006).
7. T. Nagata *et al*. Discovery of N-[(1R,2S,5S)-2-{[(5-chloroindol-2-yl)carbonyl]amino}-5-(dimethylcarbamoyl)cyclohexyl]-5-methyl-4,5,6,7-tetrahydrothiazolo[5,4-c]pyridine-2-carboxamide hydrochloride: A novel, potent and orally active direct inhibitor of factor Xa. *Bioorgan. Med. Chem.* **17**, 1193−1206 (2009).
8. K. Miyashita, T. Tsunemi, T. Hosokawa, M. Ikejiri & T. Imanishi, Total synthesis of leustroducsin B. *J. Org. Chem.* **73**, 5360−5370 (2008). doi: 10.1021/jo8005599
9. C. Palomo *et al*. A chiral acrylate equivalent for metal-free Diels−Alder reactions: endo-2-acryloylisoborneol. *J. Am. Chem. Soc.* **124**, 10288−10289 (2002).
10. T. Poll, A. Sobczak, H. Hartmann & G. Helmchen, Diastereoface-discriminative metal coordination in asymmetric synthesis: D-pantolactone as practical chiral auxiliary for Lewis acid catalyzed Diels-Alder reactions. *Tetrahedron Lett.* **26**, 3095−3098 (1985).
11. R. Akkari *et al*. (R)- or (S)-4-(3-hydroxy-4,4-dimethyl-2-oxopyrrolidin-1-yl)benzoic acid as a new chiral auxiliary for solid phase asymmetric Diels–Alder reactions. *Tetrahedron: Asymmetry* **15**, 2515−2525 (2004).
12. J. Klepp, C. J. Sumby, & B. W. Greatrex, Synthesis of a chiral auxiliary family from levoglucosenone and evaluation in the Diels–Alder reaction. *Synlett* **29**, 1441−1446 (2018).
